# Chromium-catalyzed stereodivergent *E*- and *Z*-selective alkyne hydrogenation controlled by cyclic (alkyl)(amino)carbene ligands

**DOI:** 10.1038/s41467-023-36677-9

**Published:** 2023-02-22

**Authors:** Liang Ling, Chenyang Hu, Linhong Long, Xue Zhang, Lixing Zhao, Liu Leo Liu, Hui Chen, Meiming Luo, Xiaoming Zeng

**Affiliations:** 1 Key Laboratory of Green Chemistry & Technology, Ministry of Education, College of Chemistry, Sichuan University, 610064 Chengdu, China; 2 Shenzhen Grubbs Institute and Department of Chemistry, Southern University of Science and Technology, 518055 Shenzhen, China; 3 Beijing National Laboratory for Molecular Sciences (BNLMS), Key Laboratory of Photochemistry, CAS Research/Education Center for Excellence in Molecular Sciences, Institute of Chemistry, Chinese Academy of Sciences, 100190 Beijing, China

**Keywords:** Synthetic chemistry methodology, Stereochemistry, Homogeneous catalysis

## Abstract

The hydrogenation of alkynes allows the synthesis of olefins, which are important feedstock for the materials, pharmaceutical, and petrochemical industry. Thus, methods that enable this transformation via low-cost metal catalysis are desirable. However, achieving stereochemical control in this reaction is a long-standing challenge. Here, we report on the chromium-catalyzed *E*- and *Z*-selective olefin synthesis via hydrogenation of alkynes, controlled by two carbene ligands. A cyclic (alkyl)(amino)carbene ligand that contains a phosphino anchor enables the hydrogenation of alkynes in a *trans*-addition manner, selectively forming *E*-olefins. With an imino anchor-incorporated carbene ligand, the stereoselectivity can be switched, giving mainly *Z*-isomers. This ligand-enabled geometrical stereoinversion strategy by one metal catalysis overrides common methods in control of the *E*- and *Z*-selectivity with two different metal catalysis, allowing for highly efficient and on-demand access to both *E*- and *Z*-olefins in a stereo-complementary fashion. Mechanistic studies indicate that the different steric effect between these two carbene ligands may mainly dominate the selective forming *E*- or *Z*-olefins in control of the stereochemistry.

## Abstract (web summary)

The stereoselective synthesis of olefins from alkynes is a long-standing challenge in organic synthesis. Here, the authors report the chromium-catalyzed alkyne hydrogenation; *E*- or *Z*-olefins are obtained depending on the carbene ligand used.

## Introduction

**Fig. 1:**
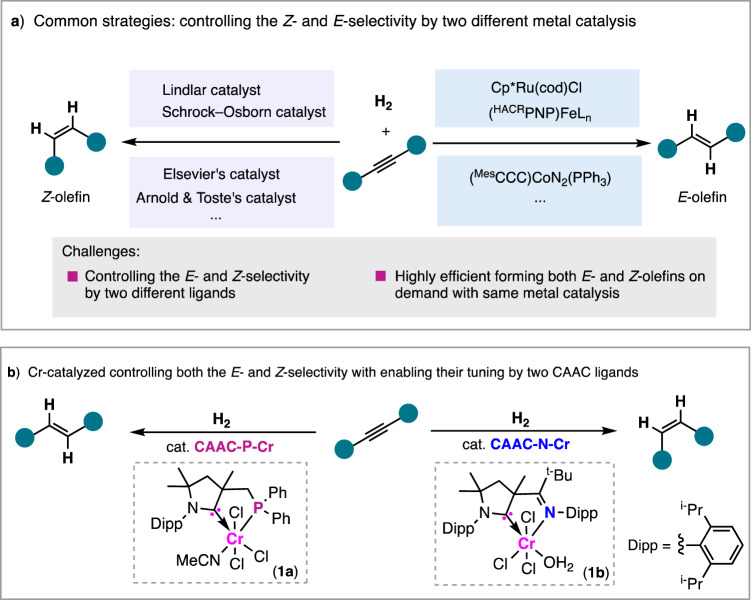
Controlling the stereochemistry in alkyne hydrogenation for highly selective formation of *E*- and *Z*-olefins. **a** Commonly used strategy in the control of the *E*- and *Z*-selectivity by two different metal catalysis. **b** Controlling the *E*- and *Z*-selectivity in the hydrogenation of alkynes by two CAAC ligands. CAAC cyclic (alkyl)(amino)carbene, CAAC-P–Cr CAAC-phosphino-ligated Cr complex **1a**, CAAC-N–Cr CAAC-imino-ligated Cr complex **1b**.

Olefins are among the most useful chemicals in industry, serving as feedstocks in the large-scale production of high-value-added polymers, fibers, food, pharmaceuticals, and agrochemicals^1^. An important challenge in the construction of olefins is the creation of the carbon–carbon double bonds in a defined manner of selectivity and stereochemistry, i.e., the *E*- versus *Z*-isomer^2,3^. This is particularly important because of the close relationship between geometry and function, as well as reducing the effort required to separate these isomers (a difficult task)^4^. Although several strategies to access olefins have been established, e.g., the classic Wittig, Peterson, and Takai olefinations, control of the *E*/*Z* selectivity in olefin formation has long been challenging^5^. Transition-metal catalysis has the ability to afford high selectivity, as evident in transformations of H_2_-semihydrogenation, in the creation of olefinic C–C bonds^6^. Pd-based Lindlar catalyst has been commonly used in industry for alkyne semihydrogenation in the selective production of Z-olefins^7^. Recently, the notable H_2_-semihydrogenation of alkynes with homogeneous metal catalysts reported by Elsevier^8^, Arnold^9^, and Toste^10^ allows for selectively accessing *Z*-olefins^11–14^. When ruthenium, iron, and cobalt complexes are used, the *E*-isomers can be predominantly formed with high selectivity (Fig. 1a)^15–37^. Compared with the achievement of either *E*- or *Z*-selectivity, control of both the *E*- and *Z*-selectivity by two different ligands with the same metal catalysis has proved to be difficult. There are a few systems that depend on transfer hydrogenation with borrowing a hydride^38^; however, because hydride sources generate large amounts of waste, in light of increasing demands for sustainability, the hydride-borrowing strategies are impractical for the preparation of olefins on a large scale.

In this work, we describe progress made in addressing the key stereochemical challenges in using two ligands in control of the selectivity in *E*- and *Z*-olefin formation by the hydrogenation of alkynes with molecular hydrogen (Fig. 1b). We develop a bidentate metal catalyst with earth-abundant chromium by chelation with a cyclic (alkyl)(amino)carbene (CAAC) ligand that contains a phosphino anchor, and accomplishes the *trans*-hydrogenation of alkynes in selectively giving *E*-olefins^39–41^. Building on this foundation, we establish that tuning the stereoselectivity achieves a bulky imino-incorporated CAAC ligand, resulting in the predominant formation of *Z*-olefins by Cr catalysis.

## Results

### Reaction development

**Fig. 2:**
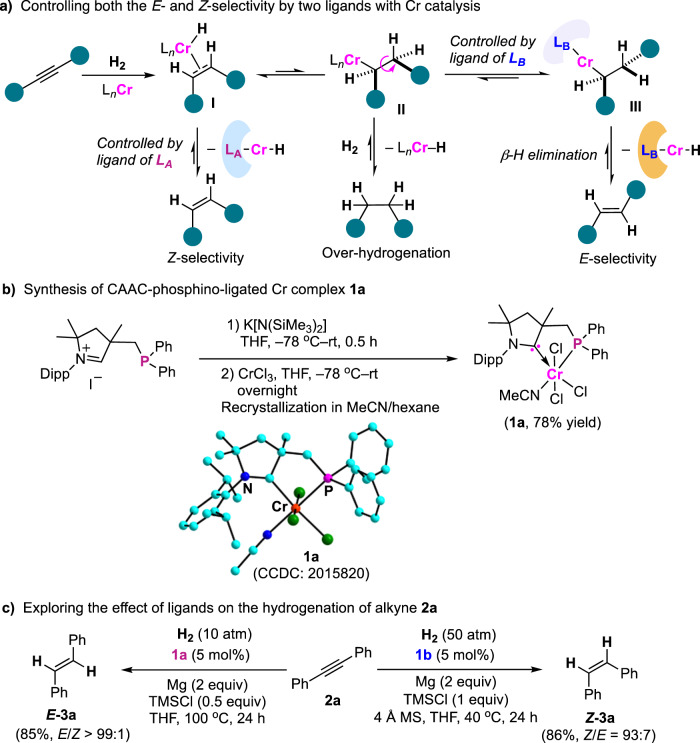
Hypothesis for controlling both the *E*- and *Z*-selectivity by two ligands with Cr catalysis and CAAC-controlled stereoselective hydrogenation of alkynes. **a** Working hypothesis. **b** Preparing complex **1a**. **c** Exploring the ligand effect on controlling the *E*/*Z* selectivity. The stereoselectivity of *E*-olefin relative to *Z*-stereomer was determined by GC analysis.

Metal-catalyzed hydrogenation of alkynes with H_2_ provides a clean and sustainable strategy for the formation of olefins, featuring 100% atom efficiency^42–47^. Controlling both the *E*- and *Z*-selectivity in alkyne hydrogenation by two different ligands is of great interest, which allows us to access on demand both *E*- and *Z*-olefins in high efficiency with the same metal catalysis. We anticipated that the hydrogenation of alkynes by low-cost chromium catalysts could occur by the *cis*-addition of hydrogen to C–C triple bonds, to form *Z*-olefins, which can be potentially attacked by a Cr–H species, to afford the alkylated chromate intermediates **II** (Fig. 2a)^48^. Subsequent transformations would involve at least three possible pathways, specifically, the reversible *β*-hydride elimination, rotation of C–C bonds and following *β*-hydride elimination, and straightforward reductive elimination, thus resulting in the formation of *Z*-olefins, *E*-isomers, and over-hydrogenated alkanes, respectively. However, controlling the stereochemistry of hydrogenation by two ligands for forming both *Z*- and *E*-olefins in high efficiency has been a long-standing challenge with the same metal catalysis. It may be due to the lack of effective ligands by the same metal catalysis to address the two critical mechanistic issues: (a) inhibiting rotation of the C–C bond of alkylated metal complexes **II** for selectively accessing a *Z*-olefin, and (b) facilitating the C–C bond rotation in the main formation of an *E*-olefin. In addition, the inhibition of reductive elimination of metal hydride **II** is indispensable for avoiding over-hydrogenation during the processes.

### Catalyst development

Cyclic (alkyl)(amino)carbenes (CAACs) are more nucleophilic and electrophilic than more commonly used *N*-heterocyclic carbenes (NHCs)^39,49^. The use of CAACs as ligands in promoting the Rh-catalyzed stereoselective hydrogenation of arenes has been reported^50–53^, as well as in assisting the Cr-catalyzed deoxygenative hydroboration of nitro motifs^54^. Given the different electronic properties and steric circumstances between phosphino and imino substituents, the installation of diphenylphosphino motif within the side chain of CAAC ligand led to bidentate complex **1a** with Cr (Fig. 2b)^55^. Its structure was confirmed by X-ray diffraction analysis, showing an octahedral geometry at Cr in coordination with the phosphino CAAC ligand, three chloride ligands, and an additional acetonitrile ligand.

### Exploring the effect of ligands on hydrogenation of alkynes

We explored the effect of CAAC ligands of Cr complexes on the *E*/*Z* selectivity in catalytic hydrogenation of diphenylethyne, with magnesium as a reductant, for forming reactive Cr in situ^56–62^. The phosphino-containing complex **1a** has the ability to promote the addition of hydrogen to a triple bond in a *trans*-selective manner, affording *E*-stilbene (***E*-3a**) with up to 99:1 *E*/*Z* selectivity (Fig. 2c). By contrast, complex **1b** that contains an imino anchor in a CAAC ligand allows for controlling the stereoselectivity of hydrogen addition to affording 99:1 *Z*/*E* selectivity. The replacement of the 2,6-di-isopropylphenyl substituent of the imino in **1b** by a phenyl (**1c**) led to low conversion and *Z*-selectivity (see Supplementary Information). Performing the hydrogenation with complex **1b** at ambient temperature and high pressure did not lead to hexaphenylbenzene, giving high conversion and selectivity of *Z*-stilbene. In these cases, only trace amounts of over-reduced 1,2-diphenylethane were detected.

### Scope of CAAC-P ligand-enabled *trans*-hydrogenation

**Fig. 3:**
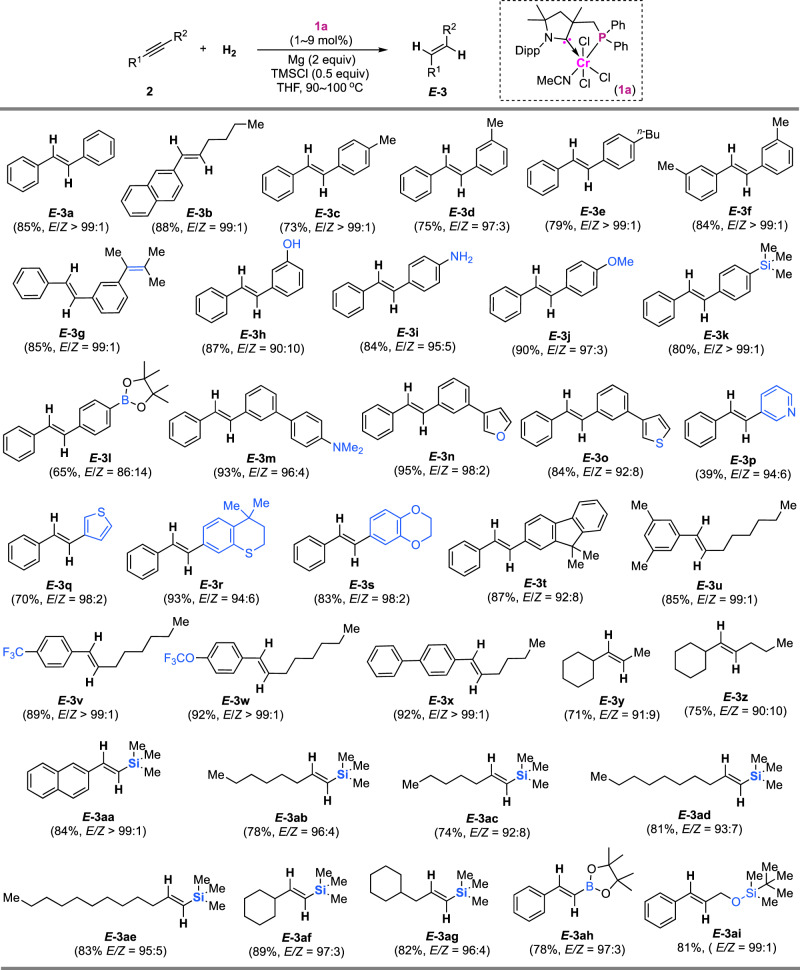
CAAC-P ligand-enabled *E*-selectivity of hydrogenation of alkynes with chromium catalysis. Substrate scope for *E*-selective hydrogenation of alkynes.

Using complex **1a**, diarylacetylenes containing alkyl substituents undergo the *trans*-hydrogenation smoothly, affording access to *E*-olefins (***E*-3c**–**f**) in good yields and with high selectivity (Fig. 3). As expected, the alkenyl substituent in the alkyne substrate is retained in the hydrogenation and then provides a strategy to construct alkenyl-substituted *E*-stilbene (***E*-3g**). The incorporation of OH and NH_2_ groups into precursors does not inhibit the hydrogenation of alkynes (***E*-3h** and **i**). Common functionalities of methoxy, trimethylsilyl, pinacol boronate ester and amino are compatible with the hydrogenation system (***E*-3j**–**m**). Hydrogenation with furanyl-, thiophenyl-, and pyridyl-containing alkynes enables stereoselectively accessing *E*-alkenyl-substituted heteroarene products (***E*-3n**–**q**). Through hydrogenation, functionalized *E*-olefins of styrylthiochromane, styryl-dihydrobenzo[*b*][1,4]dioxine, and styryl-9*H*-fluorene are stereoselectively accessible (***E*-3r**–**t**). The strategy was extended to hydrogenate alkyl aryl alkynes, affording alkylarylated olefins with 99:1 *E*/*Z* selectivity (***E*-3u**–**x**). The CAAC-P–Cr-catalyzed *trans*-hydrogenation of dialkylalkynes occurs smoothly, providing access to *E*-dialkylalkene compounds ***E*-3y** and **3z** in good selectivity. Alkynes that a silicon or boron functional group is directly connected with the reactive triple bonds and have rarely been hydrogenated in the stereoselective formation of olefins. Interestingly, silyls that are directly connected to the alkynes are tolerated in the system, offering a strategy to access a range of *E*-silylated alkenes ***E*-3aa**–**ag** in up to >99:1 *E*/*Z* selectivity. Boronate ester-substituted alkyne undergoes hydrogenation in a *trans*-selective manner, resulting in the formation of *E*-borylated alkene ***E*-3ah**. The incorporation of a silyloxy into the alkyl substituent does not affect the stereoselectivity to afford *E*-alkene (***E*-3ai**).

### Scope of CAAC-imino ligand-enabled *cis*-hydrogenation

**Fig. 4:**
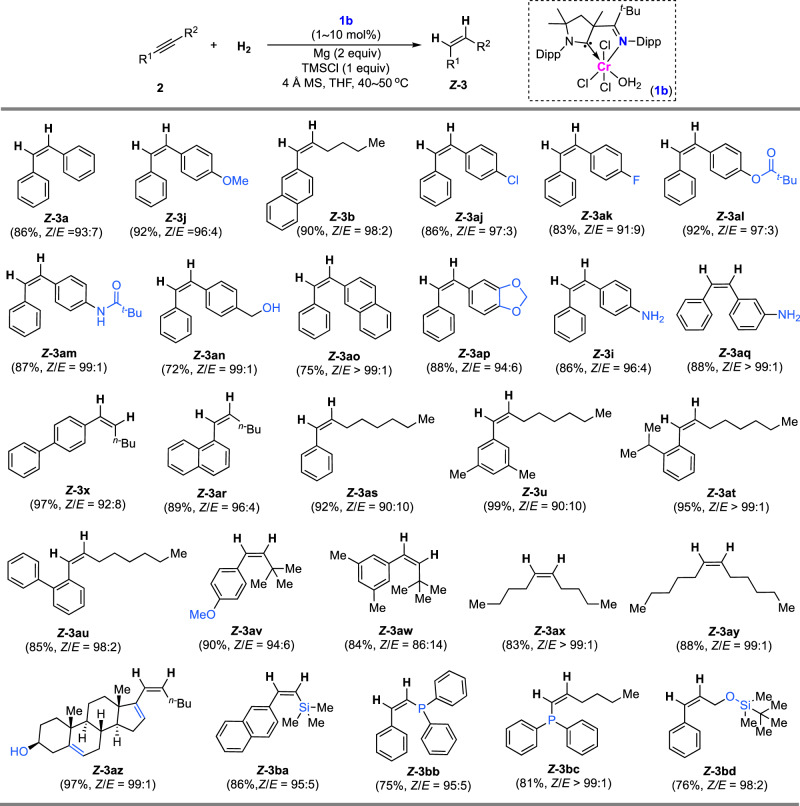
*Z*-selective hydrogenation of alkynes enabled by CAAC-imino ligand. Substrate scope for *Z*-selective hydrogenation of alkynes.

By control of the stereochemistry with complex **1b**, *Z*-stilbenes that contain a variety of functionalities such as chloride, fluoride, ester, amide, hydroxyl, and naphthyl (***Z*-3aj**–**ao**) can be formed in good yields and with high selectivity (Fig. 4). The hydrogenation strategy was successfully used in the construction of *Z*-styrylanilines ***Z*-3i** and ***Z*-3aq** containing medicinally interesting amino functionality. Aryl- and alkyl-substituted alkynes undergo hydrogenation smoothly without influencing the *Z*-selectivity (***Z*-3x** and -**3ar**). Compared with hydrogenating alkynes without *ortho*-substituents in the aryls (***Z*-3as** and -**3u**), excellent *Z*-selectivity was obtained when *ortho*-isopropyl- or phenyl-substituted aryl alkynes were used (***Z*-3at** and -**3au**). This indicates that sterically congested alkynes may facilitate the delivery of high *Z*-selectivity in the hydrogenation. Alkynes containing two aliphatic substituents of butyl and pentyl readily undergo hydrogenation, leading to the formation of *Z*-olefins with 99:1 *Z*/*E* selectivity (***Z*-3ax** and -**3ay**). Hydrogenation of the alkyne with a biologically active androsta-5,16-dien-3-ol scaffold enables the synthesis of a *Z*-olefin derivative in excellent yield and excellent selectivity (***Z*-3az**). Using the CAAC-imino–Cr complex, alkynes that a silyl or phosphino substituent is directly connected with the triple bonds are hydrogenated effectively, giving high *Z*-selectivity in the formation of compounds of ***Z*-3ba**–**bc**.

### Gram-scale hydrogenation and application in accessing *E*- and *Z*-olefin-bearing derivatives

**Fig. 5:**
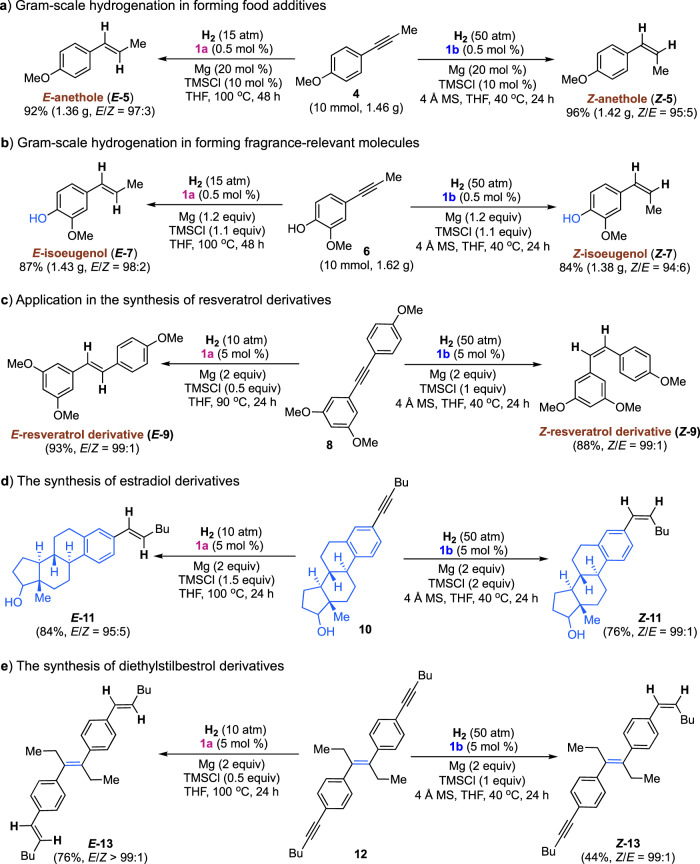
Gram-scale hydrogenation and application in the efficient synthesis of both *E*- and *Z*-selective olefin derivatives. **a** Gram-scale hydrogenation in forming food additives. **b** Gram-scale hydrogenation in forming fragrance-relevant molecules. **c** Application in the synthesis of resveratrol derivatives. **d** The synthesis of estradiol derivatives. **e** The synthesis of diethylstilbestrol derivatives.

Applying the *E*/*Z* selectivity controllable hydrogenation strategy, we then hydrogenated alkyne precursors related to the industrially produced *E*- and *Z*-stereoselective anetholes (**5**) and isoeugenol (**7**) on a one-gram scale with 0.5 mol% of CAAC–Cr precatalysts (Fig. 5). Polyphenolic phytoalexins of *E*- and *Z*-resveratrol derivatives (**9**) were readily accessible by the hydrogenation of alkynes. Hydrogenation afforded pharmaceutically interesting estradiol-derived *E*- and *Z*-olefin derivatives in high efficiency (**11**). In the presence of CAAC-P–Cr complex **1a**, the two alkynyl in diethylstilbestrol-containing alkyne (**12**) are hydrogenated in the formation of the olefin derivative, with extraordinary *E*/*Z* selectivity (>99:1). By contrast, CAAC-imino-ligated Cr complex **1b** selectively hydrogenates one of the triple bonds, while another alkynyl functionality remains intact, thus affording the alkynyl- and alkenyl-containing derivative ***Z*-13** with 99:1 *Z*/*E* selectivity.

## Discussion

### Kinetic studies

**Fig. 6:**
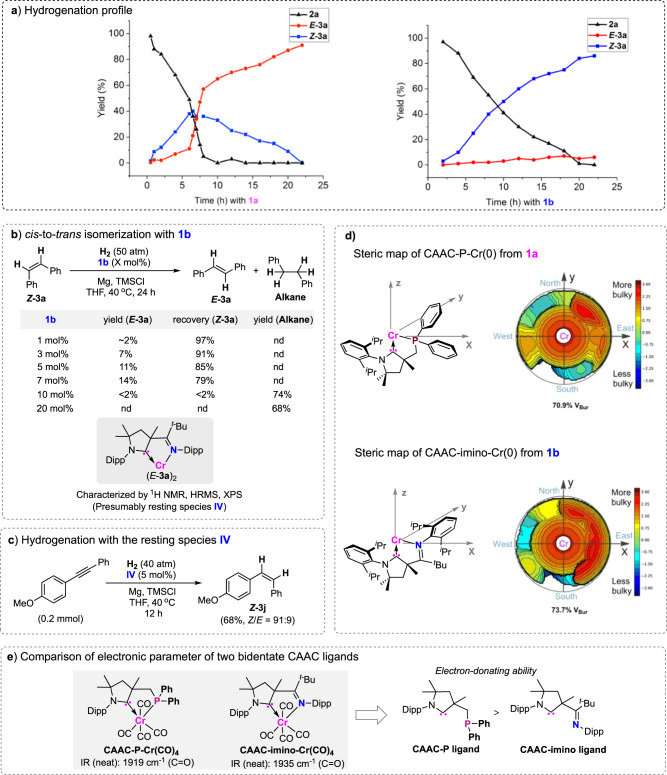
Mechanistic studies of controlling the *E/Z* selectivity by CAAC ligands. **a** Reaction profile for the *E/Z* selective hydrogenation with complexes **1a** and **1b**. **b** Studying the *cis*-to-*trans* isomerization with complex **1b**. **c** Hydrogenation catalyzed by the resting species **IV**. **d** Steric maps of CAAC–Cr(0). **e** Comparison of electronic parameters of two bidentate CAAC ligands.

The reaction time course with CAAC-P-ligated Cr complex **1a** suggested that *Z*-stilbene was mainly formed at the early stage of hydrogenation, whereas it was sluggishly consumed after 6 h, probably by a *cis*-to-*trans* geometrical isomerization (Fig. 6a). This is in line with an increased rate for the formation of *E*-stilbene, giving yields from 11% to 57%, with complete consumption of alkyne within 2 h. In contrast, the hydrogenation using CAAC-imino-ligated complex **1b** mainly gives *Z*-olefins, which almost do not convert into the related *E*-stereomer during the process.

### Mechanistic studies

To gain insight into achieving high *Z*-selectivity with **1b**, the possibility of the stereoisomerization of ***Z*-3a** was examined. The almost twofold formation of *E*-stilbene relative to CAAC-imino–Cr was observed when <8 mol% of **1b** was used (Fig. 6b). Analysis of the resulting complex by ^1^H nuclear magnetic resonance spectroscopy and high-resolution mass spectroscopy indicates that the formation of a resting species **IV** by chelation of *E*-stereomer with CAAC-imino–Cr in a 2:1 ratio may be considered, in which Cr adopts the zero-valent state, based on analysis by X-ray photoelectron spectroscopy (see Supplementary Information). The use of a large amount of CAAC-imino–Cr complex in reactions (e.g., 10 mol%) mainly gives an over-hydrogenated alkane, probably because of the high concentration of catalyst resulting in direct olefin hydrogenation. Although the resting species is inefficient in the catalytic stereoisomerization of *Z*-olefins, it could promote the catalytic hydrogenation of alkyne, which is in agreement with its good performance in the achievement of high *Z*-selectivity (Fig. 6c).

The effect of steric circumstances around the Cr centers in complexes **1a** and **1b** was investigated by calculation of the percentage buried volume (%*V*_bur_) and topographic steric map of their optimized structure of CAAC–Cr(0) (Fig. 6d). Compared with CAAC-phosphino-bearing Cr(0) derived from **1a**, the steric map for CAAC-imino-ligated Cr(0) shows a higher percentage buried volume by occupying a different portion of the space around the Cr with the ligand (73.7% vs. 70.9%), causing greater steric congestion in the reactive pocket. We next studied the electronic parameter of these bidentate CAAC ligands, by preparing their coordinated Cr carbonyl complexes by literature methods and measuring the IR spectra (Fig. 6e)^63^. Comparing the values of the *ν*(C = O) vibration indicate that a strong electron-donating character of CAAC-phosphino ligand relative to CAAC-imino in the Cr complexes is considered^64,65^. The alkyne hydrogenation using CAAC–Cr complexes may initially lead to the formation of *Z*-olefins, which can be stereo-isomerized with sterically less bulky and more electron-rich CAAC-phosphino–Cr **1a** in giving *E*-stereomers. Theoretical studies by density functional theory (DFT) calculations suggest that the stereoisomerization of ***Z*-3a** with reactive CAAC-phosphino–Cr–H species occurs by a pathway involving hydrometalation and *β*-hydride elimination, by overcoming low reaction energies in forming ***E*-3a** (Supplementary Data 1 contains the cartesian coordinates of the structures). In contrast, the use of CAAC-imino–Cr–H in the stereoisomerization is unfavorable in energy because of relatively higher activation barriers. The structure of related transition state (**TS-9N**) for the difficult *β*-hydride elimination step shows a congested circumstance in the reactive pocket, wherein the steric repulsion was mainly caused by the proximity of two diisopropyl phenyl groups of CAAC-imino and phenyl substituents of olefins. Analysis of the charge population of **TS-9N** suggested that the charge distributions on the chromated C_olefin_ moiety have limited change compared with that of the related transition state (**TS-10P**) with CAAC-phosphino (see Supplementary Information), unlike their relatively large difference in Cr–C_olefin_ bond distance, indicating a minor contribution of the electronic features of the ligand on the stereoisomerization. The major role of the steric effect of these CAAC ligands on such unique selectivity may be considered. We hypothesize that the crowded circumstances in the reactive pocket with **1b** are unfavorable to the stereoisomerization of *cis*-olefins, but presumably, allow hydrogenation of alkynes because of the relatively strong coordination between alkynes and metals. This may account for the inefficiency of the CAAC-imino–Cr complex in the stereoisomerization of olefins, and therefore high *Z*-selectivity is attained in the hydrogenation.

In conclusion, we have devised strategies that allow for alkyne hydrogenation to proceed in an *E*- and *Z*-selectivity-controllable manner with two distinct bidentate CAAC ligands by Cr catalysis. The feasibility of using two CAAC ligands by modifying anchors to control both the *E*- and *Z*-selectivity of hydrogenation enhances the potential effect of this strategy. With the environmental and cost benefits that catalytic hydrogenation brings to chemical synthesis, these ligand-controllable and stereo-complementary approaches enable the large-scale, sustainable, and selective H_2_-hydrogenation of simple alkynes to produce on-demand valuable sets of *E*- and *Z*-olefins in high efficiency. This is particularly relevant to cases where there are electronic and steric differences between the two CAAC ligand anchors^66–69^. The methods described above are expected to lead to the design of robust metal catalysts for developing ligand-controlled selective reactions in the efficient construction of industrially important chemicals.

## Methods

### General procedure of CAAC-phosphino–Cr-catalyzed *trans*-selective hydrogenation of alkynes for the synthesis of *E*-olefins

In a Schlenk tube were placed alkyne **2** (0.2 mmol), **1a** (1–9 mol%), Mg (10 mg), TMSCl (13 μL), and THF (2 mL) under an atmosphere of nitrogen. The tube was quickly moved to a high-pressure autoclave and stirred under an atmosphere of H_2_ (6–15 atm) at 90–100 °C for 24 h. After quenching with HCl_aq_ (2 mL, 1 M), the crude product was extracted with ethyl acetate (3 × 4 mL). The combined organic phases were dried over anhydrous Na_2_SO_4_ and concentrated under a vacuum. The stereoselectivity of *E*-olefin relative to *Z*-stereomer was determined by GC analysis or ^1^H NMR prior to purification. The crude product was purified by silica gel chromatography to afford the related olefin compound.

### General procedure of CAAC-Imino–Cr-catalyzed *Cis*-selective hydrogenation of alkynes for the synthesis of *Z*-olefins

In a Schlenk tube were placed **1b** (1–10 mol%), alkyne **2** (0.2 mmol), Mg (10 mg), 4 Å MS (25 mg), TMSCl (25 μL), and THF (2 mL) under an atmosphere of nitrogen. The tube was quickly moved to a high–pressure autoclave and stirred under an atmosphere of H_2_ (40–50 atm) at 40–50 ^o^C for 24 h. After quenching with HCl_aq_ (2 mL, 1 M), the crude product was extracted with ethyl acetate (3 × 4 mL). The combined organic phases were dried over anhydrous Na_2_SO_4_ and concentrated under a vacuum. The stereoselectivity of the *Z*-olefin relative to *E*-stereomer was determined by GC analysis or ^1^H NMR prior to purification. The crude product was purified by silica gel chromatography to afford the desired olefin product.

### Spectroscopic methods

^1^H and ^13^C NMR spectra were recorded on a Bruker DRX-400 (operating at 400 MHz for ^1^H and 100 MHz for ^13^C). GC–MS spectra were recorded on an Agilent Technologies 7890B GC-system with an Agilent 5977B MSD and an HP-5MS column (0.25 mm × 30 m × 0.25 μm). High-resolution mass spectra (HRMS) were recorded on the Exactive Mass Spectrometer (Thermo Scientific, USA) equipped with ESI ionization source. X-ray photoelectron spectroscopy (XPS) data were collected with a Thermo Fisher ESCALAB Xi^+^ spectrometer equipped with monochromatic Al Kα radiation. IR spectra were recorded on a PerkinElmer spectrum two spectrometers using the transmittance method. Electron paramagnetic resonance (EPR) spectroscopic measurements were performed on the Bruker A300 spectrometer. Elemental analysis (EA) spectroscopic measurements were recorded on the Elemantar Vario EL cube.

### Single-crystal X-ray structure determinations

The crystal data of **1a** were collected on a Bruker SMART CCD diffractometer with MoK*α* radiation (*λ* = 0.71073 Å). The structures were solved by direct methods and refined on *F*^2^ using SHELXTL. All nonhydrogen atoms were refined anisotropically.

## Supplementary information

Supplementary Information

Description of Additional Supplementary Files

Supplementary Data 1

## Data Availability

The X-ray crystallographic coordinates for structures that support the findings of this study have been deposited at the Cambridge Crystallographic Data Centre (CCDC) with the accession code CCDC 2015820 (**1a**). The authors declare that all other data supporting the findings of this study are available within the article and Supplementary Information files, and also are available from the corresponding author upon request.
