# Supplementary material for: Chromium-catalyzed stereodivergent E- and Z-selective alkyne hydrogenation controlled by cyclic (alkyl)(amino)carbene ligands
Source: Nat Commun. 2023 Feb 22;14:990. doi: 10.1038/s41467-023-36677-9 (PMC9947122; doi:10.1038/s41467-023-36677-9)
Supplement: Supplementary file 1 — Supplementary Information [file 41467_2023_36677_MOESM1_ESM.pdf]

Supplementary Information

Chromium-catalyzed stereodivergent *E*- and *Z*-selective alkyne hydrogenation controlled by cyclic (alkyl)(amino)carbene ligands

Liang Ling,[1] Chenyang Hu,[2] Linhong Long[3], Xue Zhang,[1] Lixing Zhao,[1] Liu Leo Liu,[2] Hui Chen[3]*, Meiming Luo[1]* & Xiaoming Zeng[1]*

[1]Key Laboratory of Green Chemistry & Technology, Ministry of Education, College of Chemistry, Sichuan University, Chengdu 610064, China;
[2]Shenzhen Grubbs Institute and Department of Chemistry, Southern University of Science and Technology; Shenzhen 518055, China
[3]Beijing National Laboratory for Molecular Sciences (BNLMS), Key Laboratory of Photochemistry, CAS Research/Education Center for Excellence in Molecular Sciences, Institute of Chemistry, Chinese Academy of Sciences, Beijing 100190, China

Table of Contents

1. Supplementary Methods 3

2. Supplementary Synthesis of Chromium Complex 4

3. Supplementary Synthesis of Substrates 11

4. Supplementary Optimizing Reaction Parameters 13

5. Supplementary General Procedure of CAAC-Phosphino-Cr-Catalyzed *Trans*-Selective Hydrogenation of Alkynes for the Synthesis of *E*-Olefins 17

6. Supplementary General Procedure of CAAC-Imino-Cr-Catalyzed *Cis*-Selective Hydrogenation of Alkynes for the Synthesis of *Z*-Olefins 36

7. Supplementary Reaction Profile for Hydrogenation of 2a 52

8. Supplementary Mechanistic Experiments. 56

9. Supplementary Synthetic Applications in Accessing Functionalized *E*- and *Z*-olefin Derivatives 69

10. Supplementary the Single Crystal Data of the Complex of 1a (CCDC: 2015820) 75

11. Supplementary DFT Computational Study 89

12. Supplementary ^{1}H NMR, ^{13}C NMR and ^{19}F NMR Spectra 93

13. Supplementary References 169

1. Supplementary Methods

General information

All reactions dealing with air- or moisture-sensitive compounds were carried out in a flame-dried and sealed Schlenk tube under atmosphere of nitrogen. Analytical thin-layer chromatography was performed on glass plates coated with silica gel (0.25 mm, 230–400 mesh) containing a fluorescent indicator (Merck), or phosphomolybdic acid hydrate or $KMnO_4$ staining solutions and followed by heating. Flash silica gel column chromatography was performed on silica gel 60 N (spherical and neutral, 140–325 mesh). NMR spectra were measured on a Bruker AVANCE III HD spectrometer and reported in parts per million (ppm). ^1H NMR spectra were recorded at 400 MHz in $CDCl_3$ were referenced internally to tetramethylsilane as standard, ^{13}C NMR spectra were recorded at 100 MHz and referenced to the solvent resonance. ^{19}F NMR spectra are not calibrated by an internal reference. Chemical shifts are reported in ppm, relative to solvent residual peaks) and coupling constants *J* in Hertz (Hz). Signals are described as s = singlet, d = doublet, dd = doublet of doublets, t = triplet, q = quartet and m = multiplet. Melting points were determined with a Hanon Instruments MP300. Analytical gas chromatography (GC) was carried out on an Agilent Technologies 7890B GC–system, equipped with FID detector and a J&W GC column (0.32 mm × 30 m × 0.25 μm). The methods were used by starting with the injection temperature T_0; after holding the related temperature for 3 min, the column was heated to temperature T_1 (ramp). (GC Method: T_0 = 50 °C, T1 = 280 °C, ramp = 15 °C/min). GC–MS spectra were recorded on an Agilent Technologies 7890B GC–system with an Agilent 5977B MSD and a HP–5MS column (0.25 mm × 30 m × 0.25 μm). The major signals are quoted in *m*/*z* with the relative intensity in parentheses. The methods were used by starting with the injection temperature T_0; after holding this temperature for 3 min, the column was heated to the temperature T_1 (ramp). (GC–MS Method: T_0 = 50 °C, T_1 = 280 °C, ramp = 15 °C/min). High resolution mass spectra (HRMS) were recorded on the Exactive Mass Spectrometer

(Thermo Scientific, USA) equipped with ESI ionization source. X-ray photoelectron spectroscopy (XPS) data were collected with a Thermo Fisher ESCALAB Xi^+ spectrometer equipped with monochromatic Al Kα radiation. IR spectra were recorded on a PerkinElmer Spectrum Two spectrometer using transmittance method. Electron paramagnetic resonance (EPR) spectroscopic measurements were performed on the Bruker A300 spectrometer. Elemental analysis (EA) spectroscopic measurements were recorded on the Elemantar Vario EL cube.

Material. Unless otherwise noted, materials were purchased from Tokyo Chemical Industry Co., Aldrich Inc., Alfa Aesar, Adamas-beta®, Energy Chemical and other commercial suppliers and used as received. Solvents were dried over CaH_2 (for DCM, $CDCl_3$ and MeCN) and sodium (for THF, Toluene and hexane) by refluxing for overnight and freshly distilled prior to use. $CrCl_3$ (99.99% purity) were purchased from Aldrich Inc. and used as received. $Cr(acac)_3$ (97% purity), $CrCl_2$ (99% purity) were purchased from Alfa Aesar and used as received. The alkyne precursors were purchased or prepared according to the known procedures. Alkynes **2a** (98% purity), **2ax** (98% purity), **2ay** (98% purity) were purchased for Energy Chemical Inc. The preparation of CAAC-Cr complexes was performed under atmosphere of nitrogen inside a glovebox. Molecular sieve was activated at high temperature under nitrogen atmosphere before utilization. Glassware and stirrer bars were dried in oven for 24 hours prior to use. Liquid reagents, solutions or solvents were added via syringe or cannula through rubber septa. The selectivity of *E*-olefins relative to Z-stereomers was determined by GC analysis or ^{1}H NMR prior to purification.

2. Supplementary Synthesis of Chromium Complex

The related iminium salts **14–16** and **1a–1d** were prepared according to the reported methods in literatures.[1–4]

General procedure for EPR experiments.

The resulting THF solutions bellow (**1a–1d**) were partly transferred into a capillary column (20 cm). The sample was then put into a quartz tube and measured at 77k (Frequency = 9.852947 GHz, Power = 19.00 mW, Field Center = 2750.000 G, Width +/- = 4500.000 G, Sweep Time = 122.88 sec, Modulation Frequency = 100.00 kHz, Amplitude 1.00 G, Time Constant 10.24 msec, Phase 0.00 deg. The EPR spectrum were shown in Supplementary Figure S1-4.

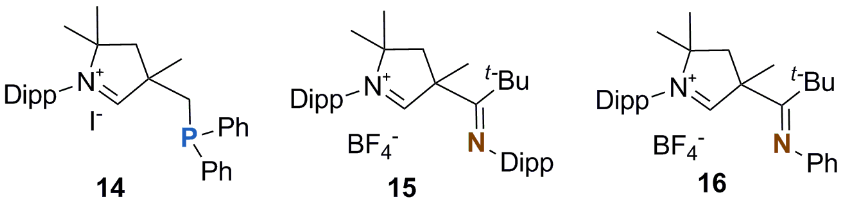

Dipp = 2,6-diisopropylphenyl

2.1 Synthesis of Iminium Salt 14

A dried Schlenk tube was charged with cyclic iminium salt (2.70 g, 5 mmol), diphenylphosphine (1.02 g, 5.5 mmol), *N*,*N*-diisopropylethylamine (2.58 g, 20 mmol) in THF (10 mL). The mixture was stirred at 100 °C for 48 h. After removal of volatiles under vacuum, K_3PO_4 (1.27 g, 6 mmol) and MeCN (20 mL) were added and the mixture was stirred at room temperature for 12 h. After removal of solvents under vacuum, the crude product was extracted with CH_2Cl_2 (30 mL). After removal of the volatiles under vacuum, the resulting yellow solid was washed with THF (60 mL) to afford a white solid powder (1.79g, 60%). ^{1}H NMR (400 MHz, $CDCl_3$): δ 10.77 (s, 1H), 7.62–7.55 (m, 2H), 7.48–7.37 (m, 6H), 7.30–7.15 (m, 5H), 3.13–2.97 (m, 3H), 2.64–2.51 (m, 1H), 2.26 (d, J = 14.0 Hz, 1H), 2.13 (d, J = 14.0 Hz, 1H), 1.87 (s, 3H), 1.44 (s, 3H), 1.31–1.21 (m, 12H), 1.16 (d, J = 6.6 Hz, 3H); ^{13}C NMR (100 MHz, $CDCl_3$): δ 191.5 (d, J = 6.3 Hz), 145.1, 144.5, 137.2 (d, J = 7.8 Hz), 135.6 (d, J = 10.0 Hz), 134.0, 133.8, 132.6, 132.4, 131.9, 130.2, 129.3, 129.3, 128.7, 128.6, 125.4, 125.3, 82.7, 52.9 (d, J = 19.0 Hz), 46.5 (d, J = 6.5 Hz), 37.5 (d, J = 15.4 Hz), 30.0 (d, J = 7.3 Hz), 29.9, 29.7, 28.7, 28.6, 27.7, 27.6, 22.2, 21.8; ^{31}P NMR (162 MHz, $CDCl_3$): δ –19.0.

2.2 Synthesis of CAAC Iminium Salt 15

In a dried Schlenk tube, *N*-diisopropylphenylpivalimidoyl chloride (2.79 g, 10 mmol), 1-(2,6-diisopropylphenyl)-2,2,4-trimethyl-2,3-dihydro-1*H*-pyrrole (2.71 g, 10 mmol), $NaBF_4$ (1.21 g, 11 mmol) and MeCN (10 mL) were added under nitrogen atmosphere. After the mixture was stirred at room temperature for 12 h, K_3PO_4 (1.06 g, 5 mmol) was added and the mixture was stirred for 12 h. The CH_3CN solution was filtered and the solvent was removed under vacuum. The crude product was extracted with CH_2Cl_2 (30 mL). After removal of CH_2Cl_2, the yellow solid was washed with Et_2O (100 mL) to afford a white solid powder (4.87g, 81%). ^{1}H NMR (400 MHz, $CDCl_3$): δ = 8.45 (s, 1H), 7.52 (t, *J* = 8.0 Hz, 1H), 7.33 (d, *J* = 7.2 Hz, 1H), 7.28–7.26 (m, 1H), 7.03 (s, 3H), 3.12 (d, *J* = 13.6 Hz, 1H), 2.86–2.74 (m, 5H), 2.22 (s, 3H), 1.68 (s, 3H), 1.56 (s, 3H), 1.35 (d, *J* = 6.8 Hz, 3H), 1.31–1.26 (m, 6H), 1.23–1.18 (m, 18H), 0.97 (d, *J* = 6.8 Hz, 3H), 0.66 (d, *J* = 6.8 Hz, 3H); ^{13}C NMR (100 MHz, $CDCl_3$): δ = 183.5, 182.5, 145.5, 145.4, 143.4, 134.8, 134.5, 131.9, 128.6, 125.7, 125.3, 124.2, 123.2, 122.8, 83.0, 64.4, 49.8, 43.5, 29.7, 29.4, 29.0, 28.7, 28.5, 27.9, 26.5, 26.2, 24.7, 24.6, 23.8, 22.9, 22.4, 22.2, 21.9.

2.3 Synthesis of CAAC Iminium Salt 16

In a dried Schlenk tube, *N*-phenylpivalimidoyl chloride (1.95 g, 10 mmol), 1-(2,6-diisopropylphenyl)-2,2,4-trimethyl-2,3-dihydro-1*H*-pyrrole (2.71 g, 10 mmol), $NaBF_4$ (1.21 g, 11 mmol) and MeCN (10 mL) were added under nitrogen atmosphere. After the mixture was stirred at room temperature for 12 h, K_3PO_4 (1.06 g, 5 mmol) was added and the mixture was stirred for another 12 h. The CH_3CN solution was filtered and the solvent was removed under vacuum. The crude product was extracted with CH_2Cl_2 (30 mL). After removal of CH_2Cl_2, the yellow solid was washed with Et_2O (100 mL) to afford a white solid powder (3.53g, 68%). ^{1}H NMR (400 MHz, $CDCl_3$): δ 8.84 (s, 1H), 7.47 (t, *J* = 7.8 Hz, 1H), 7.32–7.21 (m, 4H), 7.03 (t, *J* = 7.4 Hz, 1H), 6.86 (s, 2H), 3.09 (d, *J* = 14.2 Hz, 1H), 3.00–2.89 (m, 1H), 2.75–2.58 (m, 2H),

2.06 (s, 3H), 1.65 (s, 3H), 1.55 (s, 3H), 1.37 (d, J = 6.8 Hz, 3H), 1.28 (s, 9H), 1.17–1.11 (m, 6H), 0.48 (d, J = 6.4 Hz, 3H); ^{13}C NMR (100 MHz, $CDCl_3$): δ 184.0, 183.2, 148.1, 146.0, 145.3, 131.5, 129.0, 128.7, 125.5, 124.9, 123.5, 117.8, 82.1, 62.7, 46.9, 42.6, 30.4, 29.9, 29.2, 27.7, 27.0, 26.1, 26.0, 23.3, 22.1, 21.8.

2.4 Synthesis of CAAC-Cr Complex 1a

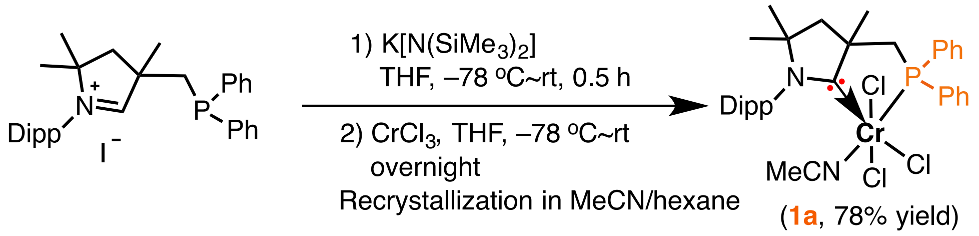

In a dried Schlenk tube, iminium salt **14** (597 mg, 1 mmol) was added under nitrogen atmosphere. After addition of THF (10 mL) by syringe at –78 °C, a solution of KHMDS (1.1 mL, 1.1 mmol, 1 M/L in THF) was added dropwise by syringe and stirred at room temperature for 0.5 h. The solution was transferred to a dry Schlenk tube containing $CrCl_3$ (158 mg, 1 mmol) under atmosphere of nitrogen at –78 °C and stirred at room temperature for overnight. After removal of volatiles under vacuum, the solid was then extracted with DCM (20 mL). After removal of DCM, the solid was washed with hexane (30 mL) to afford a dark green powder. Single crystals of **1a** suitable for X-ray crystallography was obtained by recrystallization from hexane to acetonitrile at –30 °C (520 mg, 78%). IR (neat) ν 3370, 2964, 1616, 1412, 1251, 1033, 856, 606, 558 cm^{-1}. HRMS (ESI, m/z): calcd for $C_{32}H_{40}Cl_2CrN_1P$ [M–Cl–MeCN]$^+$: 591.1681, found: 591.1675. Elemental analysis (calcd., found for $C_{34}H_{43}Cl_3CrN_2P$): C (61.04, 61.03), H (6.48, 6.41), N (4.19, 4.77).

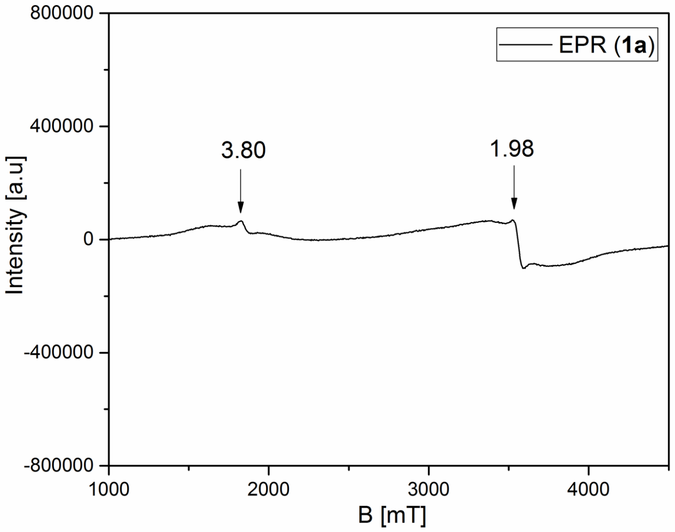

Supplementary Figure S1. EPR spectrum of 1a in THF (77 K)

2.5 Synthesis of CAAC-Cr Complex 1b[2]

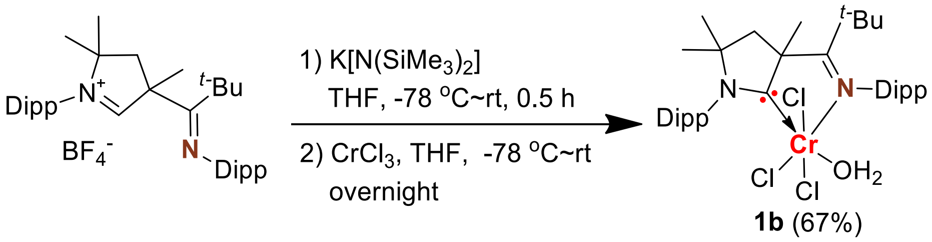

In a dried Schlenk tube, CAAC iminium salt **15** (602 mg, 1 mmol) was added under nitrogen atmosphere. After addition of THF (10 mL) by syringe at –78 ºC, a solution of KHMDS (1.1 mL, 1.1 mmol, 1 M/L in THF) was added dropwise by syringe and stirred at room temperature for 0.5 h. The solution was transferred to a dry Schlenk tube containing $CrCl_3$ (158 mg, 1 mmol) at –78 °C under atmosphere of nitrogen and stirred overnight at room temperature. After removal of volatiles under vacuum, the solid was extracted with DCM (20 mL). After removal of DCM, the solid was washed with hexane (30 mL) to afford a celadon powder (462 mg, 67%). IR (neat) ν 3740, 3418, 3152, 2969, 2876, 1636, 1466, 1406, 1034, 805, 526 cm^{-1}. Elemental analysis calcd (calcd., found for $C_{36}H_{56}Cl_3CrN_2O$): C (62.56, 62.25), H (8.17, 8.20), N (4.05, 4.03).

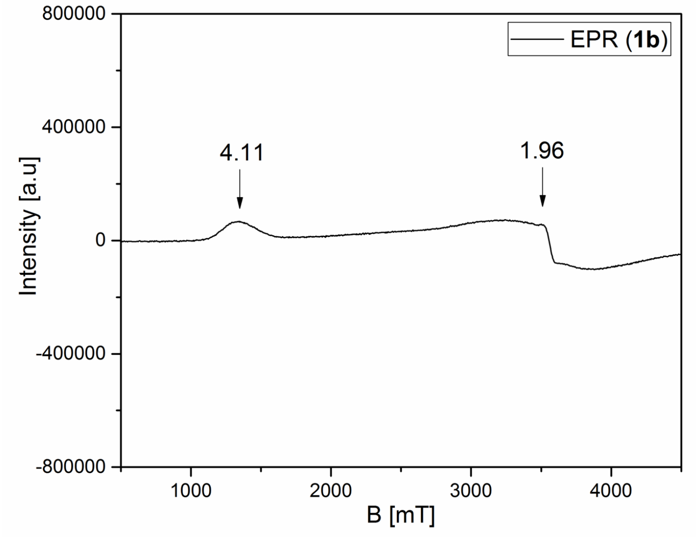

Supplementary Figure S2. EPR spectrum of 1b in THF (77 K)

2.6 Synthesis of CAAC-Cr Complex 1c

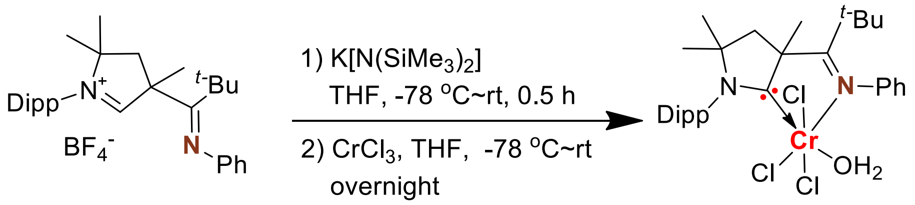

In a dried Schlenk tube, CAAC iminium salts **16** (518 mg, 1 mmol) was added under nitrogen atmosphere. After addition of THF (10 mL) by syringe at –78 ºC, a solution of KHMDS (1.1 mL, 1.1 mmol, 1 M/L in THF) was added dropwise by syringe and stirred at room temperature for 0.5 h. The solution was transferred to a dry Schlenk tube containing $CrCl_3$ (158 mg, 1 mmol) at –78 °C under atmosphere of nitrogen and stirred overnight at room temperature. After removal of volatiles under vacuum, the solid was extracted with DCM (20 mL). After removal of DCM, the solid was washed with hexane (30 mL) to afford a green powder (393 mg, 65%). IR (neat) ν 3737, 3627, 3072, 2965, 2873, 1606, 1469, 1304, 805, 529 cm^{-1}. HRMS (ESI, m/z) calcd for $C_{30}H_{42}CrN_2Na$ $[M–3Cl–H_2O+Na]^+$: 505.2645, found: 505.2659. Elemental analysis (calcd., found for $C_{30}H_{44}Cl_3CrN_2O$): C (59.36, 59.05), H (7.31,7.51), N (4.61, 4.28).

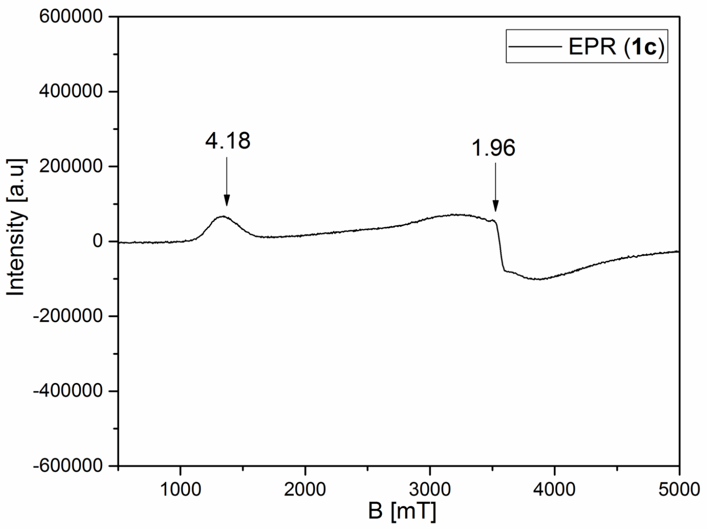

Supplementary Figure S3. EPR spectrum for 1c in THF (77 K)

2.4 Synthesis of Cr Complex 1d[3]

In a dried Schlenk tube, 4,4'-Di-tert-butyl-2,2'-bipyridine (268 mg, 1 mmol) and $CrCl_3{\cdot}(THF)_3$ (373 mg，1mmol) were added under nitrogen atmosphere. After addition of MeCN (3 mL) and sonicated for 1 min. After removal of volatiles under vacuum, the solid was then extracted with hot THF (50 mL). The resultant cloudy solution was filtered through Celite and concentrated down to half its volume. Single crystals of **1d** suitable for X-ray crystallography was obtained by recrystallization from pentane to THF. The supernatant was discarded, and the crystals were washed with pentane and dried under reduced pressure to give the Cr complex **1d** (230 mg, 52%). IR (neat) ν 3757, 3373, 3054, 2973, 2928, 1621, 1468, 1383, 743, 695, 519 cm^{-1}. Elemental analysis (calcd., found for $C_{18}H_{26}Cl_3CrN_2O$): C (48.61, 49.26), H (5.89, 5.76), N (6.30, 6.27).

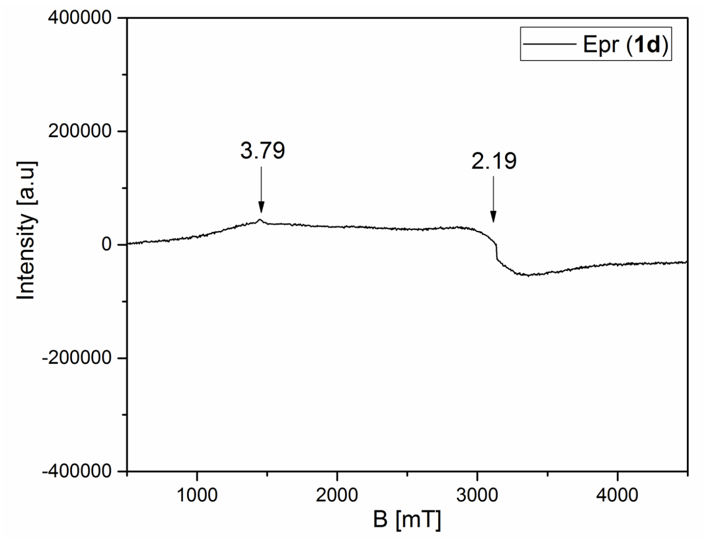

Supplementary Figure S4. EPR spectrum for 1d in THF (77 K)

3. Supplementary Synthesis of Substrates

Method A[5]

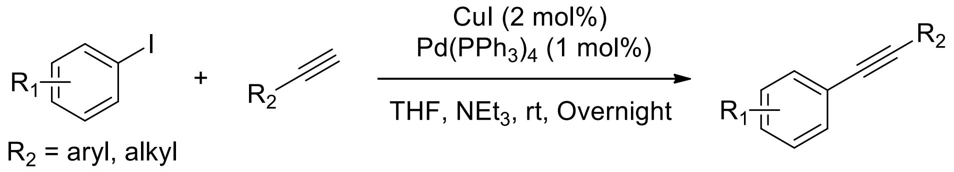

2b-2x, **2aj-2aw** **2az** and **8** were synthesized according to Method A. A mixture of iodobenzene (5 mmol), terminal alkyne (6 mmol), CuI (20 mg, 2 mol %) and $Pd(PPh_3)_4$ (58 mg, 1 mol %) was added to an over dried tube under atmosphere of nitrogen. THF (5 mL) and NEt_3 (5 mL) was then added by syringe. The mixture was stirred at room temperature for 12 h. The reaction mixture was diluted with ethyl acetate, and washed with saturated solution of sodium chloride. The organic layer was separated, dried and concentrated under vacuum. The crude product was purified by column chromatography on silica gel to afford the related alkynes.

Method B[6]

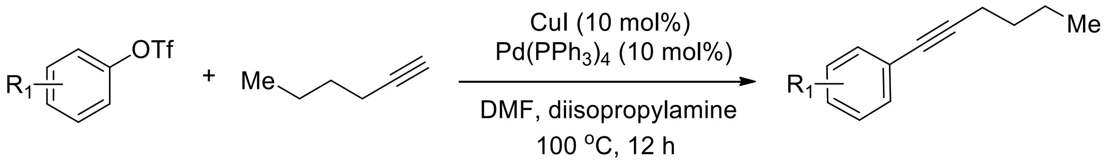

10 and **12** were synthesized according to Method B. To an over dried 25 mL two-necked flask, aryl triflyl (2 mmol), 1-hexyne (197 mg), CuI (38 mg, 10 mol %), $Pd(PPh_3)_4$ (230 mg, 10 mol %), diisopropylamine (607 mg, 3 equiv) and DMF (24 mL) were added under argon atmosphere. The mixture was stirred at 100 °C for 12 h. The reaction mixture was diluted with ethyl acetate, and washed with saturated solution of sodium chloride. The organic layer was separated, dried and concentrated under vacuum. The crude product was purified by column chromatography on silica gel to give the desired alkynes.

Method C[7]

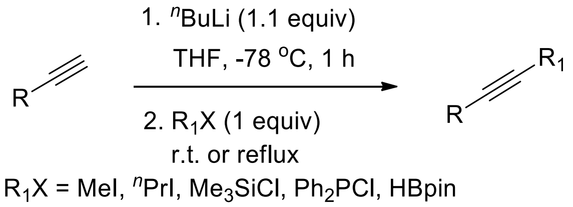

To an over dried 100 mL two-necked flask, alkynes (10 mmol) and THF (30 mL) were added under argon atmosphere. After the addition of ^{n}BuLi (11mmol, 2.5 M in hexane) by syringe at –78 °C, the mixture was stirred for 1 h. Subsequently R_1X was added and stirred at –78 °C for another 1 h, the mixture was warmed to room temperature or reflux for 12 h. The reaction was then quenched with HCl_{aq} (3 M), diluted with ethyl acetate and washed with saturated solution of sodium chloride. The organic layer was dried by anhydrous Na_2SO_4 and concentrated under vacuum. The crude product was purified by column chromatography on silica gel to give the desired alkynes. Alkynes of **2y**–**2ah** and **2ba**–**2ac** were prepared according to this procedure.

4. Supplementary Optimizing Reaction Parameters

Supplementary Table S1. Studying the Effect of Chromium Complexes on the *Trans*-Selective Alkyne Hydrogenation for Synthesis of *E*-olefin[a]

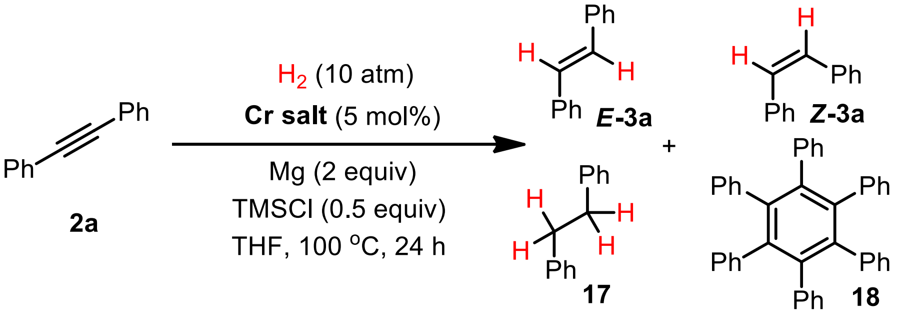

Entry	[Cr]	Yield (***E*-3a**+***Z*-3a**)	Selectivity $(E/Z)^b$	Yield (**17**)	Yield (**18**)
1	**1a**	88%	99:1	8%	nd
2	**1b**	61%	1:99	nd	10%
3	**1c**	6%	13:87	nd	nd
4	**1d**	60%	87:13	nd	13%
5	$CrCl_3$	75%	64:36	nd	nd
6	$Cr(acac)_3$	74%	90:10	nd	nd
7	–	nd	–	nd	nd

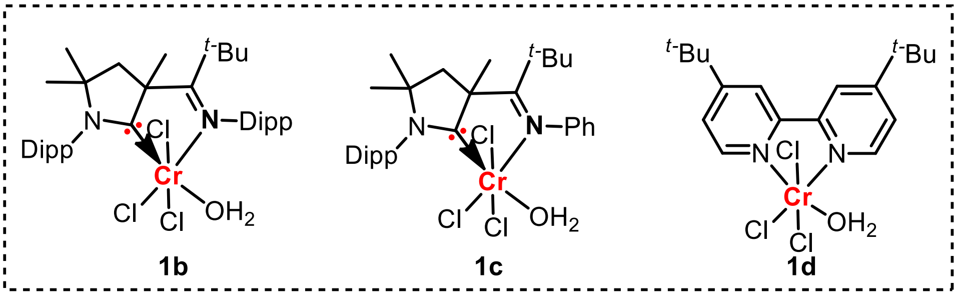

[a]Reaction conditions: Cr complex (5 mol %), **2a** (0.2 mmol), Mg (2 equiv), TMSCl (0.5 equiv), THF (2 mL), H_2 (10 atm), 100 °C, 24 h. GC yields were determined by using mesitylene as an internal standard. [b]The selectivity (*E*/*Z*) was determined by GC analysis prior to purification.

Supplementary Table S2. Studying the Effect of the Amounts of Mg, TMSCl and Temperature on the *Trans*-Selective Alkyne Hydrogenation for Synthesis of *E*-olefin[a]

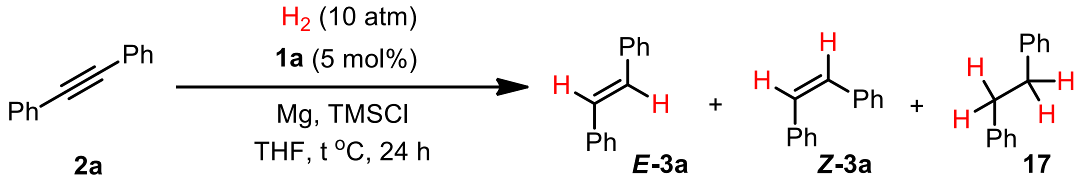

Entry	Mg (equiv)	TMSCl (equiv)	Temperature (°C)	Yield (***E*-3a**+***Z*-3a**)	Selectivity (E/Z)[b]	Yield (**17**)
1	2.5	1	90	76%	99:1	15%
2	2.5	none	90	nd	–	nd
3	2.5	0.2	90	54%	99:1	nd
4	2.5	0.5	90	75%	99:1	nd
5	2.5	0.5	100	88%	99:1	13%
6	none	0.5	100	nd	–	nd
7	0.2	0.5	100	trace	77:23	nd
8	0.5	0.5	100	57%	87:13	nd
9	1	0.5	100	72%	90:10	nd
10	1.5	0.5	100	82%	79:21	nd
11	2	0.5	100	89% (85%)[c]	99:1	8%
12[d]	2	0.5	100	nd	-	nd
13[e]	2	0.5	100	nd	-	nd

[a]Reaction conditions: **1a** (5 mol %), **2a** (0.2 mmol), Mg (x equiv), TMSCl (x equiv), THF (2 mL), H_2 (10 atm), T °C, 24 h. GC yields were determined by using mesitylene as internal standard. [b]The selectivity (Z/E) was determined by GC analysis prior to purification. [c]Isolated yield in parenthesis. [d]Manganese powder was used instead of magnesium. [e]Zinc powder was used instead of magnesium.

Supplementary Table S3. Studying the Effect of Chromium Complexes on the *Cis*-Selective Alkyne Hydrogenation for Synthesis of *Z*-olefin[a]

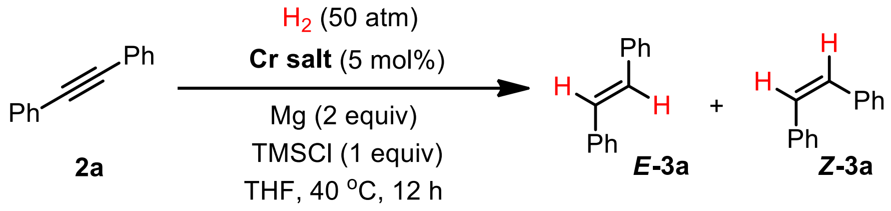

Entry	[Cr]	Yield (***Z*-3a**+***E*-3a**)	Selectivity (*Z*/*E*)[b]	Recovery (**2a**)
1	**1a**	25%	79:21	56%
2	**1b**	67%	87:13	24%
3	**1c**	53%	68:32	39%
4	**1d**	35%	72:28	46%
5	$CrCl_3$	34%	77:23	59%
6	Cr(acac)$_3$	trace	23:77	98%
7	–	nd	–	99%

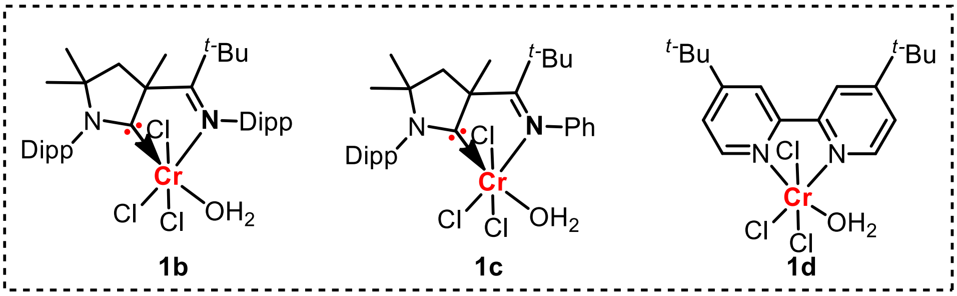

[a]Reaction conditions: Cr complex (5 mol %), **2a** (0.2 mmol), Mg (2 equiv), TMSCl (1 equiv), THF (2 mL), H_2 (50 atm), 40 °C, 12 h. GC yields were determined by using mesitylene as internal standard. [b]The selectivity (*Z*/*E*) was determined by GC analysis prior to purification.

Supplementary Table S4. Studying the Effect of the Amounts of Mg, TMSCl and Additive on the *Cis*-Selective Alkyne Hydrogenation for Synthesis of *Z*-olefin[a]

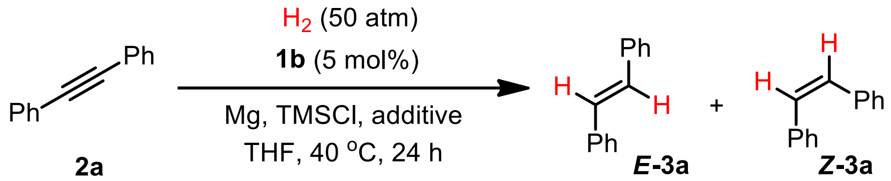

Entry	Mg (equiv)	TMSCl (equiv)	Additive (mg)	Yield (***Z*-3a**+***E*-3a**)	Recovery (**2a**)	Selectivity (*Z*/*E*)[b]
1	2.5	1	4 Å MS (150)	90%	nd	89:11
2	2.5	1	SiO_2 (150)	84%	9%	84:16
3	2	1	4 Å MS (150)	89%	nd	89:11
4	1.5	1	4 Å MS (150)	87%	5%	87:13
5	1	1	4 Å MS (150)	80%	16%	92:8
6	0.5	1	4 Å MS (150)	61%	37%	93:7
7	0.2	1	4 Å MS (150)	trace	97%	35:65
8	none	1	4 Å MS (150)	nd	99%	–
9	2	none	4 Å MS (150)	nd	99%	–
10	2	0.5	4 Å MS (150)	30%	66%	92:8
11	2	1.5	4 Å MS (150)	67%	nd	88:12
12	2	1	4 Å MS (25)	88% (86%)[e]	nd	93:7
13	2	1	4 Å MS (50)	87%	nd	90:10
14[c]	2	1	4 Å MS (50)	90%	nd	89:11
15[d]	2	1	4 Å MS (25)	90%	nd	70:30
16[f]	2	1	4 Å MS (25)	nd	97%	-
17[g]	2	1	4 Å MS (25)	nd	96%	-

[a]Reaction conditions: **1b** (5 mol %), **2a** (0.2 mmol), Mg (X equiv), TMSCl (X equiv), Additive (X mg), THF (2 mL), H_2 (50 atm), 40 °C, 24 h. GC yields were determined by using mesitylene as an internal standard. [b]The selectivity (*Z*/*E*) was determined by GC analysis prior to purification. [c]60 °C. [d]**1c** (5 mol%). [e]Isolated yield in parenthesis. [f]Manganese powder was used instead of magnesium. [g]Zinc powder was used instead of magnesium.

5. Supplementary General Procedure of CAAC-Phosphino-Cr-Catalyzed *Trans*-Selective Hydrogenation of Alkynes for the Synthesis of *E*-Olefins

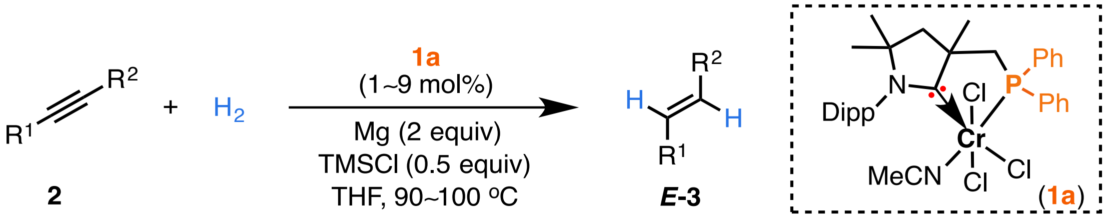

In a Schlenk tube were placed alkyne **2** (0.2 mmol), **1a** (1~9 mol %), Mg (10 mg), TMSCl (13 *u*L) and THF (2 mL) under atmosphere of nitrogen. The tube was quickly moved to a high–pressure autoclave, and stirred under atmosphere of H_2 (10 atm) at 90~100 °C for 24 h. After quenching with HCl_{aq} (2 mL, 1 M), the crude product was extracted with ethyl acetate (3 × 4 mL). The combined organic phases were dried over anhydrous Na_2SO_4 and concentrated under vacuum. The stereoselectivity of *E*-olefin relative to *Z*-stereomer was determined by GC analysis or ^{1}H NMR prior to purification. The crude product was purified by silica gel chromatography to afford the related olefin compound.

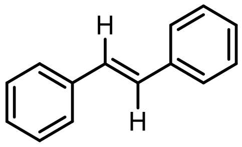

(*E*)-1,2-diphenylethene (*E*-3a)

The general procedure was applied to **1a** (6 mg, 0.01 mmol, 5 mol %), 1,2-diphenylethyne **2a** (36 mg, 0.2 mmol), Mg (10 mg), TMSCl (13 *u*L) and THF (2 mL). The reaction mixture was stirred under H_2 (10 atm) at 100 °C for 24 h. Then the resulting crude product was purified by column chromatography on silica gel with (PE:EA = 100:1) to afford the title compound as a white solid (31 mg, 85% yield, *E*:*Z* > 99:1). ^{1}H NMR (400 MHz, $CDCl_3$): δ 7.54–7.47 (m, 4H), 7.39–7.30 (m, 4H), 7.28–7.22 (m, 2H), 7.10 (s, 2H); ^{13}C NMR (100 MHz, $CDCl_3$): δ 137.5, 128.8, 128.8, 127.8, 126.7. Spectroscopic data are in accordance with those described in the literature.[5]

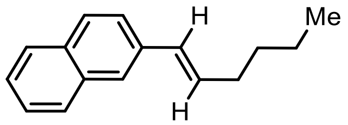

(*E*)-2-(hex-1-en-1-yl)naphthalene (*E*-3b)

The general procedure was applied to **1a** (1 mg, 0.002 mmol, 1 mol %), 2-(hex-1-yn-1-yl)naphthalene **2b** (42 mg, 0.2 mmol), Mg (10 mg), TMSCl (13 *u*L) and THF (2 mL). The reaction mixture was stirred under H_2 (10 atm) at 90 °C for 24 h. Then the resulting crude product was purified by column chromatography on silica gel with (PE:EA = 100:1) to afford the title compound as a colorless oil (37 mg, 88% yield, *E*:*Z* = 99:1). ^{1}H NMR (400 MHz, $CDCl_3$): δ 7.81–7.73 (m, 3H), 7.68 (s, 1H), 7.61–7.55 (m, 1H), 7.47–7.37 (m, 2H), 6.55 (d, *J* = 15.6 Hz, 1H), 6.37 (dt, *J* = 15.6, 6.8 Hz, 1H), 2.32–2.23 (m, 2H), 1.55–1.46 (m, 2H), 1.46–1.36 (m, 2H), 0.96 (t, *J* = 7.2 Hz, 3H). ^{13}C NMR (100 MHz, $CDCl_3$): δ 135.6, 133.9, 132.8, 131.9, 13.0, 128.2, 128.0, 127.8, 126.2, 125.5, 125.4, 123.7, 33.0, 31.7, 22.5, 14.1. Spectroscopic data are in accordance with those described in the literature.[8]

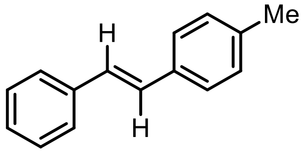

(*E*)-1-methyl-4-styrylbenzene (*E*-3c)

The general procedure was applied to **1a** (4 mg, 0.006 mmol, 3 mol %), 1-methyl-4-(phenylethynyl)benzene **2c** (38 mg, 0.2 mmol), Mg (10 mg), TMSCl (13 *u*L) and THF (2 mL). The reaction mixture was stirred under H_2 (10 atm) at 90 °C for 24 h. Then the resulting crude product was purified by column chromatography on silica gel with (PE:EA = 100:1) to afford the title compound as a white solid (28 mg, 73% yield, *E*:*Z* > 99:1). ^{1}H NMR (400 MHz, $CDCl_3$): δ 7.52–7.47 (m,2H), 7.41 (d, *J* = 8.0 Hz, 2H), 7.37–7.31 (m, 2H), 7.26–7.21 (m, 1H), 7.16 (d, *J* = 8.0 Hz, 2H), 7.10 (d, *J* = 16.4 Hz, 1H), 7.04 (d, *J* = 16.4 Hz, 1H), 2.35 (s, 3H). ^{13}C NMR (100 MHz, $CDCl_3$): δ 137.7, 134.7, 129.5, 128.8, 128.8, 127.8, 127.5, 126.6, 126.5, 21.4.

Spectroscopic data are in accordance with those described in the literature.[5]

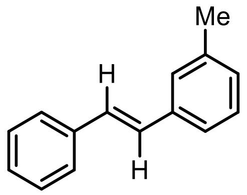

(*E*)-1-methyl-3-styrylbenzene (*E*-3d)

The general procedure was applied to **1a** (3 mg, 0.005 mmol, 2.5 mol %), 1-methyl-3-(phenylethynyl)benzene **2d** (38 mg, 0.2 mmol), Mg (10 mg), TMSCl (13 *u*L) and THF (2 mL). The reaction mixture was stirred under H_2 (10 atm) at 90 °C for 24 h. Then the resulting crude product was purified by column chromatography on silica gel with (PE:EA = 100:1) to afford the title compound as a white solid (29 mg, 75% yield, *E*:*Z* = 97:3). ^{1}H NMR (400 MHz, $CDCl_3$): δ 7.55–7.47 (m, 2H), 7.39–7.30 (m, 4H), 7.28–7.21 (m, 2H), 7.14–7.0 (m, 2H), 2.38 (s, 3H); ^{13}C NMR (100 MHz, $CDCl_3$): δ 138.4, 137.6, 137.4, 128.9, 128.8, 128.7, 128.6, 128.6, 127.7, 127.4, 126.6, 123.9, 21.6. Spectroscopic data are in accordance with those described in the literature.[5]

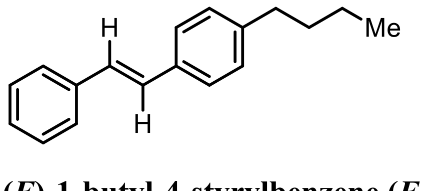

(*E*)-1-butyl-4-styrylbenzene (*E*-3e)

The general procedure was applied to **1a** (4 mg, 0.006 mmol, 3 mol %), 1-butyl-4-(phenylethynyl)benzene **2e** (47 mg, 0.2 mmol), Mg (10 mg), TMSCl (13 *u*L) and THF (2 mL). The reaction mixture was stirred under H_2 (10 atm) at 90 °C for 24 h. Then the resulting crude product was purified by column chromatography on silica gel with (PE:EA = 100:1) to afford the title compound as a white solid (37 mg, 79% yield, *E*:*Z* > 99:1). ^{1}H NMR (400 MHz, $CDCl_3$): δ 7.50 (d, *J* = 7.4 Hz, 2H), 7.43 (d, *J* = 8.0 Hz, 2H), 7.35 (t, *J* = 7.6 Hz, 2H), 7.27–7.23 (m, 1H), 7.17 (d, *J* = 8.0 Hz, 2H),

7.10 (d, J = 16.4 Hz, 1H), 7.05 (d, J = 16.4 Hz, 1H), 2.61 (t, J = 7.6 Hz, 2H), 1.68–1.56 (m, 2H), 1.41–1.31 (m, 2H), 0.93 (t, J = 7.2 Hz, 3H); ^{13}C NMR (100 MHz, $CDCl_3$): δ 142.8, 137.7, 134.9, 128.9, 128.8, 128.8, 127.9, 127.5, 126.6, 126.5, 35.6, 33.7, 22.5, 14.1. Spectroscopic data are in accordance with those described in the literature.[9]

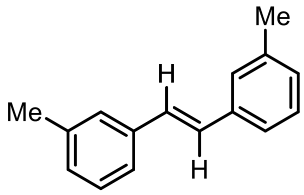

(*E*)-1,2-di-*m*-tolylethene (*E*-3f)

The general procedure was applied to **1a** (4 mg, 0.006 mmol, 3 mol %), 1,2-di-*m*-tolylethyne **2f** (41 mg, 0.2 mmol), Mg (10 mg), TMSCl (13 *u*L) and THF (2 mL). The reaction mixture was stirred under H_2 (10 atm) at 90 °C for 24 h. Then the resulting crude product was purified by column chromatography on silica gel with (PE:EA = 100:1) to afford the title compound as a white solid (35 mg, 84% yield, *E*:*Z* > 99:1). ^{1}H NMR (400 MHz, $CDCl_3$): δ 7.24 (d, J = 10.4 Hz, 4H), 7.20–7.14 (m, 2H), 7.02–6.97 (m, 4H), 2.30 (s, 6H). ^{13}C NMR (100 MHz, $CDCl_3$): δ 138.3, 137.5, 128.7, 128.7, 128.5, 127.3, 123.8, 21.6, 1.2. Spectroscopic data are in accordance with those described in the literature.[10]

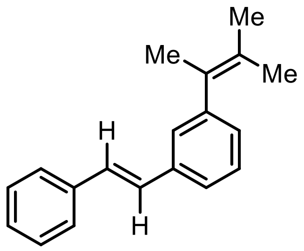

(*E*)-1-(3-methylbut-2-en-2-yl)-3-styrylbenzene (*E*-3g)

The general procedure was applied to **1a** (5 mg, 0.008 mmol, 4 mol %), 1-(3-methylbut-2-en-2-yl)-3-(phenyleth-ynyl)benzene **2g** (49 mg, 0.2 mmol), Mg (10

mg), TMSCl (13 *u*L) and THF (2 mL). The reaction mixture was stirred under H_2 (10 atm) at 90 °C for 24 h. Then the resulting crude product was purified by column chromatography on silica gel with (PE:EA = 100:1) to afford the title compound as a white solid (42 mg, 85% yield, *E*:*Z* = 99:1). mp. = 66–68 °C. ^{1}H NMR (400 MHz, $CDCl_3$): δ 7.51 (d, *J* = 7.4 Hz, 2H), 7.46 (d, *J* = 8.2 Hz, 2H), 7.35 (t, *J* = 7.6 Hz, 2H), 7.27–7.21 (m, 1H), 7.16–7.07 (m, 4H), 1.97 (s, 3H), 1.82 (s, 3H), 1.63 (s, 3H); ^{13}C NMR (100 MHz, $CDCl_3$): δ 145.0, 137.7, 135.0, 129.8, 129.0, 128.8, 128.8, 128.1, 127.8, 127.6, 126.6, 126.3, 22.3, 20.8, 20.7. HRMS (ESI, m/z): calcd for $C_{19}H_{21}$ $[M+H]^+$: 249.1638, found: 249.1637.

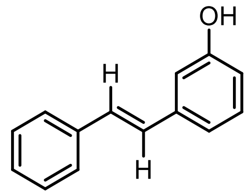

(*E*)-3-styrylphenol (*E*-3h)

The general procedure was applied to **1a** (6 mg, 0.01 mmol, 5 mol %), 3-(phenylethynyl)phenol **2h** (39 mg, 0.2 mmol), Mg (10 mg), TMSCl (38 *u*L) and THF (2 mL). The reaction mixture was stirred under H_2 (10 atm) at 100 °C for 24 h. Then the resulting crude product was purified by column chromatography on silica gel with (PE:EA = 100:1) to afford the title compound as a white solid (34 mg, 87% yield, *E*:*Z* = 90:10). ^{1}H NMR (400 MHz, $CDCl_3$): δ 7.50 (d, *J* = 7.4 Hz, 2H), 7.35 (t, *J* = 7.6 Hz, 2H), 7.30–7.18 (m, 2H), 7.12–7.03 (m, 3H), 7.01–6.95 (m, 1H), 6.74 (dd, *J* = 8.0, 1.6 Hz, 1H), 4.88 (s, 1H); ^{13}C NMR (100 MHz, $CDCl_3$): δ 155.9, 139.3, 137.3, 130.0, 129.4, 128.8, 128.4, 127.9, 126.7, 119.6, 114.8, 113.1. Spectroscopic data are in accordance with those described in the literature.[11]

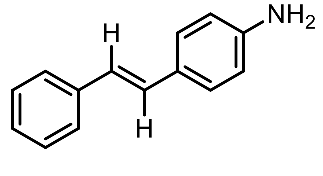

(*E*)-4-styrylaniline (*E*-3i)

The general procedure was applied to **1a** (6 mg, 0.01 mmol, 5 mol %), 4-(phenylethynyl)aniline **2i** (39 mg, 0.2 mmol), Mg (10 mg), TMSCl (13 *u*L) and THF (2 mL). The reaction mixture was stirred under H_2 (10 atm) at 90 °C for 24 h. Then the resulting crude product was purified by column chromatography on silica gel with (PE:EA = 10:1) to afford the title compound as a yellow solid (33 mg, 84% yield, *E*:*Z* = 95:5). ^{1}H NMR (400 MHz, $CDCl_3$): δ 7.47 (d, *J* = 7.6 Hz, 2H), 7.39–7.28 (m, 4H), 7.20 (t, *J* = 7.4 Hz, 1H), 7.02 (d, *J* = 16.4 Hz, 1H), 6.92 (d, *J* = 16.4 Hz, 1H), 6.67 (d, *J* = 8.4 Hz, 2H), 3.73 (s, 2H); ^{13}C NMR (100 MHz, $CDCl_3$): δ 146.3, 138.1, 128.8, 128.7, 128.2, 127. 9, 127.0, 126.2, 125.3, 115.4. Spectroscopic data are in accordance with those described in the literature.[5]

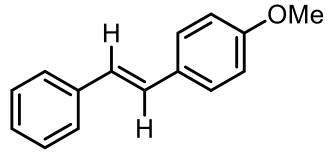

(*E*)-1-methoxy-4-styrylbenzene (*E*-3j)

The general procedure was applied to **1a** (6 mg, 0.01 mmol, 5 mol %), 1-methoxy-4-(phenylethynyl)benzene **2j** (42 mg, 0.2 mmol), Mg (10 mg), TMSCl (13 *u*L) and THF (2 mL). The reaction mixture was stirred under H_2 (10 atm) at 90 °C for 24 h. Then the resulting crude product was purified by column chromatography on silica gel with (PE:EA = 100:1) to afford the title compound as a white solid (36 mg, 90% yield, *E*:*Z* = 97:3). ^{1}H NMR (400 MHz, $CDCl_3$): δ 7.52–7.41 (m, 4H), 7.38–7.30 (m, 2H), 7.25–7.19 (m, 1H), 7.06 (d, *J* = 16.4 Hz, 1H), 6.97 (d, *J* = 16.4 Hz, 1H), 6.93–6.86 (m, 2H), 3.82 (s, 3H). ^{13}C NMR (100 MHz, $CDCl_3$): δ 159.5, 137.8, 130.3, 128.8, 128.4, 127.9, 127.4, 126.8, 126.4, 55.5. Spectroscopic data are in accordance with those described in the literature.[5]

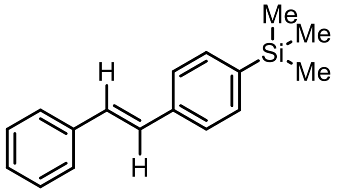

(*E*)-trimethyl(4-styrylphenyl)silane (*E*-3k)

The general procedure was applied to **1a** (5 mg, 0.008 mmol, 4 mol %), trimethyl(4-(phenylethynyl)phenyl)silane **2k** (50 mg, 0.2 mmol), Mg (10 mg), TMSCl (13 *u*L) and THF (2 mL). The reaction mixture was stirred under H_2 (10 atm) at 90 °C for 24 h. Then the resulting crude product was purified by column chromatography on silica gel with (PE:EA = 100:1) to afford the title compound as a white solid (40 mg, 80% yield, *E*:*Z* > 99:1). ^{1}H NMR (400 MHz, $CDCl_3$): δ 7.58–7.44 (m, 6H), 7.36 (t, *J* = 7.6 Hz, 2H), 7.29–7.22 (m, 1H), 7.15 (d, *J* = 16.4 Hz, 1H), 7.10 (d, *J* = 16.4 Hz, 1H), 0.28 (s, 9H). ^{13}C NMR (100 MHz, $CDCl_3$): δ 141.2, 138.9, 138.5, 134.9, 130.1, 129.8, 128.8, 127.7, 127.0, 0.03. Spectroscopic data are in accordance with those described in the literature.[12]

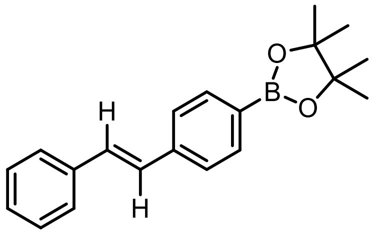

(*E*)-4,4,5,5-tetramethyl-2-(4-styrylphenyl)-1,3,2-dioxaborolane (*E*-3l)

The general procedure was applied to **1a** (5 mg, 0.008 mmol, 4 mol %), 4,4,5,5-tetramethyl-2-(4-(phenylethynyl)phenyl)-1,3,2-dioxaborolane **2l** (61 mg, 0.2 mmol), Mg (10 mg), TMSCl (13 *u*L) and THF (2 mL). The reaction mixture was stirred under H_2 (10 atm) at 90 °C for 24 h. Then the resulting crude product was purified by column chromatography on silica gel with (PE:EA = 100:1) to afford the title compound as a white solid (40 mg, 65% yield, *E*:*Z* = 86:14). ^{1}H NMR (400 MHz, $CDCl_3$): δ 7.80 (d, *J* = 8.0 Hz, 2H), 7.55–7.47 (m, 4H), 7.40–7.33 (m, 2H), 7.28–7.24 (m, 1H), 7.18 (d, *J* = 16.4 Hz, 1H), 7.11 (d, *J* = 16.4 Hz, 1H), 1.35 (s, 12H); ^{13}C NMR

(100 MHz, $CDCl_3$): δ 140.2, 137.3, 135.3, 129.8, 128.8, 128.8, 127.9, 126.8, 125.9, 83.9, 25.0. Spectroscopic data are in accordance with those described in the literature.[12]

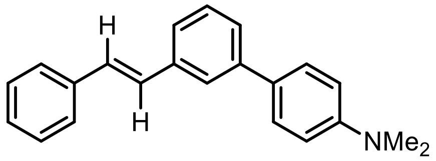

(*E*)-*N*,*N*-dimethyl-3'-styryl-[1,1'-biphenyl]-4-amine (*E*-3m)

The general procedure was applied to **1a** (12 mg, 0.018 mmol, 9 mol %), *N,N*-dimethyl-3'-(phenylethynyl)-[1,1'-biphenyl]-4-amine **2m** (59 mg, 0.2 mmol), Mg (10 mg), TMSCl (13 *u*L) and THF (2 mL). The reaction mixture was stirred under H_2 (10 atm) at 90 °C for 24 h. Then the resulting crude product was purified by column chromatography on silica gel with (PE:EA = 10:1) to afford the title compound as a yellow solid (56 mg, 93% yield, *E*:*Z* = 96:4). mp. = 56–58 °C. ^{1}H NMR (400 MHz, $CDCl_3$): δ 7.69 (s, 1H), 7.60–7.49 (m, 4H), 7.48–7.32 (m, 5H), 7.31–7.21 (m, 1H), 7.16 (s, 2H), 6.82 (d, J = 8.8 Hz, 2H), 3.00 (s, 6H). ^{13}C NMR (100 MHz, $CDCl_3$): δ 150.2, 141.8, 137.8, 137.6, 129.2, 129.1, 129.1, 128.8, 127.9, 127.7, 126.7, 125.9, 124.8, 124.3, 40.7. HRMS (ESI, m/z): calcd for $C_{22}H_{22}N$ $[M+H]^+$: 300.1747, found: 300.1748.

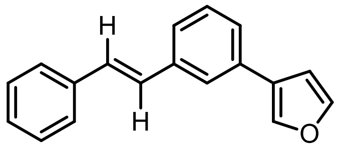

(*E*)-3-(3-styrylphenyl)furan (*E*-3n)

The general procedure was applied to **1a** (12 mg, 0.018 mmol, 9 mol %), 3-(3-(phenylethynyl)phenyl)furan **2n** (49 mg, 0.2 mmol), Mg (10 mg), TMSCl (13 *u*L) and THF (2 mL). The reaction mixture was stirred under H_2 (10 atm) at 90 °C for 24 h. Then the resulting crude product was purified by column chromatography on silica gel with (PE:EA = 100:1) to afford the title compound as a white solid (47 mg, 95%

yield, E:Z = 98:2). mp. = 66–68 °C. ^{1}H NMR (400 MHz, $CDCl_3$): δ 7.76 (s, 1H), 7.60 (s, 1H), 7.55–7.46 (m, 3H), 7.44–7.39 (m, 1H), 7.39–7.32 (m, 4H), 7.26 (t, J = 7.4 Hz, 1H), 7.13 (s, 2H), 6.73 (d, J = 1.2 Hz, 1H); ^{13}C NMR (100 MHz, $CDCl_3$): δ 143.8, 138.8, 138.0, 137.4, 133.0, 129.3, 129.2, 128.8, 128.6, 127.9, 126.7, 126.5, 125.3, 125.2, 124.2, 109.1. HRMS (ESI, m/z): calcd for $C_{18}H_{15}O$ $[M+H]^+$: 247.1117, found: 247.1122.

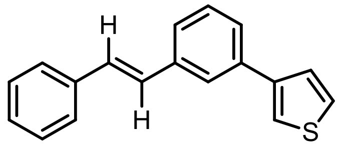

(*E*)-3-(3-styrylphenyl)thiophene (*E*-3o)

The general procedure was applied to **1a** (12 mg, 0.018 mmol, 9 mol %), 3-(3-(phenylethynyl)phenyl)thiophene **2o** (52 mg, 0.2 mmol), Mg (10 mg), TMSCl (13 *u*L) and THF (2 mL). The reaction mixture was stirred under H_2 (10 atm) at 90 °C for 24 h. Then the resulting crude product was purified by column chromatography on silica gel with (PE:EA = 100:1) to afford the title compound as a white solid (44 mg, 84% yield, E:Z = 92:8). mp. = 89–91 °C. ^{1}H NMR (400 MHz, $CDCl_3$): δ 7.72 (s, 1H), 7.56–7.35 (m, 10H), 7.30–7.23 (m, 1H), 7.16 (s, 2H); ^{13}C NMR (100 MHz, $CDCl_3$): δ 142.4, 138.0, 137.4, 136.4, 129.3, 129.2, 128.9, 128.7, 127.9, 126.7, 126.6, 126.4, 126.0, 125.4, 124.9, 120.7. HRMS (ESI, m/z): calcd for $C_{18}H_{15}S$ $[M+H]^+$: 263.0889, found: 263.0886.

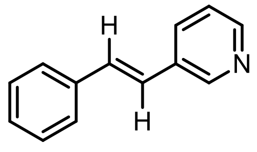

(*E*)-3-styrylpyridine (*E*-3p)

The general procedure was applied to **1a** (4 mg, 0.006 mmol, 3 mol %), 3-(phenylethynyl)pyridine **2p** (36 mg, 0.2 mmol), Mg (10 mg), TMSCl (13 *u*L) and THF (2 mL). The reaction mixture was stirred under H_2 (10 atm) at 90 °C for 24 h.

Then the resulting crude product was purified by column chromatography on silica gel with (PE:EA = 4:1) to afford the title compound as a white solid (14 mg, 39% yield, E:Z = 94:6). ^{1}H NMR (400 MHz, $CDCl_3$): δ 8.72 (d, J = 2.0 Hz, 1H), 8.49 (dd, J = 4.8, 1.6 Hz, 1H), 7.83 (dt, J = 8.0, 2.0 Hz, 1H), 7.56–7.49 (m, 2H), 7.42–7.35 (m, 2H), 7.32–7.25 (m, 2H), 7.17 (d, J = 16.4 Hz, 1H), 7.07 (d, J = 16.4 Hz, 1H); ^{13}C NMR (100 MHz, $CDCl_3$): δ 148.7, 136.8, 133.1, 132.8, 131.0, 128.9, 128.4, 126.8, 125.0, 123.7. Spectroscopic data are in accordance with those described in the literature.[13]

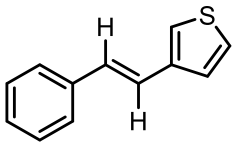

(*E*)-3-styrylthiophene (*E*-3q)

The general procedure was applied to **1a** (4 mg, 0.006 mmol, 3 mol %), 3-(phenylethynyl)thiophene **2q** (37 mg, 0.2 mmol), Mg (10 mg), TMSCl (13 *u*L) and THF (2 mL). The reaction mixture was stirred under H_2 (10 atm) at 100 °C for 24 h. Then the resulting crude product was purified by column chromatography on silica gel with (PE:EA = 100:1) to afford the title compound as a white solid (26 mg, 70% yield, E:Z = 98:2). ^{1}H NMR (400 MHz, $CDCl_3$): δ 7.47 (d, J = 7.4 Hz, 2H), 7.39–7.29 (m, 4H), 7.29–7.21 (m, 2H), 7.12 (d, J = 16.4 Hz, 1H), 6.95 (d, J = 16.4 Hz, 1H); ^{13}C NMR (100 MHz, $CDCl_3$): δ 140.3, 137.5, 128.8, 127.6, 126.4, 126.3, 125.1, 123.0, 122.5. Spectroscopic data are in accordance with those described in the literature.[14]

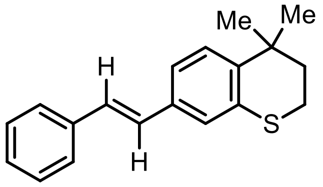

(*E*)-4,4-dimethyl-6-styrylthiochroman (*E*-3r)

The general procedure was applied to **1a** (5 mg, 0.008 mmol, 4 mol %),

4,4-dimethyl-6-(phenylethynyl)thiochroman **2r** (56 mg, 0.2 mmol), Mg (10 mg), TMSCl (13 *u*L) and THF (2 mL). The reaction mixture was stirred under H_2 (10 atm) at 100 °C for 24 h. Then the resulting crude product was purified by column chromatography on silica gel with (PE:EA = 100:1) to afford the title compound as a white solid (52 mg, 93% yield, *E*:*Z* = 94:6). mp. = 73–75 °C. ^{1}H NMR (400 MHz, $CDCl_3$): δ 7.53–7.47 (m, 2H), 7.45 (d, *J* = 1.8 Hz, 1H), 7.37–7.30 (m, 2H), 7.26–7.20 (m, 2H), 7.07 (d, *J* = 8.2 Hz, 2H), 7.05 (d, *J* = 16.4 Hz, 1H), 7.00 (d, *J* = 16.4 Hz, 1H), 3.11–2.97 (m, 2H), 2.03–1.92 (m, 2H), 1.37 (s, 6H). ^{13}C NMR (100 MHz, $CDCl_3$): δ 142.2, 137.7, 133.5, 131.6, 128.8, 128.8, 127.5, 127.3, 127.0, 126.5, 125.3, 123.8, 37.8, 33.2, 30.3, 23.3. HRMS (ESI, m/z): calcd for $C_{19}H_{21}S$ $[M+H]^+$: 281.1358, found: 281.1359.

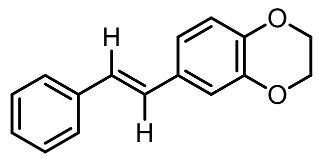

(*E*)-6-styryl-2,3-dihydrobenzo[*b*][1,4]dioxine (*E*-3s)

The general procedure was applied to **1a** (9 mg, 0.014 mmol, 7 mol %), 6-(phenylethynyl)-2,3-dihydrobenzo-[*b*][1,4]dioxine **2s** (47 mg, 0.2 mmol), Mg (10 mg), TMSCl (13 *u*L) and THF (2 mL). The reaction mixture was stirred under H_2 (10 atm) at 90 °C for 24 h. Then the resulting crude product was purified by column chromatography on silica gel with (PE:EA = 100:1) to afford the title compound as a white solid (40 mg, 83% yield, *E*:*Z* = 98:2). ^{1}H NMR (400 MHz, $CDCl_3$): δ 7.47 (d, *J* = 7.6 Hz, 2H), 7.33 (t, *J* = 7.6 Hz, 2H), 7.26–7.19 (m, 1H), 7.05–6.90 (m, 4H), 6.84 (d, *J* = 8.4 Hz, 1H), 4.26 (s, 4H); ^{13}C NMR (100 MHz, $CDCl_3$): δ 143.8, 143.5, 137.6, 131.3, 128.8, 128.2, 127.4, 127.3, 126.5, 120.2, 117.6, 115.1, 64.6, 64.5. Spectroscopic data are in accordance with those described in the literature.[15]

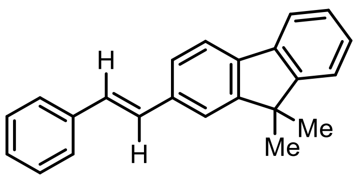

(*E*)-9,9-dimethyl-2-styryl-9*H*-fluorene (*E*-3t)

The general procedure was applied to **1a** (8 mg, 0.012 mmol, 6 mol %), 9,9-dimethyl-2-(phenylethynyl)-9*H*-fluorene **2t** (59 mg, 0.2 mmol), Mg (10 mg), TMSCl (13 *u*L) and THF (2 mL). The reaction mixture was stirred under H_2 (10 atm) at 90 °C for 24 h. Then the resulting crude product was purified by column chromatography on silica gel with (PE:EA = 100:1) to afford the title compound as a white solid (52 mg, 87% yield, *E*:*Z* = 92:8). mp. = 98–110 °C. ^{1}H NMR (400 MHz, $CDCl_3$): δ 7.75–7.65 (m, 2H), 7.63–7.50 (m, 3H), 7.50–7.40 (m, 2H), 7.40–7.10 (m, 7H), 1.51 (d, *J* = 4.2 Hz, 6H). ^{13}C NMR (100 MHz, $CDCl_3$): δ 154.3, 154.1, 139.1, 139.0, 137.7, 136.7, 129.3, 128.8, 128.3, 127.6, 127.4, 127.2, 126.6, 126.1, 122.7, 120.6, 120.4, 120.2, 46.9, 27.4. HRMS (ESI, m/z): calcd for $C_{23}H_{21}$ $[M+H]^+$: 297.1638, found: 297.1641.

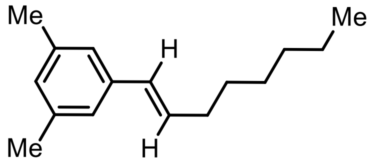

(*E*)-1,3-dimethyl-5-(oct-1-en-1-yl)benzene (*E*-3u)

The general procedure was applied to **1a** (3 mg, 0.004 mmol, 2 mol %), 1,3-dimethyl-5-(oct-1-yn-1-yl)benzene **2u** (43 mg, 0.2 mmol), Mg (10 mg), TMSCl (13 *u*L) and THF (2 mL). The reaction mixture was stirred under H_2 (10 atm) at 90 °C for 24 h. Then the resulting crude product was purified by column chromatography on silica gel with (PE:EA = 100:1) to afford the title compound as a colorless oil (37 mg, 85% yield, *E*:*Z* = 99:1). ^{1}H NMR (400 MHz, $CDCl_3$): δ 6.97 (s, 2H), 6.84 (s, 1H), 6.32 (d, *J* = 15.6 Hz, 1H), 6.20 (dt, *J* = 15.6, 6.8 Hz, 1H), 2.30 (s, 6H), 2.24–2.16 (m, 2H), 1.49–1.42 (m, 2H), 1.36–1.27 (m, 6H), 0.90 (t, *J* = 6.8 Hz, 3H). ^{13}C NMR (100

MHz, $CDCl_3$): δ 138.0, 131.0, 129.9, 128.6, 124.0, 33.2, 31.9, 29.6, 29.1, 22.8, 21.4, 14.3. Spectroscopic data are in accordance with those described in the literature.[16]

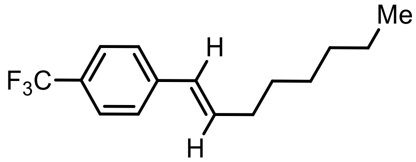

(*E*)-1-(oct-1-en-1-yl)–4–(trifluoromethyl)benzene (*E*-3v)

The general procedure was applied to **1a** (3 mg, 0.004 mmol, 2 mol %), 1-(oct-1-yn-1-yl)-4-(trifluoromethyl)benzene **2v** (51 mg, 0.2 mmol), Mg (10 mg), TMSCl (13 *u*L) and THF (2 mL). The reaction mixture was stirred under H_2 (10 atm) at 90 °C for 24 h. Then the resulting crude product was purified by column chromatography on silica gel with (PE:EA = 100:1) to afford the title compound as a colorless oil (46 mg, 89% yield, *E*:*Z* > 99:1). ^{1}H NMR (400 MHz, $CDCl_3$): δ 7.55 (d, *J* = 8.0 Hz, 2H), 7.43 (d, *J* = 8.0 Hz, 2H), 6.42 (d, *J* = 16.0 Hz, 1H), 6.35 (dt, *J* = 15.8, 6.4 Hz, 1H), 2.25 (dd, *J* = 14.0, 7.0 Hz, 2H), 1.55–1.44 (m, 2H), 1.43–1.30 (m, 6H), 0.92 (t, *J* = 6.8 Hz, 3H); ^{13}C NMR (100 MHz, $CDCl_3$): δ 141.6 (q, *J* = 1.1 Hz), 134.3, 128.8 (q, *J* = 32.0 Hz), 128.7, 126.2, 125.6 (q, *J* = 3.8 Hz), 124.5 (q, *J* = 270.0 Hz), 33.3, 31.9, 29.3, 29.1, 22.8, 14.2; ^{19}F NMR (376 MHz, $CDCl_3$): δ -62.40. Spectroscopic data are in accordance with those described in the literature.[15]

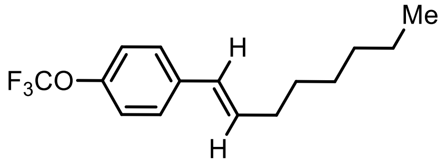

(*E*)-1-(oct-1-en-1-yl)-4-(trifluoromethoxy)benzene (*E*-3w)

The general procedure was applied to **1a** (3 mg, 0.004 mmol, 2 mol %), 1-(oct-1-yn-1-yl)-4-(trifluoromethoxy)benzene **2w** (54 mg, 0.2 mmol), Mg (10 mg), TMSCl (13 *u*L) and THF (2 mL). The reaction mixture was stirred under H_2 (10 atm) at 90 °C for 24 h. Then the resulting crude product was purified by column chromatography on silica gel with (PE:EA = 100:1) to afford the title compound as a

colorless oil (50 mg, 92% yield, E:Z > 99:1). ^1H NMR (400 MHz, $CDCl_3$): δ 7.38–7.31 (m, 2H), 7.14 (d, J = 8.0 Hz, 2H), 6.36 (d, J = 15.6 Hz, 2H), 6.21 (dt, J = 15.6, 6.8 Hz, 1H), 2.27–2.17 (m, 2H), 1.52–1.43 (m, 2H), 1.39–1.27 (m, 6H), 0.91 (t, J = 7.2 Hz, 3H); ^{13}C NMR (100 MHz, $CDCl_3$): δ 148.1 (q, J = 1.9 Hz), 136.9, 132.6, 128.5, 127.2, 121.2, 120.68 (q, J = 255.0 Hz), 33.2, 31.9, 29.4, 29.1, 22.8, 14.2; ^{19}F NMR (376 MHz, $CDCl_3$): δ -57.9. HRMS (ESI, m/z): calcd for $C_{15}H_{20}F_3O$ $[M+H]^+$: 273.1461, found: 273.1458.

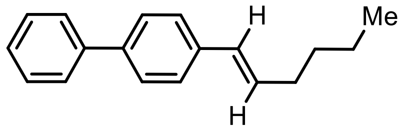

(*E*)-4-(hex-1-en-1-yl)-1,1'-biphenyl (*E*-3x)

The general procedure was applied to **1a** (3 mg, 0.004 mmol, 2 mol %), 4-(hex-1-yn-1-yl)-1,1'-biphenyl **2x** (47 mg, 0.2 mmol), Mg (10 mg), TMSCl (13 *u*L) and THF (2 mL). The reaction mixture was stirred under H_2 (10 atm) at 90 °C for 24 h. Then the resulting crude product was purified by column chromatography on silica gel with (PE:EA = 100:1) to afford the title compound as a colorless oil (43 mg, 92% yield, E:Z > 99:1). ^1H NMR (400 MHz, $CDCl_3$): δ 7.64–7.58 (m, 2H), 7.57–7.52 (m, 2H), 7.47–7.40 (m, 4H), 7.37–7.31 (m, 1H), 6.43 (d, J = 15.8 Hz, 1H), 6.29 (dt, J = 15.8, 6.8 Hz, 1H), 2.30–2.18 (m, 2H), 1.52–1.35 (m, 4H), 0.95 (t, J = 7.2 Hz, 3H). ^{13}C NMR (100 MHz, $CDCl_3$): δ 141.0, 139.7, 137.2, 131.6, 129.4, 128.9, 127.3, 127.3, 127.0, 126.4, 32.9, 31.7, 22.4, 14.1. Spectroscopic data are in accordance with those described in the literature.[17]

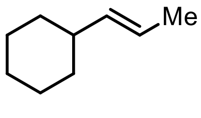

(*E*)-prop-1-en-1-ylcyclohexane (*E*-3y)

The general procedure was applied to **1a** (18 mg, 0.028 mmol, 7 mol %), pent-1-yn-1-ylcyclohexane **2y** (49 mg, 0.4 mmol), Mg (19 mg), TMSCl (25 *u*L) and

THF (4 mL). The reaction mixture was stirred under H_2 (5 atm) at 80 °C for 36 h. The high volatility of the compound precluded isolation. The yield was determined by GC using ^{n}Tridecane as internal standard (71% yield, *E*:*Z* = 90:10). GC–MS (EI) calculated for C_9H_{16} (M) 124.1, found 124.0. ^1H NMR (400 MHz, $CDCl_3$) δ 5.45–5.37 (m, 1H, $C_{olefin}H$); ^{13}C NMR (100 MHz, $CDCl_3$) δ 137.9 (C_{olefin}), 122.1 (C_{olefin}). The related NMR data are in accordance with the reported literature.[17]

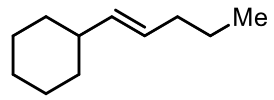

(*E*)-pent-1-en-1-ylcyclohexane (*E*-3z)

The general procedure was applied to **1a** (20 mg, 0.032 mmol, 8 mol %), pent-1-yn-1-ylcyclohexane **2z** (61 mg, 0.4 mmol), Mg (19 mg), 4,4'-di-*tert*-butyl-2,2'-bipyridine (4 mg, 0.016 mmol, 4 mol %), TMSCl (25 *u*L) and THF (4 mL). The reaction mixture was stirred under H_2 (10 atm) at 100 °C for 24 h. Then the resulting crude product was purified by column chromatography on silica gel with PE to afford the title compound as a colorless oil (46 mg, 75% yield, *E*:*Z* = 90:10). ^1H NMR (400 MHz, $CDCl_3$) δ 5.38–5.33 (m, 2H), 2.07–1.86 (m, 3H), 1.74–1.63 (m, 4H), 1.30–1.14 (m, 8H), 0.91–0.85 (m, 3H); ^{13}C NMR (100 MHz, $CDCl_3$) δ 136.8, 127.6, 40.9, 34.9, 33.5, 31.8, 26.3, 22.8, 14.3. HRMS (ESI, m/z): calcd for $C_{11}H_{20}Na$ $[M+Na]^+$: 175.1457, found: 175.1478.

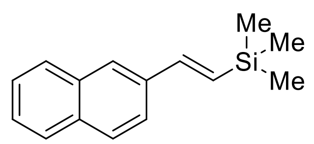

(*E*)-trimethyl(2-(naphthalen-2-yl)vinyl)silane (*E*-3aa)

The general procedure was applied to **1a** (4 mg, 0.006 mmol, 3 mol %), trimethyl(naphthalen-2-ylethynyl)silane **2aa** (45 mg, 0.2 mmol), Mg (10 mg), TMSCl (13 *u*L) and THF (2 mL). The reaction mixture was stirred under H_2 (10 atm) at 100 °C for 24 h. Then the resulting crude product was purified by column chromatography

on silica gel with (PE:EA = 100:1) to afford the title compound as a colorless liquid (31 mg, 85% yield, E:Z > 99:1). ^{1}H NMR (400 MHz, $CDCl_3$) δ 7.84–7.78 (m, 4H), 7.70 (dd, *J* = 8.4, 1.6 Hz, 1H), 7.50–7.42 (m, 2H), 7.07 (d, *J* = 19.2 Hz, 1H), 6.63 (d, *J* = 19.2 Hz, 1H), 0.22 (s, 9H); ^{13}C NMR (100 MHz, $CDCl_3$) δ 143.8, 136.0, 133.8, 133.4, 130.2, 128.3, 128.3, 127.8, 126.7, 126.3, 126.1, 123.5, -1.0. Spectroscopic data are in accordance with those described in the literature.[18]

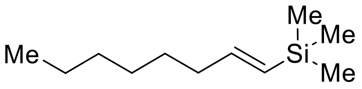

(*E*)-trimethyl(oct-1-en-1-yl)silane (*E*-3ab)

The general procedure was applied to **1a** (13 mg, 0.02 mmol, 5 mol %), trimethyl(oct-1-yn-1-yl)silane **2ab** (72 mg, 0.4 mmol), Mg (19 mg), TMSCl (25 *u*L) and THF (2 mL). The reaction mixture was stirred under H_2 (6 atm) at 80 °C for 4 h, then heated up to 100 °C for 20 h. Then the resulting crude product was purified by column chromatography on silica gel with PE to afford the title compound as a colorless liquid (58 mg, 78% yield, *E*:*Z* = 96:4). ^{1}H NMR (400 MHz, $CDCl_3$) δ 6.02 (dt, *J* = 18.4, 6.2 Hz, 1H), 5.61 (d, *J* = 18.4 Hz, 1H), 2.13-2.05 (m, 2H), 1.28 (t, *J* = 9.6 Hz, 8H), 0.88 (t, *J* = 6.8 Hz, 3H), 0.04 (s, 9H); ^{13}C NMR (100 MHz, $CDCl_3$) δ 147.6, 129.6, 36.9, 31.9, 29.0, 28.8, 22.8, 14.2, -1.0. HRMS (ESI, m/z): calcd for $C_{11}H_{24}SiNa$ $[M+Na]^+$: 207.1539, found: 207.1558.

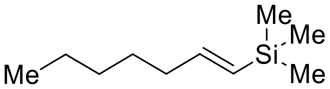

(*E*)-hept-1-en-1-yltrimethylsilane (*E*-3ac)

The general procedure was applied to **1a** (13 mg, 0.02 mmol, 5 mol %), trimethyl(oct-1-yn-1-yl)silane **2ac** (67 mg, 0.4 mmol), Mg (19 mg), TMSCl (25 *u*L) and THF (2 mL). The reaction mixture was stirred under H_2 (6 atm) at 80 °C for 4 h, then heated up to 100 °C for 20 h. Then the resulting crude product was purified by

column chromatography on silica gel with pentane to afford the title compound as a colorless liquid (50 mg, 74% yield, *E*:*Z* = 92:8). ^{1}H NMR (400 MHz, $CDCl_3$) δ 6.03 (dt, *J* = 18.4, 6.2 Hz, 1H), 5.61 (dt, *J* = 18.4, 1.5 Hz, 1H), 2.14–2.04 (m, 2H), 1.27–1.24 (m, 6H), 0.91–0.87 (t, *J* = 6.8 Hz, 3H), 0.04 (s, 9H); ^{13}C NMR (100 MHz, $CDCl_3$) δ 147.6, 129.6, 36.9, 31.6, 28.6, 22.7, 14.2, -1.0. HRMS (ESI, m/z): calcd for $C_{10}H_{23}Si$ $[M+H]^+$: 171.1564, found: 171.1568.

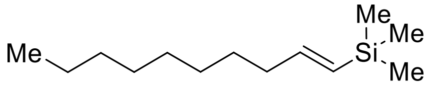

(*E*)-dec-1-en-1-yltrimethylsilane (*E*-3ad)

The general procedure was applied to **1a** (13 mg, 0.02 mmol, 5 mol %), trimethyl(oct-1-yn-1-yl)silane **2ad** (84 mg, 0.4 mmol), Mg (19 mg), TMSCl (25 *u*L) and THF (2 mL). The reaction mixture was stirred under H_2 (6 atm) at 80 °C for 4 h, then heated up to 100 °C for 20 h. Then the resulting crude product was purified by column chromatography on silica gel with PE to afford the title compound as a colorless liquid (68 mg, 81% yield, *E*:*Z* = 93:7). ^{1}H NMR (400 MHz, $CDCl_3$) δ 6.03 (dt, *J* = 18.4, 6.2 Hz, 1H), 5.61 (d, *J* = 18.4 Hz, 1H), 2.09 (dt, *J* = 7.4, 3.6 Hz, 2H), 1.28–1.26 (m, 12H), 0.88 (t, *J* = 6.4 Hz, 3H), 0.04 (s, 9H); ^{13}C NMR (100 MHz, $CDCl_3$) δ 147.6, 129.6, 36.9, 32.1, 29.9, 29.5, 29.4, 28.9, 22.9, 14.3, -1.0. HRMS (ESI, m/z): calcd for $C_{13}H_{28}SiNa$ $[M+Na]^+$: 235.1852, found: 235.1875.

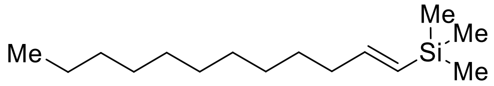

(*E*)-dodec-1-en-1-yltrimethylsilane (*E*-3ae)

The general procedure was applied to **1a** (13 mg, 0.02 mmol, 5 mol %), trimethyl(oct-1-yn-1-yl)silane **2ae** (96 mg, 0.4 mmol), Mg (19 mg), TMSCl (25 *u*L) and THF (2 mL). The reaction mixture was stirred under H_2 (6 atm) at 80 °C for 4 h, then heated up to 100 °C for 20 h. Then the resulting crude product was purified by

column chromatography on silica gel with PE to afford the title compound as a colorless liquid (80 mg, 83% yield, *E*:*Z* = 95:5). ^{1}H NMR (400 MHz, $CDCl_3$) δ 6.03 (dt, *J* = 18.4, 6.2 Hz, 1H), 5.62 (dt, *J* = 18.4, 1.4 Hz, 1H), 2.16–2.05 (m, 2H), 1.32–1.25(m, 16H), 0.89 (t, *J* = 6.8 Hz, 3H), 0.05 (s, 9H); ^{13}C NMR (100 MHz, $CDCl_3$) δ 147.6, 129.6, 37.0, 32.1, 29.8, 29.8, 29.7, 29.6, 29.4, 28.9, 22.9, 14.3, -1.0. HRMS (ESI, m/z): calcd for $C_{15}H_{33}Si$ $[M+H]^+$: 241.2346, found: 241.2347.

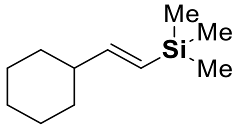

(*E*)-(2-cyclohexylvinyl)trimethylsilane (*E*-3af)

The general procedure was applied to **1a** (18 mg, 0.028 mmol, 7 mol %), trimethyl(oct-1-yn-1-yl)silane **2af** (72 mg, 0.4 mmol), Mg (19 mg), TMSCl (25 *u*L) and THF (2 mL). The reaction mixture was stirred under H_2 (6 atm) at 80 °C for 4 h, then heated up to 100 °C for 20 h. Then the resulting crude product was purified by column chromatography on silica gel with PE to afford the title compound as a colorless liquid (64 mg, 89% yield, *E*:*Z* = 97:3). ^{1}H NMR (400 MHz, $CDCl_3$) δ 5.97 (dd, *J* = 18.8, 1.4 Hz, 1H), 5.56 (dd, *J* = 18.8, 1.4 Hz, 1H), 2.00–1.88 (m, 1H), 1.77–1.69 (m, 4H), 1.68–1.60 (m, 1H), 1.30–1.27 (m, 1H), 1.23–1.02 (m, 4H), 0.04 (s, 9H); ^{13}C NMR (100 MHz, $CDCl_3$) δ 153.0, 126.3, 44.0, 32.6, 26.5, 26.3, -1.0. HRMS (ESI, m/z): calcd for $C_{11}H_{22}SiNa$ $[M+Na]^+$: 205.1383, found: 205.1355.

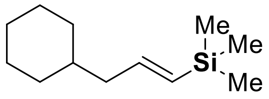

(*E*)-(3-cyclohexylprop-1-en-1-yl)trimethylsilane (*E*-3ag)

The general procedure was applied to **1a** (18 mg, 0.028 mmol, 7 mol %), trimethyl(oct-1-yn-1-yl)silane **2ag** (78 mg, 0.4 mmol), Mg (19 mg), TMSCl (25 *u*L) and THF (2 mL). The reaction mixture was stirred under H_2 (6 atm) at 80 °C for 4 h, then heated up to 100 °C for 20 h. Then the resulting crude product was purified by

column chromatography on silica gel with PE to afford the title compound as a colorless liquid (64 mg, 82% yield, *E*:*Z* = 96:4). ^{1}H NMR (400 MHz, $CDCl_3$) δ 5.99 (ddd, *J* = 18.4, 8.6, 4.8 Hz, 1H), 5.59 (dt, *J* = 18.4, 1.3 Hz, 1H), 2.00 (td, *J* = 6.8, 1.2 Hz, 2H), 1.72–1.67 (m, 4H), 1.29–1.11 (m, 5H), 0.93–0.83 (m, 2H), 0.05 (s, 9H); ^{13}C NMR (100 MHz, $CDCl_3$) δ 146.1, 131.1, 45.2, 37.7, 33.3, 26.8, 26.6, -0.9. HRMS (ESI, m/z): calcd for $C_{12}H_{24}SiK$ $[M+K]^+$: 235.1279, found: 235.1280.

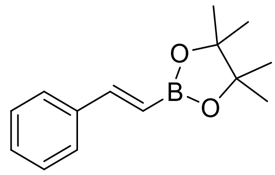

(*E*)-4,4,5,5-tetramethyl-2-styryl-1,3,2-dioxaborolane (*E*-3ah)

The general procedure was applied to **1a** (4 mg, 0.006 mmol, 3 mol %), 4,4,5,5-tetramethyl-2-(phenylethynyl)-1,3,2-dioxaborolane **2ah** (46 mg, 0.2 mmol), Mg (10 mg), TMSCl (13 *u*L) and THF (2 mL). The reaction mixture was stirred under H_2 (10 atm) at 100 °C for 24 h. Then the resulting crude product was purified by column chromatography on silica gel with (PE:EA = 50:1) to afford the title compound as a colorless liquid (36 mg, 78% yield, *E*:*Z* = 97:3). ^{1}H NMR (400 MHz, $CDCl_3$) δ 7.54–7.50 (m, 2H), 7.45 (d, *J* = 18.4 Hz, 1H), 7.39–7.30 (m, 3H), 6.22 (d, *J* = 18.4 Hz, 1H), 1.35 (s, 12H); ^{13}C NMR (100 MHz, $CDCl_3$) δ 149.6, 137.5, 129.0, 128.6, 127.1, 83.4, 24.9; ^{11}B NMR (128 MHz, $CDCl_3$) δ 30.7. Spectroscopic data are in accordance with those described in the literature.[19]

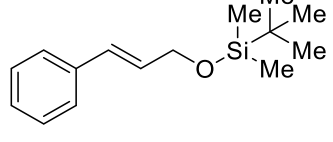

***tert*-butyl(cinnamyloxy)dimethylsilane (*E*-3ai)**

The general procedure was applied to **1a** (4 mg, 0.006 mmol, 3 mol %), tert-butyldimethyl((3-phenylprop-2-yn-1-yl)oxy)silane **2ai** (49 mg, 0.2 mmol), Mg (10 mg), TMSCl (13 *u*L) and THF (2 mL). The reaction mixture was stirred under H_2

(10 atm) at 100 °C for 24 h. Then the resulting crude product was purified by column chromatography on silica gel with (PE:EA = 50:1) to afford the title compound as a colorless liquid (40 mg, 78% yield, *E*:*Z* = 99:1). ^{1}H NMR (400 MHz, $CDCl_3$) δ 7.43 (d, *J* = 7.4 Hz, 2H), 7.36 (t, *J* = 7.6 Hz, 2H), 7.27 (t, *J* = 7.2 Hz, 1H), 6.65 (d, *J* = 15.8 Hz, 1H), 6.34 (dt, *J* = 15.8, 5.0 Hz, 1H), 4.41 (dd, *J* = 5.0, 1.6 Hz, 2H), 1.01 (s, 9H), 0.17 (s, 6H); ^{13}C NMR (100 MHz, $CDCl_3$) δ 137.3, 129.6, 129.3, 128.6, 127.4, 126.5, 64.0, 26.1, 18.6, -5.0. Spectroscopic data are in accordance with those described in the literature.[20]

6. Supplementary General Procedure of CAAC-Imino-Cr-Catalyzed *Cis*-Selective Hydrogenation of Alkynes for the Synthesis of *Z*-Olefins

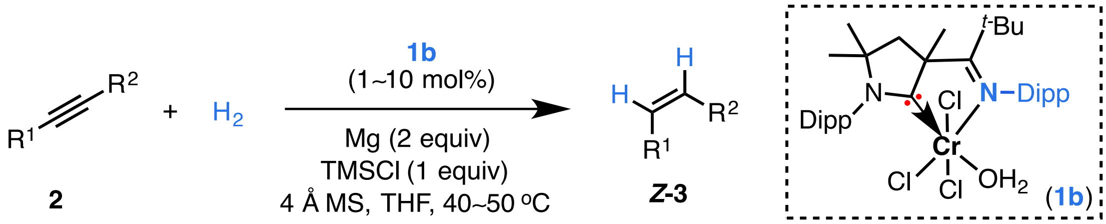

In a Schlenk tube were placed **1b** (1~10 mol %), alkyne **2** (0.2 mmol), Mg (10 mg), 4 Å MS (25 mg), TMSCl (25 *u*L) and THF (2 mL) under atmosphere of nitrogen. The tube was quickly moved to a high–pressure autoclave, and stirred under atmosphere of H_2 (40~50 atm) at 40~50 °C for 24 h. After quenching with HCl_{aq} (2 mL, 1 M), the crude product was extracted with ethyl acetate (3 × 4 mL). The combined organic phases were dried over anhydrous Na_2SO_4 and concentrated under vacuum. The stereoselectivity of the *Z*-olefin relative to *E*-stereomer was determined by GC analysis or ^{1}H NMR prior to purification. The crude product was purified by silica gel chromatography to afford the desired olefin compound.

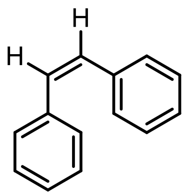

(*Z*)-1,2-diphenylethene (*Z*-3a)

The general procedure was applied to **1b** (7 mg, 0.01 mmol, 5 mol %), 1,2-diphenylethyne **2a** (36 mg, 0.2 mmol), Mg (10 mg), TMSCl (25 *u*L), 4 Å MS (25 mg) and THF (2 mL). The reaction mixture was stirred under H_2 (50 atm) at 40 °C for 24 h. Then the resulting crude product was purified by column chromatography on silica gel with PE to afford the title compound as a colorless oil (31 mg, 86% yield, *Z*/*E* = 93:7). ^{1}H NMR (400 MHz, $CDCl_3$): δ 7.19 (m, 10H), 6.58 (s, 2H); ^{13}C NMR (100 MHz, $CDCl_3$): δ 137.4, 130.4, 129.0, 128.3, 127.2. Spectroscopic data are in accordance with those described in the literature.[5]

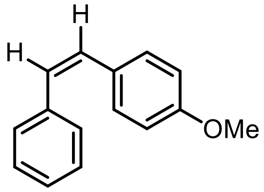

(*Z*)-1-methoxy-4-styrylbenzene (*Z*-3j)

The general procedure was applied to **1b** (7 mg, 0.01 mmol, 5 mol %), 1-methoxy-4-(phenylethynyl)benzene **2j** (42 mg, 0.2 mmol), Mg (10 mg), TMSCl (25 *u*L), 4 Å MS (25 mg) and THF (2 mL). The reaction mixture was stirred under H_2 (40 atm) at 40 °C for 24 h. Then the resulting crude product was purified by column chromatography on silica gel with (PE:EA = 100:1) to afford the title compound as a colorless oil (39 mg, 92% yield, *Z*/*E* = 96:4). ^{1}H NMR (400 MHz, $CDCl_3$): δ 7.29–7.15 (m, 7H), 6.79–6.70 (m, 2H), 6.54 (d, J = 12.0 Hz, 2H), 6.50 (d, J = 12.4 Hz, 2H), 3.78 (s, 3H); ^{13}C NMR (100 MHz, $CDCl_3$): δ 158.8, 137.8, 130.3, 129.9, 129.8, 129.0, 128.9, 128.4, 127.0, 113.7, 55.3. Spectroscopic data are in accordance with those described in the literature.[5]

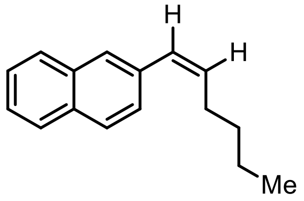

(*Z*)-2-(hex-1-en-1-yl)naphthalene (*Z*-3b)

The general procedure was applied to **1b** (6 mg, 0.008 mmol, 4 mol %), 2-(hex-1-yn-1-yl)naphthalene **2b** (42 mg, 0.2 mmol), Mg (10 mg), TMSCl (25 *u*L), 4 Å MS (25 mg) and THF (2 mL). The reaction mixture was stirred under H_2 (50 atm) at 40 °C for 24 h. Then the resulting crude product was purified by column chromatography on silica gel with (PE:EA = 100:1) to afford the title compound as a colorless oil (38 mg, 90% yield, *Z*/*E* = 98:2). ^{1}H NMR (400 MHz, $CDCl_3$): δ 7.85–7.75 (m, 3H), 7.72 (s, 1H), 7.51–7.38 (m, 3H), 6.56 (d, *J* = 11.6 Hz, 1H), 5.76 (dt, *J* = 11.6, 7.2 Hz, 1H), 2.5–2.32 (m, 2H), 1.53–1.33 (m, 4H), 0.91 (t, *J* = 7.2 Hz, 3H); ^{13}C NMR (100 MHz, $CDCl_3$): δ 135.5, 133.9, 133.5, 132.3, 128.9, 128.1, 127.7, 127.7, 127.5, 127.4, 126.1, 125.8, 32.3, 28.6, 22.6, 14.1. HRMS (ESI, m/z): calcd for $C_{16}H_{19}$ $[M+H]^+$: 211.1481, found: 211.1480.

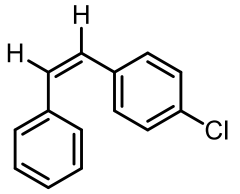

(*Z*)-1-chloro-4-styrylbenzene (*Z*-3aj)

The general procedure was applied to **1b** (11 mg, 0.016 mmol, 8 mol %), 1-chloro-4-(phenylethynyl)benzene **2aj** (42 mg, 0.2 mmol), Mg (10 mg), TMSCl (25 *u*L), 4 Å MS (25 mg) and THF (2 mL). The reaction mixture was stirred under H_2 (50 atm) at 40 °C for 24 h. Then the resulting crude product was purified by column chromatography on silica gel with (PE:EA = 100:1) to afford the title compound as a colorless oil (37 mg, 86% yield, *Z*/*E* = 97:3). ^{1}H NMR (400 MHz, $CDCl_3$): δ 7.27–7.11 (m, 9H), 6.62 (dd, *J* = 12.4, 1.2 Hz, 1H), 6.52 (d, *J* = 12.4 Hz, 1H); ^{13}C

NMR (100 MHz, $CDCl_3$): δ 137.0, 135.8, 132.9, 131.1, 130.4, 129.1, 128.9, 128.5, 128.5, 127.5. Spectroscopic data are in accordance with those described in the literature.[5]

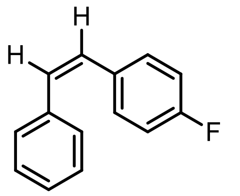

(*Z*)-1-fluoro-4-styrylbenzene (*Z*-3ak)

The general procedure was applied to **1b** (14 mg, 0.02 mmol, 10 mol %), 1-fluoro-4-(phenylethynyl)benzene **2ak** (39 mg, 0.2 mmol), Mg (10 mg), TMSCl (25 *u*L), 4 Å MS (25 mg) and THF (2 mL). The reaction mixture was stirred under H_2 (50 atm) at 40 °C for 24 h. Then the resulting crude product was purified by column chromatography on silica gel with (PE:EA = 100:1) to afford the title compound as a colorless oil (33 mg, 83% yield, *Z*/*E* = 91:9). ^{1}H NMR (400 MHz, $CDCl_3$): δ 7.31–7.13 (m, 7H), 6.97–6.82 (m, 2H), 6.59 (d, *J* = 12.0 Hz, 1H), 6.53 (d, *J* = 12.0 Hz, 1H); ^{13}C NMR (100 MHz, $CDCl_3$): δ 162.0 (d, *J* = 245.1 Hz), 137.2, 133.3 (d, *J* = 3.3 Hz), 130.7 (d, *J* = 7.8 Hz), 130.4, 129.2, 129.0, 128.4, 127.3, 115.3 (d, *J* = 21.3 Hz); ^{19}F NMR (376 MHz, $CDCl_3$): δ –114.6. Spectroscopic data are in accordance with those described in the literature.[5]

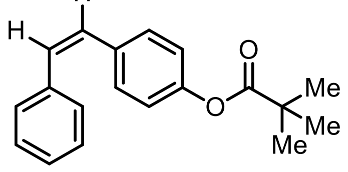

(*Z*)-4-styrylphenyl pivalate (*Z*-3al)

The general procedure was applied to **1b** (14 mg, 0.02 mmol, 10 mol %), 4-(phenylethynyl)phenyl pivalate **2al** (56 mg, 0.2 mmol), Mg (10 mg), TMSCl (25 *u*L), 4 Å MS (25 mg) and THF (2 mL). The reaction mixture was stirred under H_2 (50 atm) at 40 °C for 24 h. Then the resulting crude product was purified by column

chromatography on silica gel with (PE:EA = 50:1) to afford the title compound as a colorless oil (52 mg, 92% yield, *Z*/*E* = 97:3). ^{1}H NMR (400 MHz, $CDCl_3$): δ 7.28-7.13 (m, 7H), 6.94-6.85 (m, 2H), 6.60 (d, *J* = 12.4 Hz, 1H), 6.55 (d, *J* = 12.4 Hz, 1H), 1.34 (s, 9H); ^{13}C NMR (100 MHz, $CDCl_3$): δ 177.2, 150.1, 137.2, 134.7, 130.5, 123.0, 129.4, 129.0, 128.4, 127.3, 121.4, 39.2, 27.3. Spectroscopic data are in accordance with those described in the literature.[21]

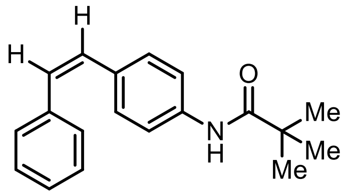

(*Z*)-*N*-(4-styrylphenyl)pivalamide (*Z*-3am)

The general procedure was applied to **1b** (14 mg, 0.02 mmol, 10 mol %), *N*-(4-(phenylethynyl)phenyl)pivalamide **2am** (55 mg, 0.2 mmol), Mg (10 mg), TMSCl (25 *u*L), 4 Å MS (25 mg) and THF (2 mL). The reaction mixture was stirred under H_2 (50 atm) at 40 °C for 24 h. Then the resulting crude product was purified by column chromatography on silica gel with (PE:EA = 10:1) to afford the title compound as a yellow oil (49 mg, 87% yield, *Z*/*E* = 99:1). ^{1}H NMR (400 MHz, $CDCl_3$): δ 7.41–7.36 (m, 2H), 7.29 (s, 1H), 7.27–7.17 (m, 7H), 6.56 (d, *J* = 12.4 Hz, 1H), 6.53 (d, *J* = 12.4 Hz, 1H), 1.30 (s, 9H); ^{13}C NMR (100 MHz, $CDCl_3$): δ 176.6, 137.4, 137.0, 133.3, 129.9, 129.7, 129.7, 129.0, 128.4, 127.2, 119.7, 39.8, 27.8. HRMS (ESI, m/z): calcd for $C_{19}H_{22}NO$ $[M+H]^+$: 280.1696, found: 280.1689.

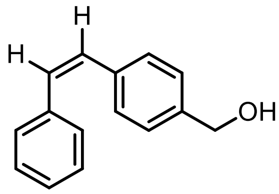

(*Z*)-(4-styrylphenyl)methanol (*Z*-3an)

The general procedure was applied to **1b** (7 mg, 0.01 mmol, 5 mol %), (4-(phenylethynyl)phenyl)methanol **2an** (42 mg, 0.2 mmol), Mg (10 mg), TMSCl (25

*u*L), 4 Å MS (25 mg) and THF (2 mL). The reaction mixture was stirred under H_2 (60 atm) at 50 °C for 24 h. Then the resulting crude product was purified by column chromatography on silica gel with (PE:EA = 5:1) to afford the title compound as a colorless oil (30 mg, 72% yield, *Z*/*E* = 99:1). ^{1}H NMR (400 MHz, $CDCl_3$): δ 7.21 (m, 9H), 6.60 (d, *J* = 12.4 Hz, 1H), 6.57 (d, *J* = 12.4 Hz, 1H), 4.63 (s, 2H), 1.80 (s, 1H); ^{13}C NMR (100 MHz, $CDCl_3$): δ 139.8, 137.3, 136.8, 130.5, 126.0, 129.2, 129.0, 128.4, 127.3, 127.0, 65.2. Spectroscopic data are in accordance with those described in the literature.[22]

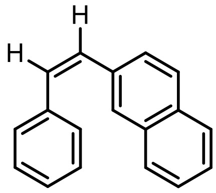

(*Z*)-2-styrylnaphthalene (*Z*-3ao)

The general procedure was applied to **1b** (7 mg, 0.01 mmol, 5 mol %), 2-(phenylethynyl)naphthalene **2ao** (46 mg, 0.2 mmol), Mg (10 mg), TMSCl (25 *u*L), 4 Å MS (25 mg) and THF (2 mL). The reaction mixture was stirred under H_2 (50 atm) at 40 °C for 24 h. Then the resulting crude product was purified by column chromatography on silica gel with PE to afford the title compound as a colorless oil (35 mg, 75% yield, *Z*/*E* > 99:1). ^{1}H NMR (400 MHz, $CDCl_3$): δ 7.83–7.69 (m, 3H), 7.65 (d, *J* = 8.4 Hz, 1H), 7.47–7.40 (m, 2H), 7.35 (dd, *J* = 8.4, 1.6 Hz, 1H), 7.31–7.27 (m, 2H), 7.25–7.18 (m, 3H), 6.77 (d, *J* = 12.4, 1H), 6.69 (d, *J* = 12.4, 1H); ^{13}C NMR (100 MHz, $CDCl_3$): δ 137.4, 135.0, 133.6, 132.7, 130.8, 130.3, 129.1, 128.4, 128.1, 128.1, 127.8, 127.6, 127.4, 127.1, 126.1, 126.0. Spectroscopic data are in accordance with those described in the literature.[8]

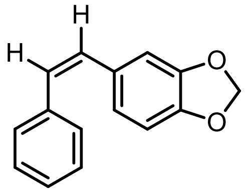

(*Z*)-5-styrylbenzo[*d*][1,3]dioxole (*Z*-3ap)

The general procedure was applied to **1b** (8.3 mg, 0.012 mmol, 6 mol %), 5-(phenylethynyl)benzo[*d*][1,3]dioxole **2ap** (44 mg, 0.2 mmol), Mg (10 mg), TMSCl (25 *u*L), 4 Å MS (25 mg) and THF (2 mL). The reaction mixture was stirred under H_2 (50 atm) at 40 °C for 24 h. Then the resulting crude product was purified by column chromatography on silica gel with PE to afford the title compound as a colorless oil (39 mg, 88% yield, *Z*/*E* = 94:6). ^1H NMR (400 MHz, $CDCl_3$): δ 7.32–7.14 (m, 5H), 6.78–6.65 (m, 3H), 6.52 (d, *J* = 12.0 Hz, 1H), 6.48(d, *J* = 12.4 Hz, 1H), 5.90 (s, 2H); ^{13}C NMR (100 MHz, $CDCl_3$): δ 147.5, 146.8, 137.4, 131.3, 129.9, 129.4, 129.0, 128.4, 127.2, 123.1, 109.1, 108.3, 101.0. Spectroscopic data are in accordance with those described in the literature.[23]

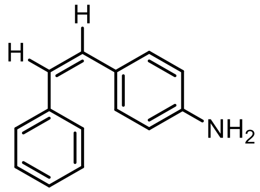

(*Z*)-4-styrylaniline (*Z*-3i)

The general procedure was applied to **1b** (7 mg, 0.01 mmol, 5 mol %), 4-(phenylethynyl)aniline **2i** (39 mg, 0.2 mmol), Mg (10 mg), TMSCl (25 *u*L), 4 Å MS (25 mg) and THF (2 mL). The reaction mixture was stirred under H_2 (50 atm) at 40 °C for 24 h. Then the resulting crude product was purified by column chromatography on silica gel with (PE:EA = 10:1) to afford the title compound as a yellow oil (34 mg, 86% yield, *Z*/*E* = 96:4). ^1H NMR (400 MHz, $CDCl_3$): δ 7.48 (d, *J* = 7.2 Hz, 2H), 7.34 (t, *J* = 8.0 Hz, 4H), 7.21 (t, *J* = 7.4 Hz, 1H), 7.08–6.87 (m, 2H), 6.68 (d, *J* = 8.4 Hz, 2H), 3.74 (s, 2H); ^{13}C NMR (100 MHz, $CDCl_3$): δ 146.3, 138.1, 128.8, 128.7, 128.2, 127.9, 127.0, 126.2, 125.3, 115.4. Spectroscopic data are in accordance with those described in the literature.[5]

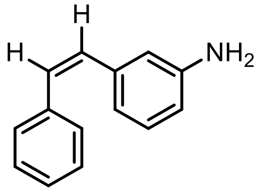

(*Z*)-3-styrylaniline (*Z*-3aq)

The general procedure was applied to **1b** (7 mg, 0.01 mmol, 5 mol %), 3-(phenylethynyl)aniline **2aq** (39 mg, 0.2 mmol), Mg (10 mg), TMSCl (25 *u*L), 4 Å MS (25 mg) and THF (2 mL). The reaction mixture was stirred under H_2 (50 atm) at 40 °C for 24 h. Then the resulting crude product was purified by column chromatography on silica gel with (PE:EA = 10:1) to afford the title compound as a yellow oil (34 mg, 88% yield, *Z*/*E* > 99:1). ^{1}H NMR (400 MHz, $CDCl_3$): δ 7.31–7.13 (m, 5H), 7.02 (t, *J* = 8.0 Hz, 1H), 6.65 (d, *J* = 7.6 Hz, 1H), 6.60–6.55 (m, 1H), 6.55–6.48 (m, 3H), 3.53 (s, 3H); ^{13}C NMR (100 MHz, $CDCl_3$): δ 146.3, 138.5, 137.5, 130.6, 130.2, 129.3, 129.1, 128.3, 127.2, 119.6, 115.5, 114.2. HRMS (ESI, m/z): calcd for $C_{14}H_{14}N$ $[M+H]^+$: 196.1121, found: 196.1120.

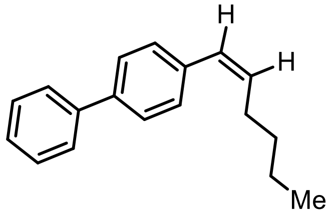

(*Z*)-4-(hex-1-en-1-yl)-1,1'-biphenyl (*Z*-3x)

The general procedure was applied to **1b** (6 mg, 0.008 mmol, 4 mol %), 4-(hex-1-yn-1-yl)-1,1'-biphenyl **2x** (47 mg, 0.2 mmol), Mg (10 mg), TMSCl (25 *u*L), 4 Å MS (25 mg) and THF (2 mL). The reaction mixture was stirred under H_2 (50 atm) at 40 °C for 24 h. Then the resulting crude product was purified by column chromatography on silica gel with (PE:EA = 100:1) to afford the title compound as a colorless oil (46 mg, 97% yield, *Z*/*E* = 92:8). ^{1}H NMR (400 MHz, $CDCl_3$): δ 7.65–7.55 (m, 4H), 7.48–7.41 (m, 2H), 7.39–7.31 (m, 3H), 6.44 (d, *J* = 11.6 Hz, 1H), 5.71 (dt, *J* = 11.6, 7.2 Hz, 1H), 2.39 (qd, *J* = 7.4, 1.6 Hz, 2H), 1.52–1.33 (m, 4H), 0.92

(t, J = 7.2 Hz, 3H); ^{13}C NMR (100 MHz, $CDCl_3$): δ 141.0, 139.3, 139.1, 137.0, 136.4, 133.7, 129.3, 128.9, 128.4, 127.3, 127.1, 127.0, 32.3, 28.7, 22.6, 14.1. Spectroscopic data are in accordance with those described in the literature.[24]

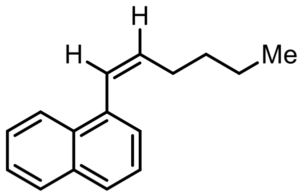

(*Z*)-1-(hex-1-en-1-yl)naphthalene (*Z*-3ar)

The general procedure was applied to **1b** (6 mg, 0.008 mmol, 4 mol %), 1-(hex-1-yn-1-yl)naphthalene **2ar** (42 mg, 0.2 mmol), Mg (10 mg), TMSCl (25 *u*L), 4 Å MS (25 mg) and THF (2 mL). The reaction mixture was stirred under H_2 (50 atma) at 40 °C for 24 h. Then the resulting crude product was purified by column chromatography on silica gel with (PE:EA = 100:1) to afford the title compound as a colorless oil (37 mg, 89% yield, Z/E = 96:4). ^{1}H NMR (400 MHz, $CDCl_3$): δ 8.07–7.98 (m, 1H), 7.90–7.83 (m, 1H), 7.78 (d, J = 8.2 Hz, 1H), 7.54–7.43 (m, 3H), 7.36 (d, J = 7.2 Hz, 1H), 6.88 (d, J = 11.2 Hz, 1H), 5.96 (dt, J = 11.6, 7.2 Hz, 1H), 2.18 (qd, J = 7.4, 1.6 Hz, 2H), 1.45–1.36 (m, 2H), 1.35–1.24 (m, 2H), 0.84 (t, J = 7.2 Hz, 3H); ^{13}C NMR (100 MHz, $CDCl_3$): δ 135.1, 134.8, 133.7, 132.1, 128.4, 127.2, 126.9, 126.5, 125.9, 125.8, 125.4, 125.2, 32.1, 28.6, 22.5, 14.1. Spectroscopic data are in accordance with those described in the literature.[25]

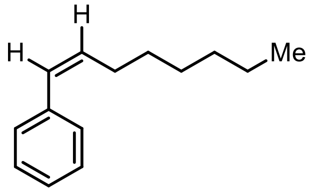

(*Z*)-oct-1-en-1-ylbenzene (*Z*-3as)

The general procedure was applied to **1b** (1 mg, 0.002 mmol, 1 mol %), oct-1-yn-1-ylbenzene **2as** (37 mg, 0.2 mmol), Mg (10 mg), TMSCl (25 *u*L), 4 Å MS (25 mg) and THF (2 mL). The reaction mixture was stirred under H_2 (40 atm) at 40 °C

for 24 h. Then the resulting crude product was purified by column chromatography on silica gel with (PE:EA = 100:1) to afford the title compound as a colorless oil (35 mg, 92% yield, *Z*/*E* = 90:10). ^{1}H NMR (400 MHz, $CDCl_3$): δ 7.34–7.24 (m, 4H), 7.22–7.16 (m, 1H), 6.40 (d, *J* = 11.6 Hz, 1H), 5.66 (dt, *J* = 11.6, 7.2 Hz, 1H), 2.32 (qd, *J* = 7.4, 1.6 Hz, 2H), 1.48–1.40 (m, 2H), 1.33–1.24 (m, 6H), 0.87 (t, *J* = 7.2 Hz, 3H). ^{13}C NMR (100 MHz, $CDCl_3$): δ 138.0, 133.4, 128.9, 128.8, 128.2, 126.5, 31.9, 30.1, 29.2, 28.8, 22.8, 14.2. Spectroscopic data are in accordance with those described in the literature.[26]

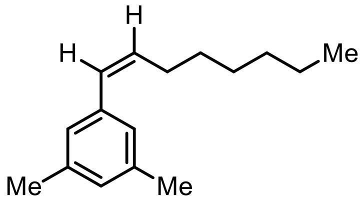

(*Z*)-1,3-dimethyl-5-(oct-1-en-1-yl)benzene (*Z*-3u)

The general procedure was applied to **1b** (3 mg, 0.004 mmol, 2 mol %), 1,3-dimethyl-5-(oct-1-yn-1-yl)benzene **2u** (43 mg, 0.2 mmol), Mg (10 mg), TMSCl (25 *u*L), 4 Å MS (25 mg) and THF (2 mL). The reaction mixture was stirred under H_2 (40 atm) at 40 °C for 24 h. Then the resulting crude product was purified by column chromatography on silica gel with (PE:EA = 100:1) to afford the title compound as a colorless oil (44 mg, 99% yield, *Z*/*E* = 90:10). ^{1}H NMR (400 MHz, $CDCl_3$): δ 6.91 (s, 2H), 6.87 (s, 1H), 6.35 (d, *J* = 11.6 Hz, 1H), 5.63 (dt, *J* = 11.6, 7.2 Hz, 1H), 2.37–2.30 (m, 8H), 1.50–1.41 (m, 2H), 1.37–1.26 (m, 6H), 0.89 (t, *J* = 6.8 Hz, 3H). ^{13}C NMR (100 MHz, $CDCl_3$): δ 137.9, 137.6, 133.1, 128.9, 128.2, 126.7, 31.9, 30.1, 29.2, 28.9, 22.8, 21.5, 14.2. HRMS (ESI, m/z): calcd for $C_{16}H_{25}$ $[M+H]^+$: 217.1951, found: 217.1953.

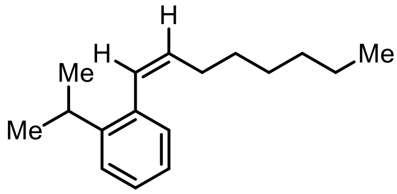

(*Z*)-1-isopropyl-2-(oct-1-en-1-yl)benzene (*Z*-3at)

The general procedure was applied to **1b** (6 mg, 0.008 mmol, 4 mol %), 1-isopropyl-2-(oct-1-yn-1-yl)benzene **2at** (46 mg, 0.2 mmol), Mg (10 mg), TMSCl (25 *u*L), 4 Å MS (25 mg) and THF (2 mL). The reaction mixture was stirred under H_2 (40 atm) at 40 °C for 24 h. Then the resulting crude product was purified by column chromatography on silica gel with (PE:EA = 100:1) to afford the title compound as a colorless oil (44 mg, 95% yield, *Z*/*E* > 99:1). ^{1}H NMR (400 MHz, $CDCl_3$): δ 7.29–7.26(m, 1H), 7.24–7.18 (m, 1H), 7.16–7.08 (m, 2H), 6.53 (d, *J* = 11.6 Hz, 1H), 5.82–5.60 (m, 1H), 3.21–3.06 (m, 1H), 2.18–1.96 (m, 2H), 1.41–1.33 (m, 2H), 1.27–1.22 (m, 6H), 1.20 (s, 3H), 1.18 (s, 3H), 0.85 (t, *J* = 7.2 Hz, 3H); ^{13}C NMR (100 MHz, $CDCl_3$): δ 146.9, 136.0, 133.4, 129.7, 128.0, 127.2, 125.2, 124.8, 31.9, 29.9, 29.9, 29.1, 28.5, 23.4, 22.8, 14.2. HRMS (ESI, m/z): calcd for $C_{17}H_{27}$ $[M+H]^+$: 231.2107, found: 231.2111.

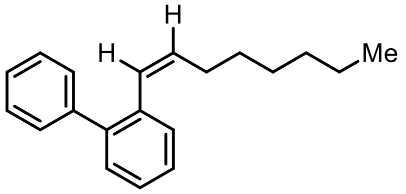

(*Z*)-2-(oct-1-en-1-yl)-1,1'-biphenyl (*Z*-3au)

The general procedure was applied to **1b** (6 mg, 0.008 mmol, 4 mol %), 2-(oct-1-yn-1-yl)-1,1'-biphenyl **2au** (52 mg, 0.2 mmol), Mg (10 mg), TMSCl (25 *u*L), 4 Å MS (25 mg) and THF (2 mL). The reaction mixture was stirred under H_2 (50 atm) at 40 °C for 24 h. Then the resulting crude product was purified by column chromatography on silica gel with (PE:EA = 100:1) to afford the title compound as a colorless oil (45 mg, 85% yield, *Z*/*E* = 98:2). ^{1}H NMR (400 MHz, $CDCl_3$): δ 7.40–7.35 (m, 5H), 7.35–7.29 (m, 4H), 6.29–6.21 (m, 1H), 5.65–5.54 (m, 1H), 2.25 (qd, *J* = 7.4, 1.6 Hz, 2H), 1.44–1.36 (m, 2H), 1.34–1.25 (m, 7H), 0.89 (t, *J* = 7.0 Hz, 3H); ^{13}C NMR (100 MHz, $CDCl_3$): δ 141.5, 141.3, 135.9, 133.0, 129.9, 129.9, 128.8, 128.0, 127.0, 126.9, 31.9, 29.9, 29.3, 28.7, 22.8, 14.2. HRMS (ESI, m/z): calcd for

$C_{20}H_{25}$ [M+H]$^+$: 265.1951, found: 265.1955.

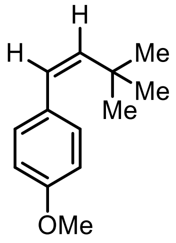

(*Z*)-1-(3,3-dimethylbut-1-en-1-yl)-4-methoxybenzene (*Z*-3av)

The general procedure was applied to **1b** (8 mg, 0.012 mmol, 6 mol %), 1-(3,3-dimethylbut-1-yn-1-yl)-4-methoxybenzene **2av** (38 mg, 0.2 mmol), Mg (10 mg), TMSCl (25 *u*L), 4 Å MS (25 mg) and THF (2 mL). The reaction mixture was stirred under H_2 (50 atm) at 40 °C for 24 h. Then the resulting crude product was purified by column chromatography on silica gel with (PE:EA = 100:1) to afford the title compound as a colorless oil (34 mg, 90% yield, *Z*/*E* = 94:6). ^{1}H NMR (400 MHz, $CDCl_3$): δ 7.10 (d, *J* = 8.6 Hz, 2H), 6.85–6.79 (m, 2H), 6.35 (d, *J* = 12.4 Hz, 1H), 5.56 (d, *J* = 12.8 Hz, 1H), 3.80 (s, 3H), 0.99 (s, 9H); ^{13}C NMR (100 MHz, $CDCl_3$): δ 158.2, 142.7, 131.8, 130.2, 126.9, 113.1, 55.3, 34.2, 31.4. HRMS (ESI, m/z): calcd for $C_{13}H_{19}O$ [M+H]$^+$: 191.1430, found: 191.1431.

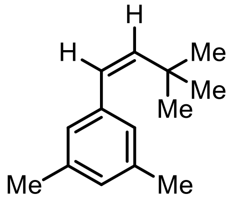

(*Z*)-1-(3,3-dimethylbut-1-en-1-yl)-3,5-dimethylbenzene (*Z*-3aw)

The general procedure was applied to **1b** (11 mg, 0.016 mmol, 8 mol %), 1-(3,3-dimethylbut-1-yn-1-yl)-3,5-dimethylbenzene **2aw** (37 mg, 0.2 mmol), Mg (10 mg), TMSCl (25 *u*L), 4 Å MS (25 mg) and THF (2 mL). The reaction mixture was stirred under H_2 (50 atm) at 40 °C for 24 h. Then the resulting crude product was purified by column chromatography on silica gel with (PE:EA = 100:1) to afford the title compound as a colorless oil (32 mg, 84% yield, *Z*/*E* = 86:14). ^{1}H NMR (400

MHz, $CDCl_3$): δ 6.85 (s, 1H), 6.81 (s, 2H), δ 6.36 (d, J = 12.4 Hz, 1H), 5.56 (d, J = 12.8 Hz, 1H), 2.30 (s, 6H), 1.00 (s, 9H); ^{13}C NMR (100 MHz, $CDCl_3$): δ 142.34, 139.40, 137.04, 127.84, 127.42, 126.89, 34.32, 31.40, 21.44. HRMS (ESI, m/z): calcd for $C_{14}H_{21}$ +H]$^+$: 189.1638, found: 189.1640.

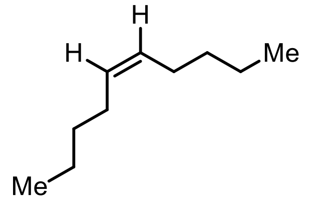

(*Z*)-dec-5-ene (*Z*-3ax)

The general procedure was applied to **1b** (1 mg, 0.002 mmol, 1 mol %), dec-5-yne **2ax** (28 mg, 0.2 mmol), Mg (10 mg), TMSCl (25 *u*L), 4 Å MS (25 mg) and THF (2 mL). The reaction mixture was stirred under H_2 (40 atm) at 40 °C for 24 h. Then the resulting crude product was purified by column chromatography on silica gel with (PE:EA = 100:1) to afford the title compound as a colorless oil (23 mg, 83% yield, *Z*/*E* > 99:1). ^1H NMR (400 MHz, $CDCl_3$): δ 5.42–5.28 (m, 2H), 2.08–1.96 (m, 4H), 1.36 1.29 (m, 5H), 0.90 (t, J = 7.2 Hz, 6H); ^{13}C NMR (100 MHz, $CDCl_3$): δ 130.0, 32.1, 27.1, 22.5, 14.1. Spectroscopic data are in accordance with those described in the literature.[27]

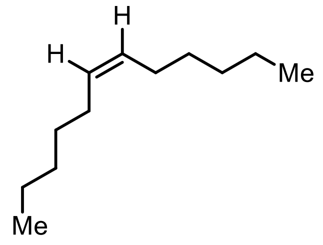

(*Z*)-dodec-6-ene (*Z*-3ay)

The general procedure was applied to **1b** (1 mg, 0.002 mmol, 1 mol %), dodec-6-yne **2ay** (33 mg, 0.2 mmol), Mg (10 mg), TMSCl (25 *u*L), 4 Å MS (25 mg) and THF (2 mL). The reaction mixture was stirred under H_2 (40 atm) at 40 °C for 24 h. Then the resulting crude product was purified by column chromatography on silica gel with

(PE:EA = 100:1) to afford the title compound as a colorless oil (30 mg, 88% yield, *Z*/*E* = 99:1). ^{1}H NMR (400 MHz, $CDCl_3$): δ 5.48–5.23 (m, 2H), 2.10–1.91 (m, 4H), 1.38–1.24 (m, 12H), 0.89 (t, *J* = 6.9 Hz, 6H); ^{13}C NMR (100 MHz, $CDCl_3$): δ 130.1, 31.7, 29.6, 27.3, 22.8, 14.2. Spectroscopic data are in accordance with those described in the literature.[12]

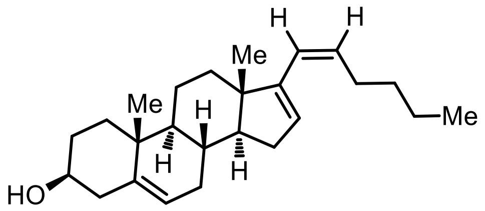

(3*S*,8*R*,9*S*,10*R*,13*S*,14*S*)-17-((*Z*)-hex-1-en-1-yl)-10,13-dimethyl-2,3,4,7,8,9,10,11,12,13,14,15-dodecahydro-1*H*-cyclopenta[*a*]phenanthren-3-ol (*Z*-3az)

The general procedure was applied to **1b** (7 mg, 0.01 mmol, 5 mol %), (3*S*,8*R*,9*S*,10*R*,13*S*,14*S*)-17-(hex-1-yn-1-yl)-10,13-dimethyl-2,3,4,7,8,9,10,11,12,13,14,15-dodecahydro-1*H*-cyclopenta[*a*]phenanthren-3-ol **2az** (70 mg, 0.2 mmol), Mg (10 mg), TMSCl (50 *u*L), 4 Å MS (25 mg) and THF (2 mL). The reaction mixture was stirred under H_2 (50 atm) at 40 °C for 24 h. Then the resulting crude product was purified by column chromatography on silica gel with (PE:EA = 10:1) to afford the title compound as a white solid (69 mg, 97% yield, *Z*/*E* = 99:1). M.p. = 98–110 °C. ^{1}H NMR (400 MHz, $CDCl_3$): δ 5.72 (d, *J* = 11.6 Hz, 1H), 5.65–5.49 (m, 2H), 5.40–5.32 (m, 1H), 3.61–3.45 (m, 1H), 2.33–2.17 (m, 4H), 2.08–1.93 (m, 2H), 1.89–1.81 (m, 2H), 1.76–1.59 (m, 5H), 1.57–1.45 (m, 2H), 1.42–1.25 (m, 7H), 1.15–0.98 (m, 5H), 0.92–0.87 (m, 3H), 0.80 (s, 3H); ^{13}C NMR (100 MHz, $CDCl_3$): δ 151.1, 141.2, 134.3, 126.5, 121.9, 121.7, 71.9, 56.3, 50.9, 47.0, 42.5, 37.3, 36.9, 34.8, 32.2, 32.1, 31.8, 30.7, 29.2, 22.6, 21.0, 19.5, 16.1, 14.1. HRMS (ESI, m/z): calcd for $C_{25}H_{39}O$ $[M+H]^+$: 355.2995, found: 355.2986.

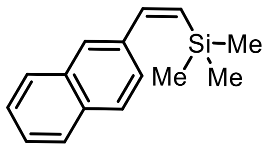

(*Z*)-trimethyl(2-(naphthalen-2-yl)vinyl)silane (Z-3ba)

The general procedure was applied to **1b** (7 mg, 0.01 mmol, 5 mol %), trimethyl(naphthalen-2-ylethynyl)silane **2ba** (45 mg, 0.2 mmol), Mg (10 mg), TMSCl (25 *u*L), 4 Å MS (25 mg) and THF (2 mL). The reaction mixture was stirred under H_2 (50 atm) at 25 °C for 24 h. Then the resulting crude product was purified by column chromatography on silica gel with (PE:EA = 50:1) to afford the title compound as a colorless liquid (39 mg, 86% yield, *Z*:*E* = 95:5). ^{1}H NMR (400 MHz, $CDCl_3$) δ 7.90–7.75 (m, 4H), 7.60–7.44 (m, 4H), 6.01–5.95 (m, 1H), 0.15 (d, J = 1.2 Hz, 9H); ^{13}C NMR (100 MHz, $CDCl_3$) δ 146.7, 137.7, 133.5, 133.3, 132.8, 128.1, 127.8, 127.6, 127.2, 126.5, 126.3, 126.0, 0.5. Spectroscopic data are in accordance with those described in the literature.[28]

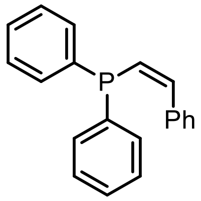

(*Z*)-diphenyl(styryl)phosphine (*Z*-3bb)

The general procedure was applied to **1b** (7 mg, 0.010 mmol, 5 mol %), diphenyl(phenylethynyl)phosphine **2bb** (57 mg, 0.2 mmol), Mg (10 mg), TMSCl (25 *u*L), 4 Å MS (25 mg) and THF (2 mL). The reaction mixture was stirred under H_2 (50 atm) at r.t. for 24 h. Then the resulting crude product was purified by column chromatography on silica gel with (PE:EA = 50:1) to afford the title compound as a white solid (43 mg, 75% yield, *Z*:*E* = 95:5). ^{1}H NMR (400 MHz, $CDCl_3$) δ 7.57–7.45 (m, 6H), 7.41–7.28 (m, 10H), 6.50 (dd, J = 12.8, 2.8 Hz, 1H); ^{13}C NMR (100 MHz, $CDCl_3$) δ 144.2 (d, J = 19.1 Hz), 139.4 (d, J = 9.5 Hz), 137.0 (d, J = 2.3 Hz), 132.8 (d, J = 18.8 Hz), 129.7 (d, J = 8.3 Hz), 129.6 (d, J = 16.0 Hz), 128.7, 128.6, 128.6, 128.2;

^{31}P NMR (162 MHz, $CDCl_3$) δ -24.8. Spectroscopic data are in accordance with those described in the literature.[29]

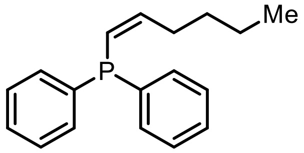

(*Z*)-hex-1-en-1-yldiphenylphosphine (*Z*-3bc)

The general procedure was applied to **1b** (3 mg, 0.004 mmol, 2 mol %), hex-1-yn-1-yldiphenylphosphine **2bc** (53 mg, 0.2 mmol), Mg (10 mg), TMSCl (25 *u*L), 4 Å MS (25 mg) and THF (2 mL). The reaction mixture was stirred under H_2 (50 atm) at 40 °C for 24 h. Then the resulting crude product was purified by column chromatography on silica gel with (PE:EA = 50:1) to afford the title compound as a colorless liquid (43 mg, 81% yield, *Z*:*E* > 99:1). ^{1}H NMR (400 MHz, $CDCl_3$) δ 7.45–7.37 (m, 4H), 7.36–7.27 (m, 6H), 6.44 (ddt, *J* = 23.8, 11.2, 7.2 Hz, 1H), 6.21–6.14 (m, 1H), 2.46 (td, *J* = 7.2, 1.6 Hz, 2H), 1.44–1.28 (m, 4H), 0.89 (t, *J* = 7.2 Hz, 3H); ^{13}C NMR (100 MHz, $CDCl_3$) δ 148.2 (d, *J* = 24.4 Hz), 139.7 (d, *J* = 9.1 Hz), 132.7 (d, *J* = 18.7 Hz), 128.5 (d, *J* = 6.5 Hz), 128.3, 127.8 (d, *J* = 9.0 Hz), 31.5, 30.94 (d, *J* = 21.1 Hz), 22.4, 14.1; ^{31}P NMR (162 MHz, $CDCl_3$) δ -31.3. HRMS (ESI, m/z): calcd for $C_{18}H_{22}P$ $[M+H]^+$: 269.1454, found: 269.1446.

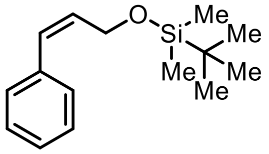

***(Z)-tert*-butyldimethyl((3-phenylallyl)oxy)silane (*Z*-3bd)**

The general procedure was applied to **1b** (7 mg, 0.01 mmol, 5 mol %), *tert*-butyldimethyl((3-phenylprop-2-yn-1-yl)oxy)silane **2bd** (49 mg, 0.2 mmol), Mg (10 mg), TMSCl (25 *u*L), 4 Å MS (25 mg) and THF (2 mL). The reaction mixture was stirred under H_2 (50 atm) at 40 °C for 24 h. Then the resulting crude product was purified by column chromatography on silica gel with (PE:EA = 50:1) to afford the

title compound as a colorless liquid (38 mg, 76% yield, *Z*:*E* = 98:2). (Due to the formation of perhydrogenation product, the pure product cannot be separated by column chromatography on silica). ^{1}H NMR (400 MHz, $CDCl_3$) δ 7.38–7.32 (m, 2H), 7.28 (d, *J* = 5.2 Hz, 1H), 7.21 (d, *J* = 7.2 Hz, 2H), 6.52 (d, *J* = 11.8 Hz, 1H), 5.85 (dt, *J* = 11.8, 6.2 Hz, 1H), 4.48 (dd, *J* = 6.2, 1.6 Hz, 2H), 0.92 (s, 9H), 0.08 (s, 6H); ^{13}C NMR (100 MHz, $CDCl_3$) δ 137.0, 132.7, 129.7, 128.9, 128.3, 127.1, 60.5, 26.1, 18.5, -5.0. Spectroscopic data are in accordance with those described in the literature.[30]

7. Supplementary Reaction Profile for Hydrogenation of 2a

7. 1 Reaction Profile for *trans*-Hydrogenation of 2a with Complex 1a

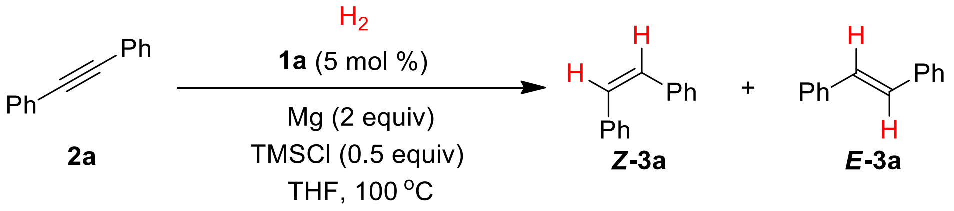

In a high-pressure autoclave was placed a tube containing **1a** (6 mg, 0.01 mmol), **2a** (36 mg, 0.2 mmol), Mg (10 mg), TMSCl (13 *u*L) and THF (2 mL). The reaction mixture was stirred at 100 °C under H_2 (10 atm). The related yields for ***Z*-3a** and ***E*-3a**, and recovery of **2a** were determined by GC analysis using mesitylene as internal standard.

Supplementary Table S5. Studying Reaction Profile for *trans*-Hydrogenation of 2a with 1a

Time (h)	Yield (***Z*-3a**)	Yield (***E*-3a**)	Recovery (**2a**)
0.5	1.6%	0.4%	98%
1	8.7%	2.3%	88%
2	12%	2%	84%
4	24%	6.8%	68%
6	38%	11%	49%
7	36%	34%	26%
8	36%	57%	5%
10	33%	65%	0
12	25%	70%	3%
14	22%	73%	0
16	17%	76%	0
18	15%	82%	0
20	9%	87%	0
22	0	91%	0

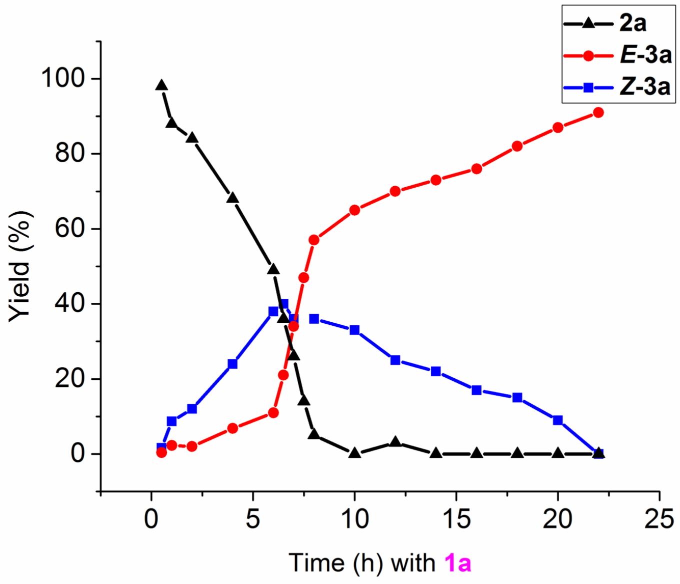

Supplementary Figure S5. Reaction profile for *trans*-hydrogenation of 2a with 1a.

7. 2 Reaction Profile for *cis*-Hydrogenation of 2a with Complex 1b

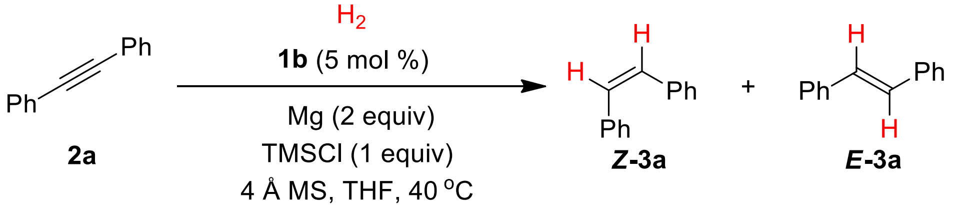

In a high-pressure autoclave was placed a tube containing **1b** (7 mg, 0.01 mmol), **2a** (36 mg, 0.2 mmol), Mg (10 mg), 4 Å MS (25 mg), TMSCl (25 *u*L) and THF (2 mL). The reaction mixture was stirred at 40 °C under H_2 (50 atm). The related yields for ***Z*-3a** and ***E*-3a**, and recovery of **2a** were determined by GC analysis using mesitylene as internal standard.

Supplementary Table S6. Studying Reaction Profile for *cis*-Hydrogenation of 2a with 1b

Time (h)	Yield (**Z-3a**)	Yield (***E*-3a**)	Recovery (**2a**)
2	3%	0	97%
4	10%	1%	88%
6	25%	2%	69%
8	40%	2%	55%
10	50%	3%	41%
12	60%	5%	30%
14	68%	4%	22%
16	72%	6%	17%
18	75%	7%	11%
20	84%	5%	1%
22	86%	6%	0

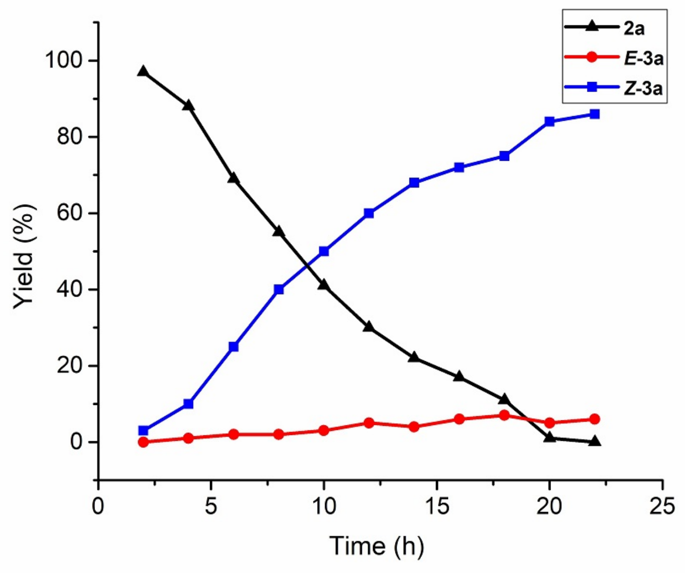

Supplementary Figure S6. Reaction profile for *cis*-hydrogenation of 2a with 1b.

8. Supplementary Mechanistic Experiments.

8.1 *Cis*-to-*trans* Isomerization with Complex 1a

The hydrogenation was carried out with **1a** (1, 3, 5, 7, 10, 20 mol%), ***Z*-3a** (36 mg, 0.2 mmol), Mg (10 mg), and TMSCl (13 *u*L) in THF (2 mL). The reaction mixture was stirred at 90 °C under H_2 (10 atm) for 24 h. The related yields for each compounds were determined by GC analysis using mesitylene as internal standard.

Supplementary Table S7. Studying the Effect of Complex 1a on the Stereoisomerization of *Z*-3a

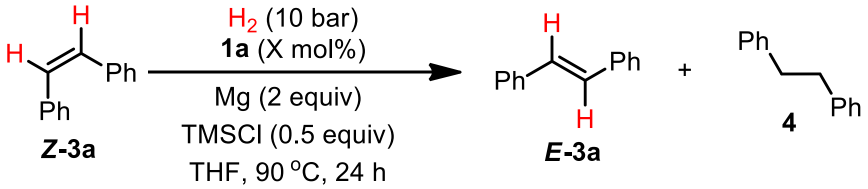

1a	yield (*E*-3a)	recovery (*Z*-3a)	yield (4)
1 mol %	18%	80%	nd
3 mol %	58%	37%	nd
5 mol %	92%	4%	nd
7 mol %	84%	nd	10%
10 mol %	51%	nd	46%
20 mol %	12%	nd	83%

8.2 *Cis*-to-*trans* Isomerization with Complex 1b

The hydrogenation was carried out with **1b** (1, 3, 5, 7, 10, 20 mol%), ***Z*-3a** (36 mg, 0.2 mmol), Mg (10 mg), and TMSCl (25 *u*L) in THF (2 mL). The reaction mixture was stirred at 40 °C under H_2 (50 atm) for 24 h. The related yields for each compounds were determined by GC analysis using mesitylene as internal standard.

Supplementary Table S8. Studying the Effect of Complex 1b on the Stereoisomerization of *Z*-3a

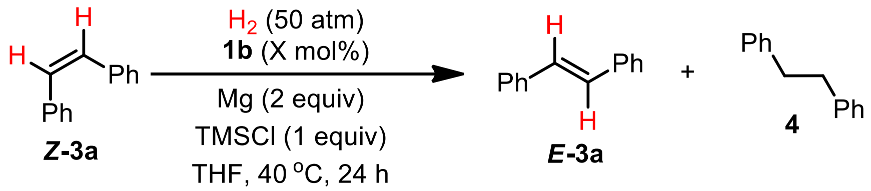

1b	yield (*E*-3a)	recovery (*Z*-3a)	yield (4)
1 mol %	~2%	97%	nd
3 mol %	7%	91%	nd
5 mol %	11%	85%	nd
7 mol %	14%	79%	nd
10 mol %	<2%	<2%	74%
20 mol %	nd	nd	68%

8.3 Preparation and Characterization of the Resting Species IV

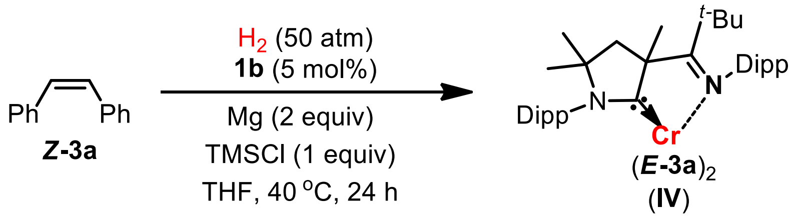

The preparation of the resting species **IV** was carried out with complex **1b** (7 mg, 0.01 mmol, 5 mol %), ***Z*-3a** (36 mg, 0.2 mmol), Mg (10 mg) and TMSCl (25 *u*L) in THF (2 mL). The reaction mixture was stirred under H_2 (50 atm) at 40 °C for 24 h. After filtration and removal of the volatiles under vacuum, the residue was wash with hexane under atmosphere of nitrogen. The resulting complex was characterized by analysis of ^{1}H NMR, HRMS and XPS techniques. The formation of resting complex **IV** by ligation of *E*-stereomer with CAAC-imino-Cr in a 2:1 ratio can be considered. ^{1}H NMR (400 MHz, $CDCl_3$): δ 7.52 (d, *J* = 7.4 Hz, 6H), 7.36 (t, *J* = 7.6 Hz, 7H), 7.31–7.28 (m, 3H), 7.23–7.13 (m, 6H), 7.11 (s, 4H, $C_{olefin}H$), 7.09–6.97 (m, 4H), 3.25–3.19 (m, 1H, C*H*Me$_2$), 3.11–3.06 (m, 1H, C*H*Me$_2$), 3.01–2.96 (m, 1H, C*H*Me$_2$), 2.81–2.74 (m, 1H, C*H*Me$_2$), 2.44 (d, *J* = 12.4 Hz, 1H, C*H*$_2$). HRMS (ESI, m/z): calcd

for $C_{64}H_{78}CrN_2Na$ $[M+Na]^+$: 949.5462, found: 949.5483.

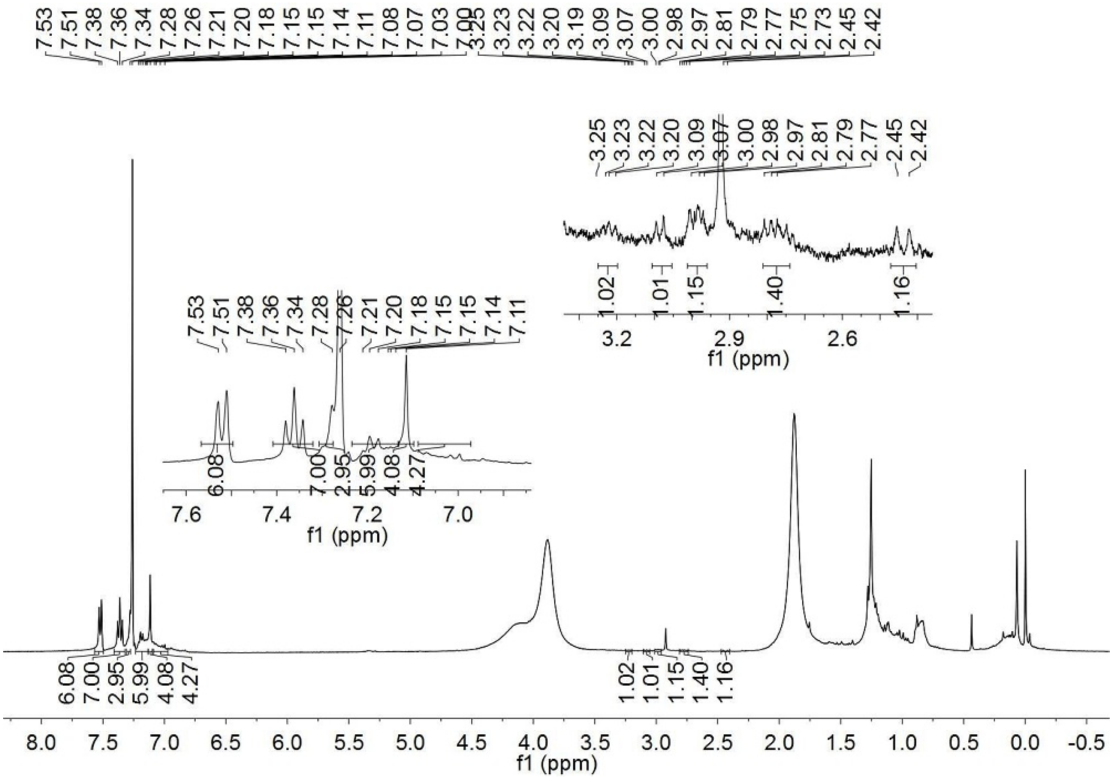

Supplementary Figure S7. ^{1}H NMR spectrum of the resting species IV (400 MHz, $CDCl_3$, 25 ^{o}C).

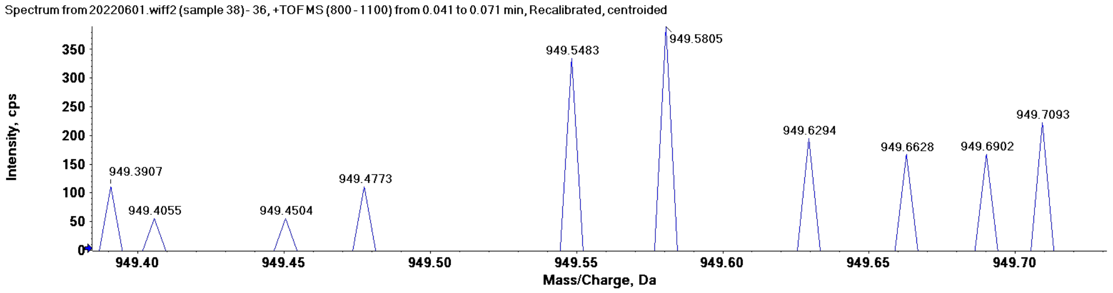

Supplementary Figure S8. HMRS analysis (ESI$^+$)

The oxidation state of the Cr species **IV** was analyzed by Cr 2p core-level X-ray photoelectron spectroscopy (XPS), which were fitted with spin-orbital split $2p_{3/2}$ and $2p_{1/2}$ components (Figure *S8*). The related spectrum shows two sets of XPS peaks, with the $2p_{1/2}$ component appearing at 583.43 eV and the $2p_{3/2}$ component appearing

at 574.28 eV of the binding energy, respectively. These data are nearly in accordance with that of related binding energies for Cr(0), indicating that the formation of Cr(0) species can be considered.

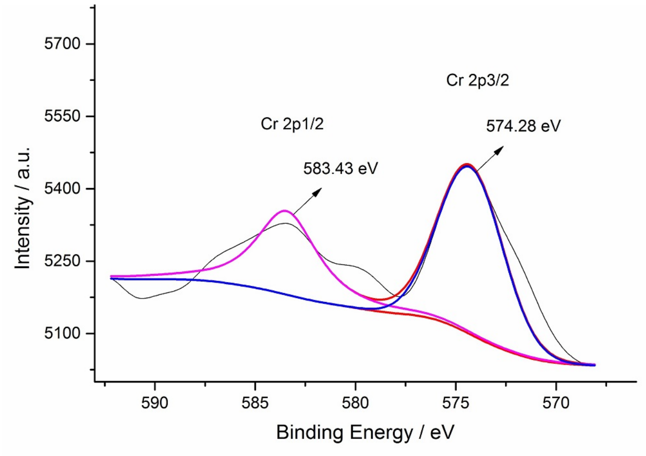

Supplementary Figure S9. Binding energies of XPS spectra of the resting species IV.

8.4 The Catalytic Hydrogenation of 2j with Resting Species IV

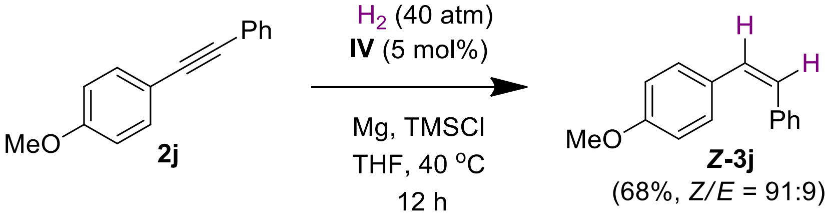

The catalytic activity of the resting species **IV** in the hydrogenation of **2j** was studied. 1-Methoxy-4-(phenylethynyl)benzene **2j** (0.2 mmol, 42 mg) was treated with **IV** (0.01 mmol, 9 mg), Mg (10 mg), TMSCl (25 *u*L) under atmosphere of hydrogen (40 atm). The reaction mixture was stirred at 40 °C for 12 h. The formation of *Z*-selective olefin of ***Z*-3j** (68% yield, *Z*/*E* = 91:9) was observed by GC analysis with mesitylene as internal standard.

8.5 Deuterium Experiment for the *cis*-to-*trans* Isomerization

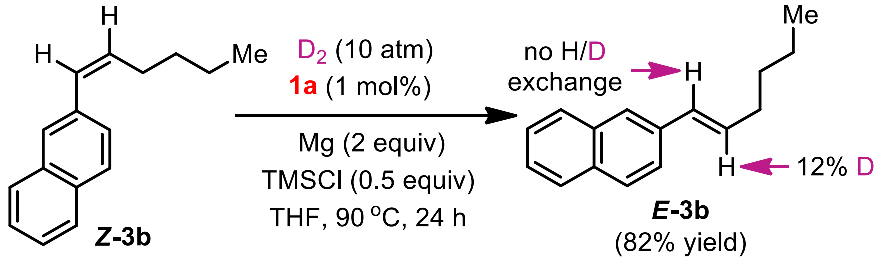

The deuterium experiment of the *cis*-to-*trans* isomerization of ***Z*-3b** (42 mg, 0.2 mmol) was performed with complex **1a** (1 mg, 0.002 mmol), Mg (10 mg) and TMSCl (13 *u*L) under D_2 (10 atm). The reaction mixture was stirred at 90 °C for 24 h. The resulting crude product was purified by column chromatography on silica gel with (PE:EA = 100:1) to afford ***E*-3b** in 82% yield. The related ^{1}H NMR spectrum suggests that no deuterium was incorporated the naphthylated olefinic carbon.

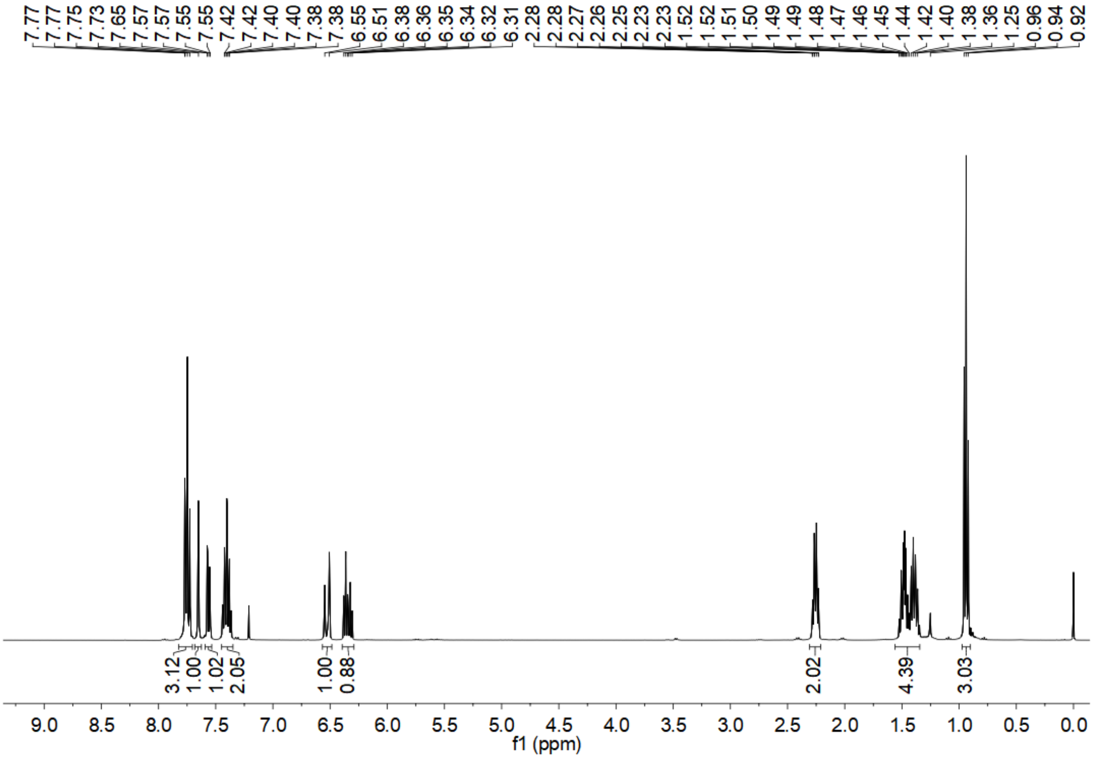

Supplementary Figure S10. ^{1}H NMR spectrum of deuterated compound *E*-3b (400 MHz, $CDCl_3$, 25 °C).

8.6 The *cis*-to-*trans* Isomerization of Alkene Without Hydrogen.

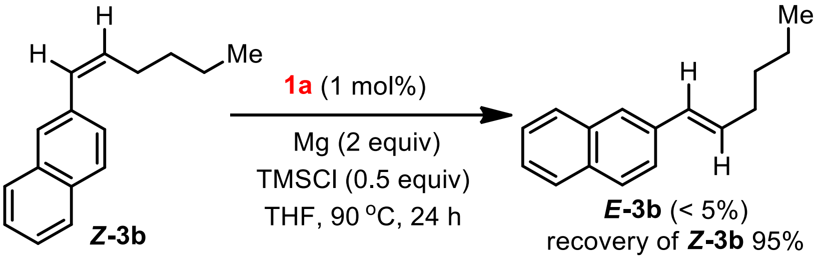

The *cis*-to-*trans* isomerization of ***Z*-3b** (42 mg, 0.2 mmol) was performed with complex **1a** (1 mg, 0.002 mmol), Mg (10 mg) and TMSCl (13 *u*L). The reaction mixture was stirred at 90 °C for 24 h. The formation of ***E*-3b** in low yield (<5%) was observed. These results suggest that the metal hydride intermediates may be involved in the reaction.

8.7 The Catalytic Hydrogenation of Alkyne Without TMSCl

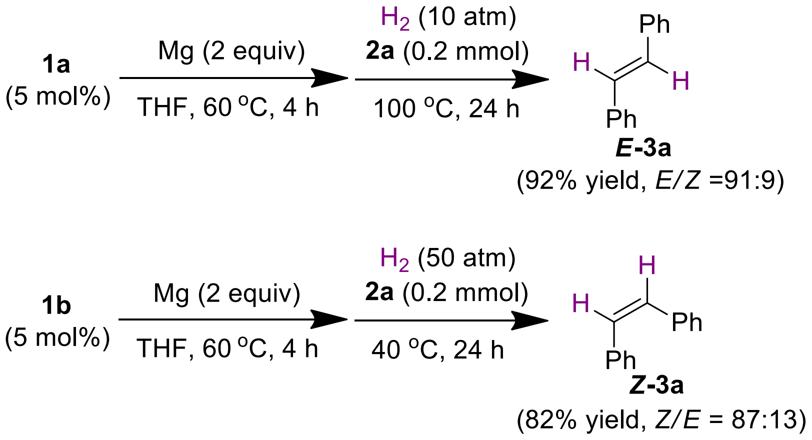

By initially reducing Cr complexes with Mg in forming reactive Cr, we investigated the catalytic hydrogenation of alkynes without the use of TMSCl. In a dried Schlenk tube were placed magnesium (10 mg, 0.4 mmol) and heated to around 400 °C under vacuum for 5 min using a heat gun. After cooling to room temperature, complex **1a** or **1b** (0.01 mmol, 6 or 7 mg) (**1a** and **1b** were prepared and worked up under inert atmosphere) and dry THF (2 mL) was added under atmosphere of nitrogen. After stirring the mixture at 60 °C for 24 h, alkyne **2a** (0.2 mmol, 32 mg) was added and the

mixture was treated with H_2 (1 or 50 atm) at 100 or 40 °C for 24 h. The formation of ***E*-3a** or ***Z*-3a** in yield of 92% yield (*E*/*Z* = 91:9) or 82% yield (*Z*/*E* = 87:13) was observed, respectively. These results suggest that the *E*/*Z* selective hydrogenation of alkynes occurs smoothly with reactive Cr species without TMSCl.

8.8 The Catalytic Hydrogenation Using the In-Situ Formed Reactive Cr Without Mg

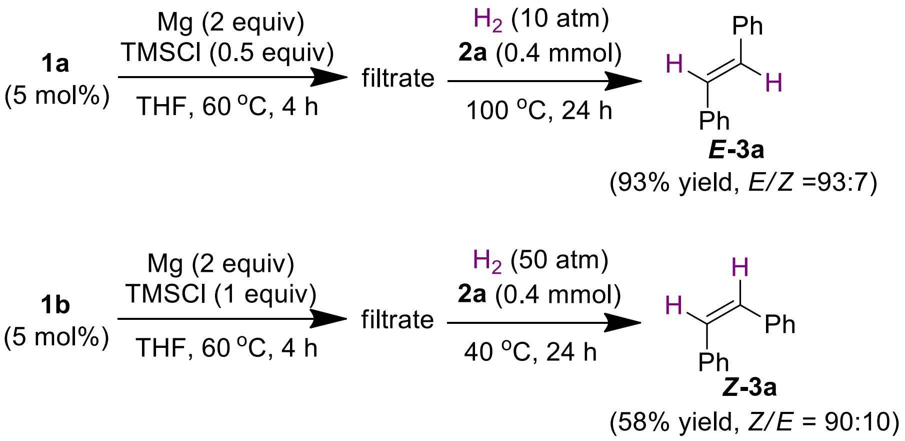

We studied the catalytic hydrogenation of alkynes with the in-situ formed reactive Cr complex without the use of magnesium. In a dried Schlenk tube were placed complex **1a** or **1b** (0.01 mmol, 13 or 14 mg), magnesium (20 mg, 0.8 mmol), TMSCl (25 or 50 *u*L) and dry THF (2 mL) was added under atmosphere of nitrogen. After stirring the mixture at 60 °C for 4 h. The resulting mixture was filtrated under atmosphere of nitrogen, alkyne **2a** (0.4 mmol, 64 mg) was added and the mixture was treated with H_2 (1 or 50 atm) at 100 or 40 °C for 24 h. The formation of ***E*-3a** or ***Z*-3a** in yield of 93% yield (*E*/*Z* = 93:7) or 58% yield (*Z*/*E* = 90:10) was observed, respectively. These results suggest that the catalytic hydrogenation of alkynes with in-situ formed reactive Cr occurs smoothly without the use of magnesium.

8.9 Synthesis of Chromium carbonyl complexes

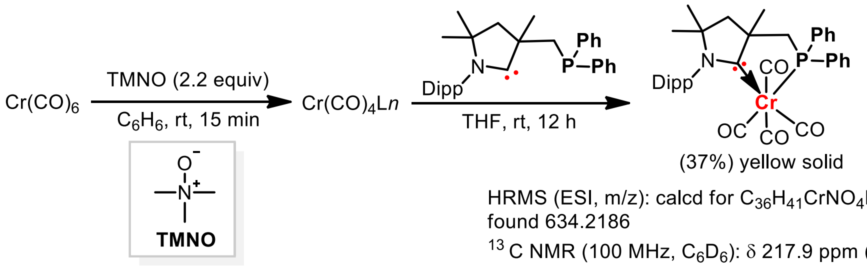

HRMS (ESI, m/z): calcd for $C_{36}H_{41}CrNO_4P$ $[M+H]^+$: 634.2173, found 634.2186

^{13}C NMR (100 MHz, C_6D_6): δ 217.9 ppm (C=O)

^{31}P NMR (162 MHz, C_6D_6): δ 39.0 ppm

IR (neat): 1919 cm^{-1} (C=O)

CAAC-phosphino-$Cr(CO)_4$ was prepared based on the method previously described.[31-33] $Cr(CO)_6$ (220 g, 1 mmol) and trimethylamine-*N*-oxide (TMNO) (165 mg, 2.2 mmol) were added to 10 mL of toluene in a dry Schlenk flask and stirred for 15 minutes until the solution turned yellow. The free carbene ((1 mmol, 0.1 M in THF) The free carbene was prepared according to the method for the synthesis of **CAAC-Cr Complex 1a**) solution was transferred to Schlenk tube containing $Cr(CO)_4$L*n* under atmosphere of nitrogen at 0 °C and stirred at room temperature for 12 h. After removal of volatiles under vacuum, the solid was then extracted with DCM (20 mL). After removal of DCM, the solid was washed with hexane (30 mL) to afford a yellow solid (234 mg, 37%). IR (neat) ν 3434, 2968, 2921, **1919 (C=O)**, 1641, 1462, 1434, 1382, 1117, 804, 667, 652 cm^{-1}. ^{1}H NMR (400 MHz, C_6D_6) δ 8.25–7.53 (m, 4H), 7.37–7.17 (m, 5H), 7.12–6.80 (m, 4H), 3.86–3.49 (m, 2H), 3.42–2.93 (m, 2H), 2.49 (d, *J* = 12.8 Hz, 1H), 1.89 (d, *J* = 12.2 Hz, 1H), 1.42–1.32 (m, 3H), 1.32–1.27(m, 3H), 1.26–1.16 (m, 6H), 1.15–1.03 (m, 3H), 0.98–0.79 (m, 6H); ^{13}C NMR (100 MHz, C_6D_6) δ **217.9 (C≡O)**; ^{31}P NMR (162 MHz, C_6D_6) δ **39.0**. HRMS (ESI, m/z): calcd for $C_{36}H_{41}CrNO_4P$ $[M+H]^+$: 634.2173, found 634.2186.

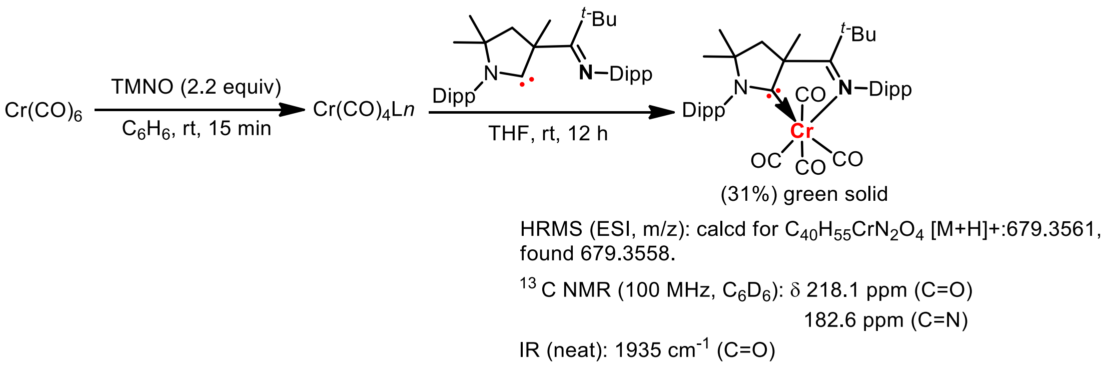

CAAC-imino-$Cr(CO)_4$ was prepared based on the method previously described.[31-33] $Cr(CO)_6$ (220 g, 1 mmol) and trimethylamine-*N*-oxide (TMNO) (165 mg, 2.2 mmol) were added to 10 mL of toluene in a dry Schlenk flask and stirred for 15 minutes until the solution turned yellow. The free carbene ((1 mmol, 0.1 M in THF) The free carbene was prepared according to the method for the synthesis of **CAAC-Cr Complex 1b**) solution was transferred to Schlenk tube containing $Cr(CO)_4L$*n* under atmosphere of nitrogen at 0 °C and stirred at room temperature for 12 h. After removal of volatiles under vacuum, the solid was then extracted with DCM (20 mL). After removal of DCM, the solid was washed with hexane (30 mL) to afford a green solid (210 mg, 31%). IR (neat) ν 3436, 2966, 2870, **1935 (C=O)**, 1637, 1465, 1384, 1305, 1124, 1084, 1063, 1035, 937, 808, 534, 522 cm^{-1}. ^1H NMR (400 MHz, C_6D_6) δ 7.11–6.86 (m, 5H), 6.83–6.69 (s, 1H), 3.16–2.90 (m, 1H), 2.73–2.58 (m, 2H), 2.46–2.33 (m, 1H), 2.17–2.10 (m, 1H), 1.85–1.71 (m, 1H), 1.50–1.36 (m, 6H), 1.35–1.03 (m, 26H), 1.00–0.85 (m, 6H), 0.55 (s, 3H); ^{13}C NMR (100 MHz, C_6D_6) δ **218.1 (C≡O)**, **182.6 (C=N)**. HRMS (ESI, m/z): calcd for $C_{40}H_{55}CrN_2O_4$ $[M+H]^+$:679.3561, found 679.3558.

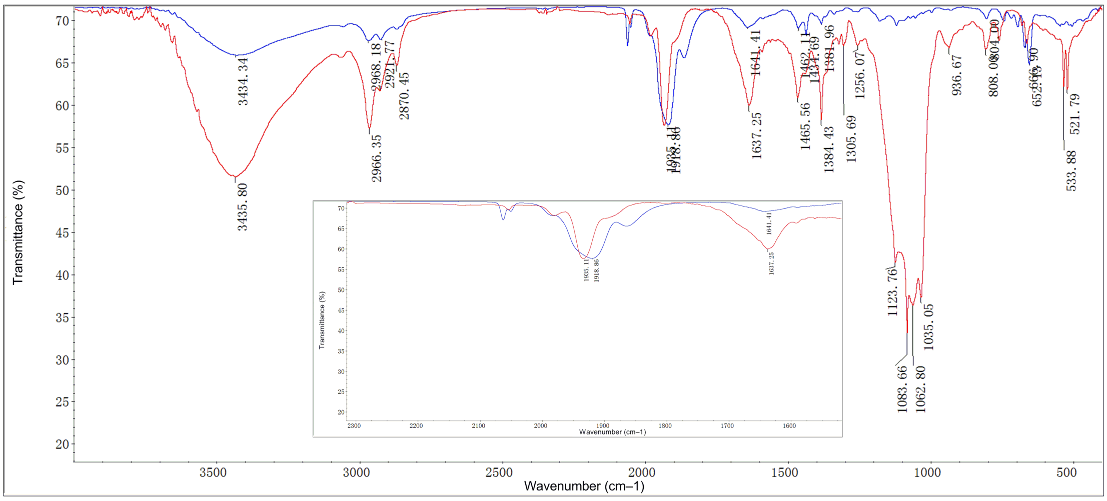

Supplementary Figure S11. IR spectra of (CAAC-phosphino-Cr(CO)$_4$ (blue line) and CAAC-imino-Cr(CO)$_4$ (red line)

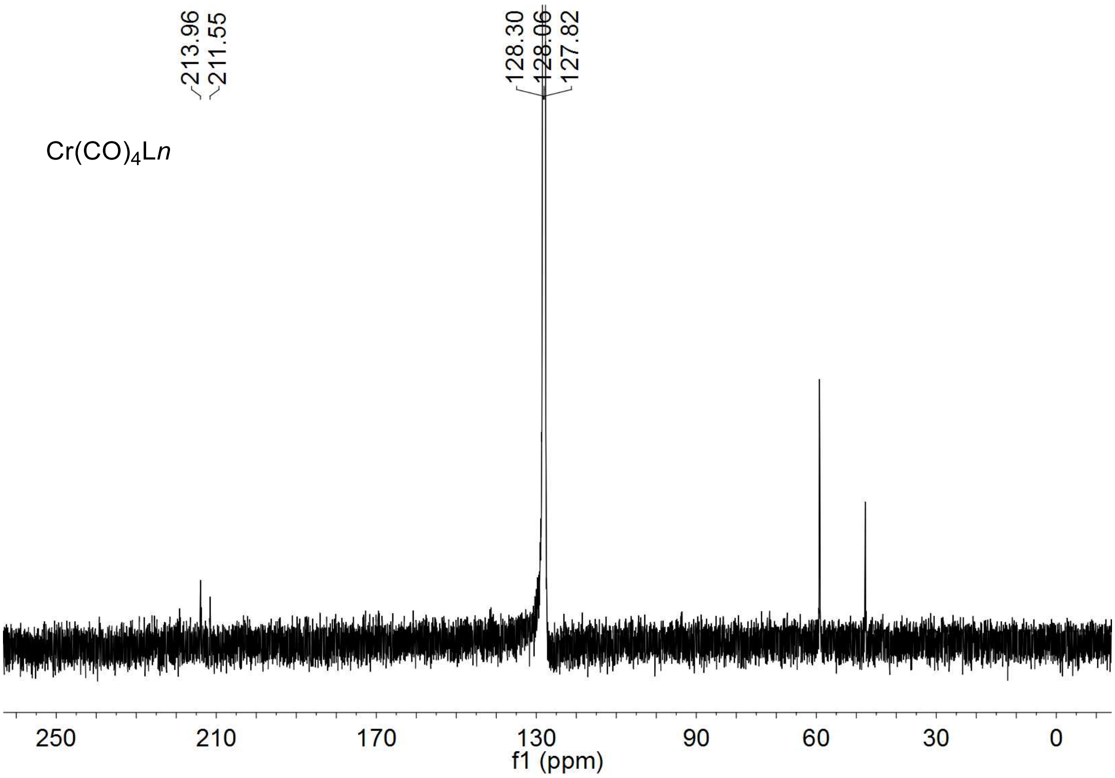

Supplementary Figure S12. ^{13}**C NMR spectrum for complex of Cr(CO)$_4$L$_n$ (100 MHz, CDCl$_3$, 25 °C).**

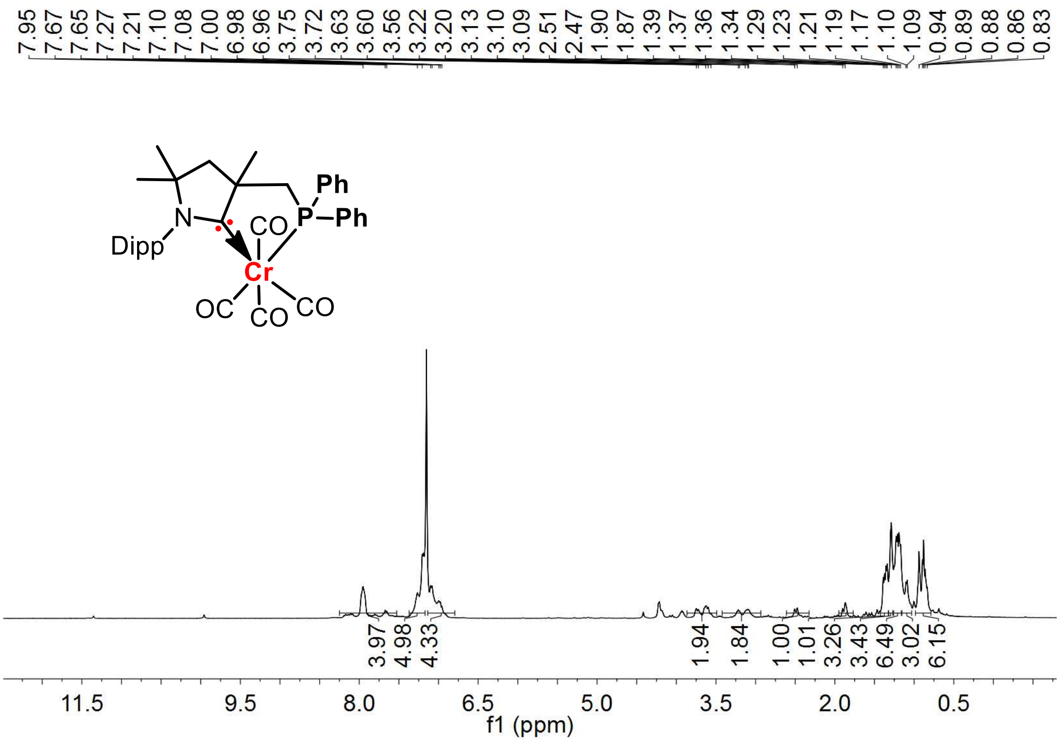

Supplementary Figure S13. ^{1}H NMR spectrum for complex of CAAC-phosphino-$Cr(CO)_4$ (400 MHz, $CDCl_3$, 25 °C).

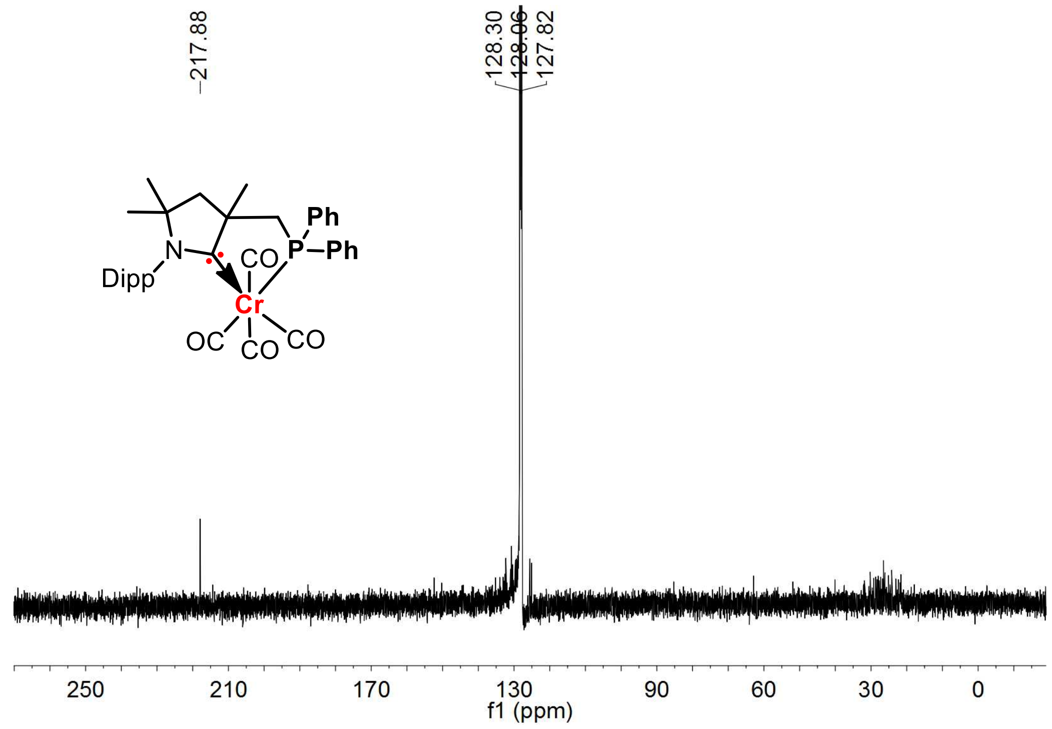

Supplementary Figure S14. ^{13}C NMR spectrum for complex of CAAC-phosphino -$Cr(CO)_4$ (100 MHz, $CDCl_3$, 25 °C).

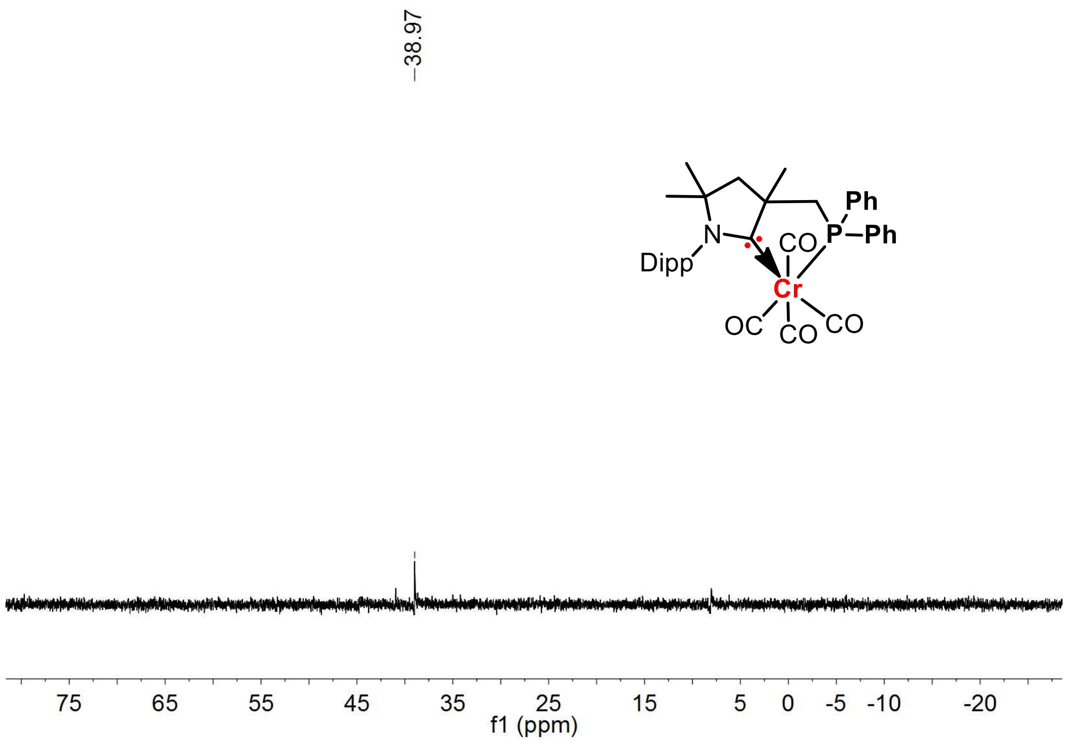

Supplementary Figure S15. ^{31}P NMR spectrum for complex of CAAC-phosphino -Cr(CO)4 (162 MHz, CDCl3, 25 °C).

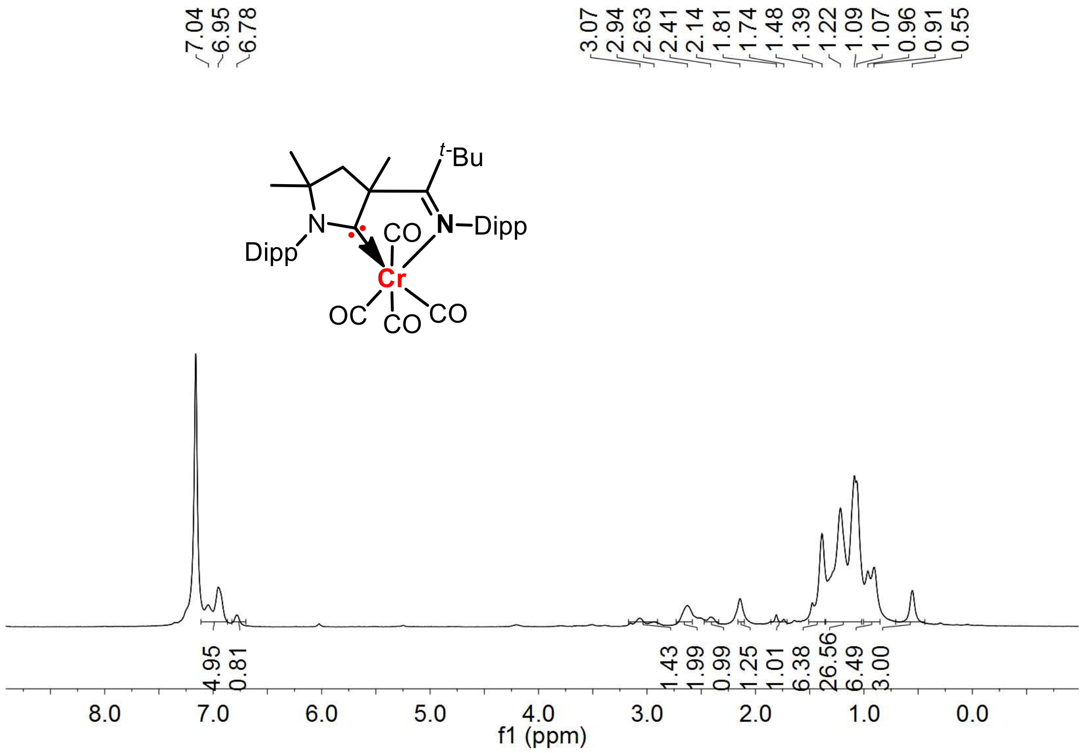

Supplementary Figure S16. ^{1}H NMR spectrum for complex of CAAC-imino -Cr(CO)4 (400 MHz, CDCl3, 25 °C).

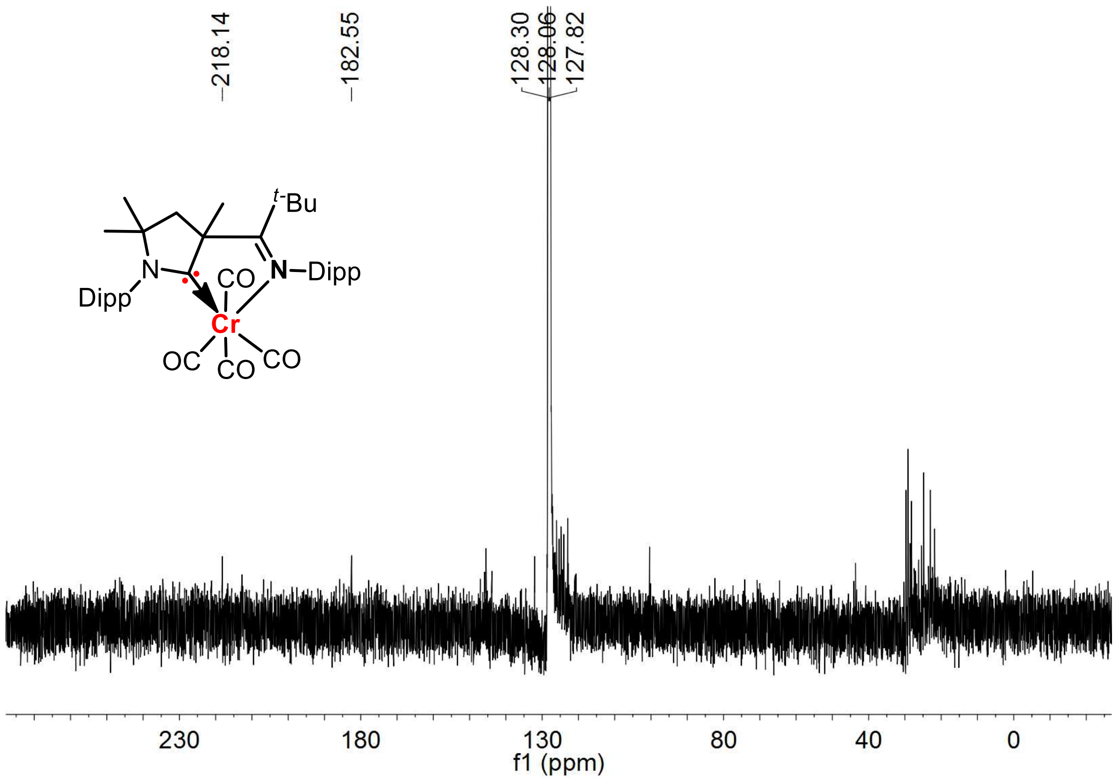

Supplementary Figure S17. ^{13}**C NMR spectrum for complex of CAAC-imino-Cr(CO)$_4$ (100 MHz, CDCl$_3$, 25 °C).**

8.10 Discussion of the effect of ligands on hydrogenation of alkynes.

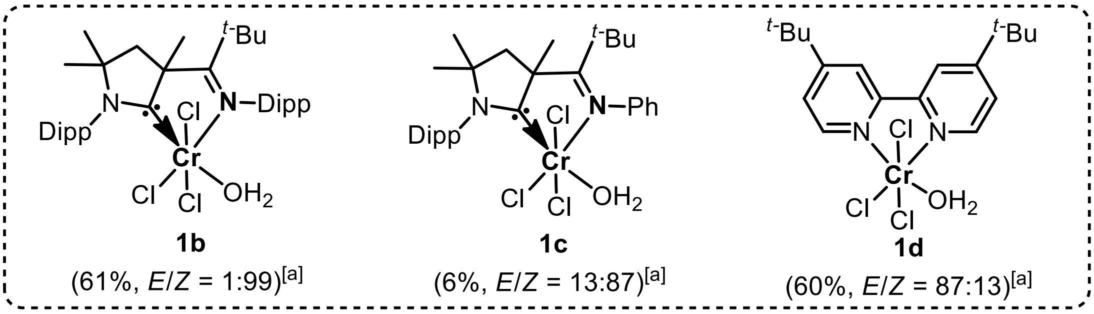

The phosphino-containing complex **1a** shows the ability in promoting the addition of hydrogen to alkyne in a *trans*-selective manner, forming *E*-stilbene (***E*-3a**) in up to 99:1 *E*/*Z* selectivity. By contrast, complex **1b** that contains an imino anchor in CAAC ligand is able to switch the stereoselectivity in alkyne hydrogenation to give 99:1 *Z*/*E* selectivity. The replacement of 2,6-di-isopropylphenyl substituent of imino in CAAC ligand (**1b**) by phenyl group (**1c**) led to low *Z*-selectivity and conversion in the alkyne

hydrogenation. These differences with these ligands may ascribe to the different steric and electronic effect that are caused by the two imino scaffolds. Hydrogenation of alkyne by using 4,4'-di-*tert*-butylbipyridine as ligand (**1d**) formed *E*-stereomer as major product (see Table S1).

9. Supplementary Synthetic Applications in Accessing Functionalized *E*- and *Z*-olefin Derivatives

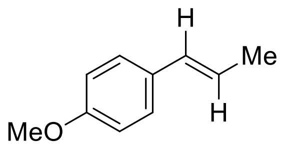

(*E*)-1-methoxy-4-(prop-1-en-1-yl)benzene (*E*-5)

The general procedure was applied to **1a** (32 mg, 0.05 mmol, 0.5 mol %), 1-methoxy-4-(prop-1-yn-1-yl)benzene **4** (1.46 g, 10 mmol), Mg (48 mg), TMSCl (127 *u*L) and THF (60 mL). The reaction mixture was stirred under H_2 (15 atm) at 100 °C for 48 h. Then the resulting crude product was purified by column chromatography on silica gel with (PE:EA = 100:1) to afford the title compound as a yellow liquid (1.36 g, 92% yield, *E*/*Z* = 97:3). ^{1}H NMR (400 MHz, $CDCl_3$): δ 7.25–7.16 (m, 2H), 6.84–6.71 (m, 2H), 6.30 (dd, *J* = 15.8, 1.6 Hz, 1H), 6.11–5.98 (m, 1H), 3.75 (s, 3H), 1.81 (dd, *J* = 6.6, 1.8 Hz, 3H); ^{13}C NMR (100 MHz, $CDCl_3$): δ 158.7, 131.0, 130.5, 127.0, 123.6, 114.0, 55.4, 18.5. Spectroscopic data are in accordance with those described in the literature.[34]

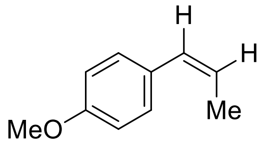

(*Z*)-1-methoxy-4-(prop-1-en-1-yl)benzene (*Z*-5)

The general procedure was applied to **1b** (35 mg, 0.05 mmol, 0.5 mol %), 1-methoxy-4-(prop-1-yn-1-yl)benzene **4** (1.46 g, 10 mmol), Mg (48 mg), TMSCl (127

uL), 4 Å MS (1 g) and THF (60 mL). The reaction mixture was stirred under H_2 (50 atm) at 40 °C for 24 h. Then the resulting crude product was purified by column chromatography on silica gel with (PE:EA = 100:1) to afford the title compound as a colorless oil (1.42 g, 96% yield, *Z*/*E* = 95:5). ^{1}H NMR (400 MHz, $CDCl_3$): δ 7.30–7.19 (m, 2H), 6.94–6.83 (m, 2H), 6.37 (dt, *J* = 12.8, 6.2 Hz, 1H), 5.70 (dq, *J* = 11.6, 7.2 Hz, 1H), 3.82 (s, 3H), 1.90 (dd, *J* = 7.2, 1.8 Hz, 3H); ^{13}C NMR (100 MHz, $CDCl_3$): δ 158.2, 130.5, 130.1, 129.4, 125.3, 113.7, 55.4, 14.7. Spectroscopic data are in accordance with those described in the literature.[35]

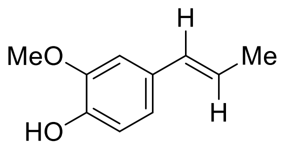

(*E*)-2-methoxy-4-(prop-1-en-1-yl)phenol (*E*-7)

The general procedure was applied to **1a** (32 mg, 0.05 mmol, 0.5 mol %), 2-methoxy-4-(prop-1-yn-1-yl)phenol **6** (1.62 g, 10 mmol), Mg (288 mg), TMSCl (1.39 mL) and THF (60 mL). The reaction mixture was stirred under H_2 (15 atm) at 100 °C for 48 h. Then the resulting crude product was purified by column chromatography on silica gel with (PE:EA = 100:1) to afford the title compound as a yellow liquid (1.43 g, 87% yield, *E*/*Z* = 98:2). ^{1}H NMR (400 MHz, $CDCl_3$): δ 6.87–6.80 (m, 3H), 6.36–6.28 (m, 1H), 6.07 (dq, *J* = 15.8, 6.6 Hz, 1H), 5.55 (s, 1H), 3.90 (s, 3H), 1.86 (dd, *J* = 6.6, 1.8 Hz, 3H); ^{13}C NMR (100 MHz, $CDCl_3$): δ 146.7, 144.9, 130.9, 130.8, 123.6, 119.5, 114.5, 108.0, 56.0, 18.5. Spectroscopic data are in accordance with those described in the literature.[34]

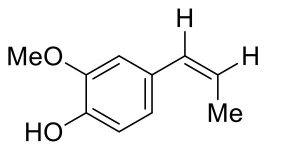

(*Z*)-2-methoxy-4-(prop-1-en-1-yl)phenol (*Z*-7)

The general procedure was applied to **1b** (35 mg, 0.05 mmol, 0.5 mol %), 2-methoxy-4-(prop-1-yn-1-yl)phenol **6** (1.62 g, 10 mmol), Mg (288 mg), TMSCl (1.39 mL), 4 Å MS (1 g) and THF (60 mL). The reaction mixture was stirred under H_2 (50 atm) at 40 °C for 24 h. Then the resulting crude product was purified by column chromatography on silica gel with (PE:EA = 10:1) to afford the title compound as a colorless oil (1.38 g, 84% yield, *Z*/*E* = 94:6). ^{1}H NMR (400 MHz, $CDCl_3$): δ 6.91–6.88 (m, 1H), 6.85–6.80 (m, 2H), 6.41–6.30 (m, 1H), 5.70 (dq, *J* = 11.6, 7.2 Hz, 1H), 5.58 (s, 1H), 3.89 (s, 3H), 1.90 (dd, *J* = 7.2, 1.8 Hz, 3H); ^{13}C NMR (100 MHz, $CDCl_3$): δ 146.3, 144.4, 130.2, 129.8, 125.3, 122.2, 114.2, 111.6, 56.0, 14.8. HRMS (ESI, m/z): calcd for $C_{10}H_{13}O_2$ $[M+H]^+$: 165.0910, found: 165.0905.

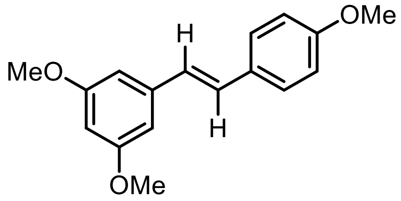

(*E*)-1,3-dimethoxy-5-(4-methoxystyryl)benzene (*E*-9)

The general procedure was applied to **1a** (6 mg, 0.01 mmol, 5 mol %), 1,3-dimethoxy-5-((4-methoxyphenyl)ethynyl)benzene **8** (54 mg, 0.2 mmol), Mg (10 mg), TMSCl (13 *u*L) and THF (2 mL). The reaction mixture was stirred under H_2 (10 atm) at 90 °C for 24 h. Then the resulting crude product was purified by column chromatography on silica gel with (PE:EA = 20:1) to afford the title compound as a white solid (52 mg, 93% yield, *E*/*Z* = 99:1). ^{1}H NMR (400 MHz, $CDCl_3$): δ 7.49–7.42 (d, *J* = 8.7 Hz, 2H), 7.05 (d, *J* = 16.4 Hz, 1H), 6.95–6.87 (m, 3H), 6.67 (d, *J* = 2.2 Hz, 2H), 6.39 (t, *J* = 2.2 Hz, 1H), 3.84 (s, 6H), 3.83 (s, 3H); ^{13}C NMR (100 MHz, $CDCl_3$): δ 161.1, 159.5, 139.8, 130.0, 128.9, 127.9, 126.7, 114.3, 104.5, 99.7, 55.5, 55.4. Spectroscopic data are in accordance with those described in the literature.[36]

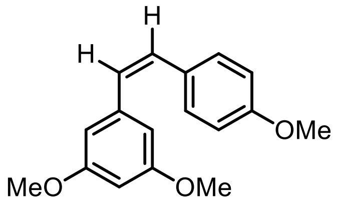

(*Z*)-1,3-dimethoxy-5-(4-methoxystyryl)benzene (*Z*-9)

The general procedure was applied to **1b** (7 mg, 0.01 mmol, 5 mol %), 1,3-dimethoxy-5-((4-methoxyphenyl)ethynyl)benzene **8** (54 mg, 0.2 mmol), Mg (10 mg), TMSCl (25 *u*L), 4 Å MS (25 mg) and THF (2 mL). The reaction mixture was stirred under H_2 (50 atm) at 40 °C for 24 h. Then the resulting crude product was purified by column chromatography on silica gel with (PE:EA = 20:1) to afford the title compound as a colorless oil (47 mg, 88% yield, *Z*/*E* = 99:1). ^{1}H NMR (400 MHz, $CDCl_3$): δ 7.25–7.17 (m, 2H), 6.81–7.75 (m, 2H), 6.53 (d, *J* = 12.4 Hz, 1H), 6.48-6.41 (m, 3H), 6.33 (t, *J* = 2.4 Hz, 1H), 3.78 (s, 3H), 3.67 (s, 6H); ^{13}C NMR (100 MHz, $CDCl_3$): δ 160.7, 158.9, 139.6, 130.4, 130.3, 129.7, 128.8, 113.7, 106.8, 99.8, 55.4. Spectroscopic data are in accordance with those described in the literature.[36]

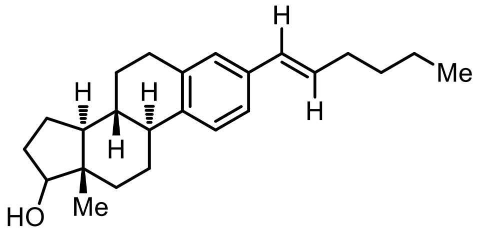

(8*R*,9*S*,13*S*,14*S*)-3-((*E*)-hex-1-en-1-yl)-13-methyl-7,8,9,11,12,13,14,15,16,17-decahydro-6H-cyclopenta[*a*]phenanthren-17-ol (*E*-11)

The general procedure was applied to **1a** (6 mg, 0.01 mmol, 5 mol %), (8*R*,9*S*,13*S*,14*S*)-3-(hex-1-yn-1-yl)-13-methyl-7,8,9,11,12,13,14,15,16,17-decahydro-6H-cyclopenta[*a*]phenanthren-17-ol **10** (67 mg, 0.2 mmol), Mg (10 mg), TMSCl (38 *u*L) and THF (2 mL). The reaction mixture was stirred under H_2 (10 atm) at 100 °C for 24 h. Then the resulting crude product was purified by column chromatography on silica gel with (PE:EA = 10:1) to afford the title compound as a white solid (57 mg,

84% yield, *E*/*Z* = 95:5). M.p. = 62–64 °C. (Due to the formation of perhydrogenation product, the pure product cannot be separated by column chromatography on silica). ^{1}H NMR (400 MHz, $CDCl_3$): δ 7.22 (t, *J* = 7.0 Hz, 1H), 7.16–6.88 (m, 2H), 6.33 (d, *J* = 15.6 Hz, 1H), 6.17 (dt, *J* = 15.8, 6.8 Hz, 1H), 3.74 (t, *J* = 8.4 Hz, 1H), 2.91–2.79 (m, 2H), 2.38–2.29 (m, 1H), 2.27–2.09 (m, 3H), 1.99–1.85 (m, 2H), 1.75–1.53 (m, 3H), 1.51–1.41 (m, 4H), 1.39–1.26 (m, 6H), 1.24–1.15 (m, 1H), 0.93 (t, J = 7.2 Hz, 3H), 0.79 (s, 3H); ^{13}C NMR (101 MHz, $CDCl_3$): δ 139.1, 136.8, 135.5, 130.6, 129.6, 126.6, 125.6, 123.4, 82.0, 50.3, 44.5, 43.4, 38.8, 36.9, 32.9, 31.7, 30.7, 29.7, 27.4, 26.3, 23.3, 22.4, 14.1, 11.2. HRMS (ESI, m/z): calcd for $C_{24}H_{35}O$ $[M+H]^+$: 339.2682, found: 339.2679.

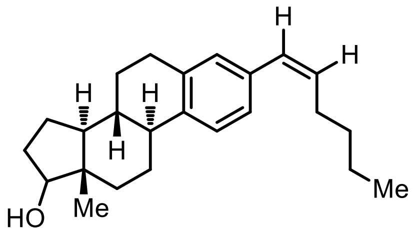

(8*R*,9*S*,13*S*,14*S*)-3-((*Z*)-hex-1-en-1-yl)-13-methyl-7,8,9,11,12,13,14,15,16,17-decahydro-6*H*-cyclopenta[*a*]phenanthren-17-ol (*Z*-11)

The general procedure was applied to **1b** (7 mg, 0.01 mmol, 5 mol %), (8*R*,9*S*,13*S*,14*S*)-3-(hex-1-yn-1-yl)-13-methyl-7,8,9,11,12,13,14,15,16,17-decahydro-6H-cyclopenta[*a*]phenanthren-17-ol **10** (67 mg, 0.2 mmol), Mg (10 mg), TMSCl (50 *u*L), 4 Å MS (25 mg) and THF (2 mL). The reaction mixture was stirred under H_2 (50 atm) at 40 °C for 24 h. Then the resulting crude product was purified by column chromatography on silica gel with (PE:EA = 10:1) to afford the title compound as a colorless oil (51 mg, 76% yield, *Z*/*E* = 99:1). ^{1}H NMR (400 MHz, $CDCl_3$): δ 7.25 (d, *J* = 8.0 Hz, 1H), 7.08 (d, *J* = 8.4 Hz, 1H), 6.99 (s, 1H), 6.33 (d, *J* = 11.6 Hz, 1H), 5.61 (dt, *J* = 11.6, 7.2 Hz, 1H), 3.73 (t, *J* = 8.4 Hz, 1H), 2.96–2.74 (m, 2H), 2.38–2.30 (m, 2H), 2.28–2.17 (m, 1H), 2.18–2.07 (m, 1H), 2.00–1.84 (m, 2H), 1.76–1.59 (m, 2H), 1.54–1.16 (m, 12H), 0.91 (t, *J* = 7.2 Hz, 3H), 0.78 (s, 3H); ^{13}C NMR (100 MHz,

CDCl3): δ 138.7, 136.5, 135.3, 132.8, 129.5, 128.5, 126.2, 125.2, 82.0, 50.3, 44.5, 43.4, 38.8, 36.9, 32.4, 30.7, 29.7, 28.6, 27.4, 26.2, 23.3, 22.6, 14.2, 11.2. HRMS (ESI, m/z): calcd for $C_{24}H_{35}O$ $[M+H]^+$: 339.2682, found: 339.2678.

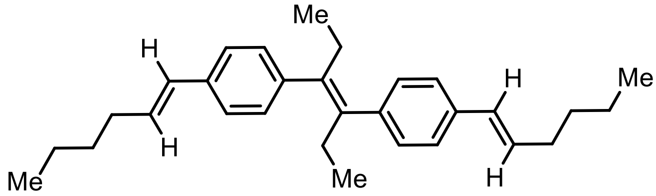

(*E*)-4,4'-((*E*)-hex-3-ene-3,4-diyl)bis(((*E*)-hex-1-en-1-yl)benzene) (*E*-13)

The general procedure was applied to **1a** (6 mg, 0.01 mmol, 5 mol %), (*E*)-4,4'-(hex-3-ene-3,4-diyl)bis(hex-1-yn-1-ylbenzene) **12** (79 mg, 0.2 mmol), Mg (10 mg), TMSCl (13 *u*L) and THF (2 mL). The reaction mixture was stirred under H_2 (10 atm) at 100 °C for 24 h. Then the resulting crude product was purified by column chromatography on silica gel with (PE:EA = 100:1) to afford the title compound as a white solid (61 mg, 76% yield, *E*/*Z* > 99:1). M.p. = 39–41 °C. ^1H NMR (400 MHz, CDCl3): δ 7.36 (d, J = 8.0 Hz, 4H), 7.15 (d, J = 8.0 Hz, 4H), 6.42 (d, J = 16.0 Hz, 2H), 6.27 (dt, J = 16.0, 6.8 Hz, 2H), 2.24 (q, J = 7.4 Hz, 4H), 2.16 (q, J = 7.4 Hz, 4H), 1.53–1.36 (m, 8H), 0.95 (t, J = 7.2 Hz, 6H), 0.78 (t, J = 7.2 Hz, 6H); ^{13}C NMR (100 MHz, CDCl3): δ 141.4, 139.2, 136.0, 130.8, 129.7, 129.1, 125.7, 32.9, 31.8, 28.6, 22.4, 14.1, 13.5. HRMS (ESI, m/z): calcd for $C_{30}H_{41}$ $[M+H]^+$: 401.3203, found: 401.3239.

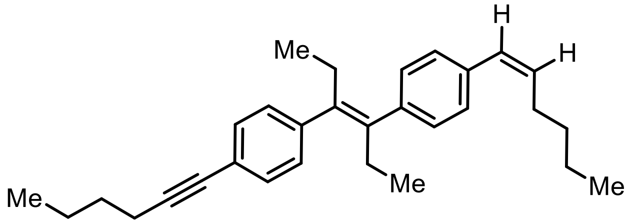

1-((*Z*)-hex-1-en-1-yl)-4-((*E*)-4-(4-(hex-1-yn-1-yl)phenyl)hex-3-en-3-yl)benzene (*Z*-13)

The general procedure was applied to **1b** (7 mg, 0.01 mmol, 5 mol %),

(*E*)-4,4'-(hex-3-ene-3,4-diyl)bis(hex-1-yn-1-ylbenzene) **12** (79 mg, 0.2 mmol), Mg (10 mg), TMSCl (25 *u*L), 4 Å MS (25 mg) and THF (2 mL). The reaction mixture was stirred under H_2 (50 atm) at 40 °C for 24 h. Then the resulting crude product was purified by column chromatography on silica gel with (PE:EA = 100:1) to afford the title compound as a yellow oil (35 mg, 44% yield, *Z*/*E* = 99:1), recovery 12 (32mg, 40%). ^{1}H NMR (400 MHz, $CDCl_3$): δ 7.44–7.36 (m, 2H), 7.29 (d, *J* = 8.0 Hz, 2H), 7.18–7.09 (m, 4H), 6.40 (d, *J* = 11.6 Hz, 1H), 5.66 (dt, *J* = 11.6, 7.2 Hz, 1H), 2.48–2.33 (m, 4H), 2.20–2.09 (m, 4H), 1.64–1.57 (m, 2H), 1.53–1.43 (m, 4H),1.41–1.31 (m, 2H), 0.99–0.89 (m, 6H), 0.79–0.71 (m, 6H); ^{13}C NMR (100 MHz, $CDCl_3$): δ 142.2, 140.8, 139.4, 139.0, 136.0, 133.1, 131.4, 128.8, 128.7, 128.6, 122.0, 90.3, 80.7, 32.4, 31.1, 28.7, 28.6, 28.5, 22.6, 22.2, 19.3, 14.1, 13.8, 13.5, 13.4. HRMS (ESI, m/z): calcd for $C_{30}H_{39}$ $[M+H]^+$: 399.3046, found: 399.3047.

10. Supplementary the Single Crystal Data of the Complex of 1a (CCDC: 2015820)

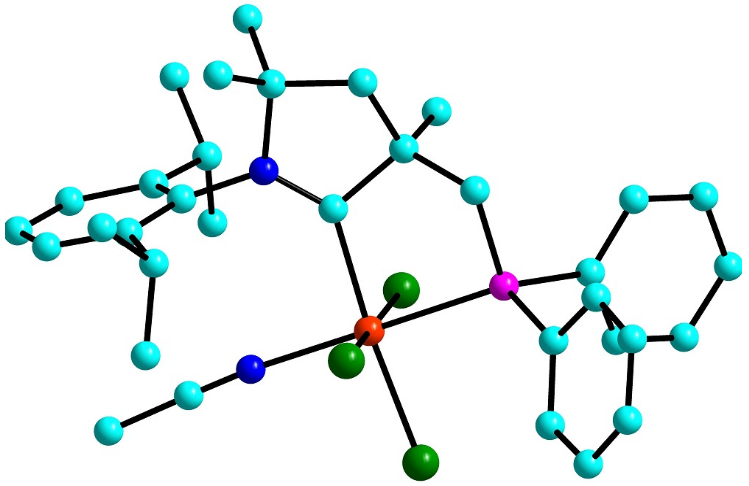

Single crystals of **1a** suitable for X-ray crystallography was obtained by recrystallization from hexane to acetonitrile at –30 °C. The shape of crystals is blue and they stored for a while outside solvent, the solvent molecules in crystals were evaporated resulting in solvent accessible VOID(S) in structures. .

alert level B

Author Response: The reflections might be missing because they are shadowed by the beam-stop due to large unit cell dimensions.

Supplementary Table S9. Crystal Data and Structure Refinement for 1a

Identification code	200604_s2_ll
Empirical formula	$C_{34}H_{43}Cl_3CrN_2P$
Formula weight	669.02
Temperature/K	293.15
Crystal system	orthorhombic
Space group	Pbca
a/Å	14.1900(6)
b/Å	32.7831(19)
c/Å	34.2410(10)
α/°	90
β/°	90
γ/°	90
Volume/Å^3	15928.6(12)
Z	16
ρ_{calc}g/cm^3	1.116
μ/mm^{-1}	0.551
F(000)	5616.0
Crystal size/mm^3	0.35 × 0.3 × 0.05
Radiation	MoKα (λ = 0.71073)
2Θ range for data collection/°	5.862 to 52.746
Index ranges	$-17 \leq h \leq 16$, $-27 \leq k \leq 40$, $-42 \leq l \leq 41$
Reflections collected	43753
Independent reflections	16201 [R_{int} = 0.0327, R_{sigma} = 0.0505]
Data/restraints/parameters	16201/2/755
Goodness-of-fit on F^2	1.049
Final R indexes [I>=2σ (I)]	R_1 = 0.0807, wR_2 = 0.2391
Final R indexes [all data]	R_1 = 0.1303, wR_2 = 0.2818
Largest diff. peak/hole / e Å^{-3}	1.15/-0.55

Supplementary Table S10. Fractional Atomic Coordinates (×10^4) and Equivalent Isotropic Displacement Parameters (Å^2×10^3) for Complex 1a. U_{eq} is defined as 1/3 of of the trace of the orthogonalised U_{IJ} tensor.

**Atom**	***x***	***y***	***z***	**U(eq)**
Cr1	1797.4(5)	5215.9(3)	3265.3(2)	50.9(2)
Cl1	2827.1(10)	5780.8(6)	3283.2(4)	76.6(5)
Cl2	555.4(9)	5587.0(4)	3514.3(3)	60.3(3)
Cl3	2941.7(11)	4834.1(6)	2960.2(4)	80.1(5)
P1	1131.2(9)	5439.7(5)	2639.3(3)	51.4(3)
N1	393(3)	4466.2(14)	3435.4(11)	56.1(10)
N2	2244(3)	5045.0(15)	3823.5(11)	59.0(11)
C1	778(3)	4734.1(15)	3204.6(13)	48.4(11)
C2	298(4)	4693.7(17)	2800.7(14)	59.0(13)
C3	-609(4)	4461(2)	2883.0(16)	74.6(17)
C4	-443(4)	4223.9(19)	3259.1(16)	69.1(15)
C5	108(3)	5109.2(17)	2609.9(14)	58.7(13)
C6	949(5)	4438(2)	2533.3(17)	80.1(18)
C7	-1303(5)	4245(2)	3521.8(19)	90(2)
C8	-145(6)	3773(2)	3190(2)	95(2)
C9	709(4)	4353.7(18)	3825.2(14)	61.2(14)
C10	243(4)	4503.3(17)	4159.3(14)	58.4(13)
C11	494(5)	4338(2)	4518.2(16)	71.9(16)
C12	1174(5)	4042(2)	4558.2(18)	82.1(19)
C13	1633(5)	3910(2)	4240.4(19)	80.3(18)
C14	1427(5)	4058.3(19)	3860.4(18)	71.6(15)
C15	-417(4)	4863.8(18)	4166.2(15)	65.0(14)
C16	6(5)	5223(2)	4385.3(16)	80.9(18)
C17	-1363(5)	4770(2)	4365.2(19)	90(2)
C18	2060(6)	3900(2)	3528.7(19)	90(2)
C19	3076(5)	4030(3)	3591(2)	113(3)
C20	2001(8)	3435(3)	3488(3)	144(4)
C21	606(3)	5945.0(18)	2591.2(13)	55.7(13)
C22	988(5)	6285(2)	2775.4(17)	77.0(17)
C23	575(6)	6659(2)	2732(2)	88(2)
C24	-236(5)	6701(2)	2519.5(18)	81.3(18)
C25	-607(4)	6379(2)	2338.1(18)	75.6(17)
C26	-205(4)	5997(2)	2366.5(15)	64.6(15)
C27	1834(3)	5414.3(18)	2198.0(13)	54.9(13)
C28	2659(4)	5639.4(19)	2188.9(14)	64.5(15)

C29	3203(4)	5660(2)	1859.6(16)	74.9(18)
C30	2935(4)	5459(2)	1525.7(16)	78.7(19)
C31	2122(5)	5223(2)	1525.8(16)	81.8(19)
C32	1572(4)	5198(2)	1862.4(14)	68.1(15)
C33	2496(4)	4982.5(17)	4128.7(14)	58.2(13)
C34	2840(4)	4906(2)	4524.7(15)	77.1(18)
Cr2	6186.7(6)	6456.2(2)	4672.4(2)	48.9(2)
Cl4	5925.4(12)	6180.9(6)	5296.9(4)	85.5(5)
Cl5	5005.4(11)	6936.8(5)	4732.1(5)	74.3(4)
Cl6	7287.6(10)	5968.2(5)	4521.5(5)	78.0(4)
P2	4922.0(9)	6060.1(4)	4364.8(3)	50.0(3)
N3	6759(3)	7022.6(13)	3950.6(11)	57.0(11)
N4	7198(4)	6818.2(16)	4922.9(13)	64.3(13)
C35	6317(3)	6713.5(15)	4100.2(12)	46.9(11)
C36	5726(4)	6520.2(17)	3778.0(13)	60.0(14)
C37	5677(5)	6838(2)	3462.5(17)	85(2)
C38	6503(5)	7120(2)	3520.9(16)	81.8(19)
C39	4803(4)	6349.7(19)	3913.3(16)	72.2(16)
C40	6312(5)	6150(2)	3622.8(18)	80.4(18)
C41	6218(6)	7560(2)	3465(2)	114(3)
C42	7347(6)	7036(3)	3261.3(18)	111(3)
C43	7488(4)	7277.2(17)	4137.2(13)	59.9(13)
C44	7250(5)	7645.0(18)	4307.3(16)	69.9(15)
C45	7997(6)	7890(2)	4433(2)	91(2)
C46	8915(6)	7780(3)	4391(2)	107(3)
C47	9136(5)	7400(2)	4238(2)	88(2)
C48	8435(4)	7143.0(19)	4115.5(16)	68.2(15)
C49	6279(5)	7803(2)	4395.4(19)	85.4(19)
C50	6126(6)	7838(2)	4846(2)	114(3)
C51	6068(6)	8224(2)	4212(3)	114(3)
C52	8778(4)	6727(2)	3990.2(19)	80.3(18)
C53	9286(5)	6503(2)	4328(2)	102(2)
C54	9452(6)	6746(3)	3639(3)	123(3)
C55	3720(4)	6064.9(16)	4557.6(16)	58.7(13)
C56	3571(4)	6065.1(17)	4953.8(16)	65.7(14)
C57	2654(5)	6048.3(19)	5096(2)	80.6(18)
C58	1911(5)	6036(2)	4842(3)	87(2)
C59	2062(5)	6028(2)	4459(2)	86(2)
C60	2954(4)	6043(2)	4308.2(19)	73.8(16)

C61	5076(4)	5518.6(16)	4265.4(14)	54.7(12)
C62	5521(4)	5282.1(18)	4540.0(17)	67.8(14)
C63	5614(5)	4867.4(19)	4482(2)	85.2(19)
C64	5244(5)	4692(2)	4151(2)	87(2)
C65	4814(5)	4919(2)	3873(2)	76.1(17)
C66	4719(4)	5334.7(18)	3925.9(16)	63.6(14)
C67	7667(8)	6968(4)	5106(3)	158(4)
C68	8385(12)	7248(6)	5373(5)	308(13)

Supplementary Table S11. Anisotropic Displacement Parameters (Å^2×10^3) for Complex 1a. The Anisotropic displacement factor exponent takes the form: -2π^2[h^{2a} *^{2}U$_{11}$+2hka*b*U$_{12}$+…].

Atom	U_{11}	U_{22}	U_{33}	U_{23}	U_{13}	U_{12}
Cr1	47.5(4)	73.5(6)	31.7(4)	5.6(3)	-2.3(3)	-6.3(4)
Cl1	70.4(8)	105.9(13)	53.5(7)	15.1(7)	-13.4(6)	-33.1(8)
Cl2	64.8(7)	73.8(9)	42.3(6)	5.9(6)	5.1(5)	3.2(7)
Cl3	70.0(8)	115.5(14)	54.9(8)	7.8(8)	10.4(6)	19.9(9)
P1	50.3(7)	70.8(9)	33.1(6)	5.8(6)	-3.2(5)	-8.8(6)
N1	66(3)	62(3)	40(2)	8.3(19)	-1.7(19)	-9(2)
N2	53(2)	82(3)	43(2)	6(2)	-4.1(18)	-3(2)
C1	50(2)	55(3)	41(2)	-5(2)	-6(2)	10(2)
C2	72(3)	64(3)	42(2)	0(2)	-10(2)	-11(3)
C3	85(4)	78(4)	61(3)	14(3)	-22(3)	-28(3)
C4	79(4)	67(4)	61(3)	9(3)	-12(3)	-23(3)
C5	53(3)	79(4)	44(2)	7(2)	-8(2)	-12(3)
C6	106(5)	81(4)	54(3)	-10(3)	-6(3)	14(4)
C7	85(4)	107(6)	76(4)	22(4)	-10(3)	-37(4)
C8	133(6)	69(4)	84(4)	-1(4)	-15(4)	-21(4)
C9	68(3)	66(3)	50(3)	14(2)	-6(2)	-12(3)
C10	62(3)	65(3)	47(3)	12(2)	0(2)	-6(3)
C11	88(4)	79(4)	48(3)	12(3)	1(3)	-15(4)
C12	102(5)	84(5)	60(4)	26(3)	-17(3)	-8(4)
C13	97(5)	70(4)	74(4)	23(3)	-14(4)	10(4)
C14	81(4)	62(4)	71(4)	5(3)	-6(3)	9(3)
C15	72(3)	77(4)	47(3)	16(3)	7(2)	-1(3)
C16	109(5)	85(5)	48(3)	4(3)	12(3)	-12(4)
C17	87(4)	115(6)	70(4)	20(4)	26(3)	-7(4)
C18	120(6)	80(5)	70(4)	5(3)	-9(4)	23(4)
C19	88(5)	148(8)	102(6)	30(5)	10(4)	45(5)

C20	208(11)	84(6)	140(8)	-18(6)	-15(7)	51(7)
C21	55(3)	76(4)	36(2)	10(2)	8(2)	-5(3)
C22	82(4)	86(5)	63(3)	1(3)	-6(3)	-11(4)
C23	126(6)	65(4)	74(4)	-3(3)	11(4)	-10(4)
C24	106(5)	76(4)	62(3)	9(3)	9(4)	14(4)
C25	70(4)	86(5)	71(4)	23(3)	3(3)	0(4)
C26	55(3)	83(4)	56(3)	16(3)	1(2)	-7(3)
C27	52(3)	78(4)	34(2)	7(2)	-1(2)	-2(3)
C28	58(3)	93(4)	42(3)	12(3)	-5(2)	1(3)
C29	53(3)	114(5)	57(3)	14(3)	4(3)	-1(3)
C30	69(4)	118(6)	48(3)	9(3)	11(3)	18(4)
C31	93(4)	112(6)	40(3)	-11(3)	-1(3)	13(4)
C32	70(3)	90(4)	44(3)	1(3)	0(2)	-6(3)
C33	56(3)	73(4)	46(3)	5(2)	0(2)	-3(3)
C34	87(4)	100(5)	44(3)	18(3)	-14(3)	-7(4)
Cr2	60.9(5)	49.1(5)	36.7(4)	1.4(3)	-5.8(3)	-2.7(4)
Cl4	97.1(11)	110.1(13)	49.4(7)	26.8(8)	-14.5(7)	-24.3(10)
Cl5	82.3(9)	58.6(8)	81.9(10)	-11.4(7)	16.4(8)	5.7(7)
Cl6	63.5(8)	64.2(9)	106.3(12)	-3.7(8)	-9.7(8)	6.5(7)
P2	58.7(7)	48.7(7)	42.7(6)	2.6(5)	-5.6(5)	-2.1(6)
N3	77(3)	55(3)	39(2)	4.3(18)	-6.5(19)	-11(2)
N4	77(3)	64(3)	52(3)	-6(2)	-23(2)	-23(3)
C35	58(3)	46(3)	37(2)	-4(2)	2(2)	3(2)
C36	74(3)	71(4)	35(2)	-1(2)	-5(2)	-15(3)
C37	111(5)	92(5)	54(3)	18(3)	-21(3)	-34(4)
C38	113(5)	87(5)	46(3)	20(3)	-19(3)	-33(4)
C39	86(4)	74(4)	57(3)	14(3)	-19(3)	-8(3)
C40	92(4)	83(5)	66(4)	-19(3)	8(3)	-3(4)
C41	155(7)	96(6)	92(5)	54(4)	-47(5)	-28(5)
C42	146(7)	139(7)	49(3)	13(4)	-2(4)	-50(6)
C43	83(4)	55(3)	41(2)	7(2)	-10(2)	-13(3)
C44	93(4)	54(3)	62(3)	3(3)	-15(3)	-13(3)
C45	126(6)	61(4)	86(5)	-12(3)	-22(4)	-10(4)
C46	109(6)	105(6)	105(6)	-2(5)	-37(5)	-40(5)
C47	88(4)	95(5)	81(4)	-9(4)	-10(3)	-20(4)
C48	83(4)	67(4)	54(3)	-2(3)	-3(3)	-19(3)
C49	115(5)	60(4)	82(4)	2(3)	-12(4)	-1(4)
C50	154(8)	77(5)	111(6)	-25(4)	14(5)	-1(5)
C51	137(7)	63(4)	144(7)	16(5)	-11(6)	6(5)

C52	75(4)	81(4)	84(4)	-11(4)	10(3)	-19(4)
C53	82(5)	106(6)	118(6)	18(5)	11(4)	2(4)
C54	101(6)	154(9)	114(6)	-28(6)	40(5)	-29(6)
C55	57(3)	52(3)	68(3)	1(3)	-3(2)	6(3)
C56	77(3)	56(3)	64(3)	5(3)	4(3)	-2(3)
C57	82(4)	69(4)	91(4)	1(3)	24(4)	-7(3)
C58	64(4)	64(4)	134(7)	-8(4)	24(4)	-5(3)
C59	61(4)	82(5)	115(6)	-11(4)	-4(4)	-5(3)
C60	65(4)	80(4)	76(4)	-5(3)	-7(3)	5(3)
C61	61(3)	51(3)	52(3)	-5(2)	11(2)	-10(2)
C62	78(4)	57(3)	68(3)	6(3)	-5(3)	-6(3)
C63	103(5)	51(4)	101(5)	12(3)	6(4)	1(4)
C64	103(5)	49(4)	109(5)	-16(4)	28(4)	-11(4)
C65	83(4)	64(4)	80(4)	-22(3)	21(3)	-17(3)
C66	57(3)	74(4)	60(3)	-14(3)	5(2)	-10(3)
C67	155(10)	154(11)	165(11)	39(9)	30(9)	9(9)
C68	243(17)	320(20)	360(30)	110(20)	-182(18)	-158(18)

Supplementary Table S12. Bond Lengths for Complex 1a

Atom	Atom	Length/Å	Atom	Atom	Length/Å
Cr1	Cl1	2.3599(17)	Cr2	Cl4	2.3505(15)
Cr1	Cl2	2.3051(15)	Cr2	Cl5	2.3095(17)
Cr1	Cl3	2.3009(17)	Cr2	Cl6	2.2948(17)
Cr1	P1	2.4548(13)	Cr2	P2	2.4528(15)
Cr1	N2	2.090(4)	Cr2	N4	2.050(4)
Cr1	C1	2.152(5)	Cr2	C35	2.141(4)
P1	C5	1.815(5)	P2	C39	1.822(5)
P1	C21	1.824(6)	P2	C55	1.829(5)
P1	C27	1.812(5)	P2	C61	1.821(5)
N1	C1	1.302(6)	N3	C35	1.297(6)
N1	C4	1.550(7)	N3	C38	1.549(6)
N1	C9	1.456(6)	N3	C43	1.475(7)
N2	C33	1.123(6)	N4	C67	1.040(11)
C1	C2	1.548(6)	C35	C36	1.524(6)
C2	C3	1.523(8)	C36	C37	1.503(8)
C2	C5	1.535(7)	C36	C39	1.498(8)
C2	C6	1.548(8)	C36	C40	1.564(8)
C3	C4	1.522(7)	C37	C38	1.506(9)
C4	C7	1.518(9)	C38	C41	1.509(10)

C4	C8	1.556(9)	C38	C42	1.517(11)
C9	C10	1.410(7)	C43	C44	1.381(8)
C9	C14	1.411(8)	C43	C48	1.416(8)
C10	C11	1.390(7)	C44	C45	1.398(9)
C10	C15	1.508(8)	C44	C49	1.503(9)
C11	C12	1.375(9)	C45	C46	1.361(11)
C12	C13	1.339(9)	C46	C47	1.387(11)
C13	C14	1.419(8)	C47	C48	1.369(8)
C14	C18	1.537(9)	C48	C52	1.511(9)
C15	C16	1.519(8)	C49	C50	1.561(10)
C15	C17	1.537(8)	C49	C51	1.546(9)
C18	C19	1.519(11)	C52	C53	1.547(10)
C18	C20	1.536(11)	C52	C54	1.539(9)
C21	C22	1.391(8)	C55	C56	1.373(7)
C21	C26	1.395(7)	C55	C60	1.384(8)
C22	C23	1.367(9)	C56	C57	1.390(8)
C23	C24	1.367(10)	C57	C58	1.366(10)
C24	C25	1.332(9)	C58	C59	1.330(10)
C25	C26	1.380(8)	C59	C60	1.368(9)
C27	C28	1.384(7)	C61	C62	1.373(8)
C27	C32	1.400(7)	C61	C66	1.404(7)
C28	C29	1.368(7)	C62	C63	1.380(8)
C29	C30	1.373(9)	C63	C64	1.374(10)
C30	C31	1.389(9)	C64	C65	1.355(10)
C31	C32	1.394(8)	C65	C66	1.381(8)
C33	C34	1.463(7)	C67	C68	1.646(14)

Supplementary Table S13. Bond Angles for Complex 1a.

Atom	Atom	Atom	Angle/°	Atom	Atom	Atom	Angle/°
Cl1	Cr1	P1	91.52(5)	Cl4	Cr2	P2	94.13(6)
Cl2	Cr1	Cl1	92.84(6)	Cl5	Cr2	Cl4	93.83(7)
Cl2	Cr1	P1	82.58(5)	Cl5	Cr2	P2	82.42(6)
Cl3	Cr1	Cl1	90.10(7)	Cl6	Cr2	Cl4	92.55(7)
Cl3	Cr1	Cl2	174.05(6)	Cl6	Cr2	Cl5	171.71(7)
Cl3	Cr1	P1	92.18(5)	Cl6	Cr2	P2	91.86(6)
N2	Cr1	Cl1	89.93(13)	N4	Cr2	Cl4	87.25(14)
N2	Cr1	Cl2	92.01(13)	N4	Cr2	Cl5	94.37(17)
N2	Cr1	Cl3	93.17(13)	N4	Cr2	Cl6	91.22(17)
N2	Cr1	P1	174.46(13)	N4	Cr2	P2	176.58(16)

N2	Cr1	C1	95.48(17)	N4	Cr2	C35	95.43(19)
C1	Cr1	Cl1	174.16(13)	C35	Cr2	Cl4	175.84(14)
C1	Cr1	Cl2	84.78(13)	C35	Cr2	Cl5	82.81(13)
C1	Cr1	Cl3	91.80(13)	C35	Cr2	Cl6	90.58(13)
C1	Cr1	P1	82.89(12)	C35	Cr2	P2	83.03(13)
C5	P1	Cr1	100.26(16)	C39	P2	Cr2	99.00(19)
C5	P1	C21	102.2(3)	C39	P2	C55	102.4(3)
C21	P1	Cr1	120.50(16)	C55	P2	Cr2	121.53(18)
C27	P1	Cr1	120.17(16)	C61	P2	Cr2	120.57(18)
C27	P1	C5	111.5(2)	C61	P2	C39	111.1(3)
C27	P1	C21	101.0(2)	C61	P2	C55	100.8(2)
C1	N1	C4	115.5(4)	C35	N3	C38	115.0(4)
C1	N1	C9	126.8(4)	C35	N3	C43	127.6(4)
C9	N1	C4	117.6(4)	C43	N3	C38	117.3(4)
C33	N2	Cr1	174.9(5)	C67	N4	Cr2	167.2(8)
N1	C1	Cr1	135.9(3)	N3	C35	Cr2	135.3(3)
N1	C1	C2	107.5(4)	N3	C35	C36	107.8(4)
C2	C1	Cr1	116.4(3)	C36	C35	Cr2	116.8(3)
C1	C2	C6	108.2(4)	C35	C36	C40	106.1(4)
C3	C2	C1	104.5(4)	C37	C36	C35	104.9(4)
C3	C2	C5	112.0(5)	C37	C36	C40	108.6(5)
C3	C2	C6	110.0(5)	C39	C36	C35	114.3(4)
C5	C2	C1	112.4(4)	C39	C36	C37	116.2(5)
C5	C2	C6	109.5(4)	C39	C36	C40	106.3(5)
C4	C3	C2	106.3(4)	C36	C37	C38	107.2(5)
N1	C4	C8	109.8(5)	C37	C38	N3	100.4(4)
C3	C4	N1	100.8(4)	C37	C38	C41	111.2(7)
C3	C4	C8	113.5(5)	C37	C38	C42	115.1(6)
C7	C4	N1	111.2(5)	C41	C38	N3	112.4(5)
C7	C4	C3	110.7(5)	C41	C38	C42	108.1(6)
C7	C4	C8	110.6(5)	C42	C38	N3	109.5(6)
C2	C5	P1	111.5(3)	C36	C39	P2	112.1(4)
C10	C9	N1	120.7(5)	C44	C43	N3	120.3(5)
C10	C9	C14	120.6(5)	C44	C43	C48	121.7(5)
C14	C9	N1	118.4(5)	C48	C43	N3	117.9(5)
C9	C10	C15	125.2(4)	C43	C44	C45	116.5(6)
C11	C10	C9	117.4(5)	C43	C44	C49	127.6(6)
C11	C10	C15	116.9(5)	C45	C44	C49	115.8(6)
C12	C11	C10	122.9(6)	C46	C45	C44	122.7(7)

C13	C12	C11	119.1(6)	C45	C46	C47	119.7(7)
C12	C13	C14	122.4(6)	C48	C47	C46	120.2(7)
C9	C14	C13	117.5(6)	C43	C48	C52	126.9(5)
C9	C14	C18	126.1(5)	C47	C48	C43	118.9(6)
C13	C14	C18	116.3(6)	C47	C48	C52	114.1(6)
C10	C15	C16	111.7(5)	C44	C49	C50	110.5(6)
C10	C15	C17	113.2(5)	C44	C49	C51	113.8(6)
C16	C15	C17	106.3(5)	C51	C49	C50	108.0(6)
C19	C18	C14	110.8(6)	C48	C52	C53	111.5(6)
C19	C18	C20	110.0(7)	C48	C52	C54	112.6(6)
C20	C18	C14	111.8(7)	C54	C52	C53	108.3(6)
C22	C21	P1	121.9(4)	C56	C55	P2	120.0(4)
C22	C21	C26	118.2(6)	C56	C55	C60	119.2(5)
C26	C21	P1	119.9(5)	C60	C55	P2	120.6(4)
C23	C22	C21	120.2(6)	C55	C56	C57	119.3(6)
C22	C23	C24	120.6(7)	C58	C57	C56	120.1(6)
C25	C24	C23	120.1(7)	C59	C58	C57	120.2(6)
C24	C25	C26	121.5(6)	C58	C59	C60	121.4(7)
C25	C26	C21	119.4(6)	C59	C60	C55	119.7(6)
C28	C27	P1	117.4(4)	C62	C61	P2	118.5(4)
C28	C27	C32	118.4(5)	C62	C61	C66	119.4(5)
C32	C27	P1	124.2(4)	C66	C61	P2	122.0(4)
C29	C28	C27	121.4(5)	C61	C62	C63	120.1(6)
C28	C29	C30	120.4(6)	C64	C63	C62	119.7(7)
C29	C30	C31	119.8(5)	C65	C64	C63	121.4(6)
C30	C31	C32	119.8(6)	C64	C65	C66	119.6(6)
C31	C32	C27	120.1(6)	C65	C66	C61	119.9(6)
N2	C33	C34	178.9(7)	N4	C67	C68	174.2(13)

Supplementary Table S14. Torsion Angles for Complex 1a.

A	B	C	D	Angle/°	A	B	C	D	Angle/°
Cr1	P1	C5	C2	-32.8(4)	Cr2	P2	C39	C36	33.5(5)
Cr1	P1	C21	C22	38.3(5)	Cr2	P2	C55	C56	-39.0(5)
Cr1	P1	C21	C26	-141.3(3)	Cr2	P2	C55	C60	145.0(4)
Cr1	P1	C27	C28	-60.5(5)	Cr2	P2	C61	C62	40.5(5)
Cr1	P1	C27	C32	122.6(4)	Cr2	P2	C61	C66	-142.2(4)
Cr1	C1	C2	C3	-158.6(4)	Cr2	C35	C36	C37	160.1(4)
Cr1	C1	C2	C5	-36.9(5)	Cr2	C35	C36	C39	31.7(6)
Cr1	C1	C2	C6	84.2(5)	Cr2	C35	C36	C40	-85.1(5)

P1	C21	C22	C23	179.9(5)	P2	C55	C56	C57	-176.8(5)
P1	C21	C26	C25	178.7(4)	P2	C55	C60	C59	177.0(5)
P1	C27	C28	C29	-175.8(5)	P2	C61	C62	C63	177.2(5)
P1	C27	C32	C31	174.9(5)	P2	C61	C66	C65	-176.8(4)
N1	C1	C2	C3	17.7(6)	N3	C35	C36	C37	-16.6(6)
N1	C1	C2	C5	139.4(5)	N3	C35	C36	C39	-145.0(5)
N1	C1	C2	C6	-99.5(5)	N3	C35	C36	C40	98.2(5)
N1	C9	C10	C11	170.9(5)	N3	C43	C44	C45	-172.1(5)
N1	C9	C10	C15	-17.2(8)	N3	C43	C44	C49	11.8(9)
N1	C9	C14	C13	-171.2(5)	N3	C43	C48	C47	171.0(5)
N1	C9	C14	C18	13.1(9)	N3	C43	C48	C52	-12.8(8)
C1	N1	C4	C3	-10.0(6)	C35	N3	C38	C37	8.8(7)
C1	N1	C4	C7	-127.4(5)	C35	N3	C38	C41	127.1(6)
C1	N1	C4	C8	109.9(5)	C35	N3	C38	C42	-112.7(6)
C1	N1	C9	C10	102.5(6)	C35	N3	C43	C44	-97.5(7)
C1	N1	C9	C14	-84.1(7)	C35	N3	C43	C48	86.0(7)
C1	C2	C3	C4	-23.7(6)	C35	C36	C37	C38	22.1(7)
C1	C2	C5	P1	46.4(5)	C35	C36	C39	P2	-44.4(6)
C2	C3	C4	N1	20.3(6)	C36	C37	C38	N3	-18.6(7)
C2	C3	C4	C7	138.0(5)	C36	C37	C38	C41	-137.7(6)
C2	C3	C4	C8	-97.0(6)	C36	C37	C38	C42	98.9(7)
C3	C2	C5	P1	163.7(4)	C37	C36	C39	P2	-166.8(4)
C4	N1	C1	Cr1	170.4(4)	C38	N3	C35	Cr2	-171.1(4)
C4	N1	C1	C2	-4.7(6)	C38	N3	C35	C36	4.8(6)
C4	N1	C9	C10	-82.3(6)	C38	N3	C43	C44	84.8(6)
C4	N1	C9	C14	91.1(6)	C38	N3	C43	C48	-91.6(6)
C5	P1	C21	C22	148.0(4)	C39	P2	C55	C56	-147.9(5)
C5	P1	C21	C26	-31.5(4)	C39	P2	C55	C60	36.1(5)
C5	P1	C27	C28	-177.3(4)	C39	P2	C61	C62	155.6(4)
C5	P1	C27	C32	5.8(6)	C39	P2	C61	C66	-27.2(5)
C5	C2	C3	C4	-145.7(5)	C39	C36	C37	C38	149.4(6)
C6	C2	C3	C4	92.2(6)	C40	C36	C37	C38	-90.9(6)
C6	C2	C5	P1	-73.9(5)	C40	C36	C39	P2	72.3(5)
C9	N1	C1	Cr1	-14.3(8)	C43	N3	C35	Cr2	11.2(9)
C9	N1	C1	C2	170.6(5)	C43	N3	C35	C36	-173.0(5)
C9	N1	C4	C3	174.2(5)	C43	N3	C38	C37	-173.2(5)
C9	N1	C4	C7	56.9(7)	C43	N3	C38	C41	-54.9(8)
C9	N1	C4	C8	-65.8(6)	C43	N3	C38	C42	65.3(7)
C9	C10	C11	C12	0.7(9)	C43	C44	C45	C46	0.2(10)

C9 C10 C15 C16 -111.0(6)	C43 C44 C49 C50 113.9(7)
C9 C10 C15 C17 129.1(6)	C43 C44 C49 C51 -124.3(7)
C9 C14 C18 C19 113.1(7)	C43 C48 C52 C53 -116.4(6)
C9 C14 C18 C20 -123.7(8)	C43 C48 C52 C54 121.7(7)
C10 C9 C14 C13 2.2(9)	C44 C43 C48 C47 -5.4(8)
C10 C9 C14 C18 -173.5(6)	C44 C43 C48 C52 170.8(6)
C10 C11 C12 C13 1.2(10)	C44 C45 C46 C47 -3.5(12)
C11 C10 C15 C16 61.0(6)	C45 C44 C49 C50 -62.2(8)
C11 C10 C15 C17 -58.9(7)	C45 C44 C49 C51 59.5(8)
C11 C12 C13 C14 -1.5(10)	C45 C46 C47 C48 2.3(11)
C12 C13 C14 C9 -0.2(10)	C46 C47 C48 C43 2.0(10)
C12 C13 C14 C18 175.9(7)	C46 C47 C48 C52 -174.7(6)
C13 C14 C18 C19 -62.6(8)	C47 C48 C52 C53 60.0(7)
C13 C14 C18 C20 60.6(9)	C47 C48 C52 C54 -61.9(8)
C14 C9 C10 C11 -2.4(8)	C48 C43 C44 C45 4.3(8)
C14 C9 C10 C15 169.5(5)	C48 C43 C44 C49 -171.9(5)
C15 C10 C11 C12 -171.9(6)	C49 C44 C45 C46 176.8(7)
C21 P1 C5 C2 -157.3(4)	C55 P2 C39 C36 158.7(4)
C21 P1 C27 C28 74.8(5)	C55 P2 C61 C62 -96.5(5)
C21 P1 C27 C32 -102.1(5)	C55 P2 C61 C66 80.8(4)
C21 C22 C23 C24 2.4(10)	C55 C56 C57 C58 -0.7(9)
C22 C21 C26 C25 -0.8(7)	C56 C55 C60 C59 0.9(9)
C22 C23 C24 C25 -3.0(10)	C56 C57 C58 C59 2.1(10)
C23 C24 C25 C26 1.7(10)	C57 C58 C59 C60 -1.9(11)
C24 C25 C26 C21 0.3(9)	C58 C59 C60 C55 0.4(11)
C26 C21 C22 C23 -0.5(8)	C60 C55 C56 C57 -0.8(9)
C27 P1 C5 C2 95.5(4)	C61 P2 C39 C36 -94.3(5)
C27 P1 C21 C22 -96.9(4)	C61 P2 C55 C56 97.4(5)
C27 P1 C21 C26 83.6(4)	C61 P2 C55 C60 -78.6(5)
C27 C28 C29 C30 0.7(10)	C61 C62 C63 C64 -1.2(10)
C28 C27 C32 C31 -2.0(9)	C62 C61 C66 C65 0.4(8)
C28 C29 C30 C31 -2.1(10)	C62 C63 C64 C65 2.2(11)
C29 C30 C31 C32 1.3(10)	C63 C64 C65 C66 -1.9(10)
C30 C31 C32 C27 0.7(10)	C64 C65 C66 C61 0.6(9)
C32 C27 C28 C29 1.3(9)	C66 C61 C62 C63 -0.1(9)

Supplementary Table S15. Hydrogen Atom Coordinates (Å×10⁴) and Isotropic Displacement Parameters (Å²×10³) for Complex 1a.

Atom	*x*	*y*	*z*	U(eq)
H3A	-1133	4648	2914	90
H3B	-751	4276	2670	90
H5A	-421	5239	2739	70
H5B	-60	5069	2338	70
H6A	1499	4594	2468	120
H6B	617	4367	2299	120
H6C	1135	4193	2667	120
H7A	-1163	4118	3768	134
H7B	-1819	4105	3400	134
H7C	-1472	4525	3564	134
H8A	412	3766	3031	143
H8B	-645	3630	3060	143
H8C	-19	3645	3437	143
H11	188	4431	4741	86
H12	1315	3935	4803	99
H13	2102	3715	4270	96
H15	-541	4948	3896	78
H16A	-415	5451	4371	121
H16B	600	5295	4270	121
H16C	102	5149	4654	121
H17A	-1661	4545	4236	136
H17B	-1764	5006	4350	136
H17C	-1256	4702	4634	136
H18	1840	4022	3284	108
H19A	3115	4322	3589	169
H19B	3462	3921	3386	169
H19C	3293	3929	3839	169
H20A	2116	3310	3737	216
H20B	2466	3343	3304	216
H20C	1385	3360	3397	216
H22	1526	6258	2928	92
H23	847	6887	2848	106
H24	-528	6954	2502	98
H25	-1149	6413	2189	91
H26	-472	5776	2237	78
H28	2847	5780	2412	77

H29	3758	5810	1862	90
H30	3296	5480	1300	94
H31	1945	5082	1302	98
H32	1032	5038	1864	82
H34A	3059	4630	4544	116
H34B	2337	4949	4708	116
H34C	3348	5090	4582	116
H37A	5090	6989	3481	103
H37B	5710	6711	3207	103
H39A	4547	6174	3712	87
H39B	4362	6572	3954	87
H40A	6912	6245	3530	121
H40B	5977	6021	3413	121
H40C	6406	5957	3830	121
H41A	5672	7618	3621	172
H41B	6076	7608	3195	172
H41C	6727	7735	3544	172
H42a	7887	7178	3361	167
H42B	7215	7129	3001	167
H42C	7472	6748	3257	167
H45	7860	8140	4549	109
H46	9393	7958	4465	128
H47	9763	7319	4218	105
H49	5820	7607	4293	102
H50A	6193	7574	4963	171
H50B	5506	7942	4897	171
H50C	6587	8021	4954	171
H51A	6328	8435	4373	172
H51B	5399	8261	4191	172
H51C	6347	8238	3956	172
H52	8228	6564	3914	96
H53A	9891	6626	4371	153
H53B	9367	6221	4260	153
H53C	8915	6523	4561	153
H54A	9157	6893	3429	185
H54B	9599	6475	3554	185
H54C	10022	6884	3714	185
H56	4078	6076	5125	79
H57	2546	6045	5364	97

H58	1298	6034	4938	105
H59	1550	6011	4290	103
H60	3045	6039	4039	89
H62	5761	5401	4766	81
H63	5925	4708	4665	102
H64	5290	4411	4118	105
H65	4584	4796	3647	91
H66	4420	5492	3737	76
H68A	8636	7465	5216	462
H68B	8892	7081	5467	462
H68C	8046	7361	5590	462

11. Supplementary DFT Computational Study

Geometry optimizations were carried out with the Gaussian 16 package with the B3LYP functional augmented with the D3BJ version of Grimme's empirical dispersion correction.[37-42] The def2-SVP basis set was used for all the atoms. Frequency calculations at the same level of theory were performed to identify the number of imaginary frequencies (zero for local minimum and one for transition states) and provide the thermal corrections of Gibbs free energy. Optimized structures are visualized by the CYL-View program.[43] The reduced forms of the pre-catalysts (coordinated with two THF molecules) was used for geometry optimizations.[2] And two THF molecules were omitted when carrying out the topological steric analysis.

The topological steric maps are generated using the web based SambVca 2.1 program developed by Cavallo *et al.* in 2016.[44] Cr was selected as the center of the sphere. Cr-C bond was selected for *z* axis definition, Cr-N/P bond was selected for *xz*-plane definition. Bondi radii scaled by 1.17 was used as atomic radii, and the Sphere radius was 3.5. The distance of the coordination point from the center of the sphere was 0. The mesh spacing for numerical integration was 0.10. All the H-atoms were included in the calculations.

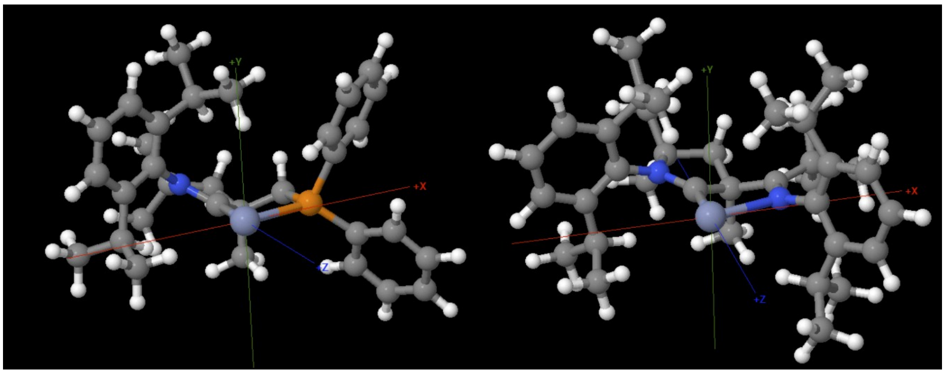

Supplementary Figure S18. Orientation of the steric map of CAAC-Cr(0) from complexes 1a and 1b.

Steric map of CAAC-phosphino-Cr(0) from 1a

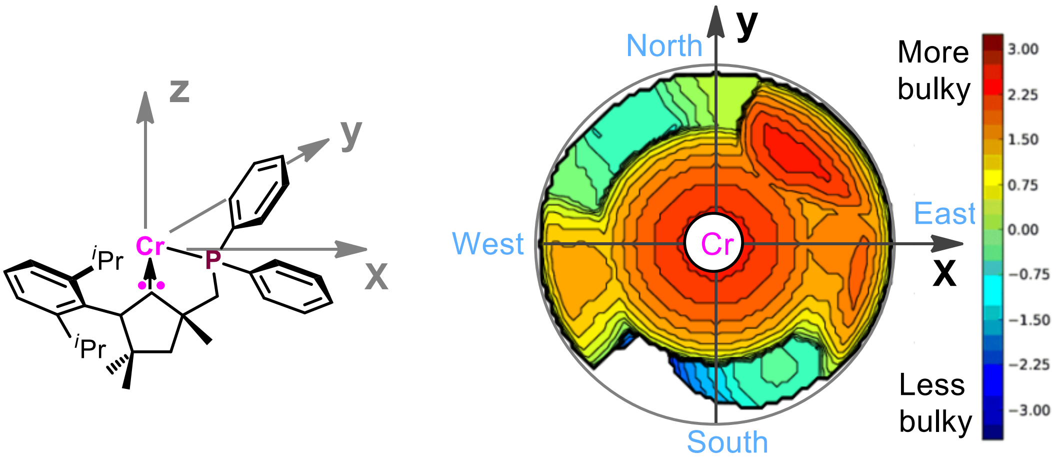

70.9% V_{Bur}

Supplementary Figure S19. Steric map of CAAC-phosphino-Cr(0) from complex 1a. The related complex is oriented according to the scheme on the left; steric map is reported on the right.

Steric map of CAAC-imino-Cr(0) from 1b

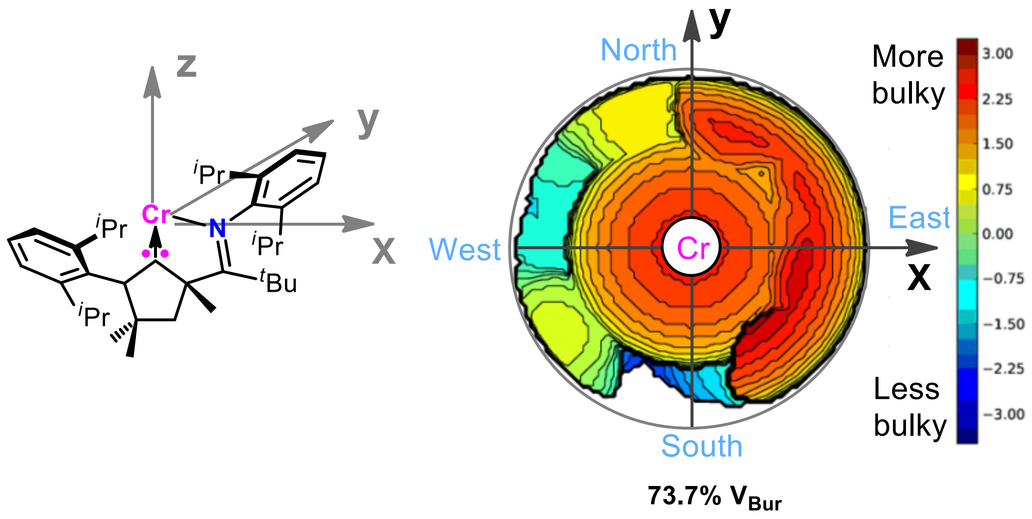

Supplementary Figure S20. Steric map of CAAC-imino-Cr(0) from complex 1b. The related complex is oriented according to the scheme on the left; steric map is reported on the right.

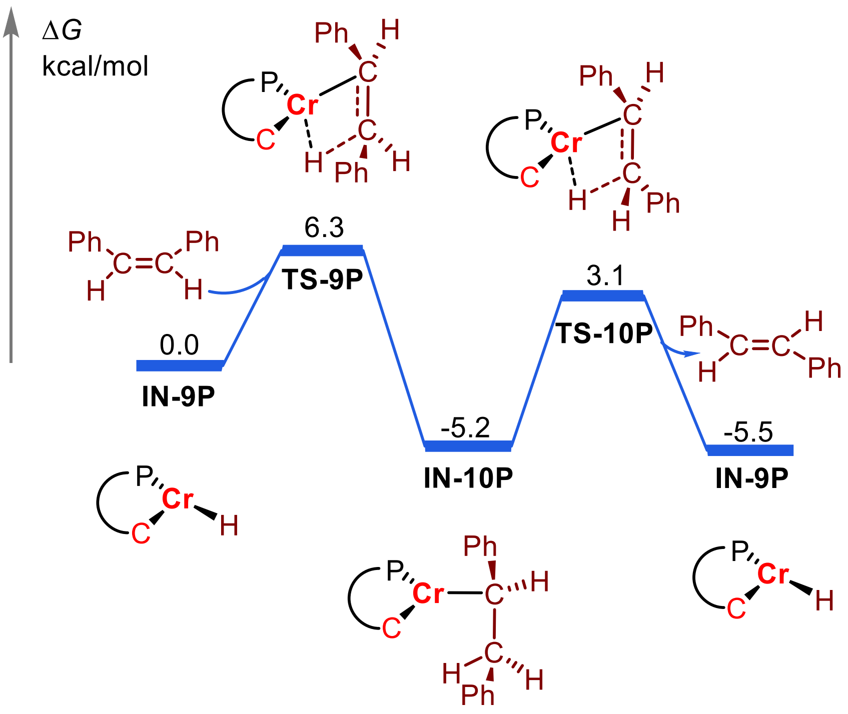

Supplementary Figure S21. DFT Calculations for *cis*-to-*trans* Stereoisomerization with Complex 1a.

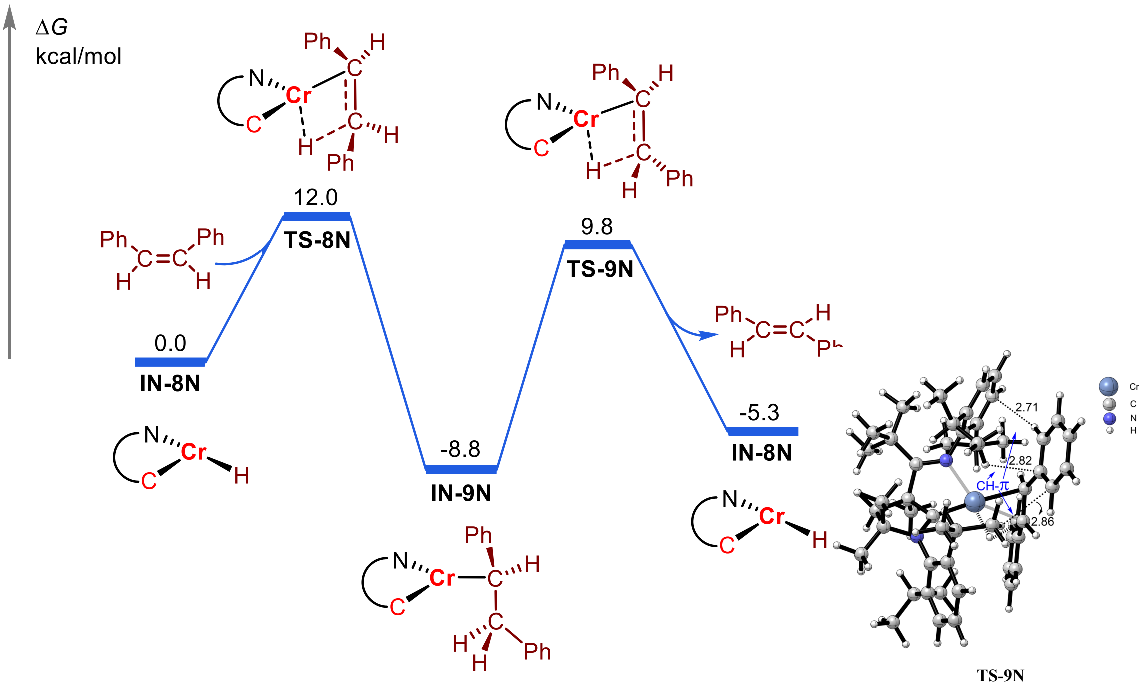

Supplementary Figure S22. DFT Calculations for *cis*-to-*trans* Stereoisomerization with Complex 1b.

Supplementary Table S16. The related parameters for the distance of Cr–C_{olefin} bonds and charge population on the chromated C_{olefin} in the related intermediates and transition states.

Species	Cr-C_{olefin} distance (Å)	Charge on chromated C_{olefin}
**TS-9P**	2.213	-0.1589
**IN-10P**	2.139	-0.2794
**TS-10P**	2.164	-0.1312
**TS-8N**	2.225	-0.1376
**IN-9N**	2.176	-0.1756
**TS-9N**	2.273	-0.0942

12. Supplementary ^{1}H NMR, ^{13}C NMR and ^{19}F NMR Spectra

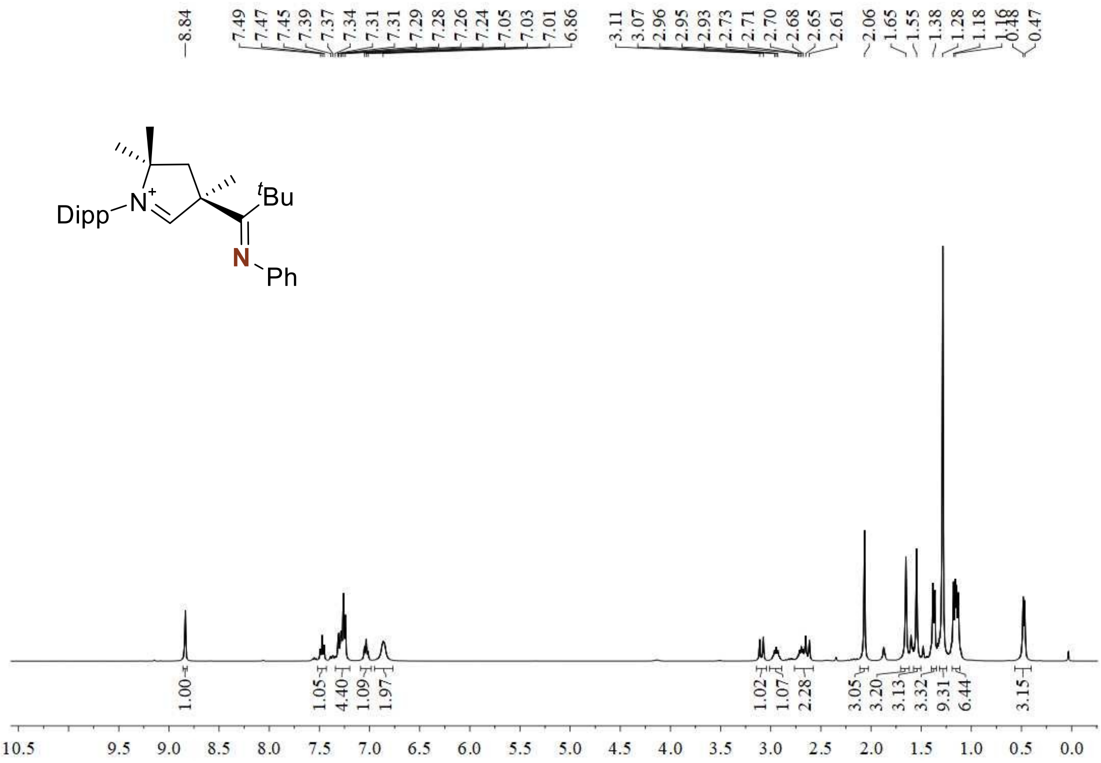

Supplementary Figure S23. ^{1}H NMR spectrum for 16 (400 MHz, $CDCl_3$, 25 °C).

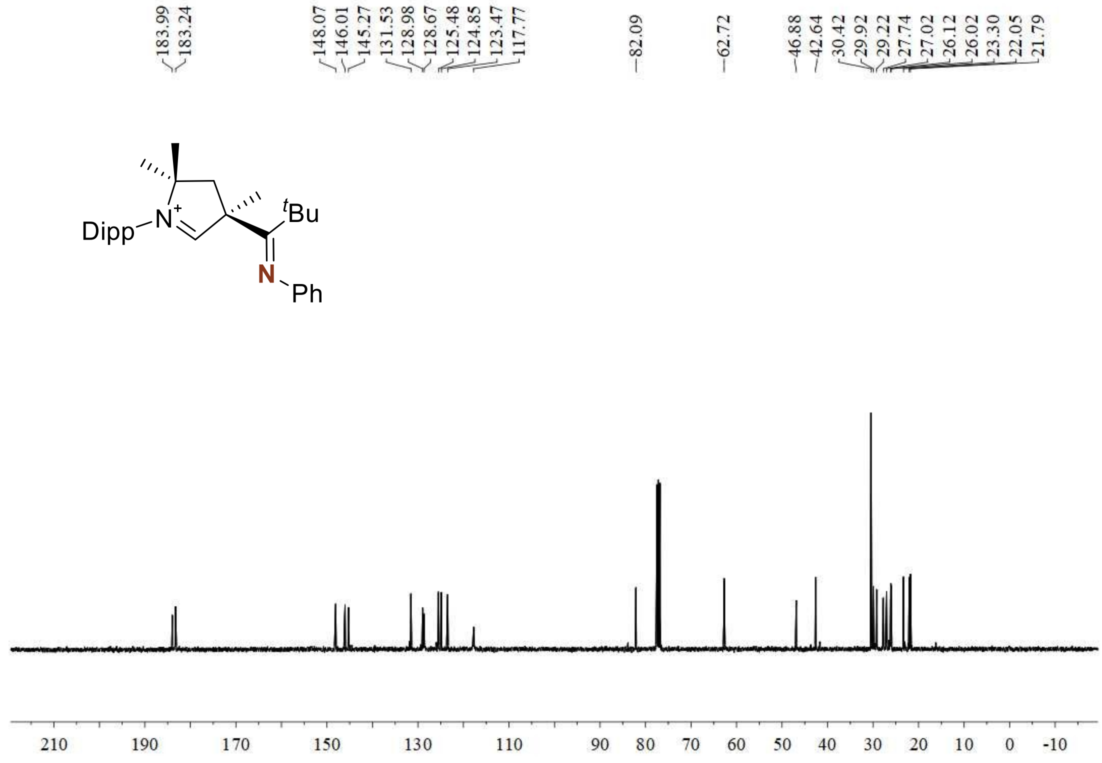

Supplementary Figure S24. ^{13}C NMR spectrum for 16 (100 MHz, $CDCl_3$, 25 °C).

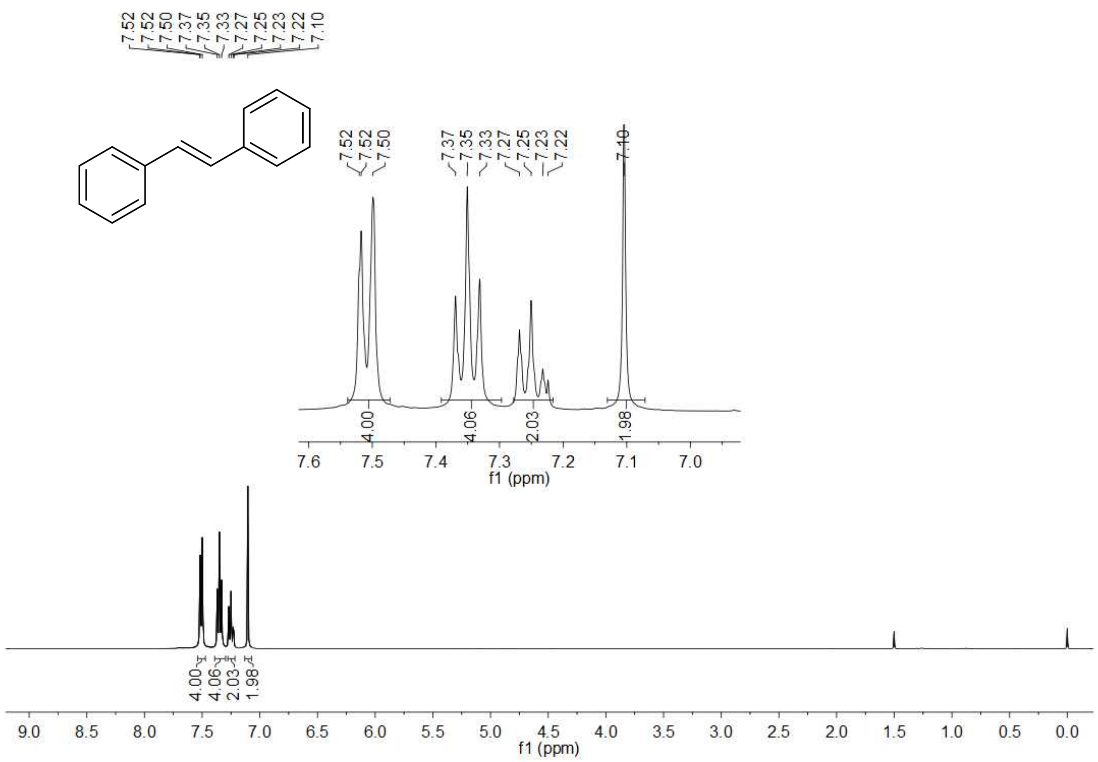

Supplementary Figure S25. ^{1}H NMR spectrum for compound *E*-3a (400 MHz, $CDCl_3$, 25 °C).

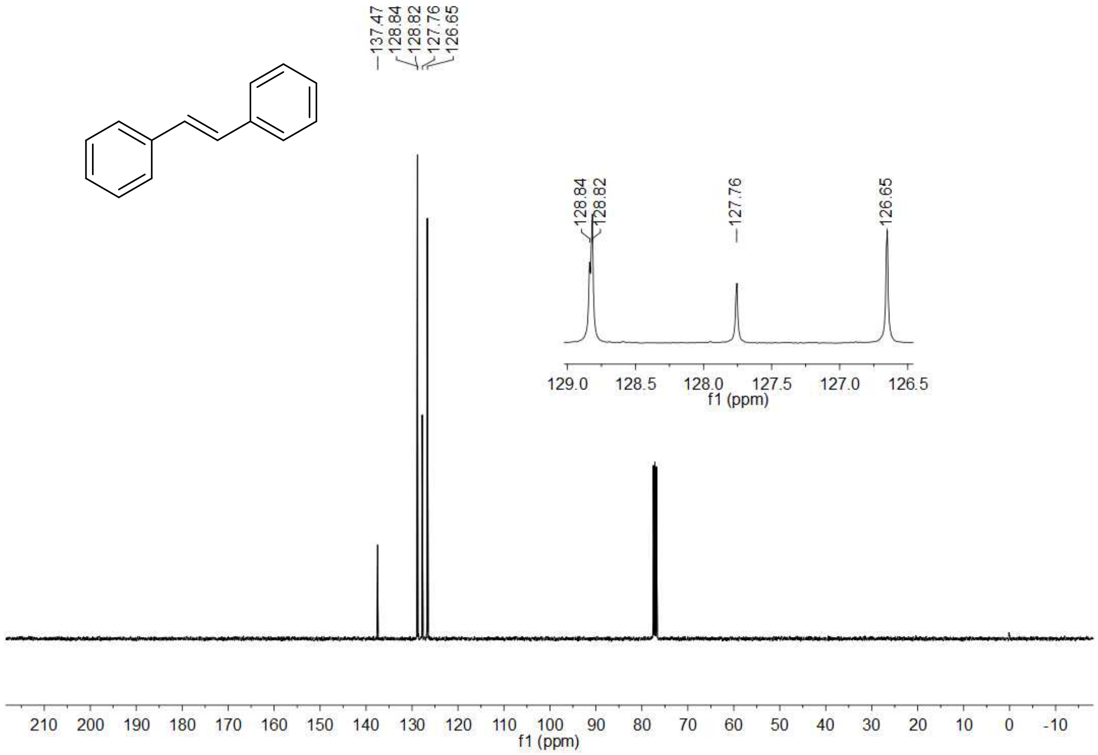

Supplementary Figure S26. ^{13}C NMR spectrum for compound *E*-3a (100 MHz, $CDCl_3$, 25 °C).

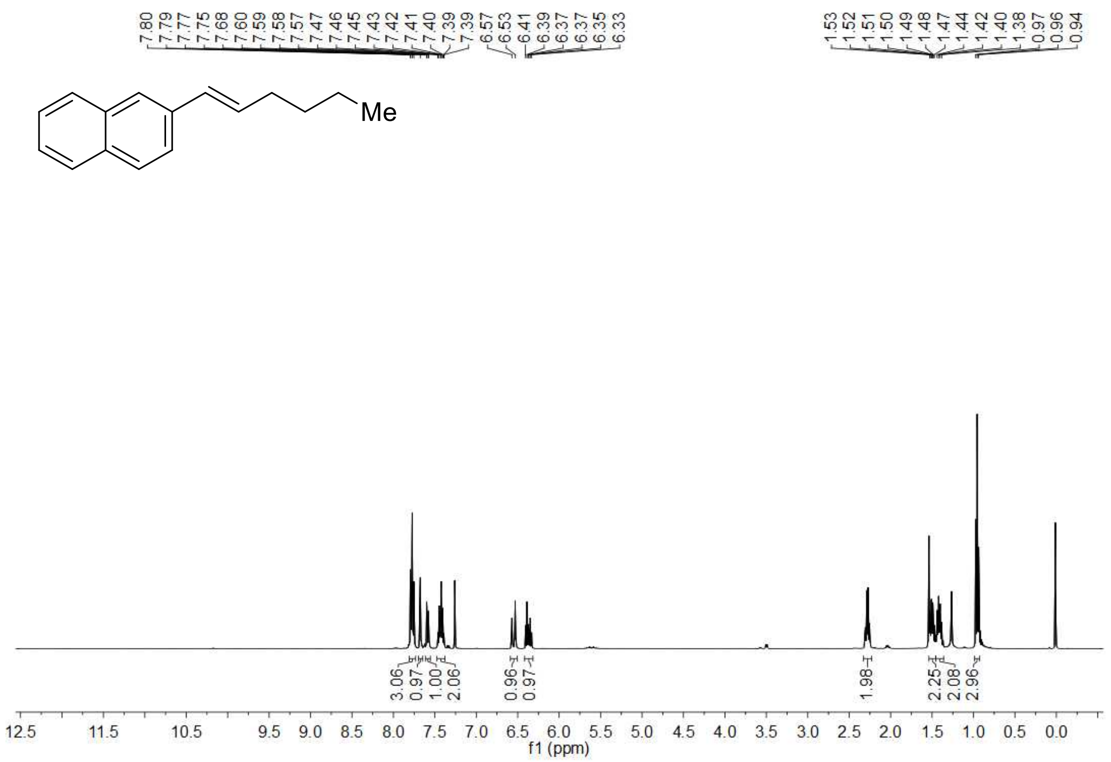

Supplementary Figure S27. ^{1}H NMR spectrum for compound *E*-3b (400 MHz, $CDCl_3$, 25 ^{o}C).

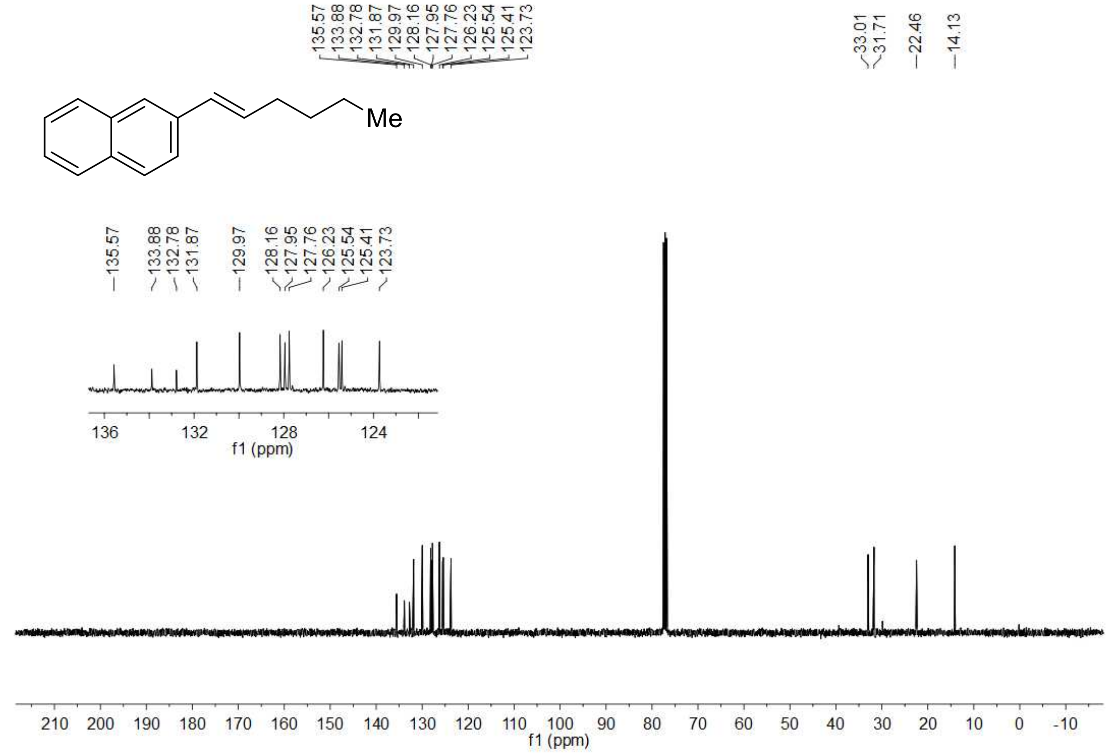

Supplementary Figure S28. ^{13}C NMR spectrum for compound *E*-3b (100 MHz, $CDCl_3$, 25 ^{o}C).

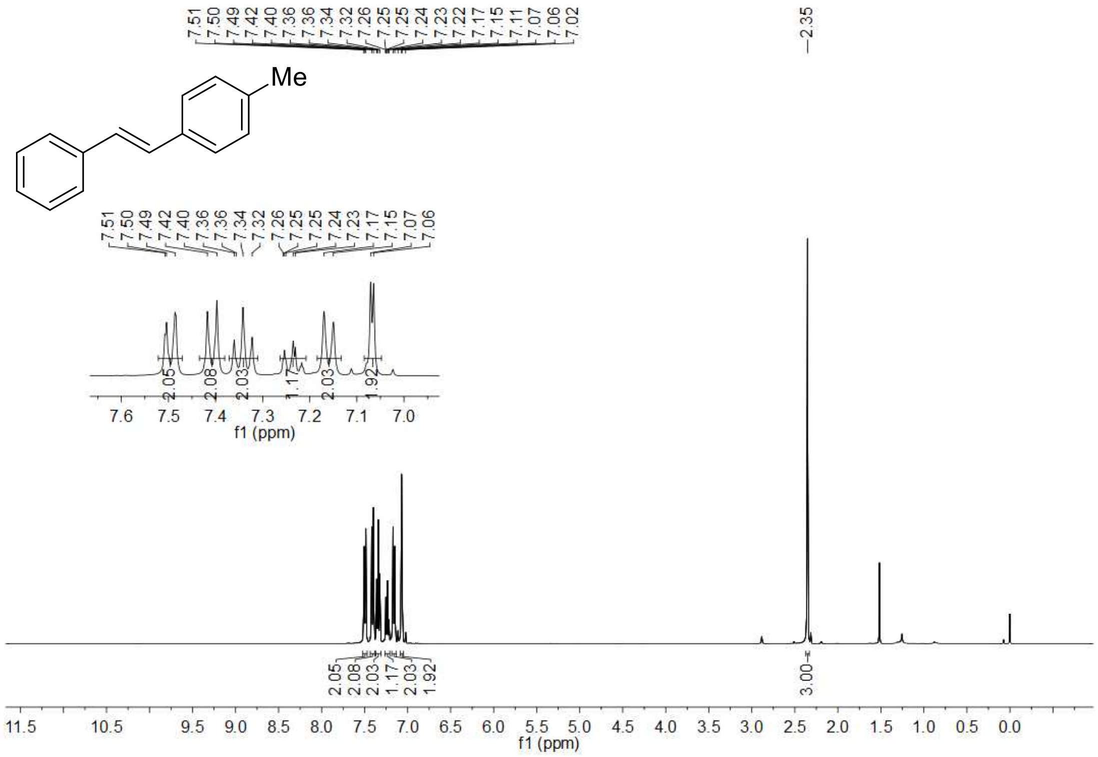

Supplementary Figure S29. ^{1}H NMR spectrum for compound *E*-3c (400 MHz, $CDCl_3$, 25 °C).

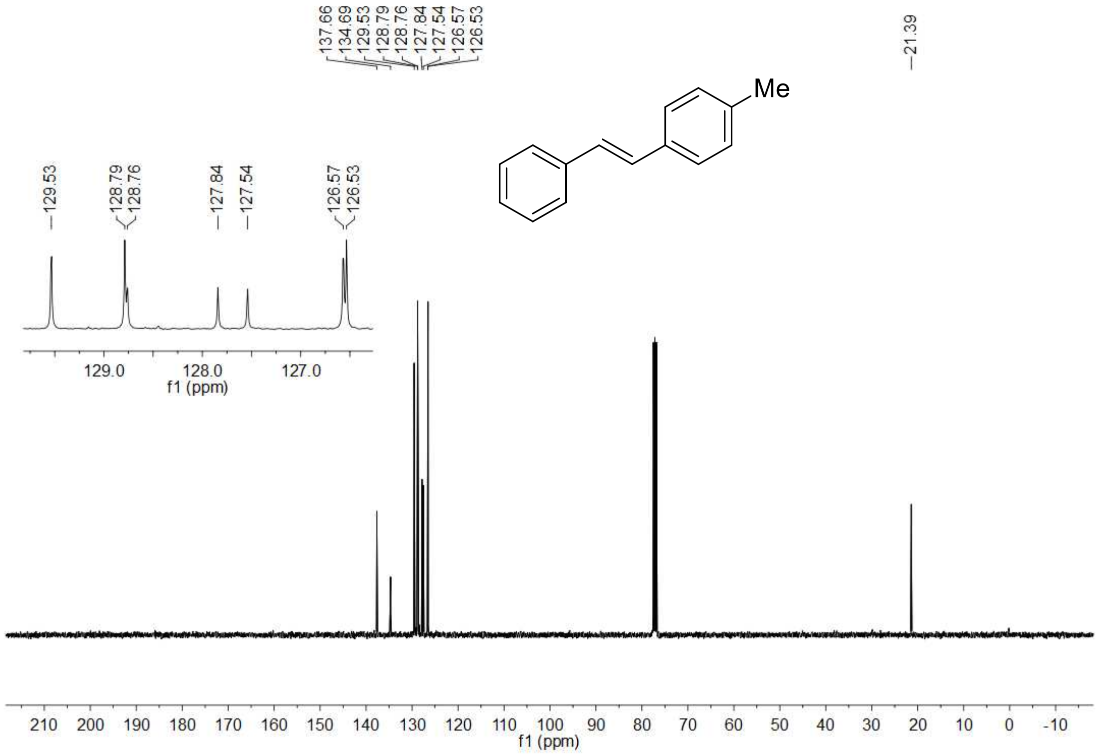

Supplementary Figure S30. ^{13}C NMR spectrum for compound *E*-3c (100 MHz, $CDCl_3$, 25 °C).

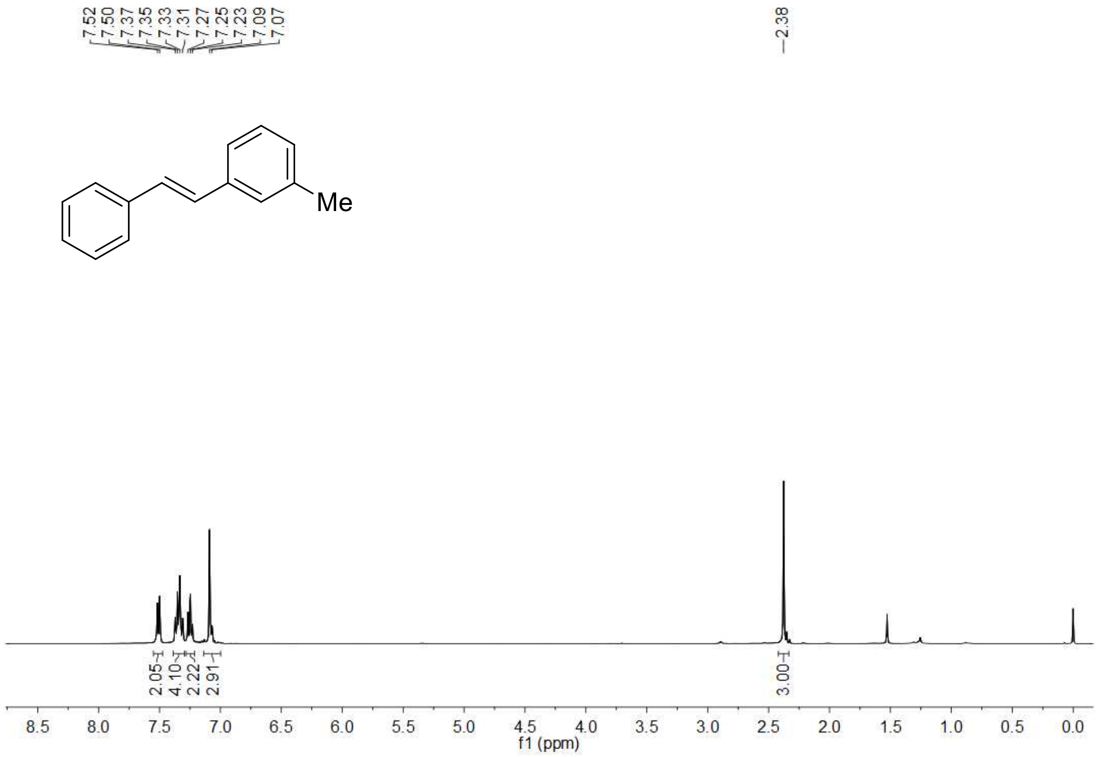

Supplementary Figure S31. **^{1}H NMR spectrum for compound *E*-3d (400 MHz, $CDCl_3$, 25 °C).**

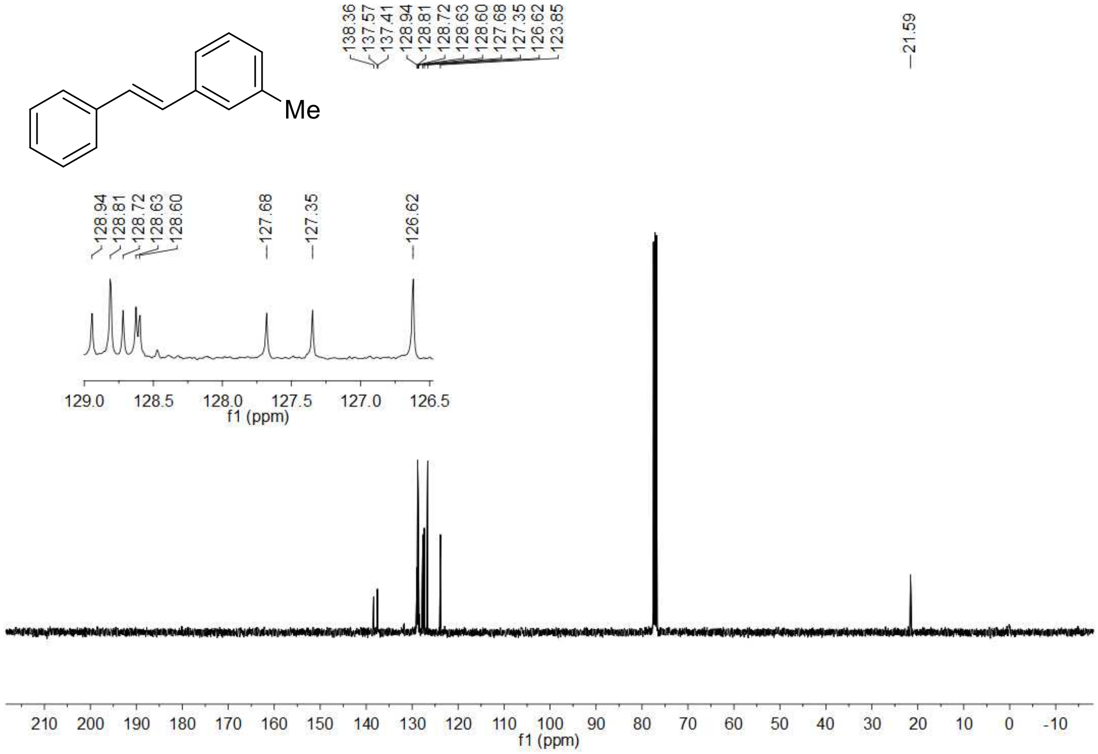

Supplementary Figure S32. **^{13}C NMR spectrum for compound *E*-3d (100 MHz, $CDCl_3$, 25 °C).**

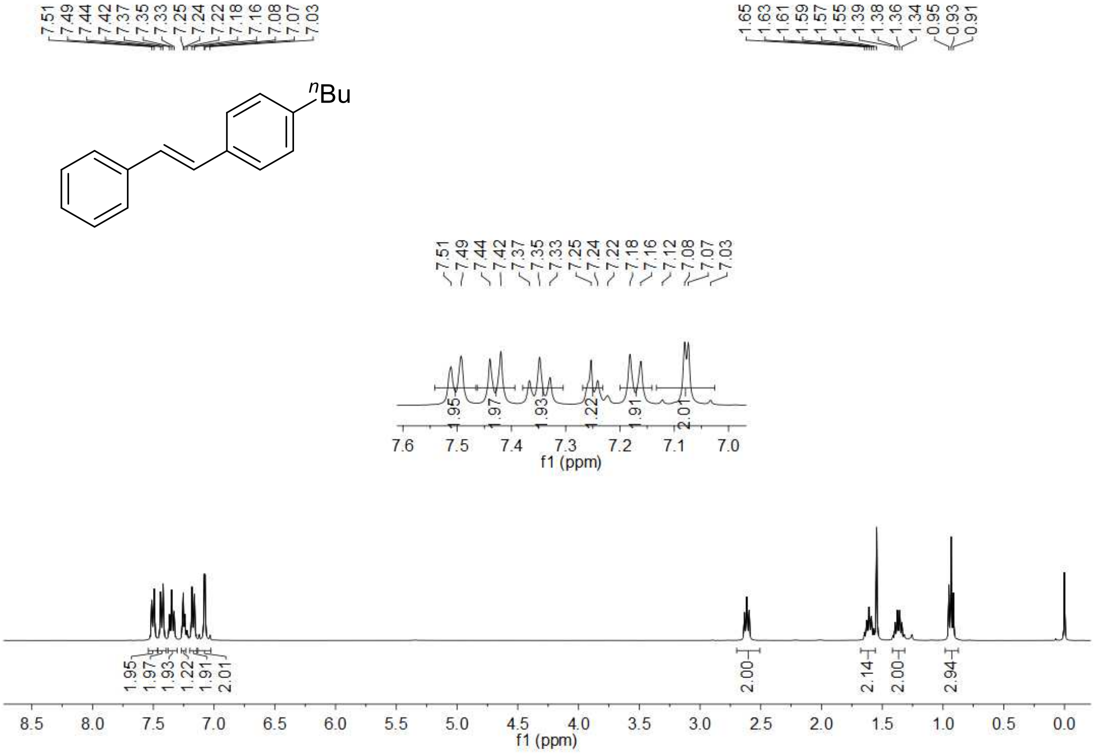

Supplementary Figure S33. **^{1}H NMR spectrum for compound *E*-3e (400 MHz, $CDCl_3$, 25 °C).**

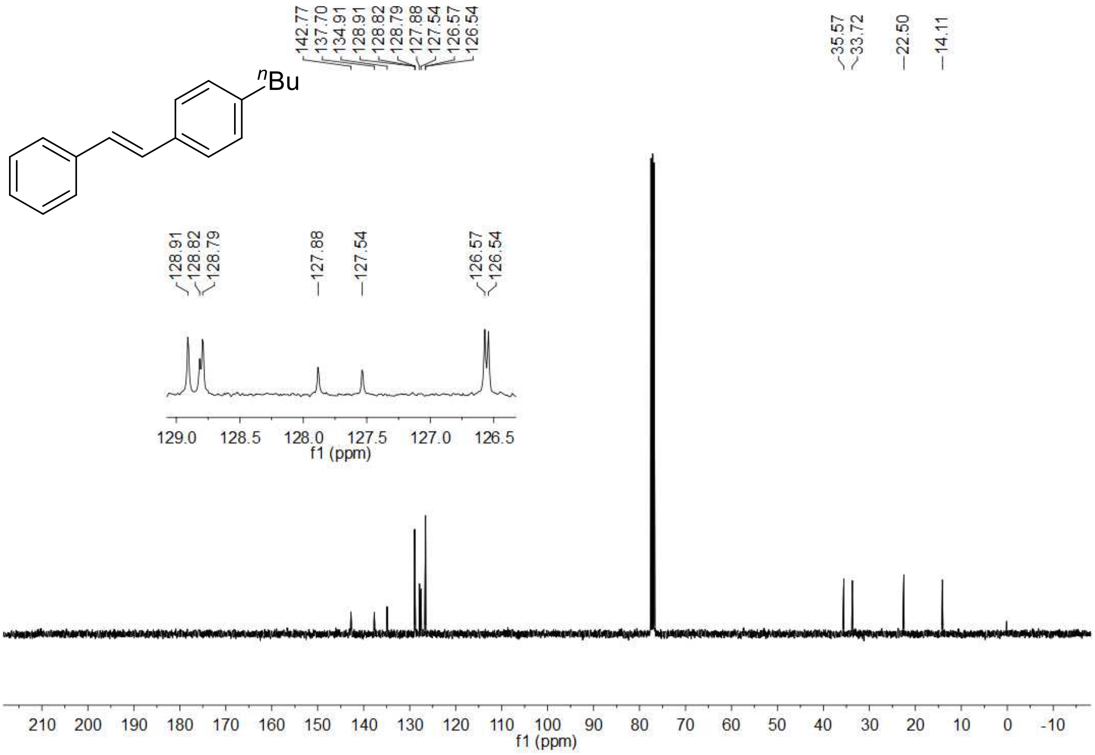

Supplementary Figure S34. **^{13}C NMR spectrum for compound *E*-3e (100 MHz, $CDCl_3$, 25 °C).**

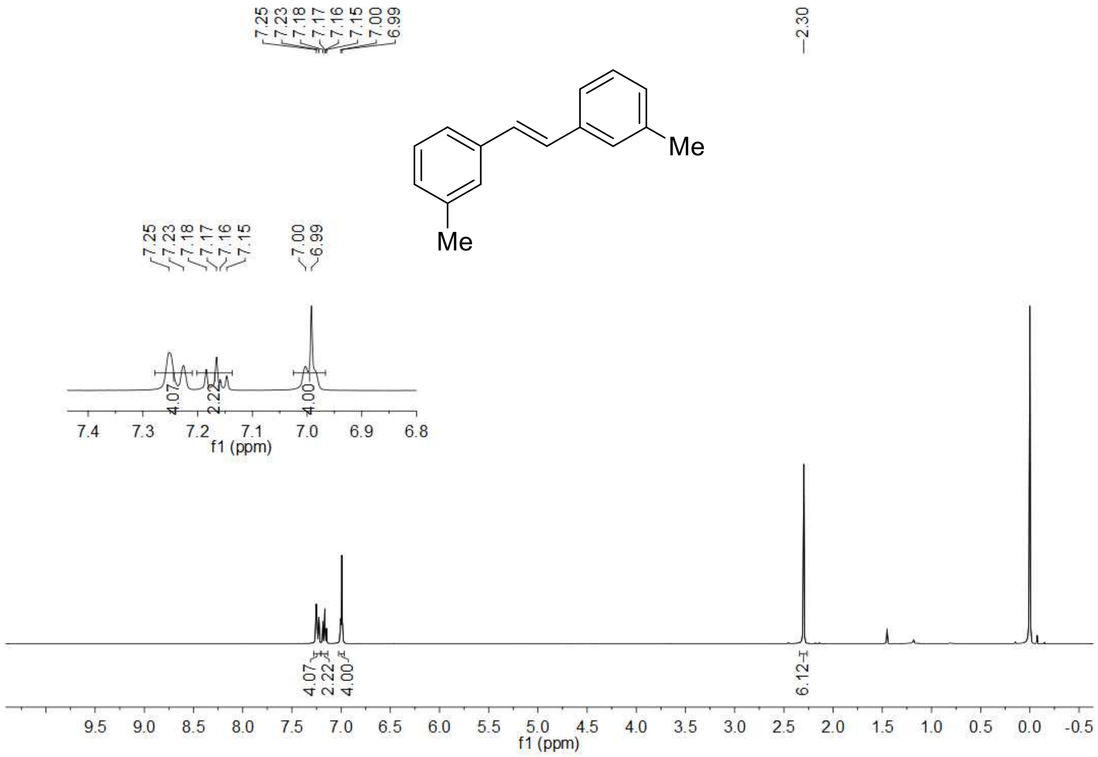

Supplementary Figure S35. ^{1}H NMR spectrum for compound *E*-3f (400 MHz, $CDCl_3$, 25 °C).

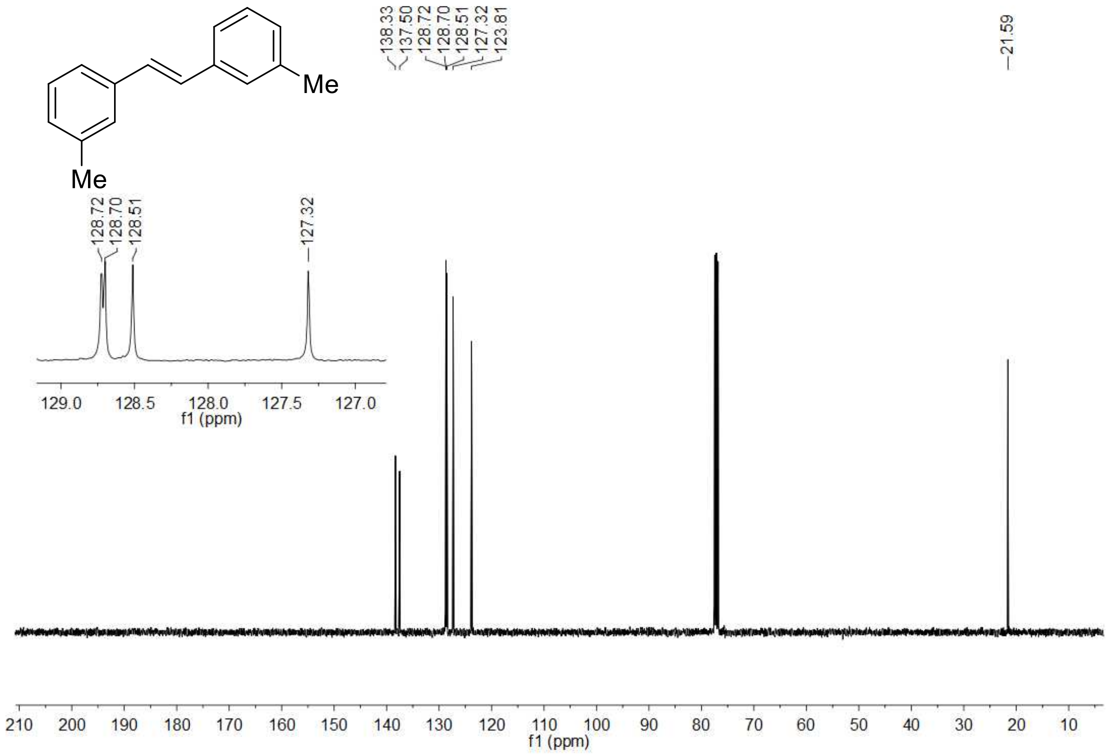

Supplementary Figure S36. ^{13}C NMR spectrum for compound *E*-3f (100 MHz, $CDCl_3$, 25 °C).

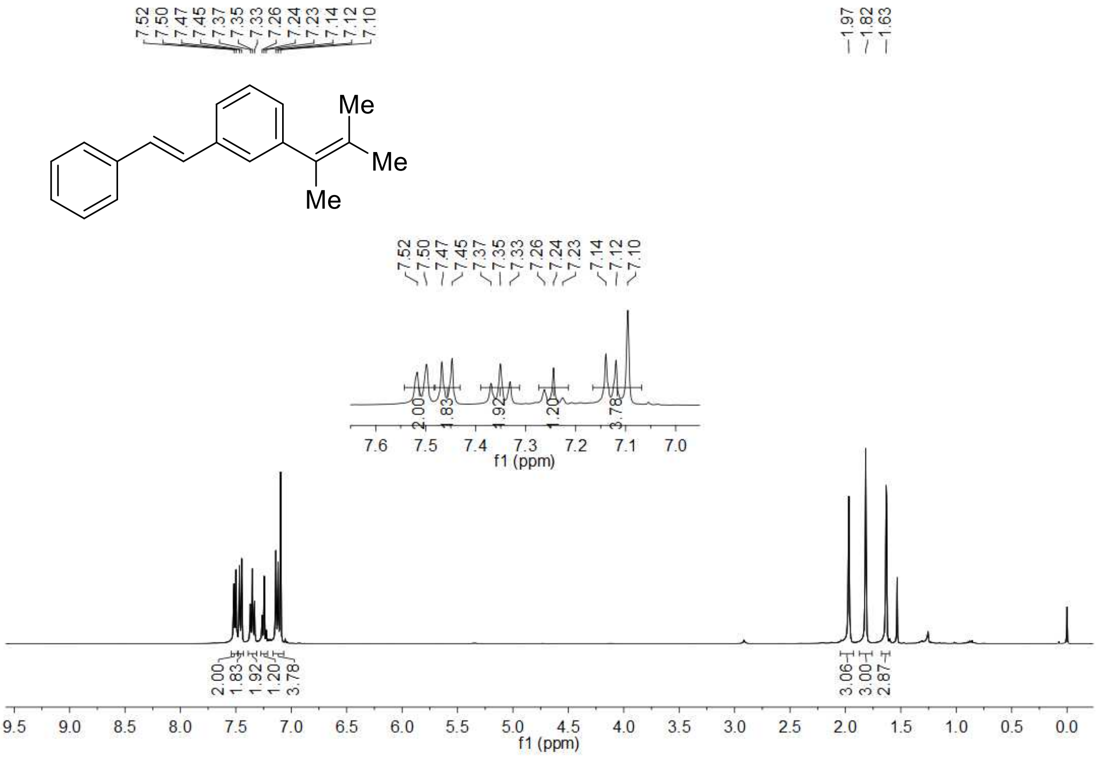

Supplementary Figure S37. ^{1}**H NMR spectrum for compound** ***E*****-3g (400 MHz, CDCl$_3$, 25 °C).**

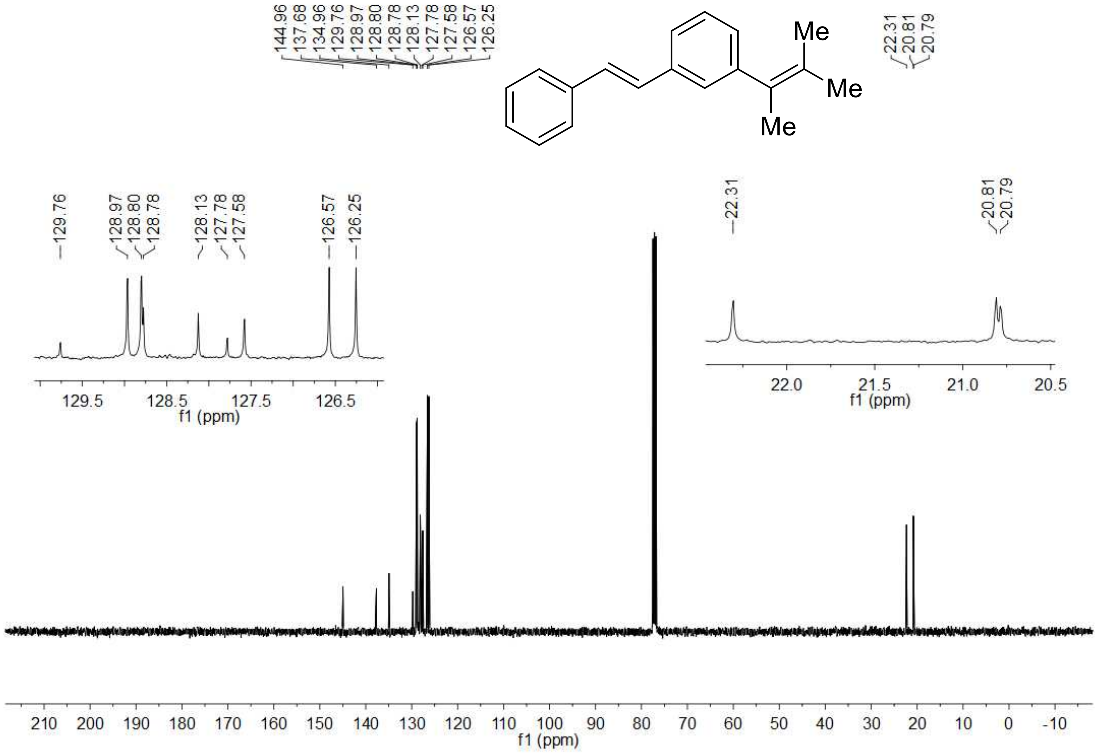

Supplementary Figure S38. ^{13}**C NMR spectrum for compound** ***E*****-3g (100 MHz, CDCl$_3$, 25 °C).**

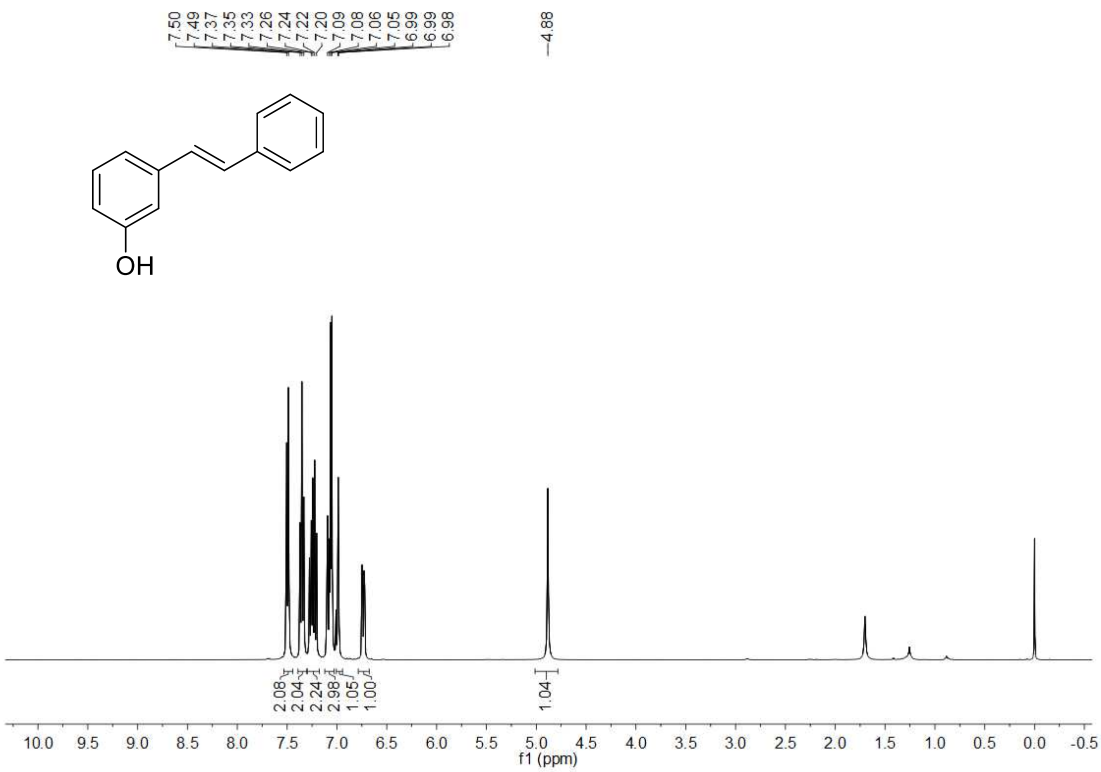

Supplementary Figure S39. ^{1}H NMR spectrum for compound *E*-3h (400 MHz, $CDCl_3$, 25 ^{o}C).

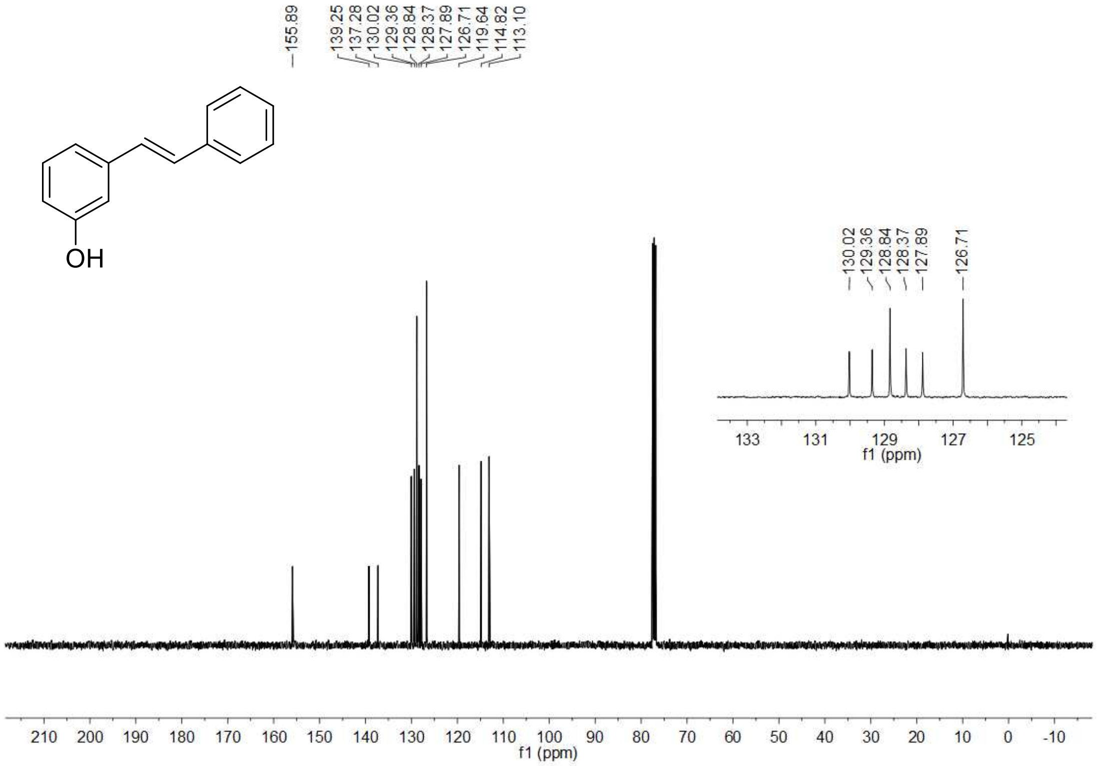

Supplementary Figure S40. ^{13}C NMR spectrum for compound *E*-3h (100 MHz, $CDCl_3$, 25 ^{o}C).

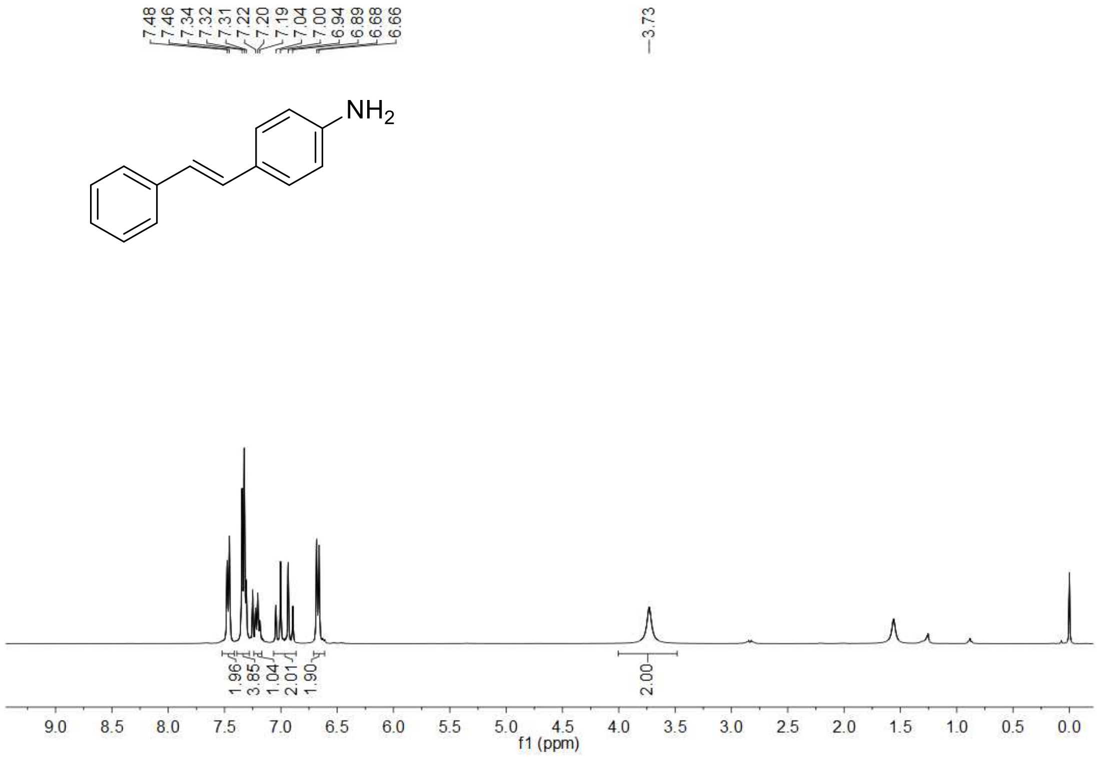

Supplementary Figure S41. ^{1}H NMR spectrum for compound *E*-3i (400 MHz, $CDCl_3$, 25 °C).

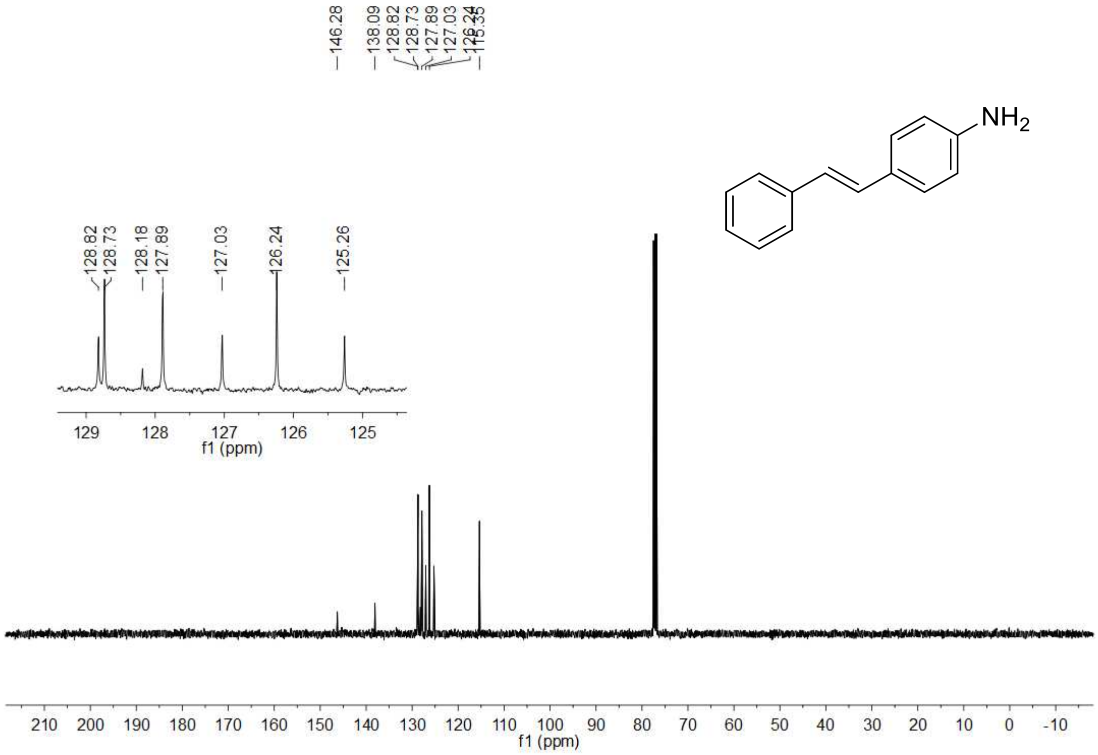

Supplementary Figure S42. ^{13}C NMR spectrum for compound *E*-3i (100 MHz, $CDCl_3$, 25 °C).

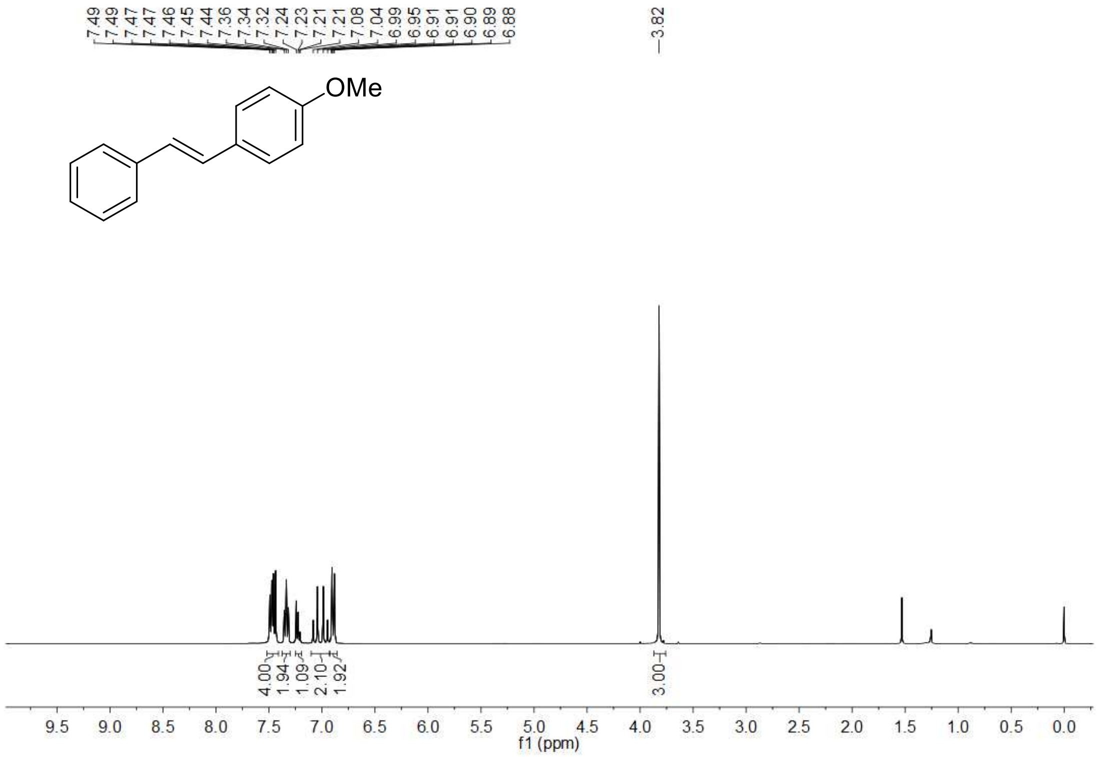

Supplementary Figure S43. ^{1}H NMR spectrum for compound *E*-3j (400 MHz, $CDCl_3$, 25 ºC).

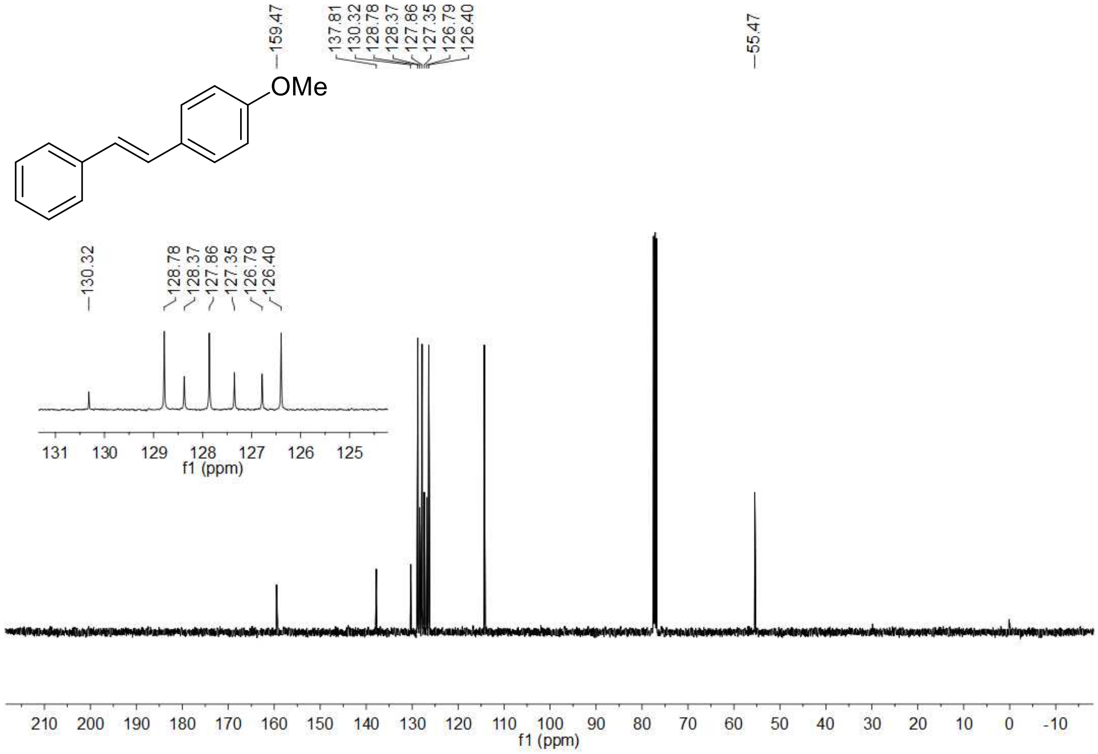

Supplementary Figure S44. ^{13}C NMR spectrum for compound *E*-3j (100 MHz, $CDCl_3$, 25 ºC).

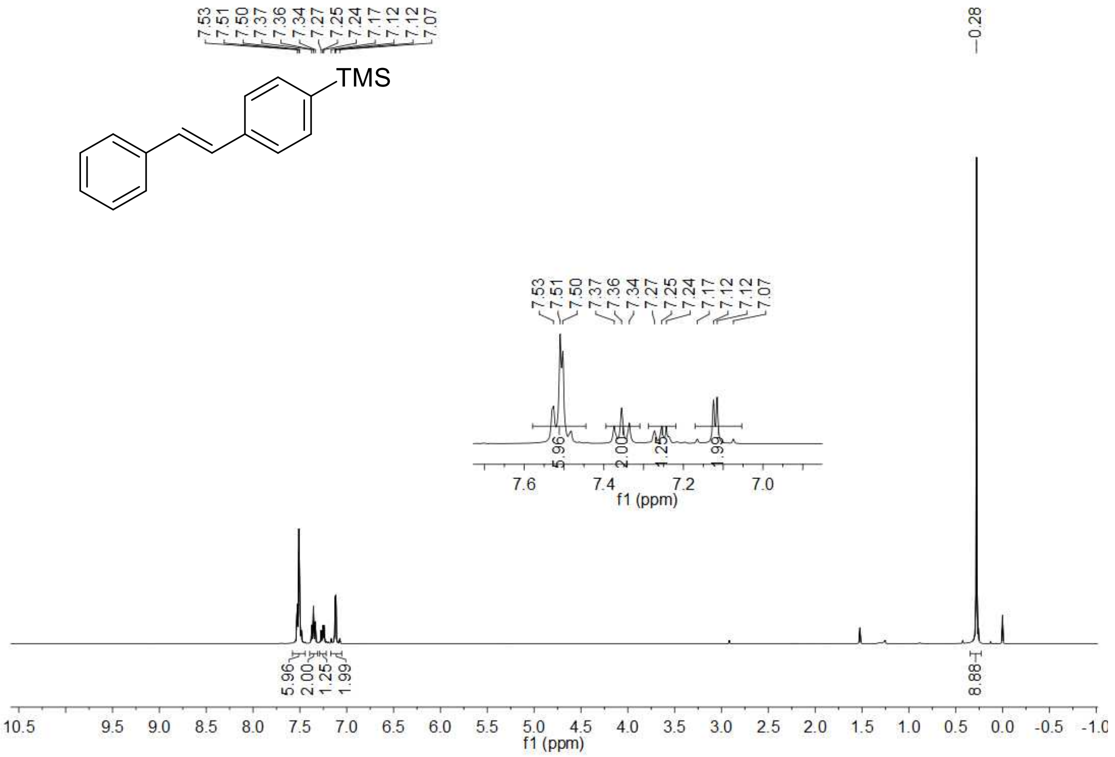

Supplementary Figure S45. ^{1}**H NMR spectrum for compound** ***E*****-3k (400 MHz, $CDCl_3$, 25 °C).**

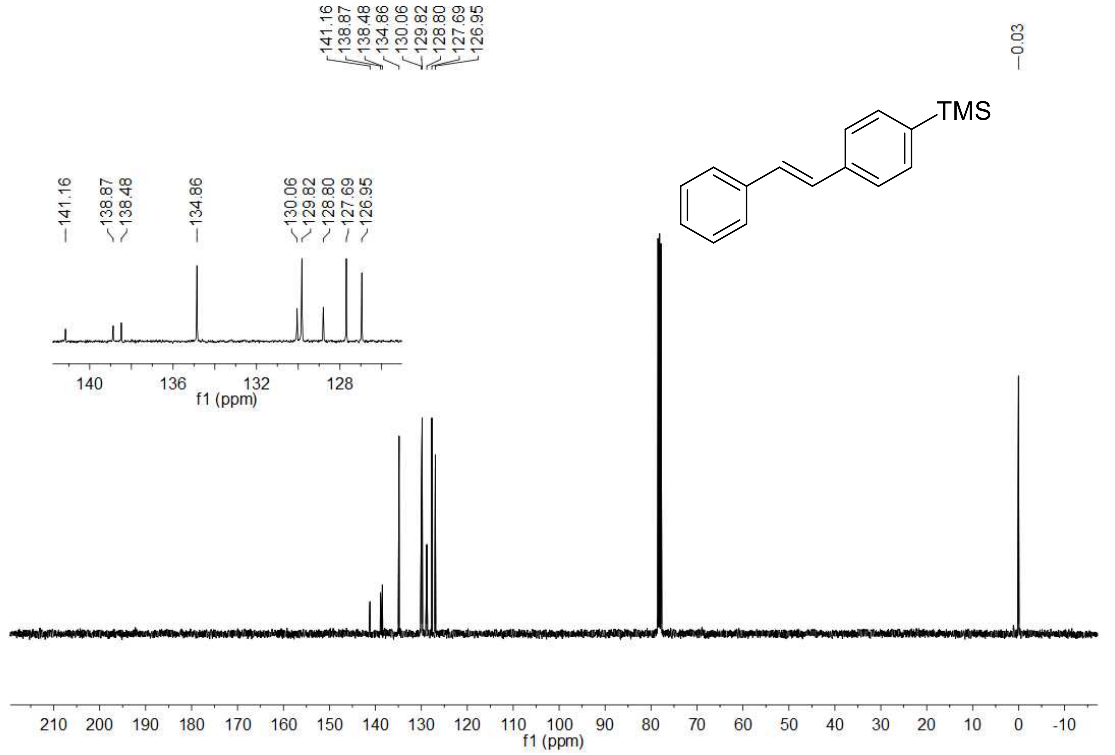

Supplementary Figure S46. ^{13}**C NMR spectrum for compound** ***E*****-3k (100 MHz, $CDCl_3$, 25 °C).**

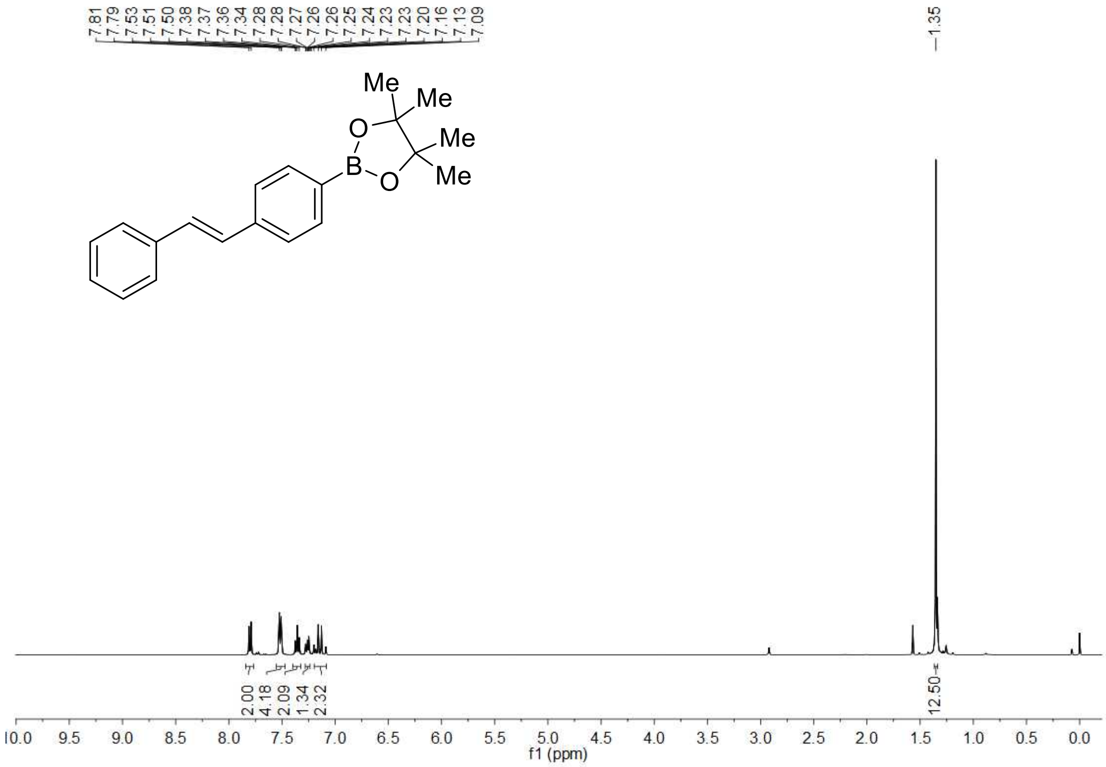

Supplementary Figure S47. **^{1}H NMR spectrum for compound *E*-3l (400 MHz, $CDCl_3$, 25 ^{o}C).**

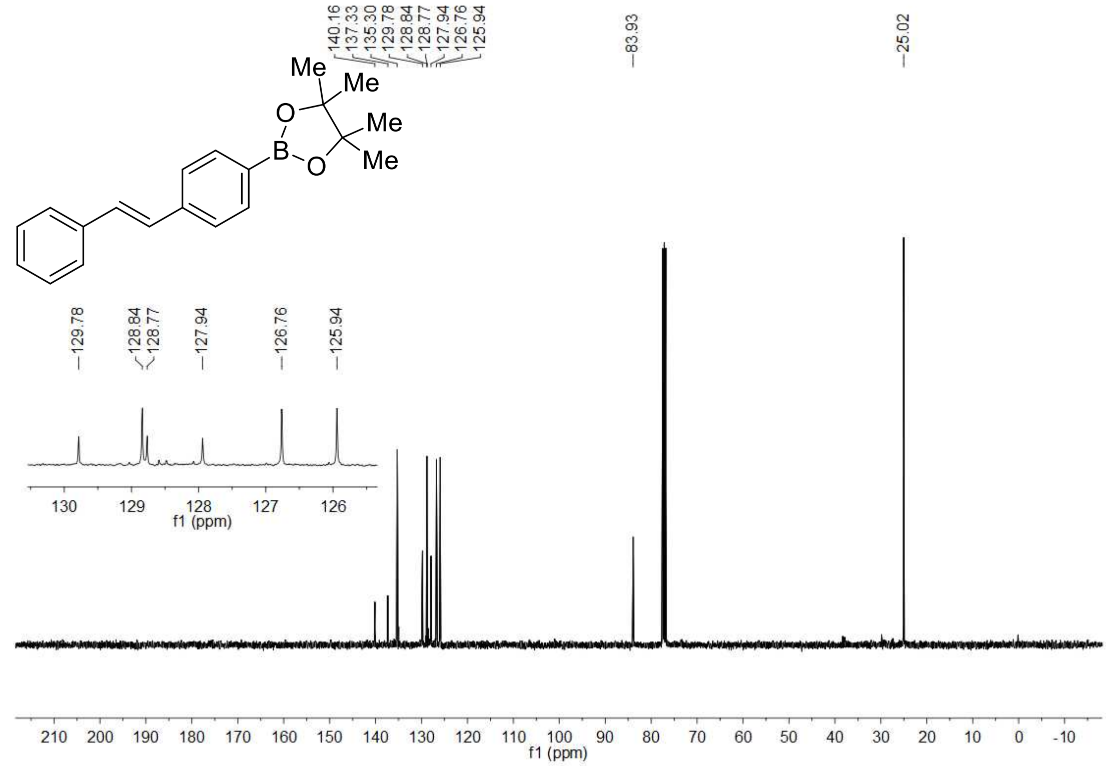

Supplementary Figure S48. **^{13}C NMR spectrum for compound *E*-3l (100 MHz, $CDCl_3$, 25 ^{o}C).**

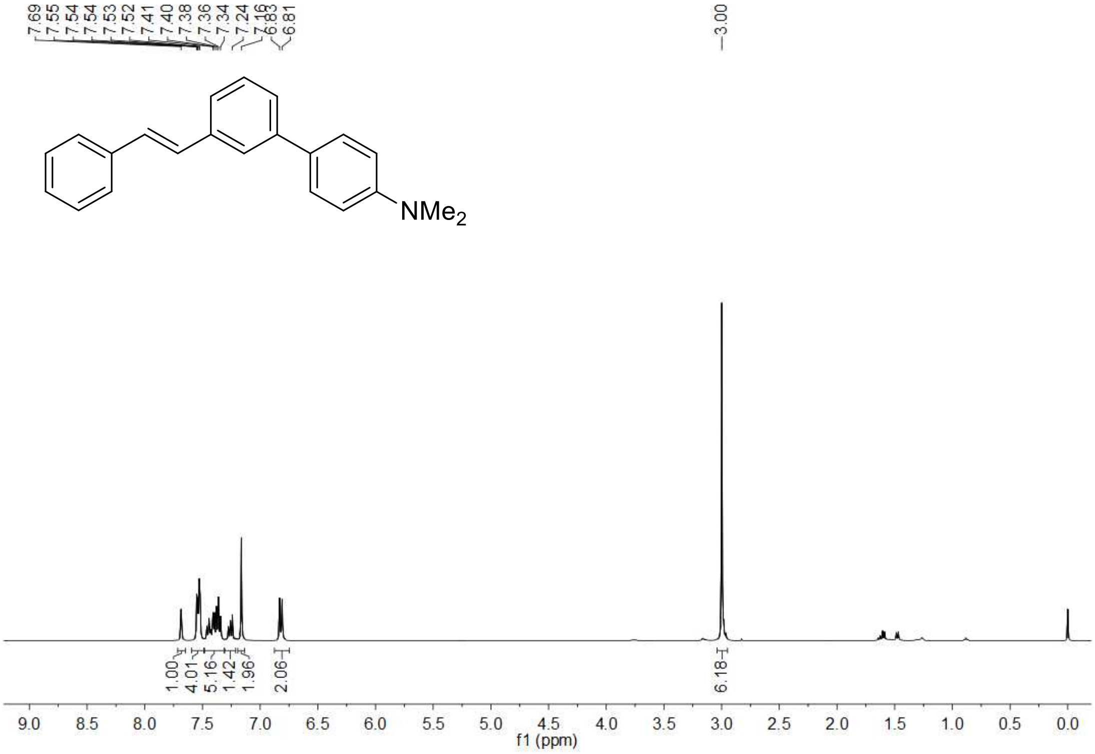

Supplementary Figure S49. ^{1}H NMR spectrum for compound *E*-3m (400 MHz, $CDCl_3$, 25 ^{o}C).

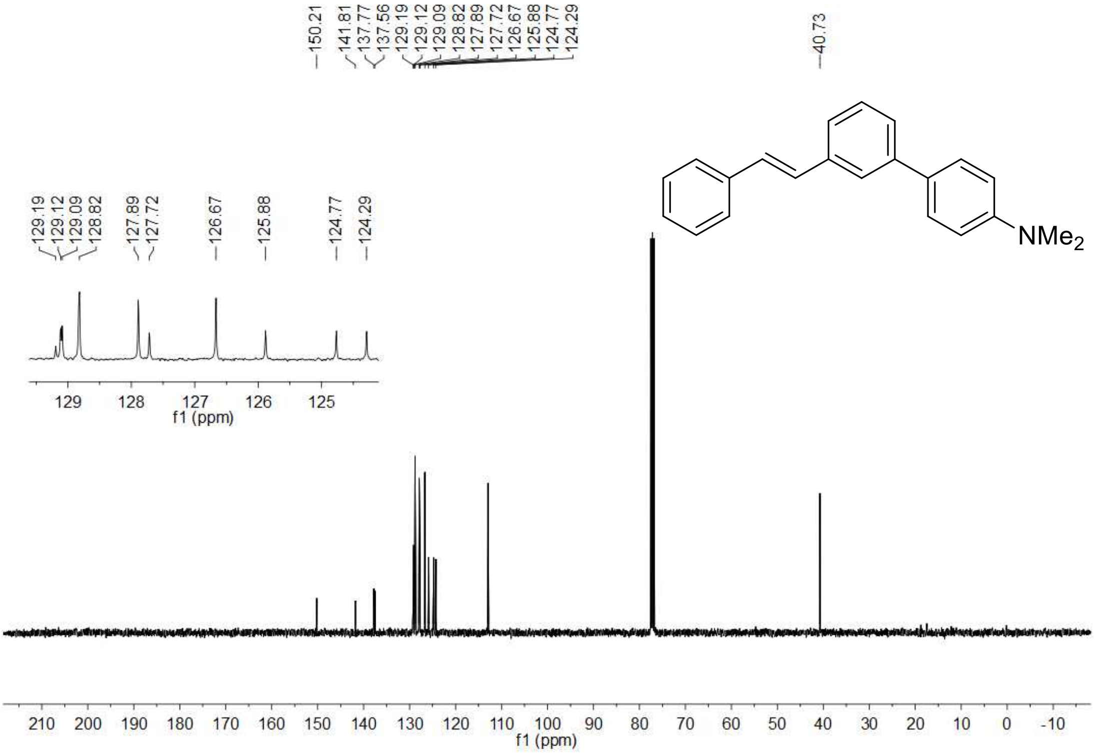

Supplementary Figure S50. ^{13}C NMR spectrum for compound *E*-3m (100 MHz, $CDCl_3$, 25 ^{o}C).

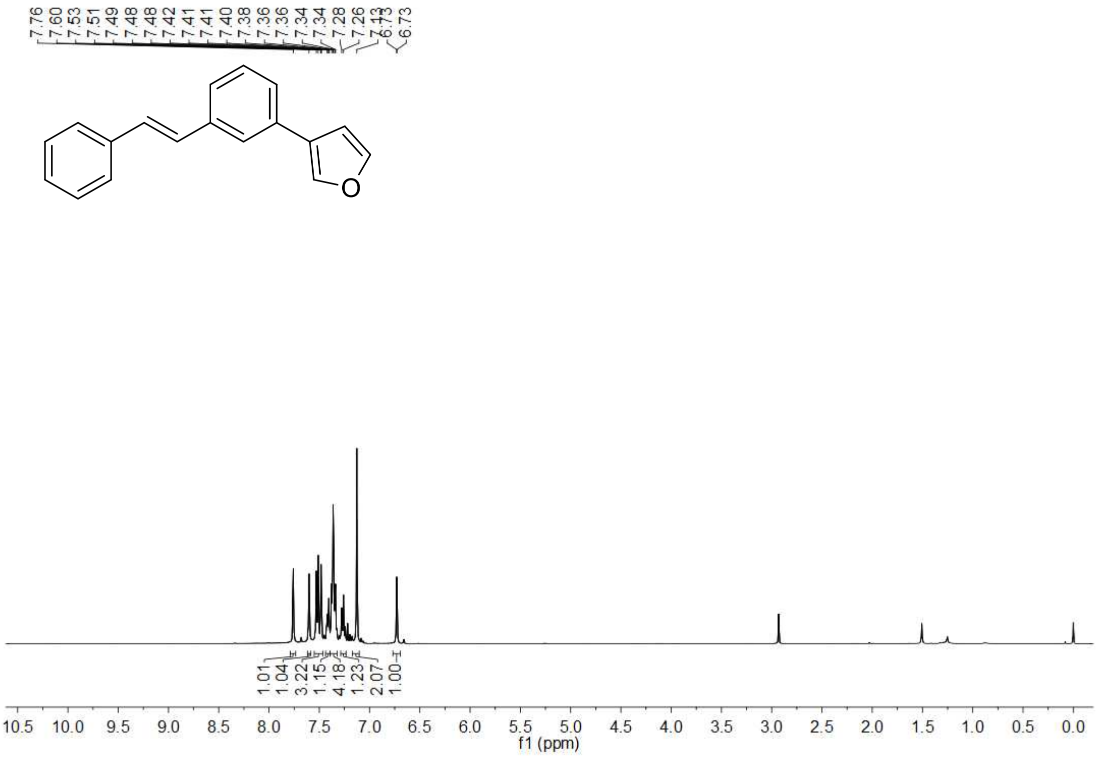

Supplementary Figure S51. **^{1}H NMR spectrum for compound *E*-3n (400 MHz, $CDCl_3$, 25 °C).**

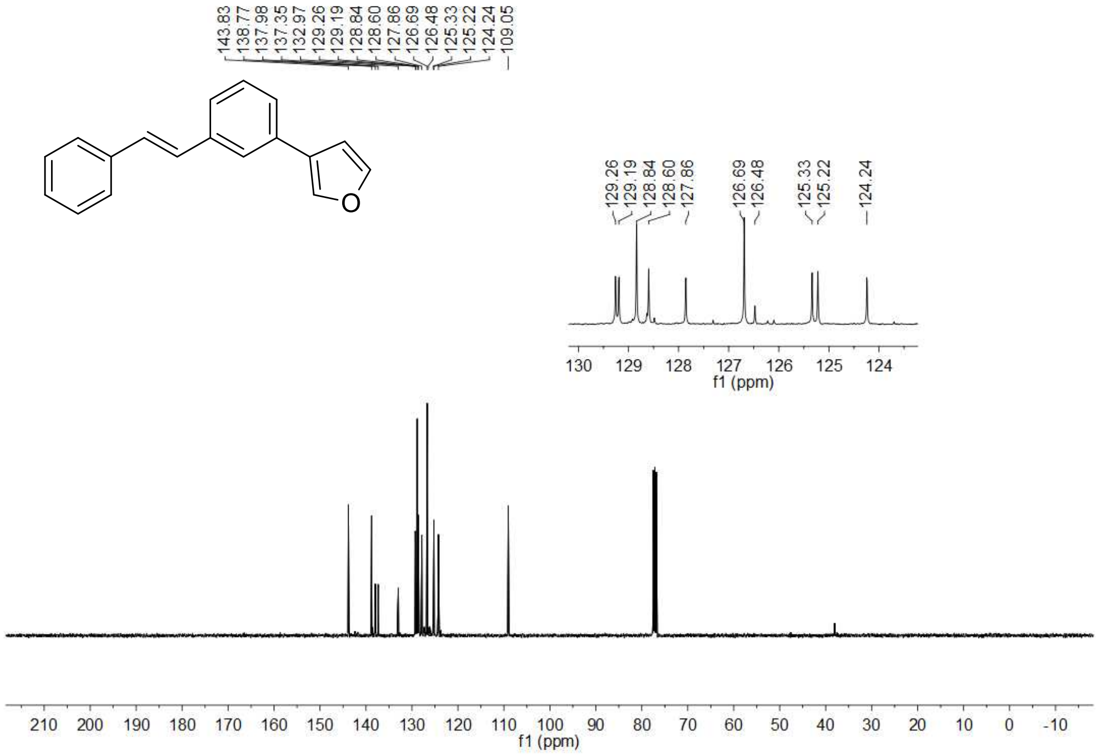

Supplementary Figure S52. **^{13}C NMR spectrum for compound *E*-3n (100 MHz, $CDCl_3$, 25 °C).**

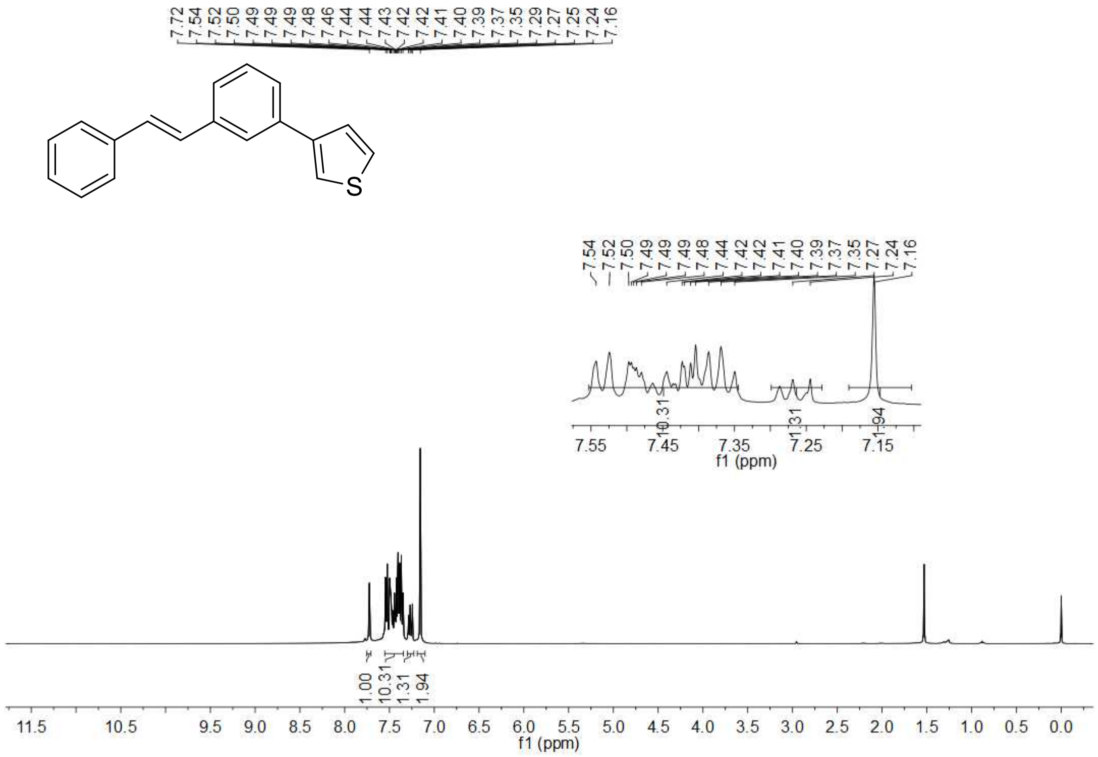

Supplementary Figure S53. **^{1}H NMR spectrum for compound *E*-3o (400 MHz, $CDCl_3$, 25 ^{o}C).**

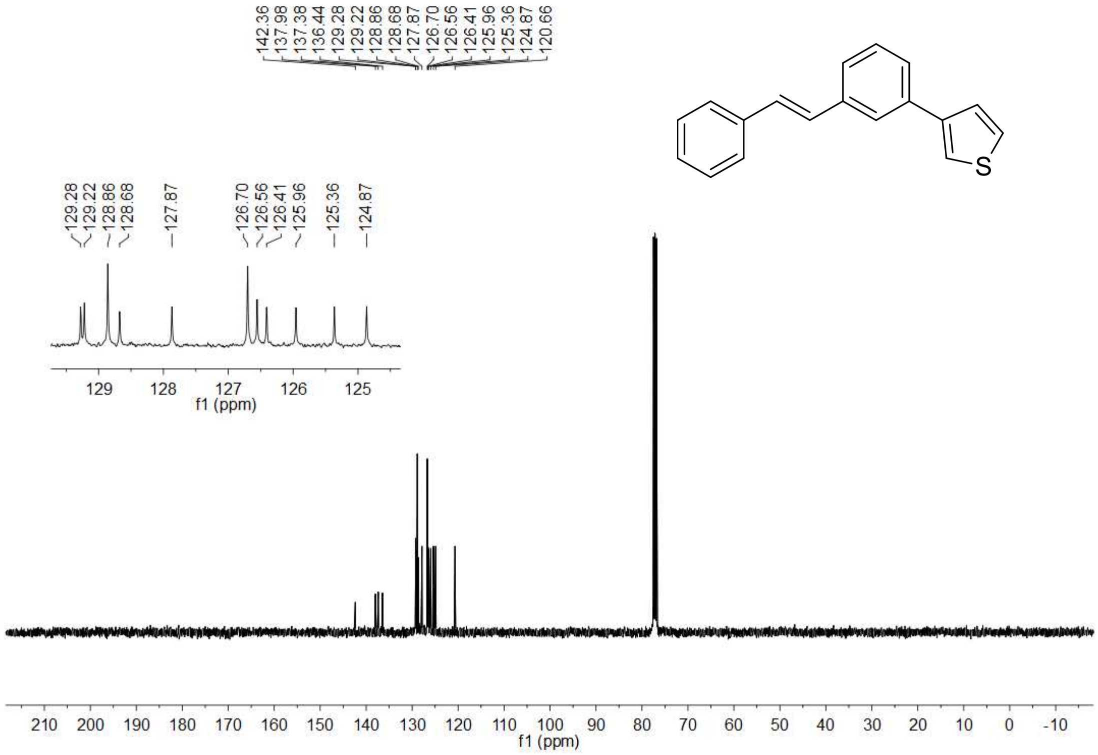

Supplementary Figure S54. **^{13}C NMR spectrum for compound *E*-3o (100 MHz, $CDCl_3$, 25 ^{o}C).**

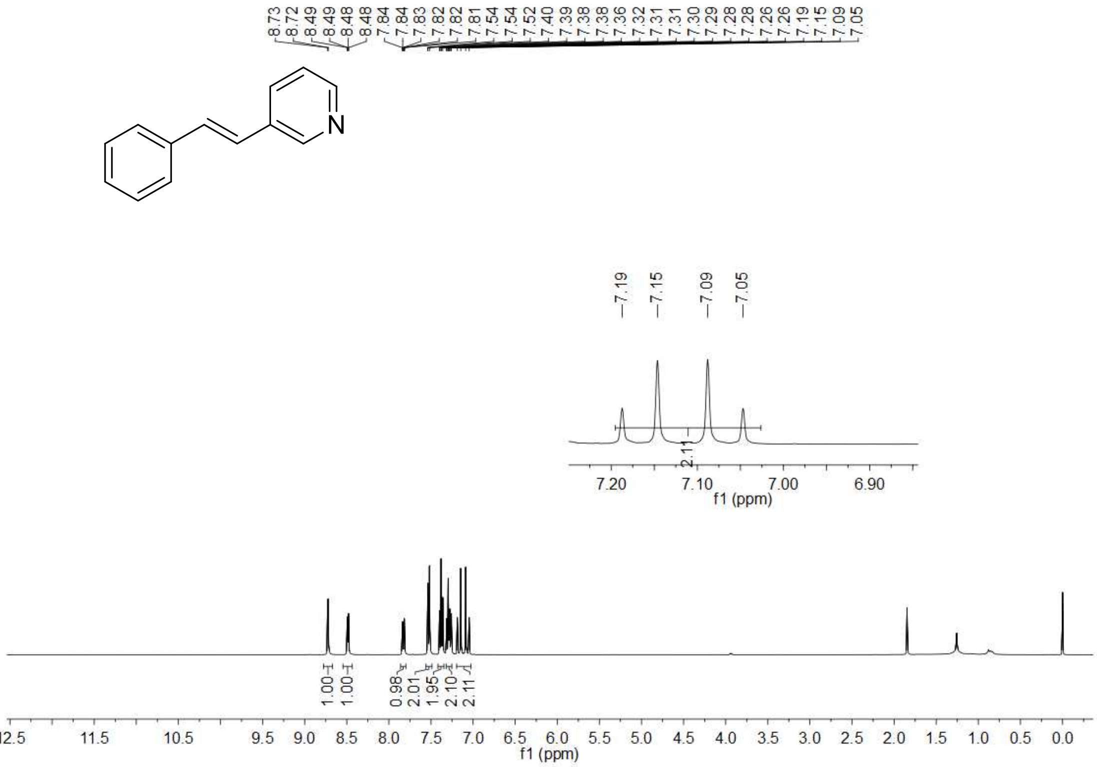

Supplementary Figure S55. **^{1}H NMR spectrum for compound *E*-3p (400 MHz, $CDCl_3$, 25 ^{o}C).**

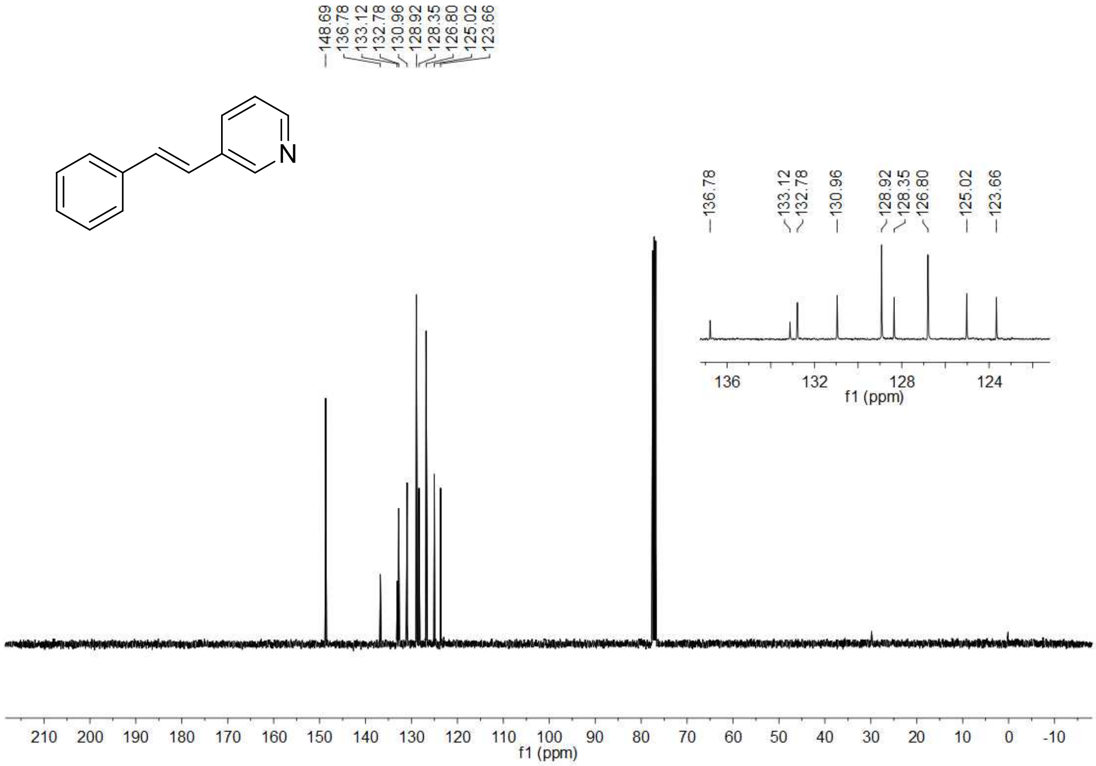

Supplementary Figure S56. **^{13}C NMR spectrum for compound *E*-3p (100 MHz, $CDCl_3$, 25 ^{o}C).**

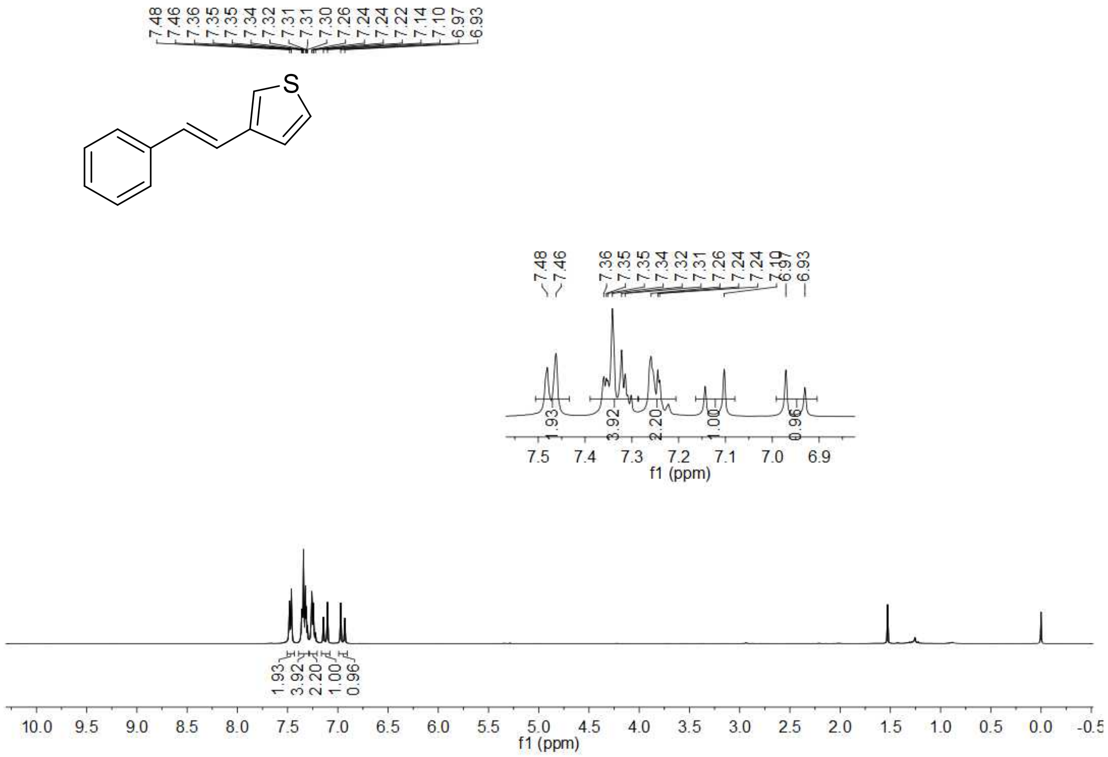

Supplementary Figure S57. **^{1}H NMR spectrum for compound *E*-3q (400 MHz, $CDCl_3$, 25 ^{o}C).**

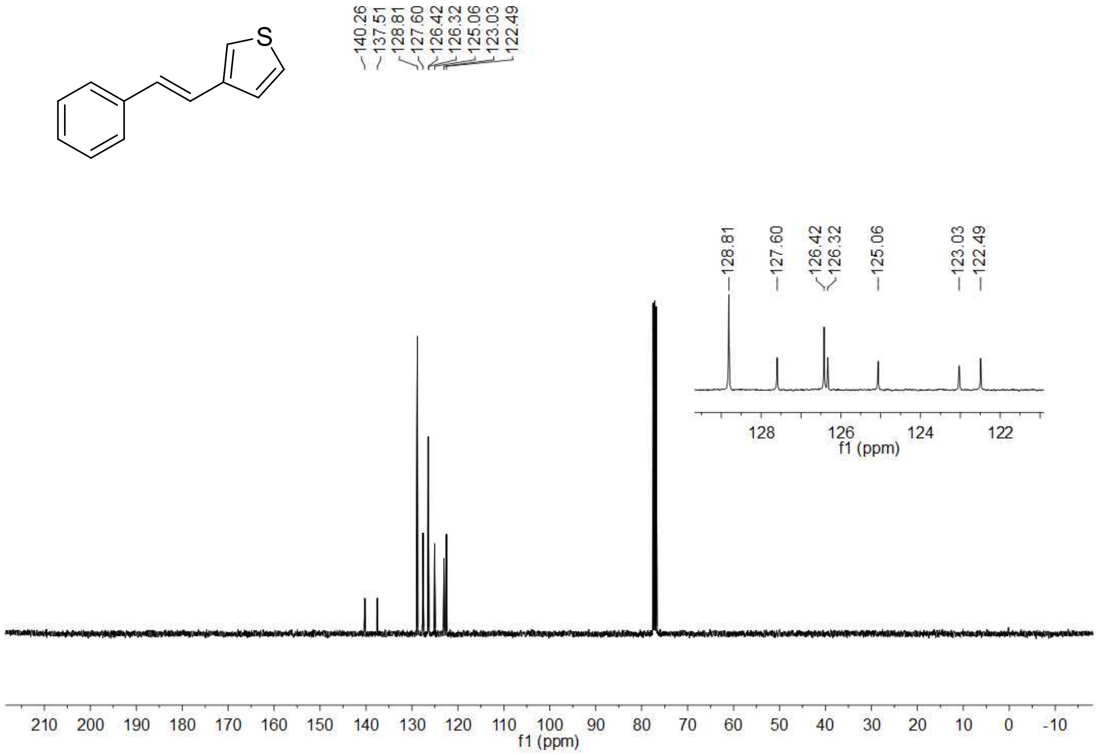

Supplementary Figure S58. **^{13}C NMR spectrum for compound *E*-3q (100 MHz, $CDCl_3$, 25 ^{o}C).**

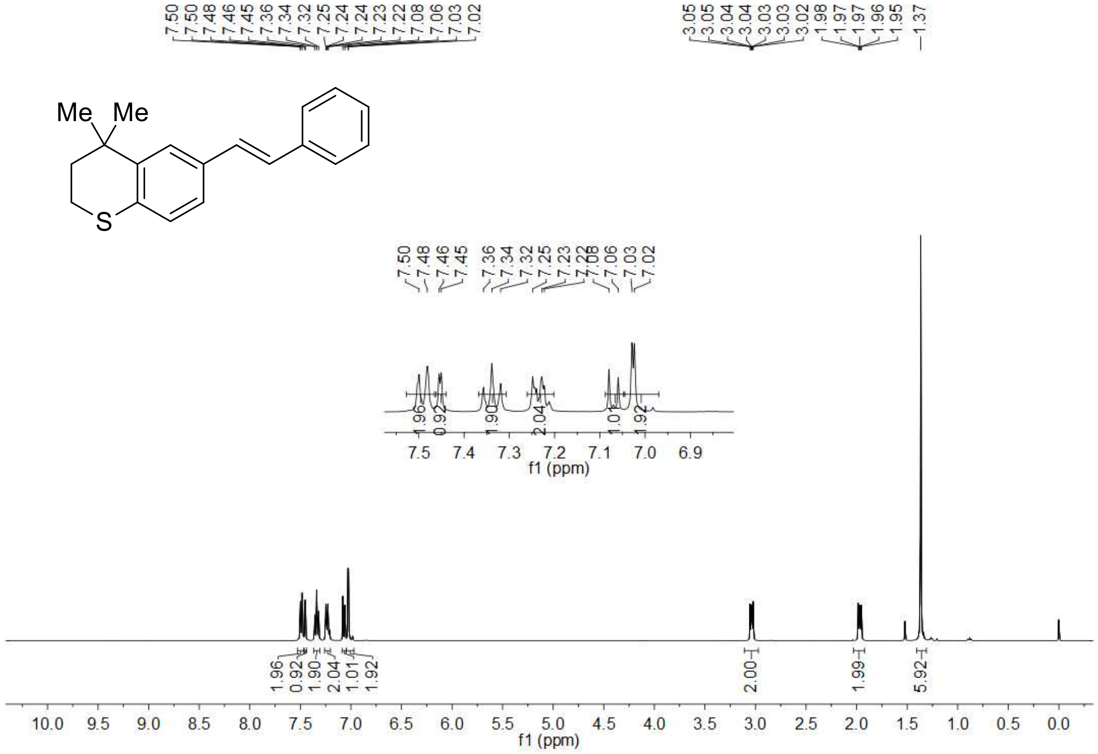

Supplementary Figure S59. ^{1}H NMR spectrum for compound *E*-3r (400 MHz, $CDCl_3$, 25 °C).

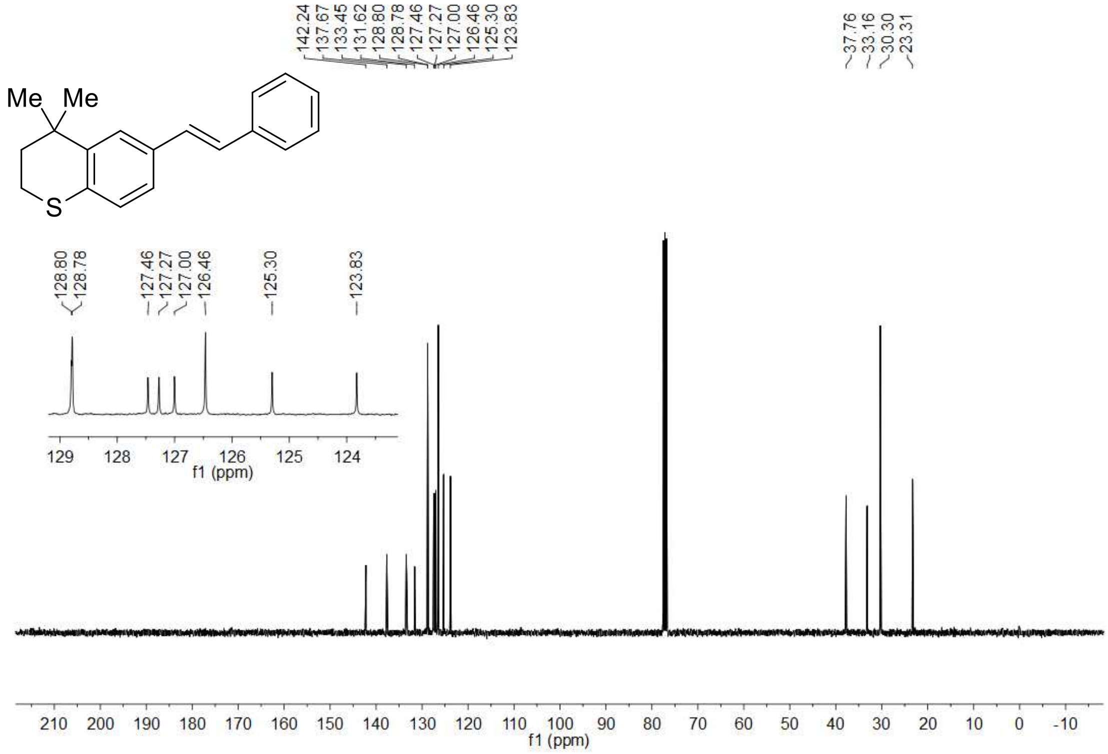

Supplementary Figure S60. ^{13}C NMR spectrum for compound *E*-3r (100 MHz, $CDCl_3$, 25 °C).

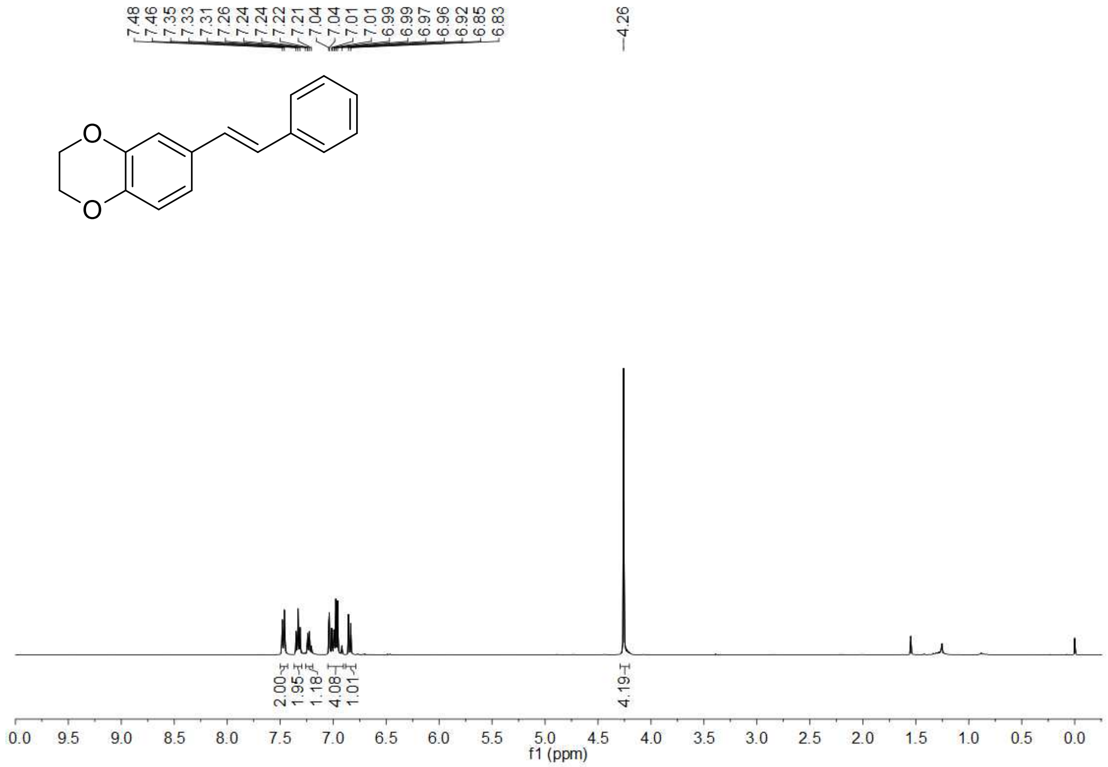

Supplementary Figure S61. ¹H NMR spectrum for compound *E*-3s (400 MHz, $CDCl_3$, 25 °C).

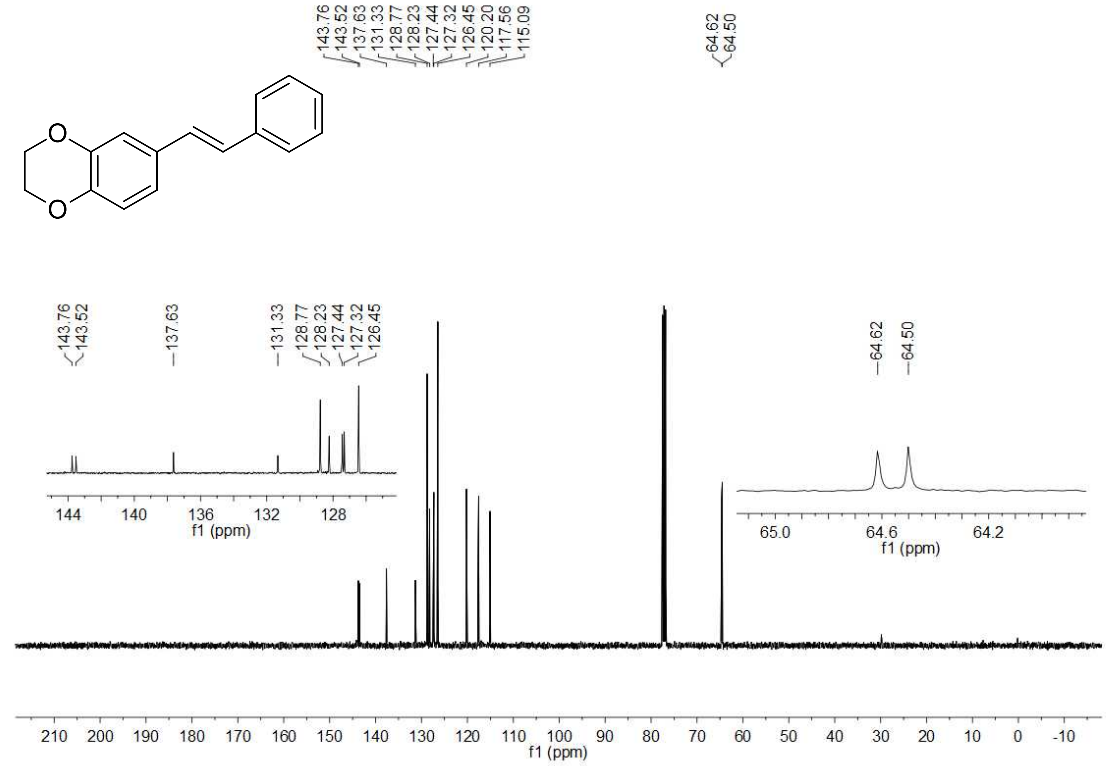

Supplementary Figure S62. ¹³C NMR spectrum for compound *E*-3s (100 MHz, $CDCl_3$, 25 °C).

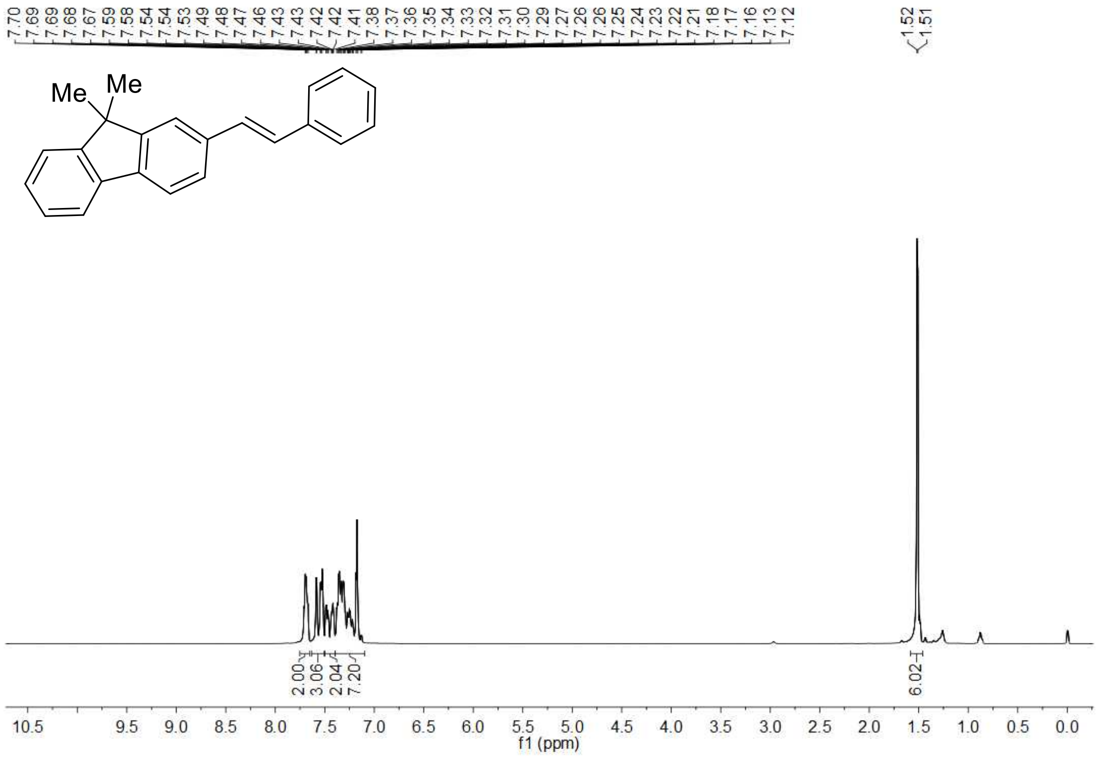

Supplementary Figure S63. ^{1}**H NMR spectrum for compound** ***E*****-3t (400 MHz, CDCl$_3$, 25 °C).**

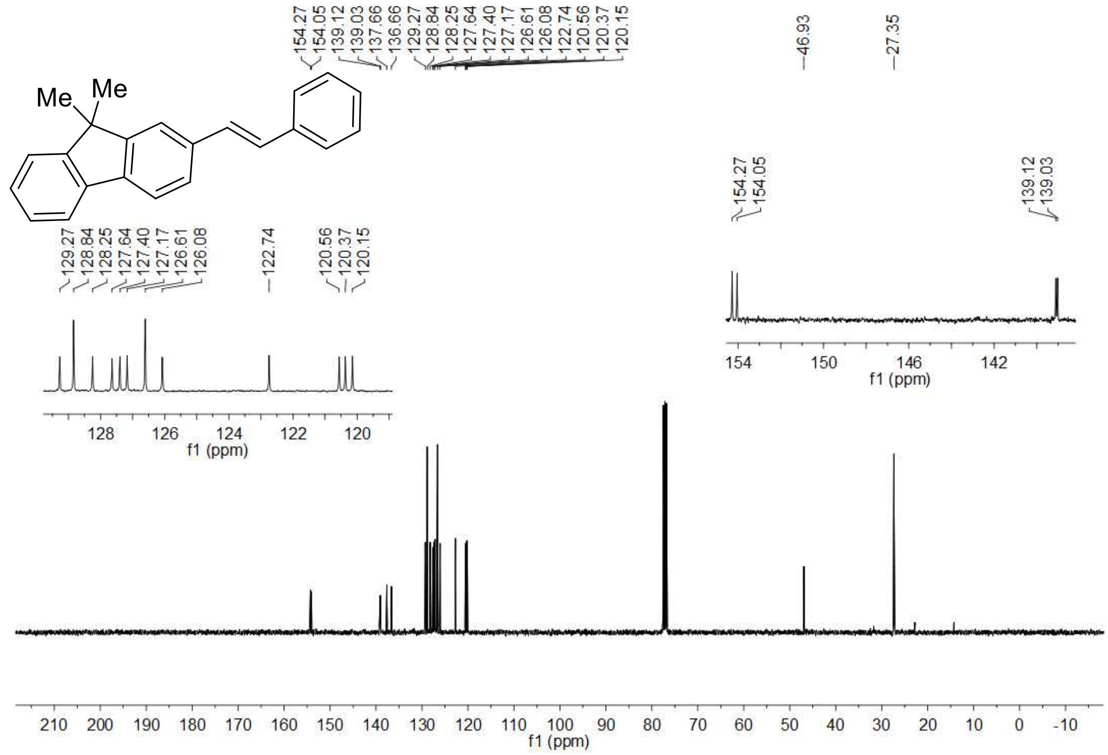

Supplementary Figure S64. ^{13}**C NMR spectrum for compound** ***E*****-3t (100 MHz, CDCl$_3$, 25 °C).**

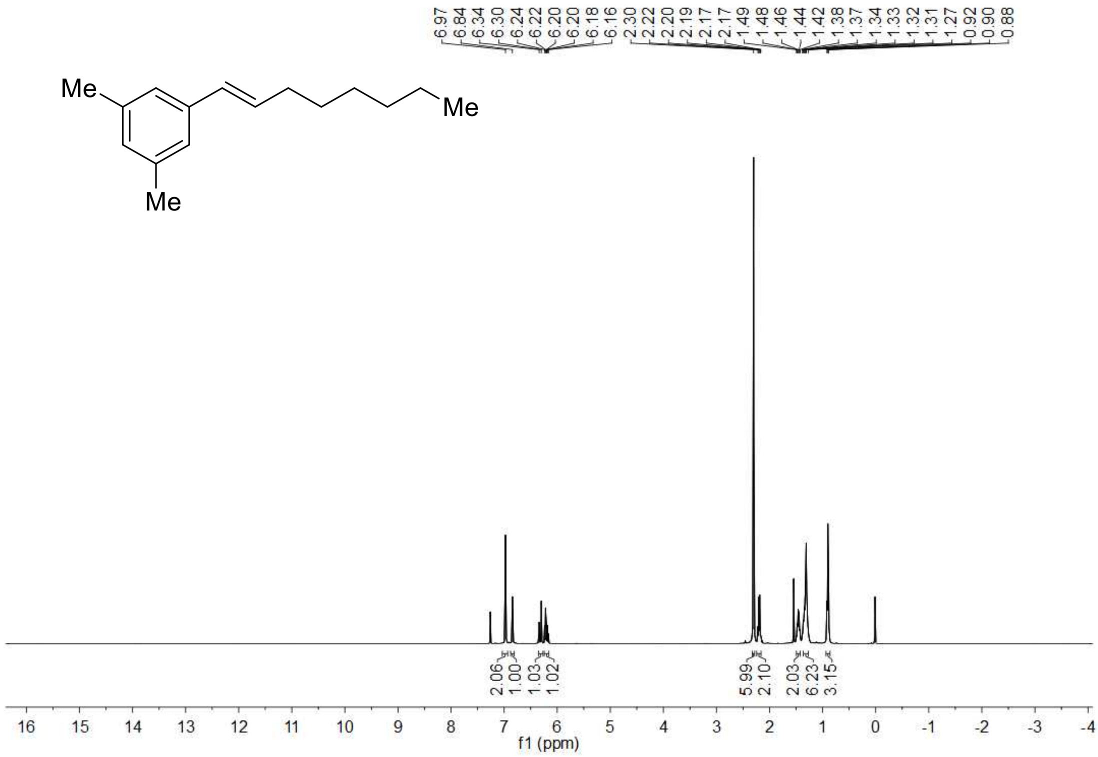

Supplementary Figure S65. ^{1}**H NMR spectrum for compound** ***E*-3u (400 MHz, $CDCl_3$, 25 °C).**

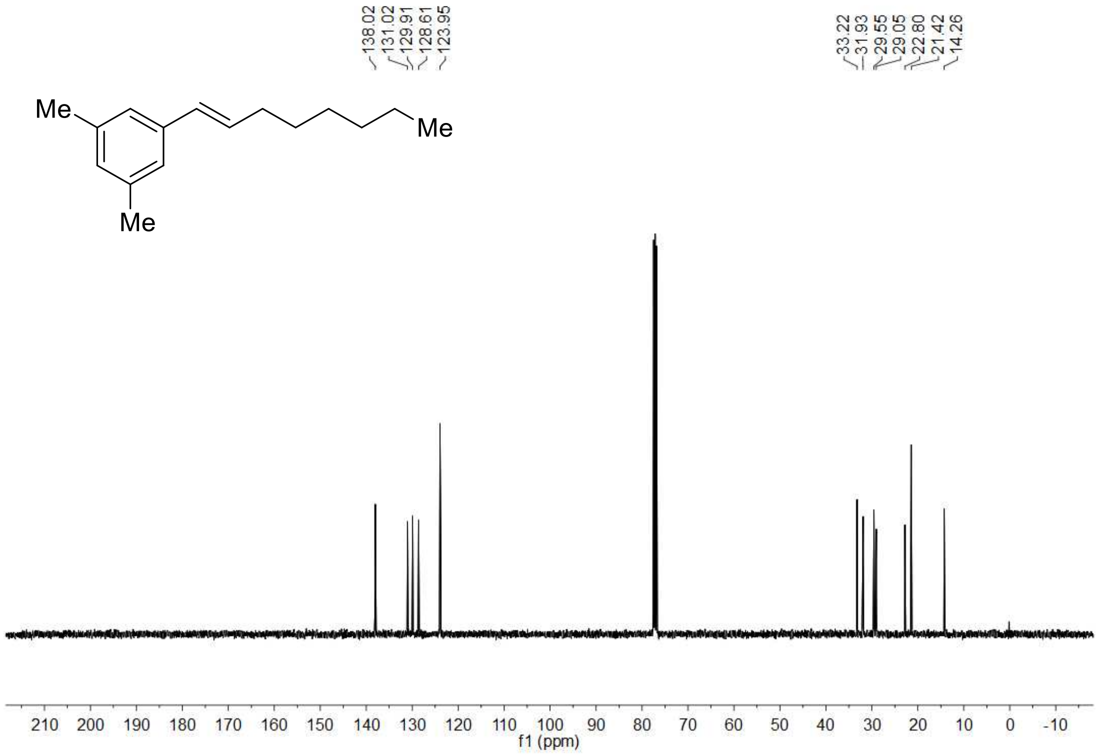

Supplementary Figure S66. ^{13}**C NMR spectrum for compound** ***E*-3u (100 MHz, $CDCl_3$, 25 °C).**

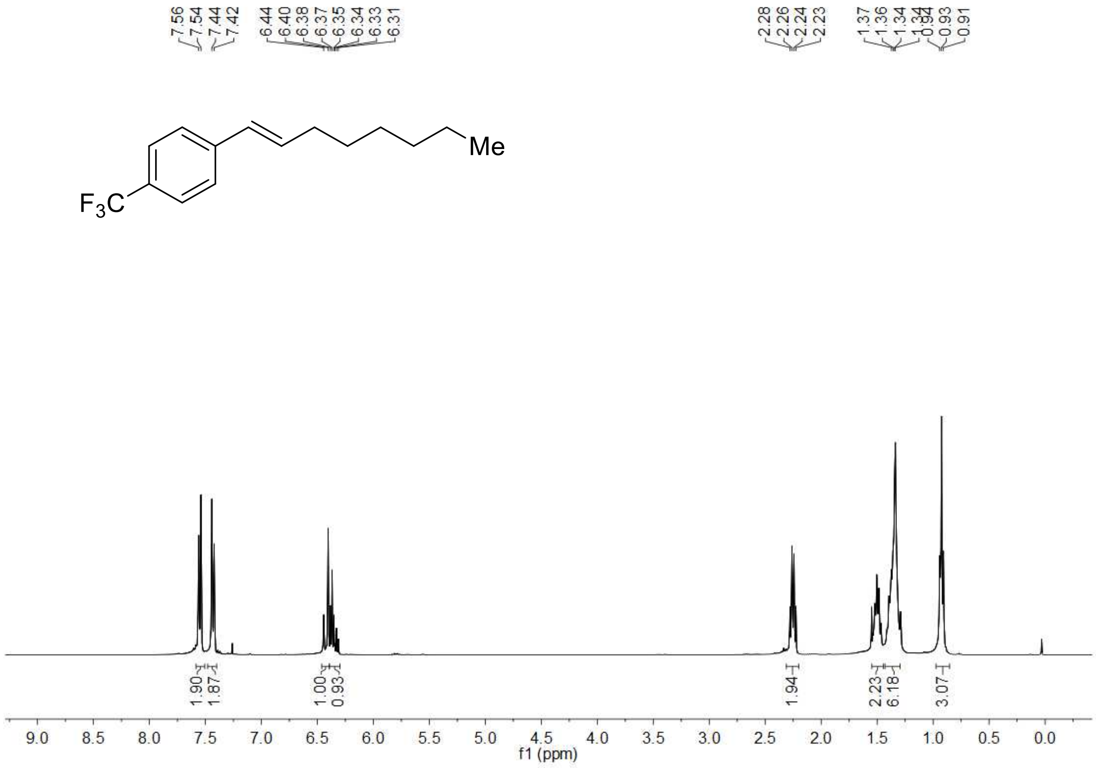

Supplementary Figure S67. **^{1}H NMR spectrum for compound *E*-3v (400 MHz, $CDCl_3$, 25 °C).**

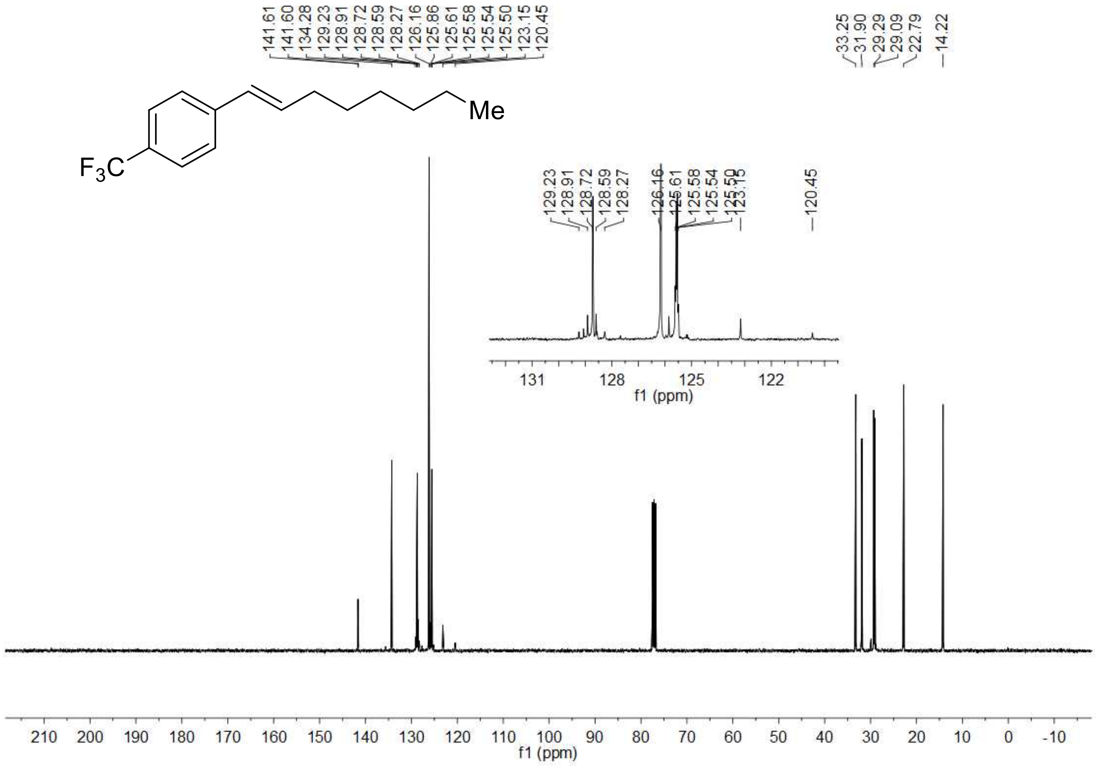

Supplementary Figure S68. **^{13}C NMR spectrum for compound *E*-3v (100 MHz, $CDCl_3$, 25 °C).**

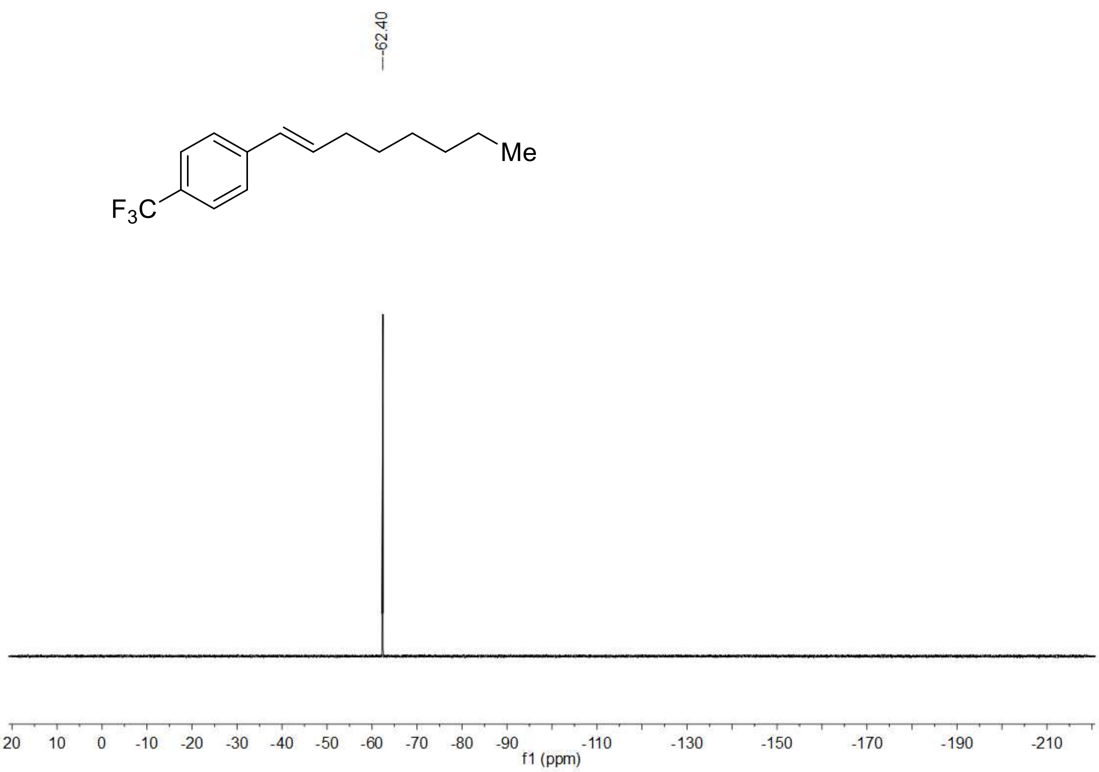

Supplementary Figure S69. ^{19}F NMR spectrum for compound *E*-3v (376 MHz, $CDCl_3$, 25 °C).

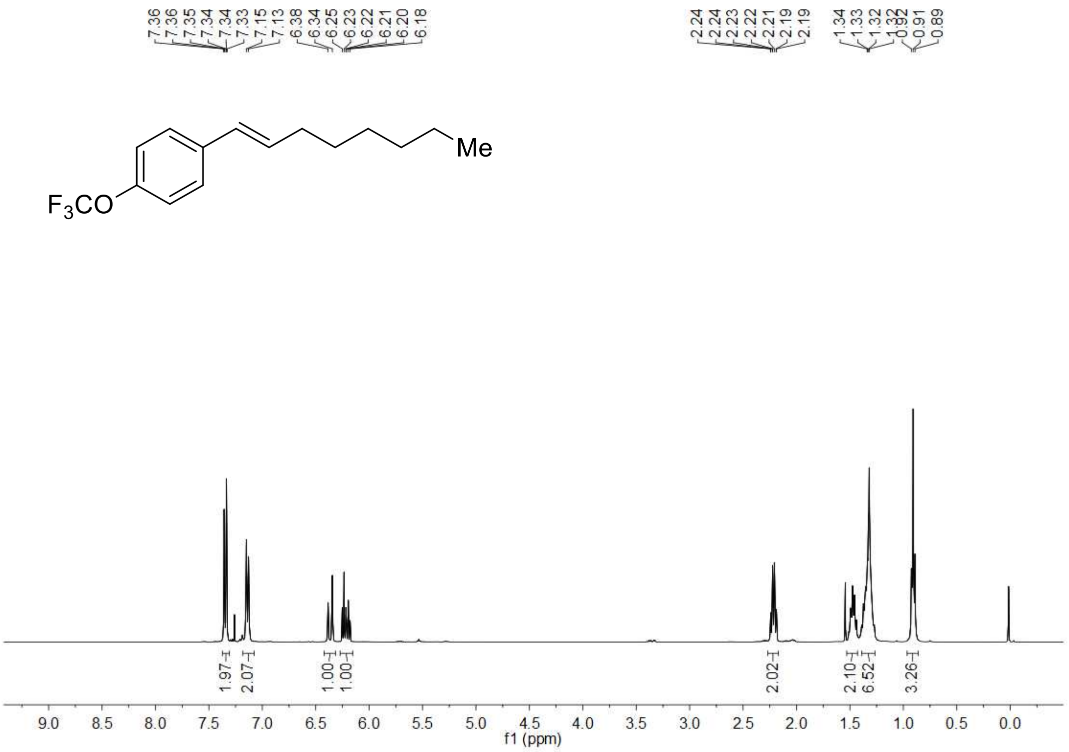

Supplementary Figure S70. ^{1}H NMR spectrum for compound *E*-3w (400 MHz, $CDCl_3$, 25 °C).

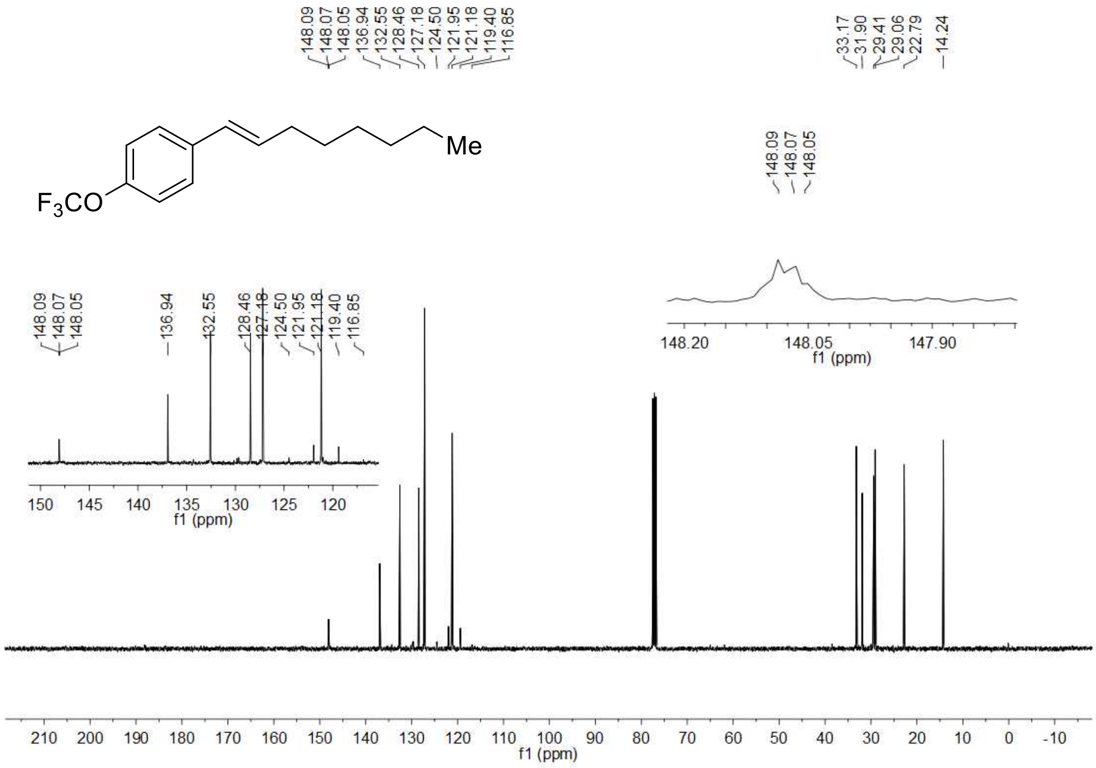

Supplementary Figure S71. ^{13}C NMR spectrum for compound *E*-3w (100 MHz, $CDCl_3$, 25 °C).

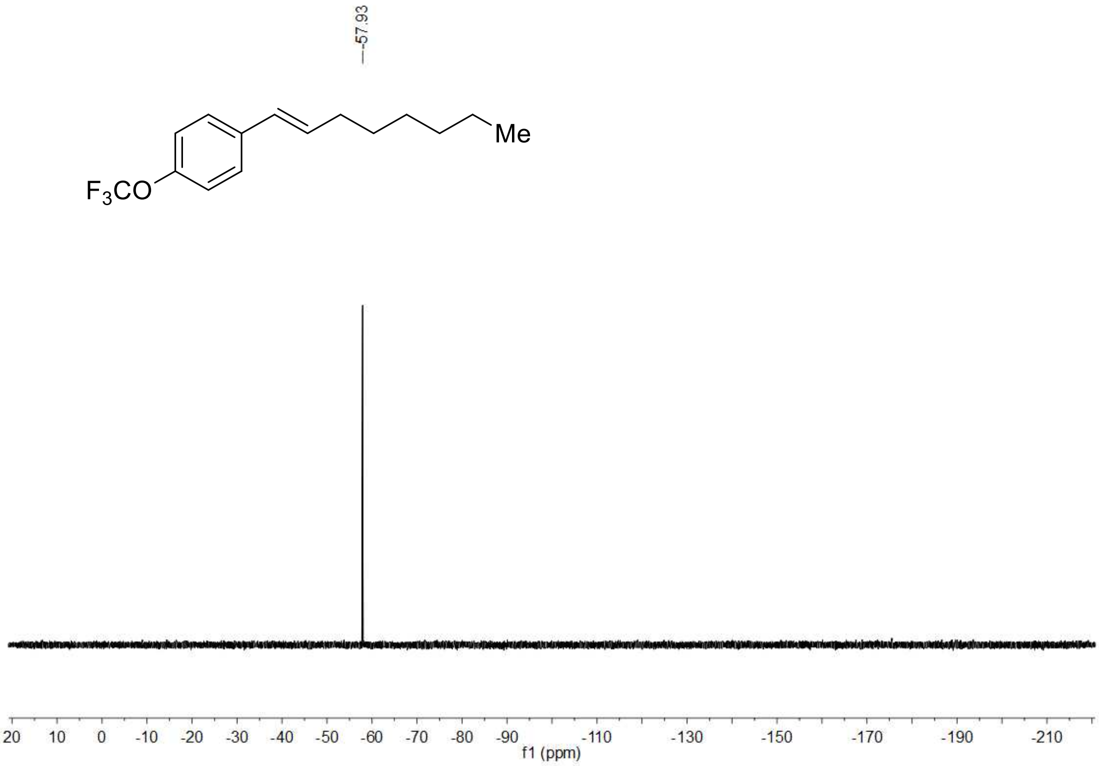

Supplementary Figure S72. ^{19}F NMR spectrum for compound *E*-3w (376 MHz, $CDCl_3$, 25 °C).

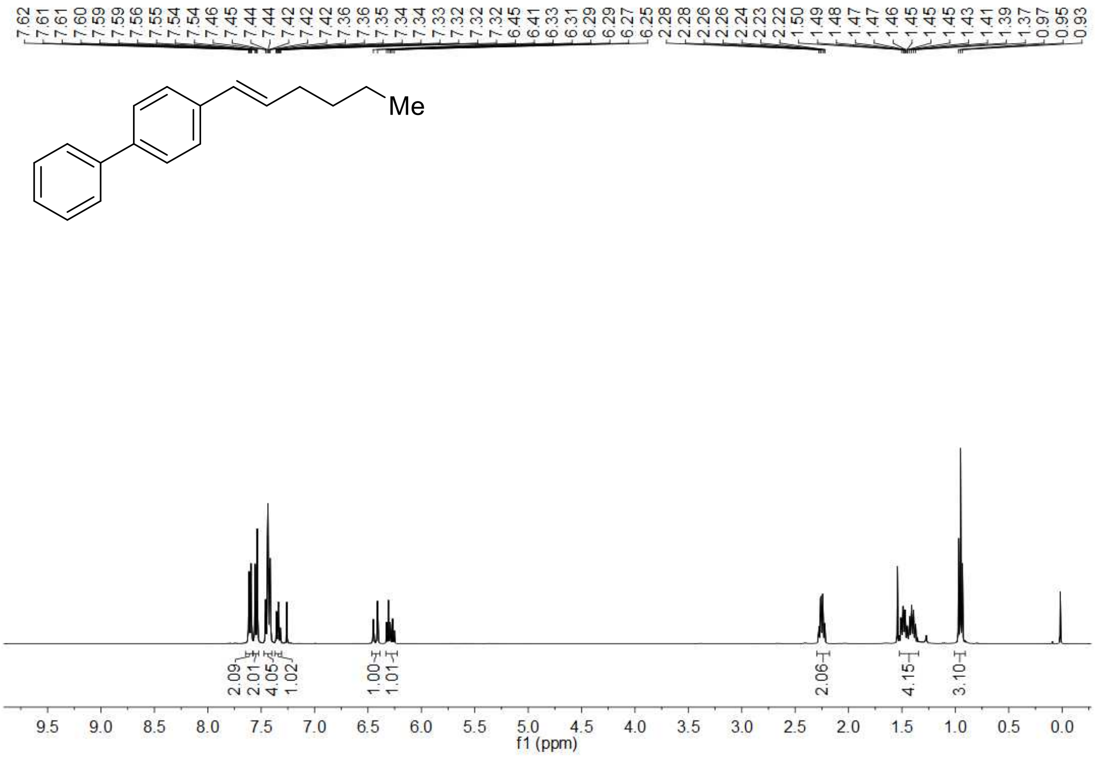

Supplementary Figure S73. ^{1}H NMR spectrum for compound *E*-3x (400 MHz, $CDCl_3$, 25 ^{o}C).

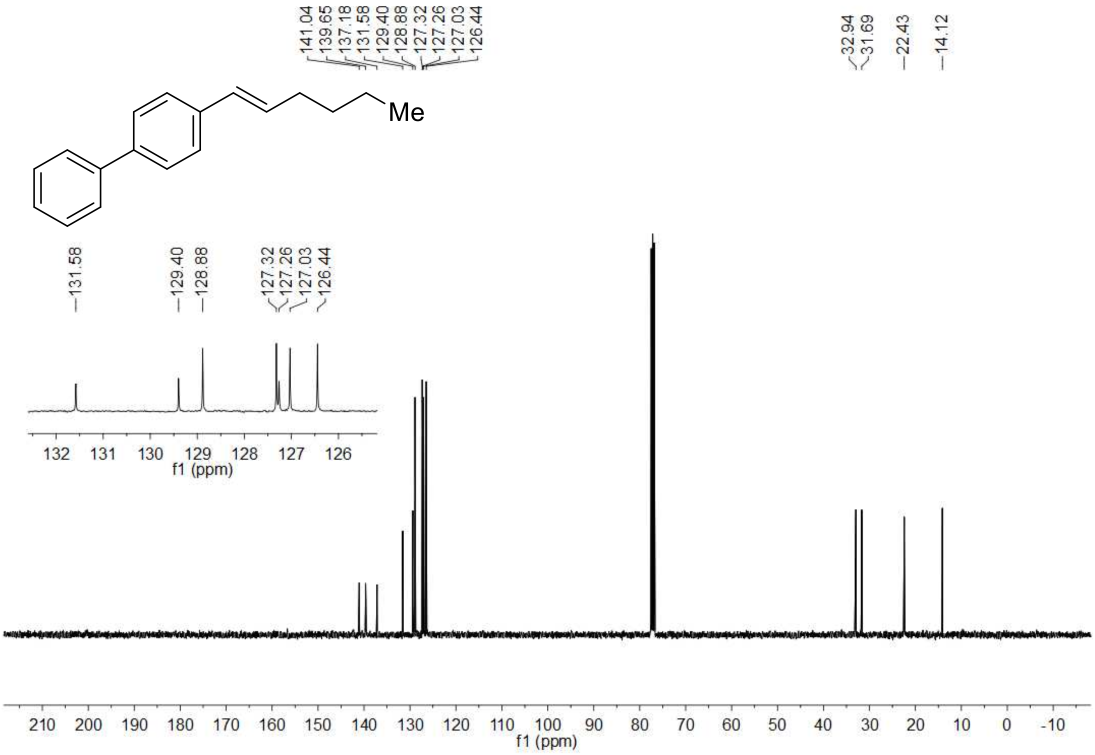

Supplementary Figure S74. ^{13}C NMR spectrum for compound *E*-3x (100 MHz, $CDCl_3$, 25 ^{o}C).

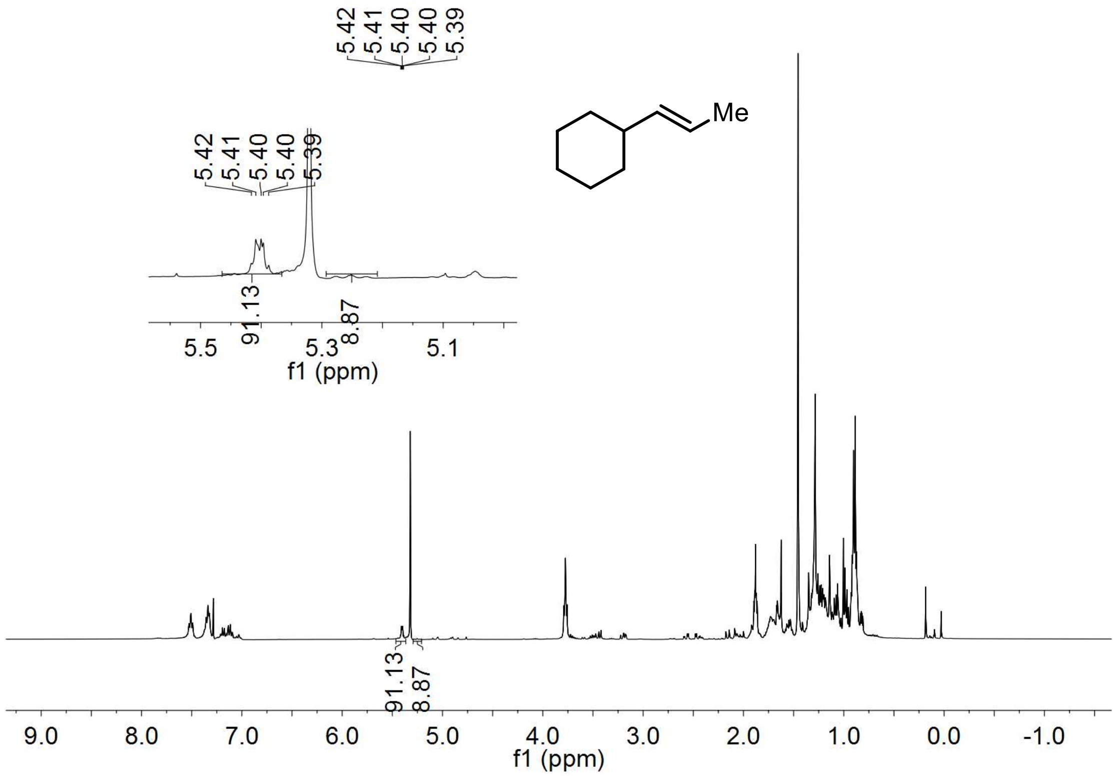

Supplementary Figure S75. **^{1}H NMR spectrum for compound *E*-3y (400 MHz, $CDCl_3$, 25 °C).**

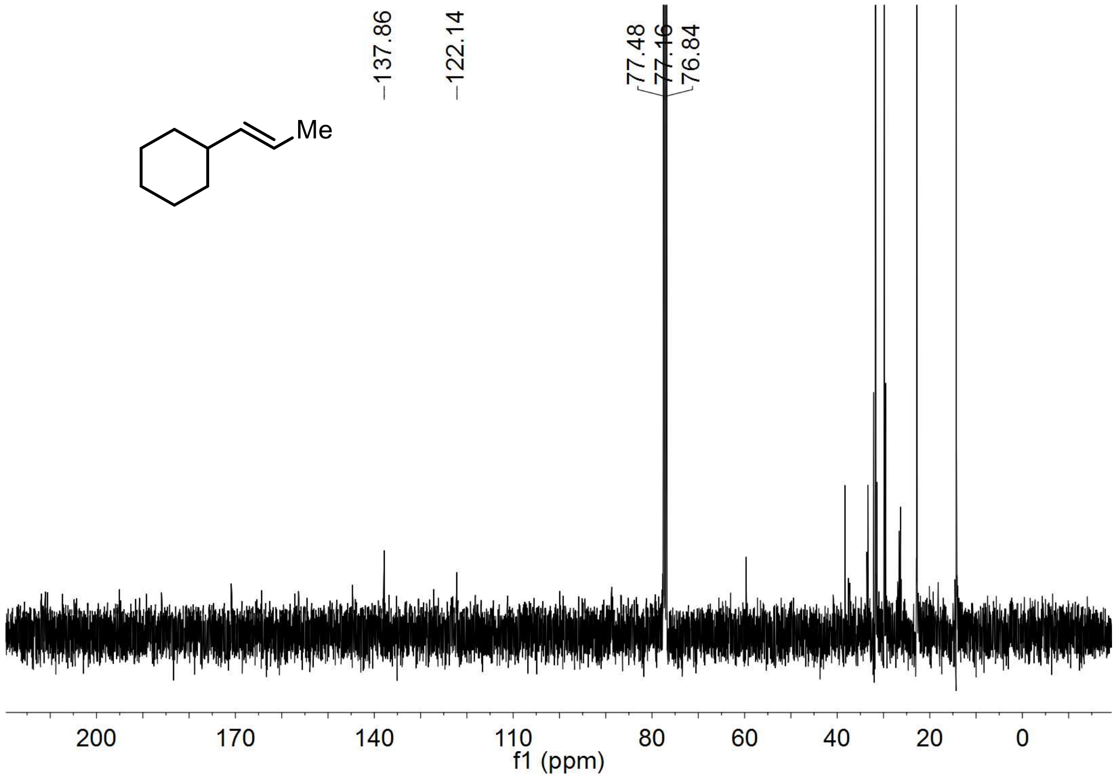

Supplementary Figure S76. **^{13}C NMR spectrum for compound *E*-3y (100 MHz, $CDCl_3$, 25 °C).**

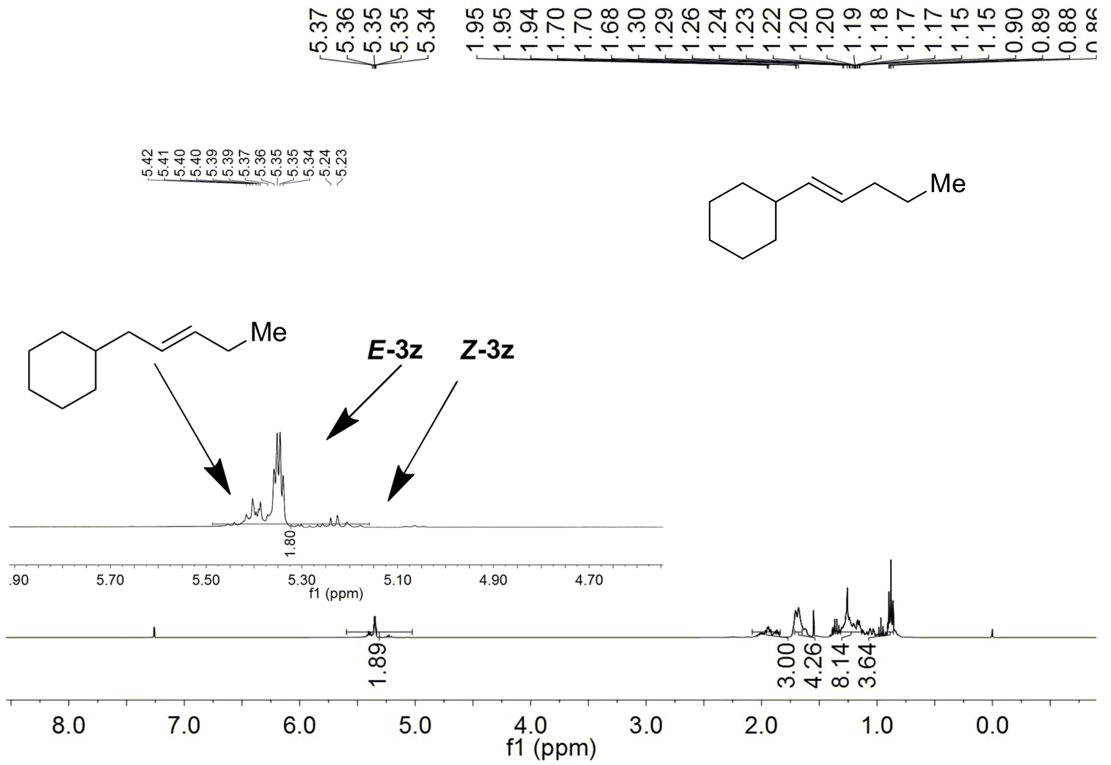

Supplementary Figure S77. ^{1}H NMR spectrum for compound *E*-3z (400 MHz, $CDCl_3$, 25 °C).

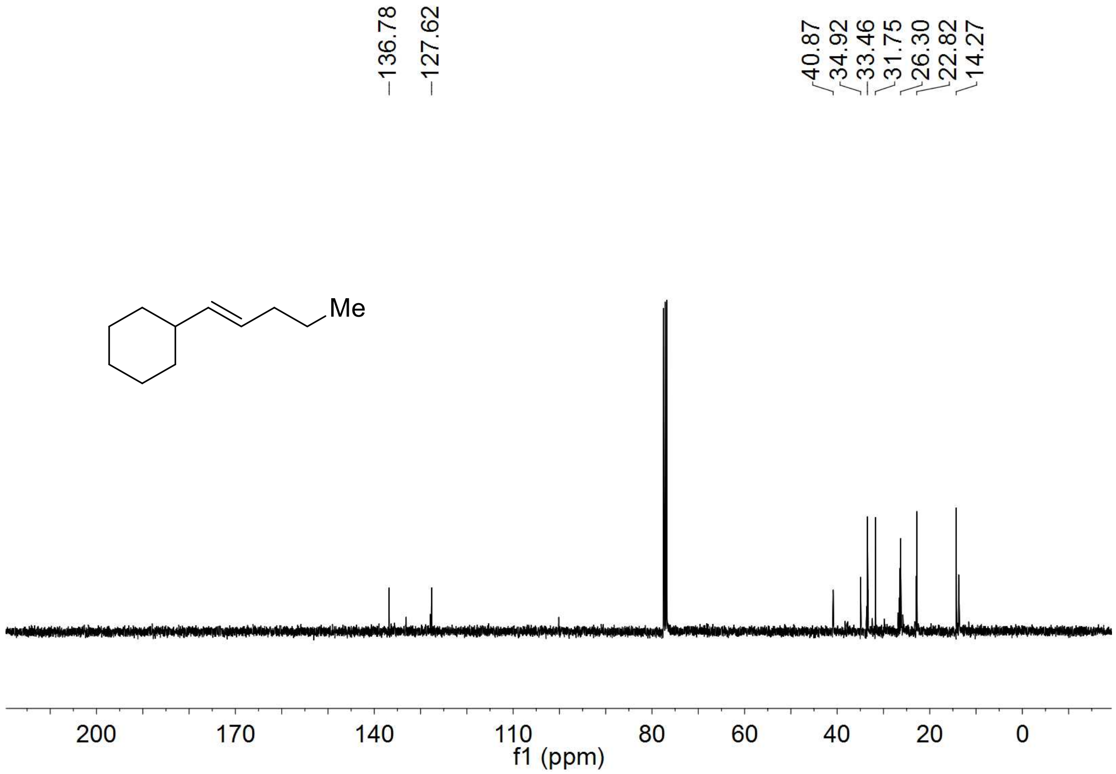

Supplementary Figure S78. ^{13}C NMR spectrum for compound *E*-3z (100 MHz, $CDCl_3$, 25 °C).

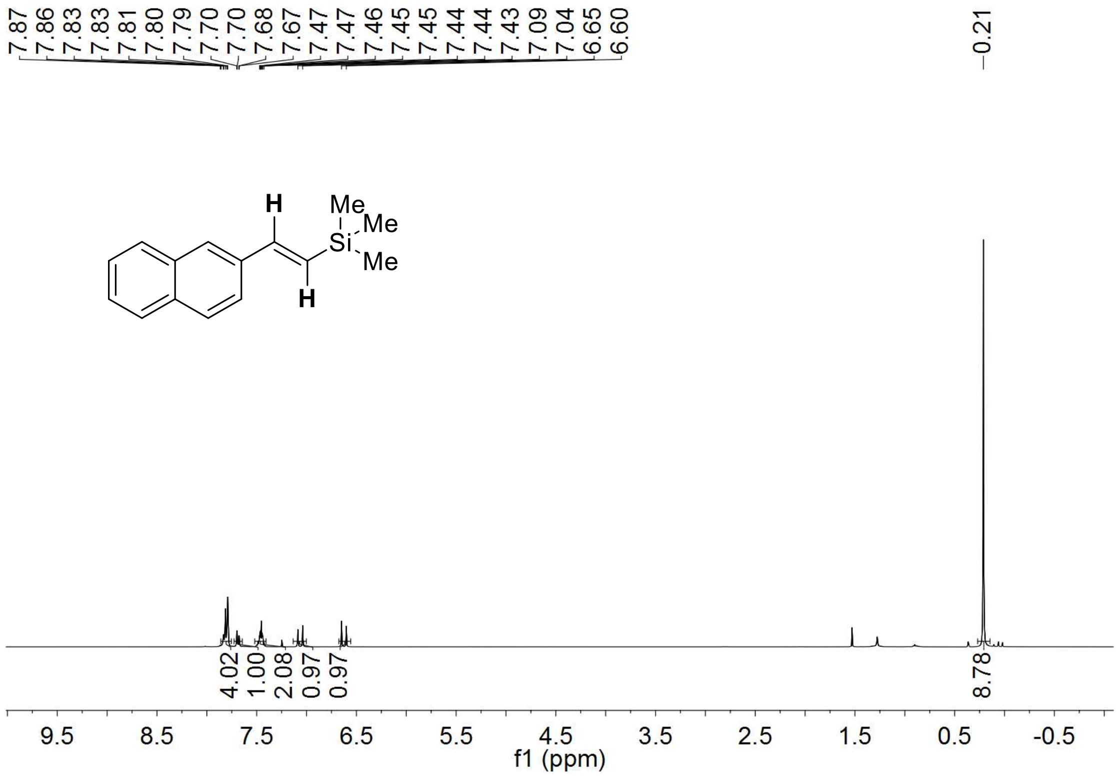

Supplementary Figure S79. ^{1}H NMR spectrum for compound *E*-3aa (400 MHz, $CDCl_3$, 25 °C).

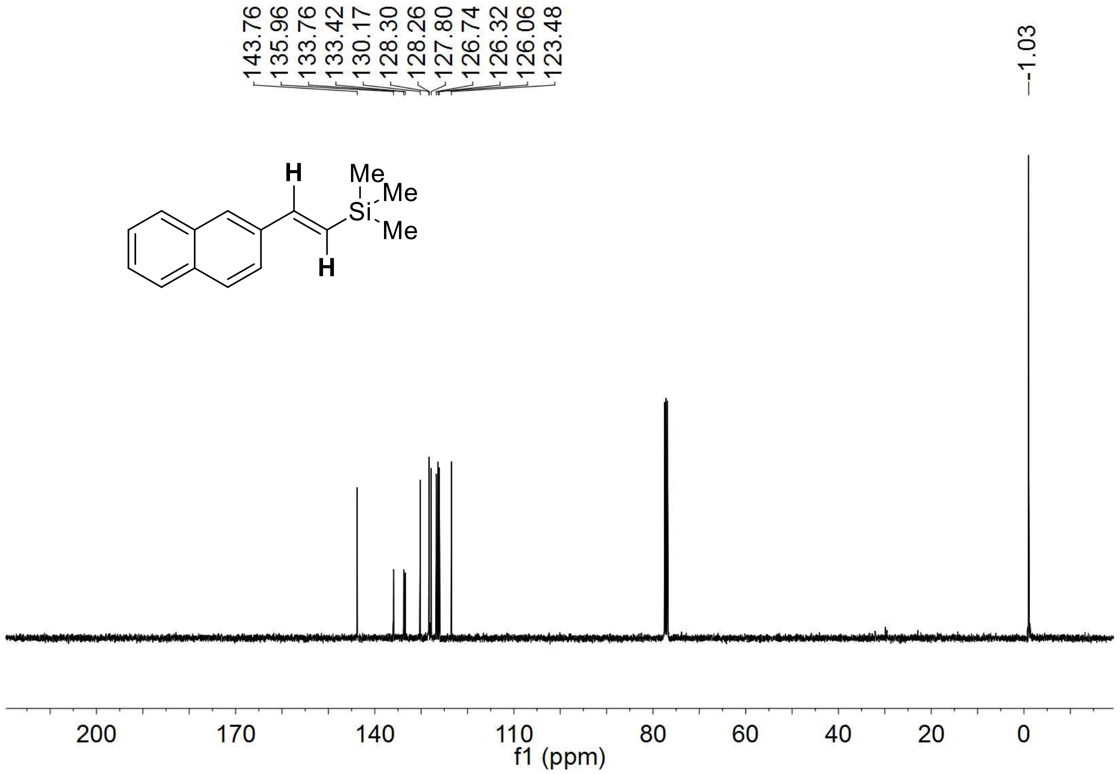

Supplementary Figure S80. ^{13}C NMR spectrum for compound *E*-3aa (100 MHz, $CDCl_3$, 25 °C).

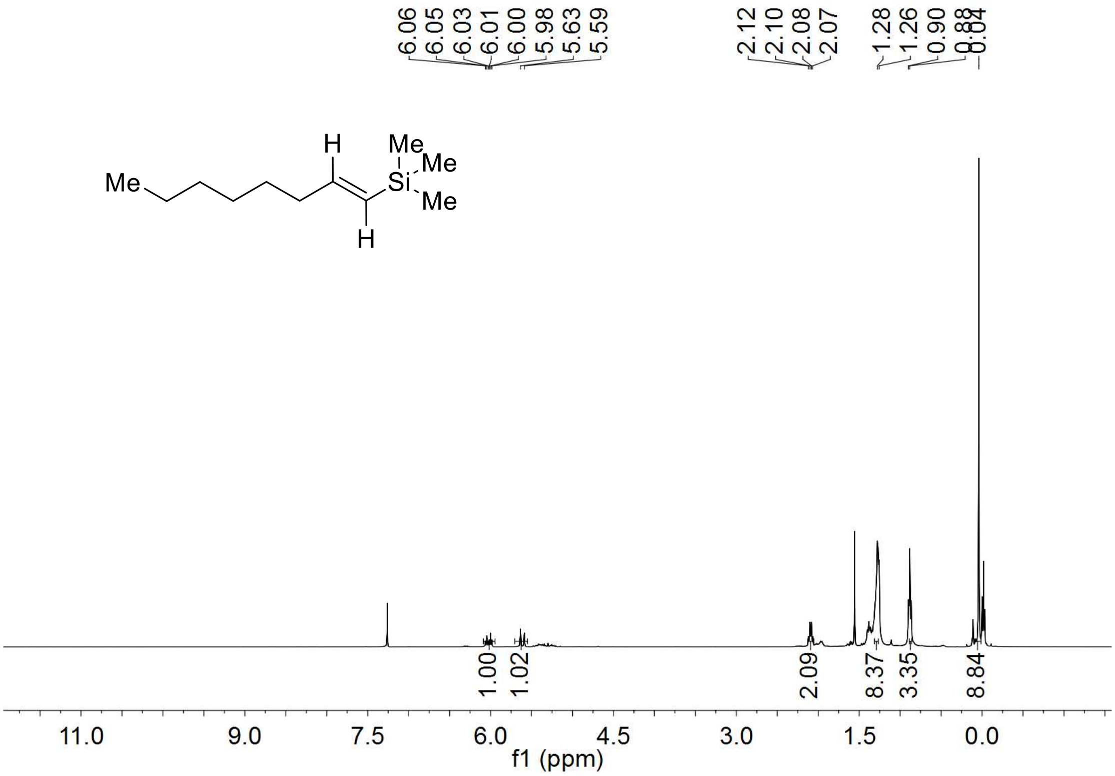

Supplementary Figure S81. ^{1}H NMR spectrum for compound *E*-3ab (400 MHz, $CDCl_3$, 25 °C).

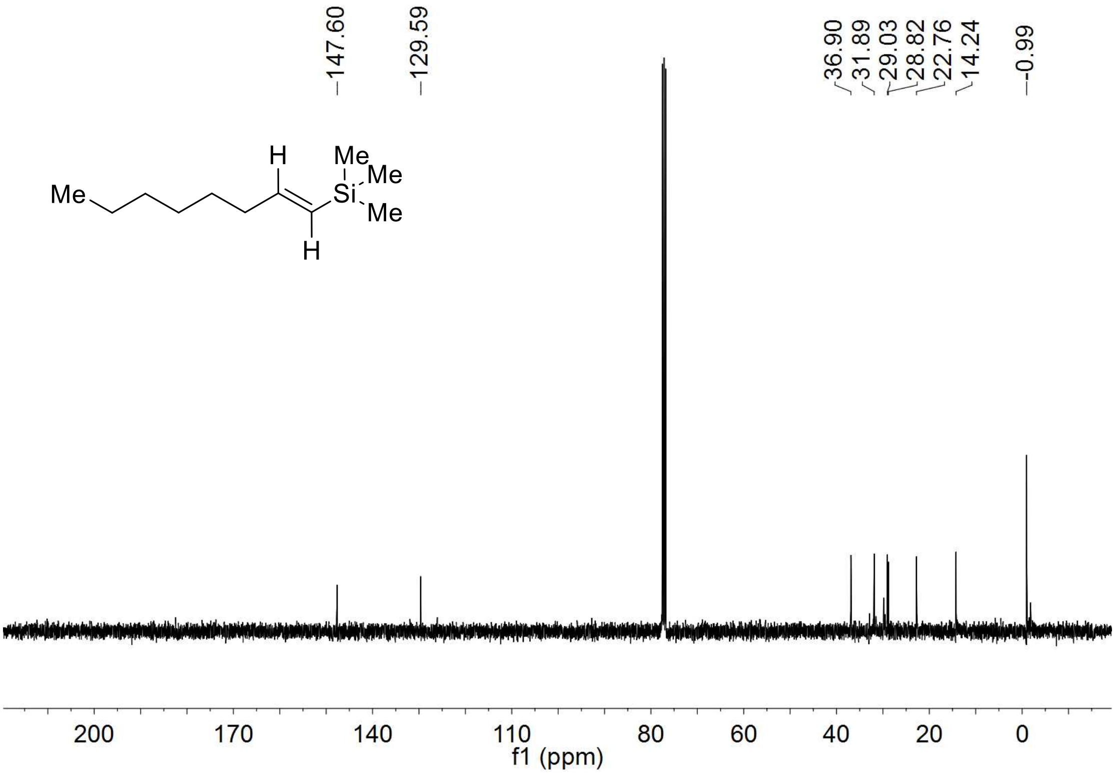

Supplementary Figure S82. ^{13}C NMR spectrum for compound *E*-3ab (100 MHz, $CDCl_3$, 25 °C).

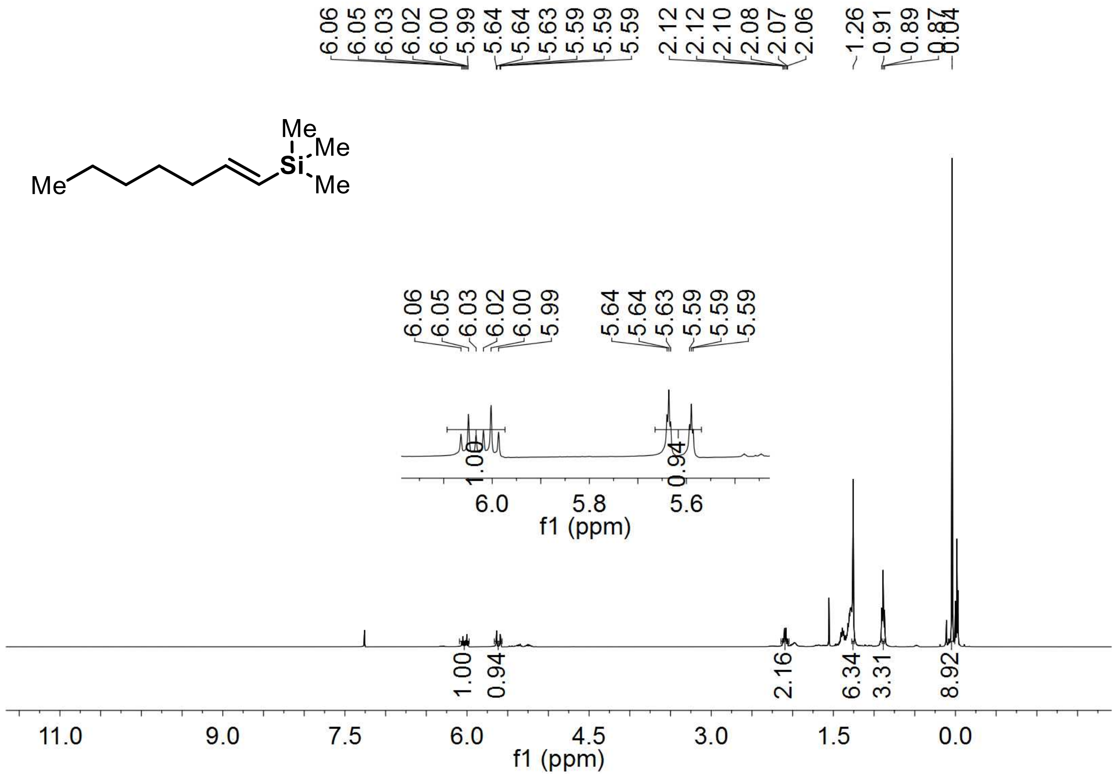

Supplementary Figure S83. ^{1}H NMR spectrum for compound *E*-3ac (400 MHz, $CDCl_3$, 25 °C).

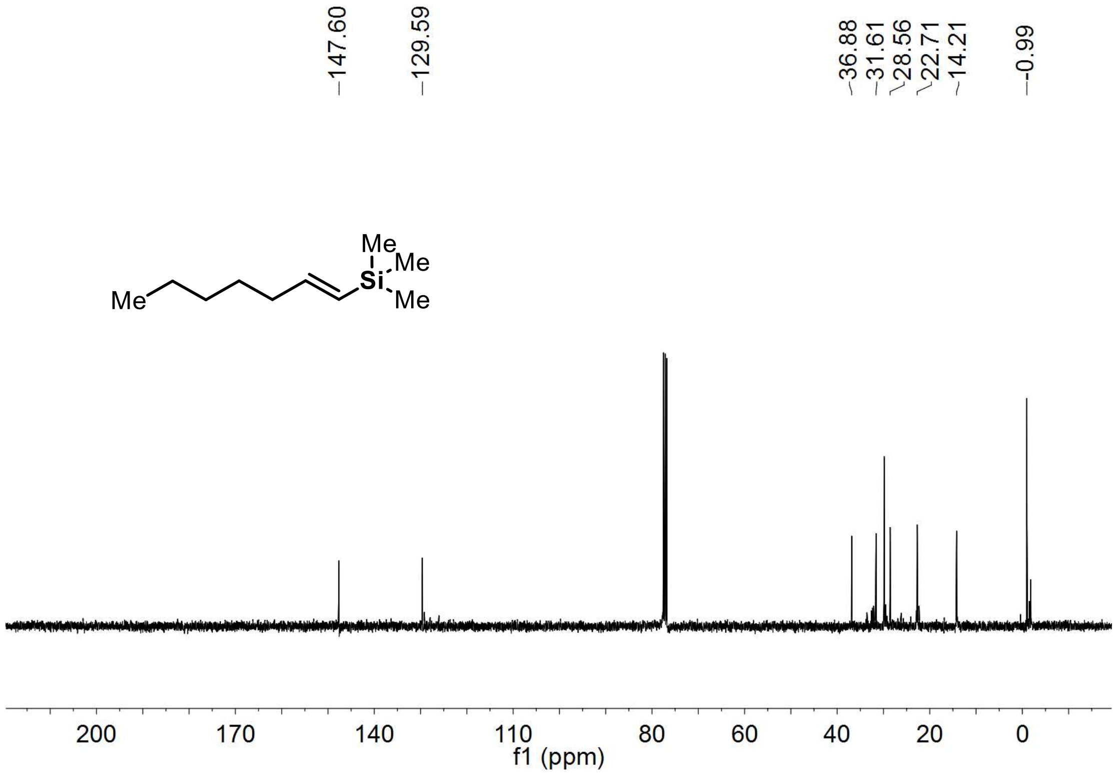

Supplementary Figure S84. ^{13}C NMR spectrum for compound *E*-3ac (100 MHz, $CDCl_3$, 25 °C).

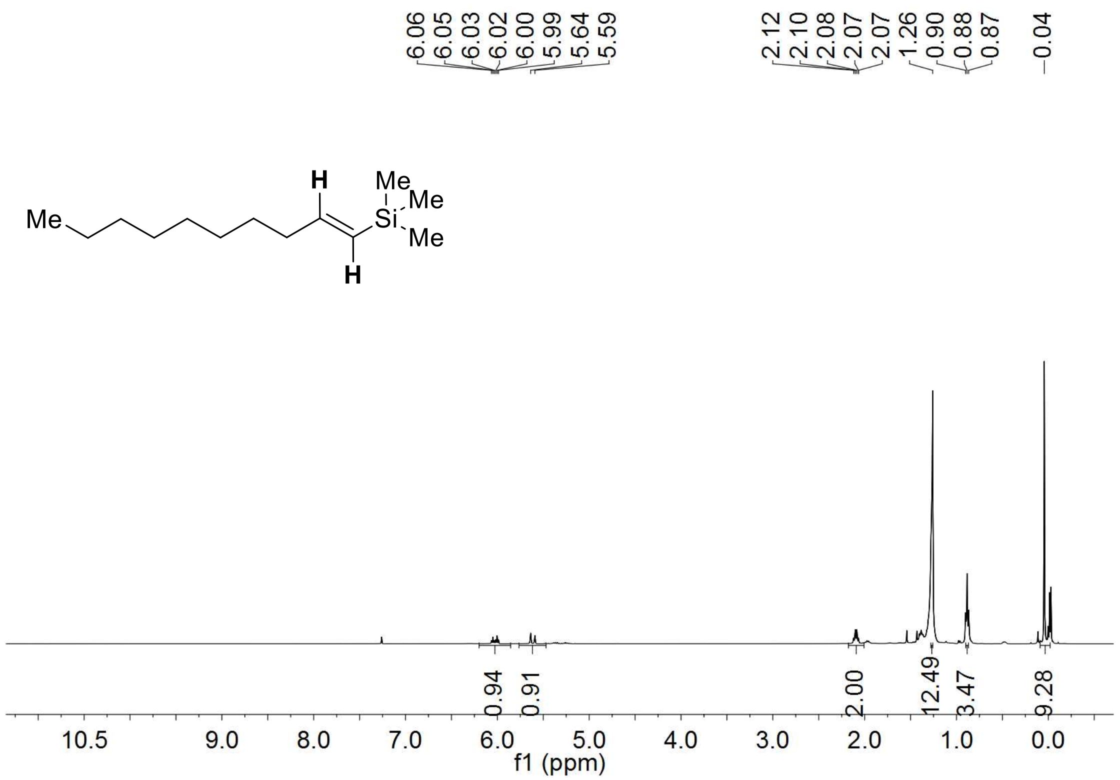

Supplementary Figure S85. ^{1}**H NMR spectrum for compound** ***E*****-3ad (400 MHz, CDCl$_3$, 25 °C).**

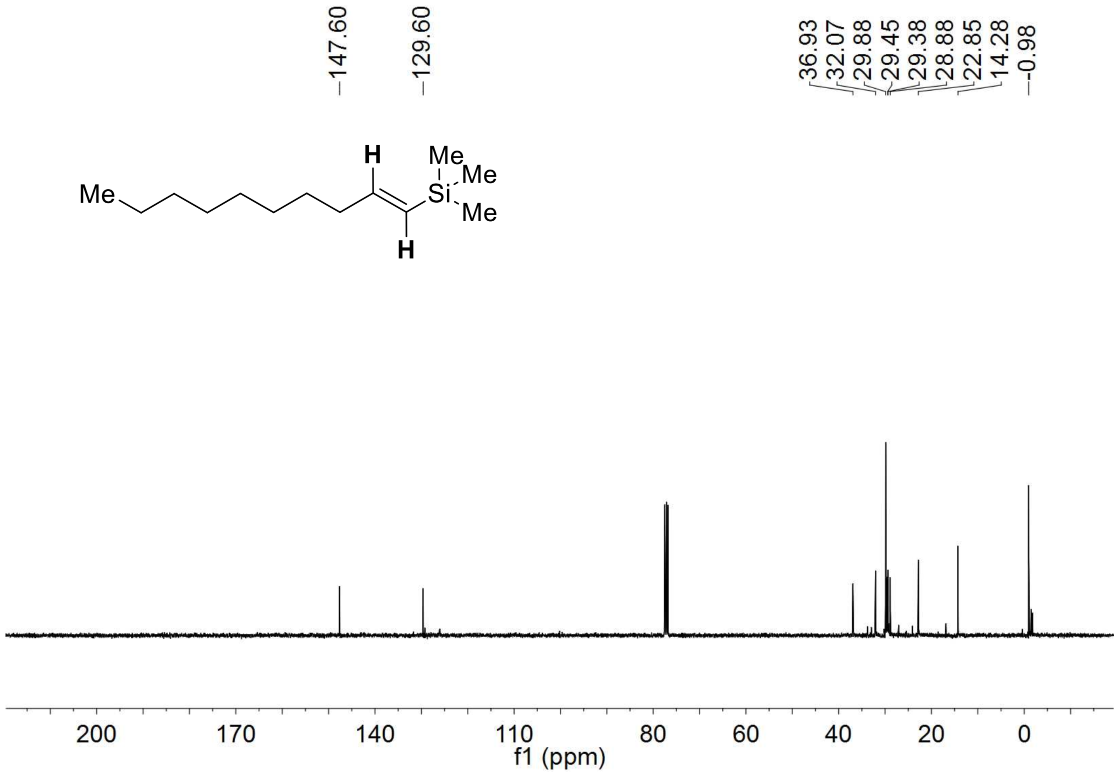

Supplementary Figure S86. ^{13}**C NMR spectrum for compound** ***E*****-3ad (100 MHz, CDCl$_3$, 25 °C).**

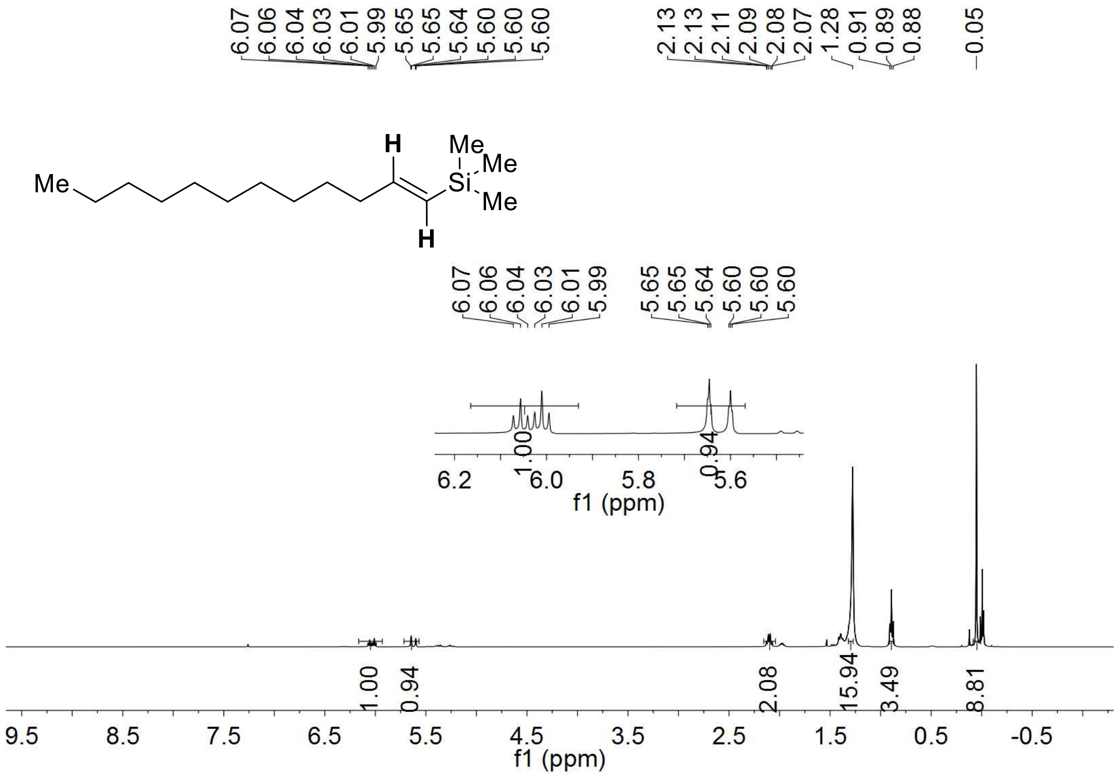

Supplementary Figure S87. ^{1}H NMR spectrum for compound *E*-3ae (400 MHz, $CDCl_3$, 25 °C).

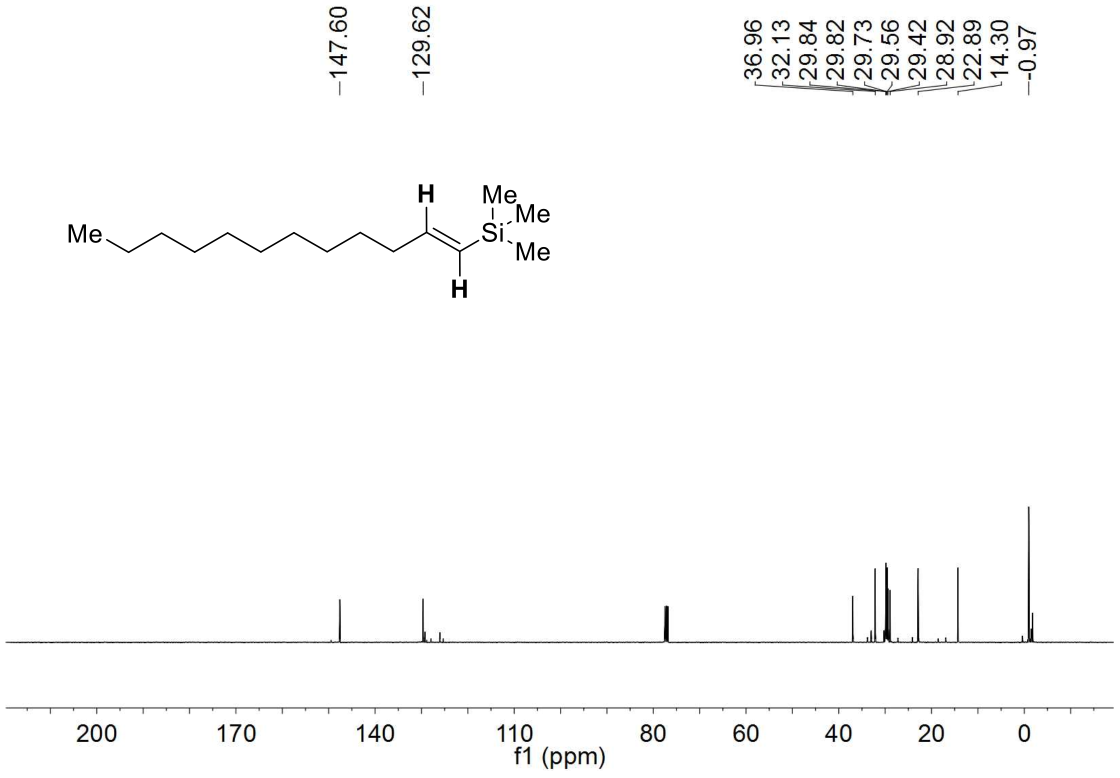

Supplementary Figure S88. ^{13}C NMR spectrum for compound *E*-3ae (100 MHz, $CDCl_3$, 25 °C).

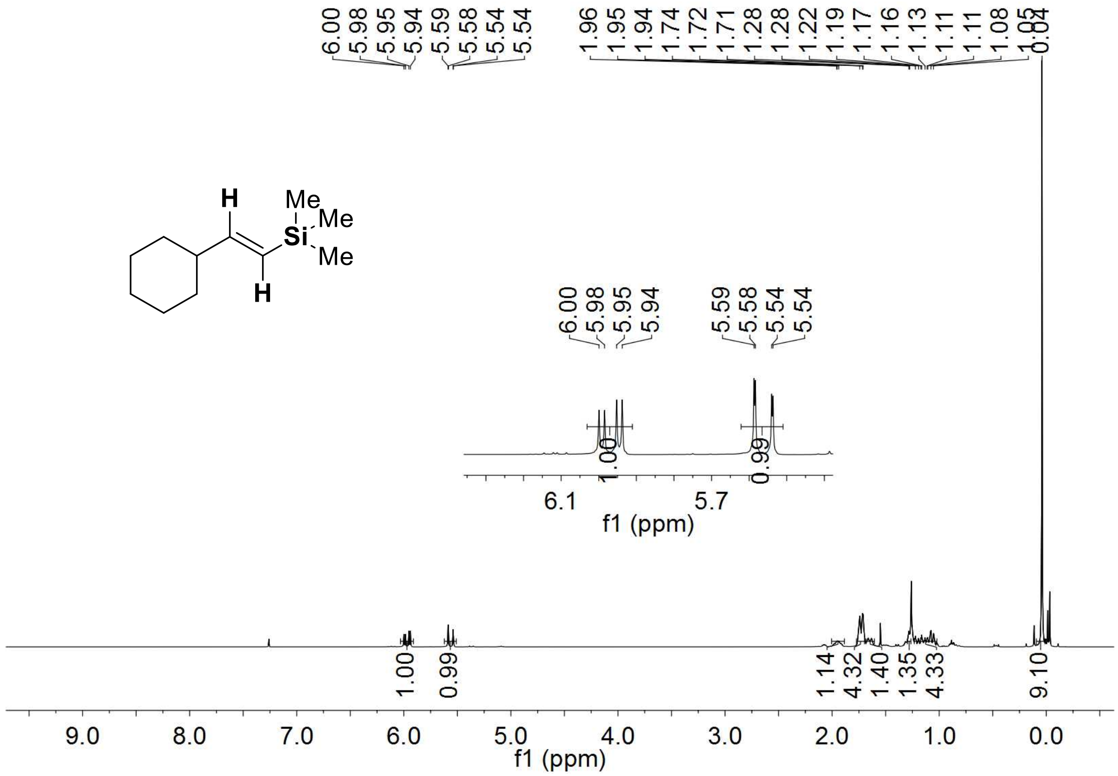

Supplementary Figure S89. ^{1}H NMR spectrum for compound *E*-3af (400 MHz, $CDCl_3$, 25 °C).

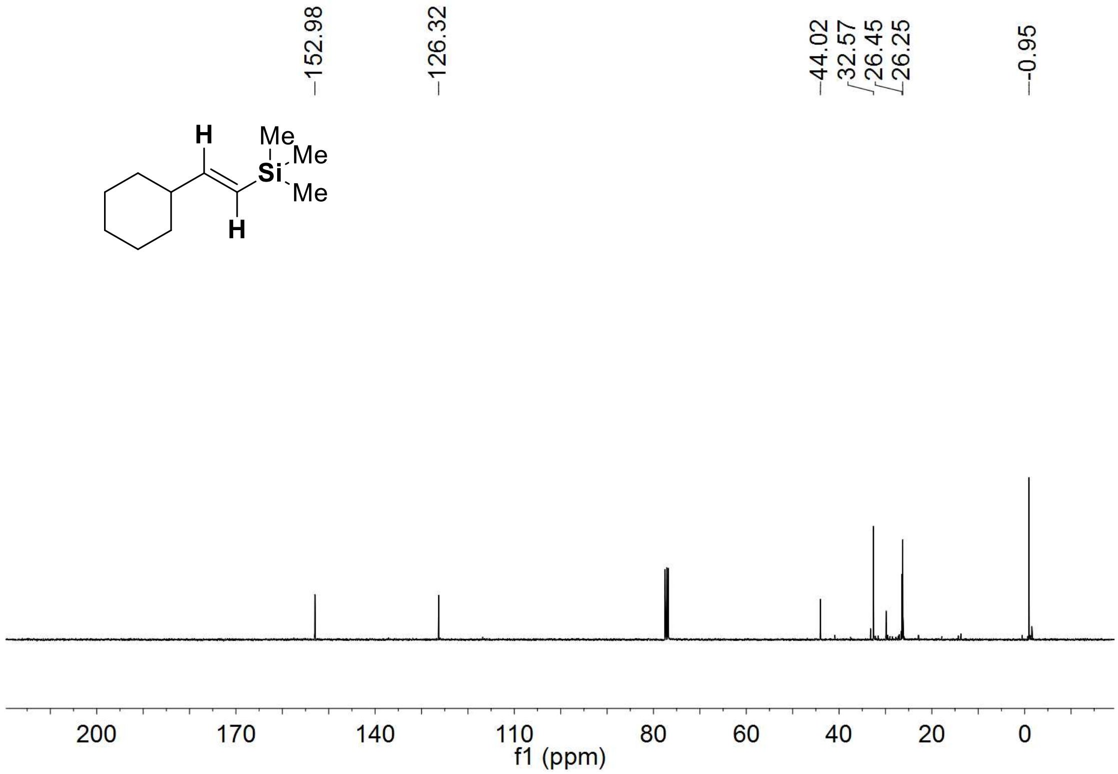

Supplementary Figure S90. ^{13}C NMR spectrum for compound *E*-3af (100 MHz, $CDCl_3$, 25 °C).

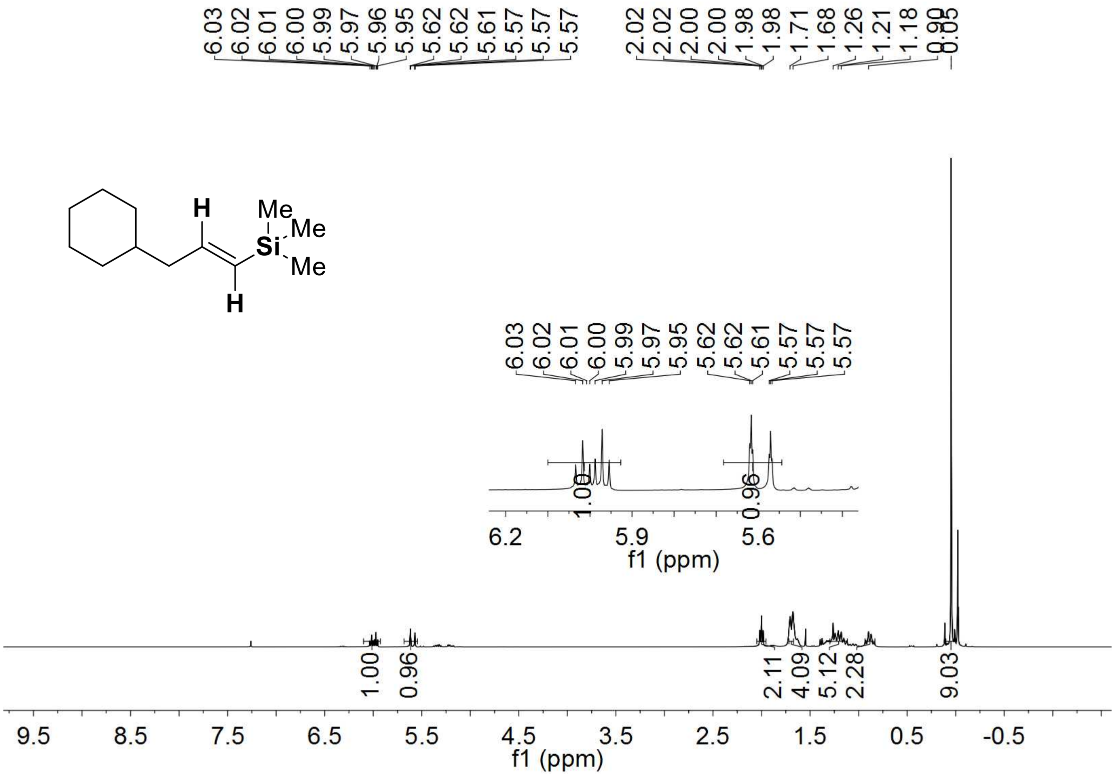

Supplementary Figure S91. ^{1}**H NMR spectrum for compound** ***E*****-3ag (400 MHz, CDCl$_3$, 25 °C).**

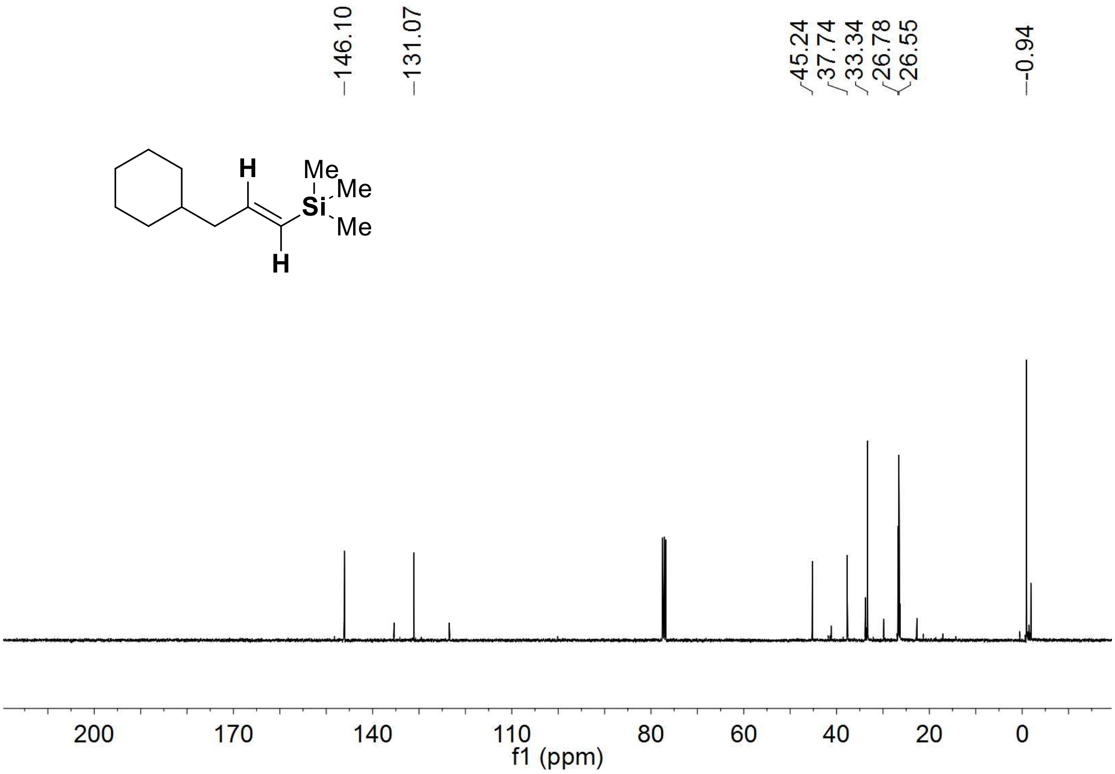

Supplementary Figure S92. ^{13}**C NMR spectrum for compound** ***E*****-3ag (100 MHz, CDCl$_3$, 25 °C).**

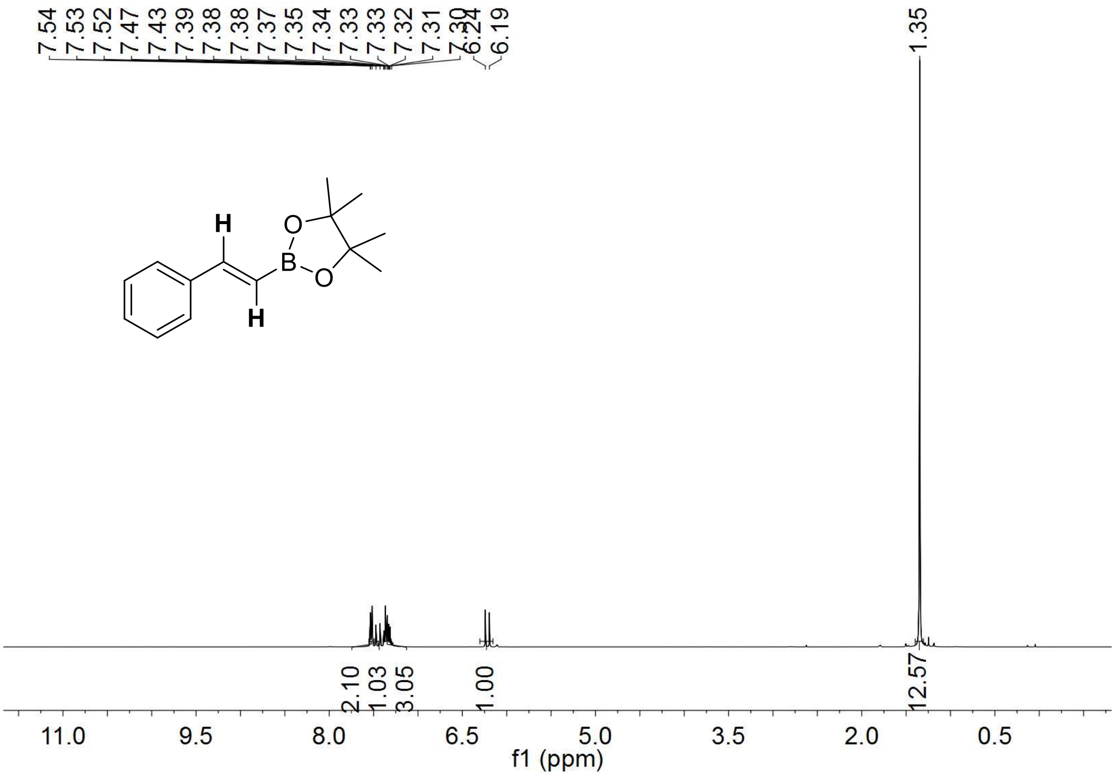

Supplementary Figure S93. ^{1}H NMR spectrum for compound *E*-3ah (400 MHz, $CDCl_3$, 25 ^{o}C).

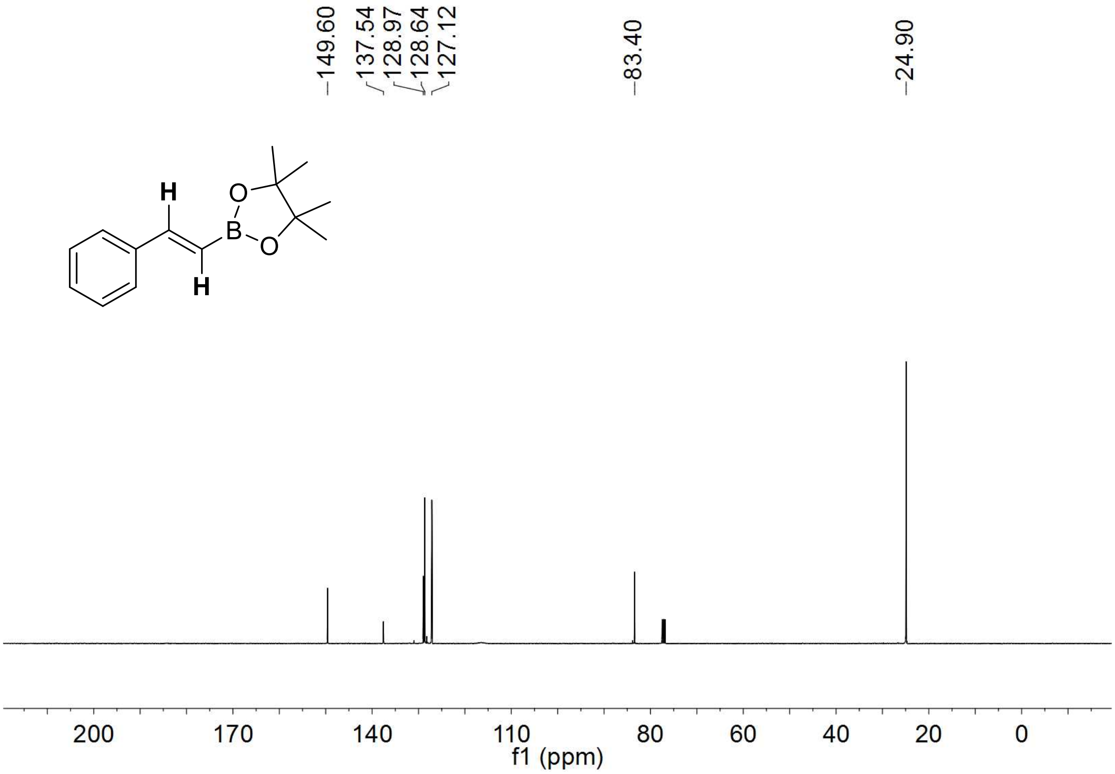

Supplementary Figure S94. ^{13}C NMR spectrum for compound *E*-3ah (100 MHz, $CDCl_3$, 25 ^{o}C).

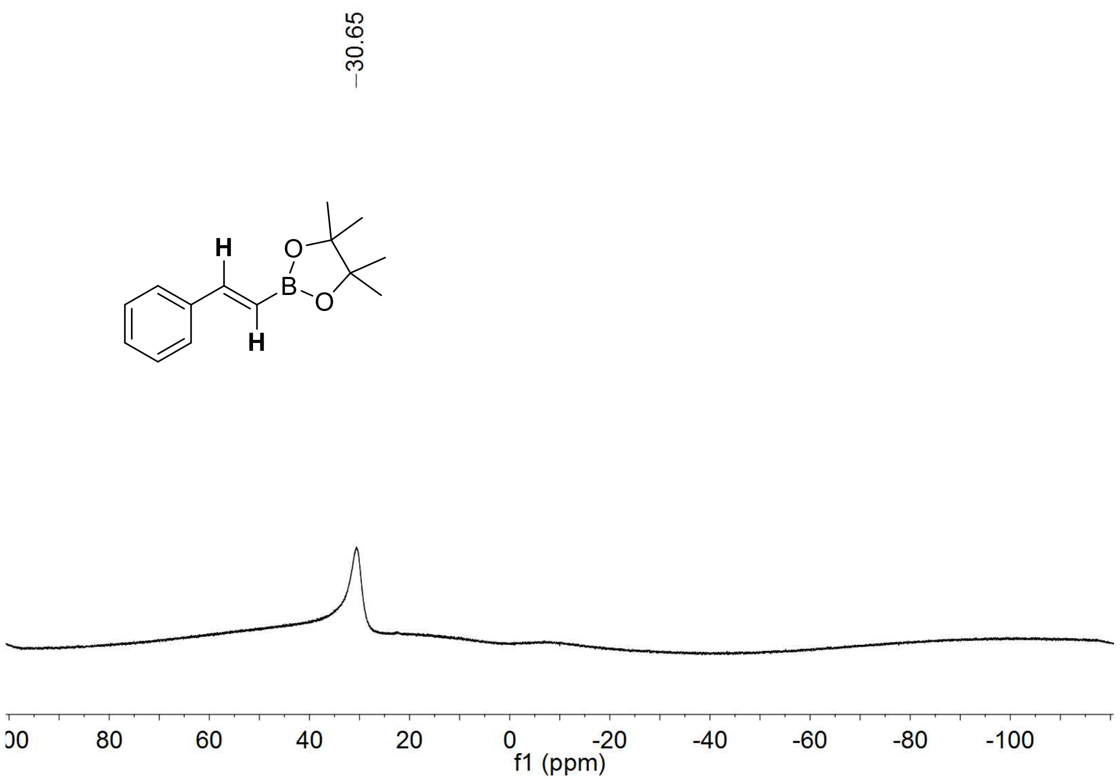

Supplementary Figure S95. ^{11}B NMR spectrum for compound *E*-3ah (128 MHz, $CDCl_3$, 25 °C).

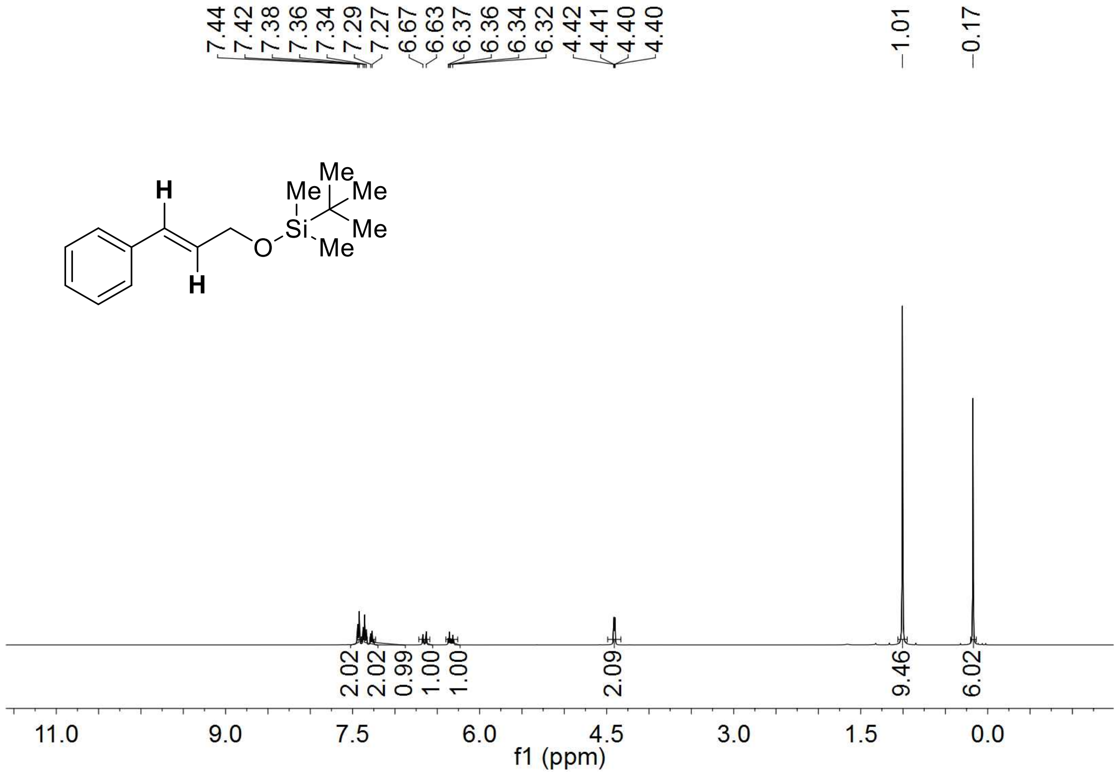

Supplementary Figure S96. ^{1}H NMR spectrum for compound *E*-3ai (400 MHz, $CDCl_3$, 25 °C).

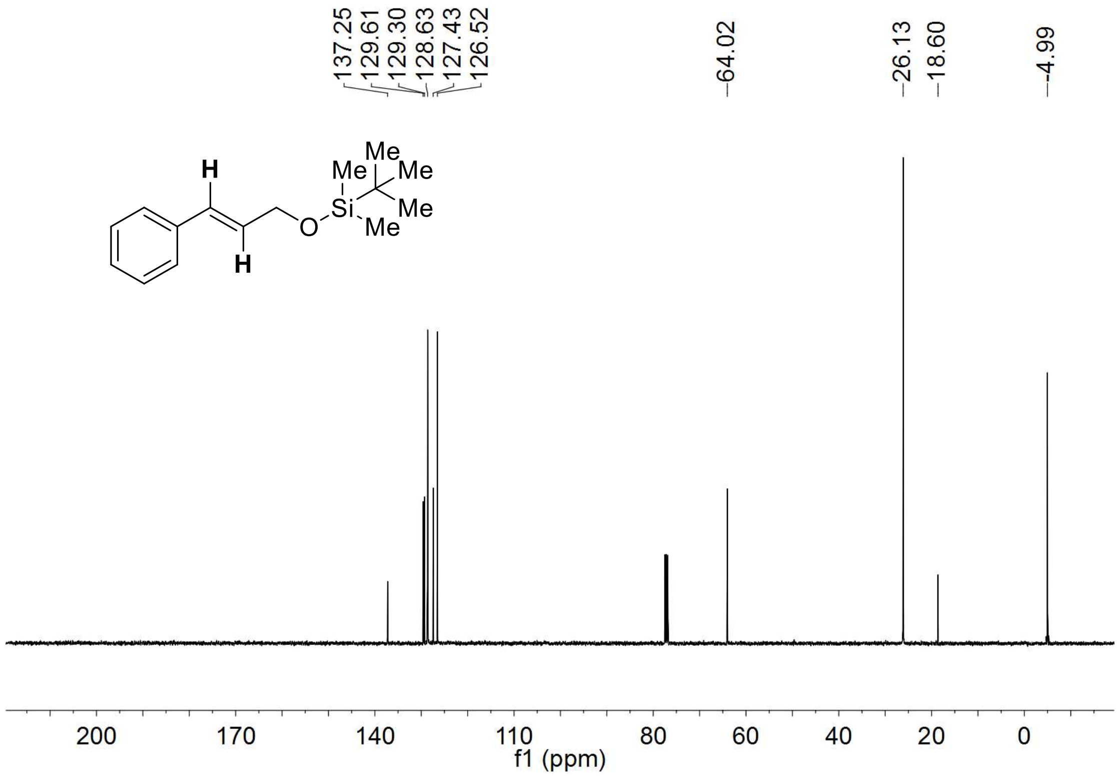

Supplementary Figure S97. ^{13}**C NMR spectrum for compound** ***E*****-3ai (100 MHz, CDCl$_3$, 25 °C).**

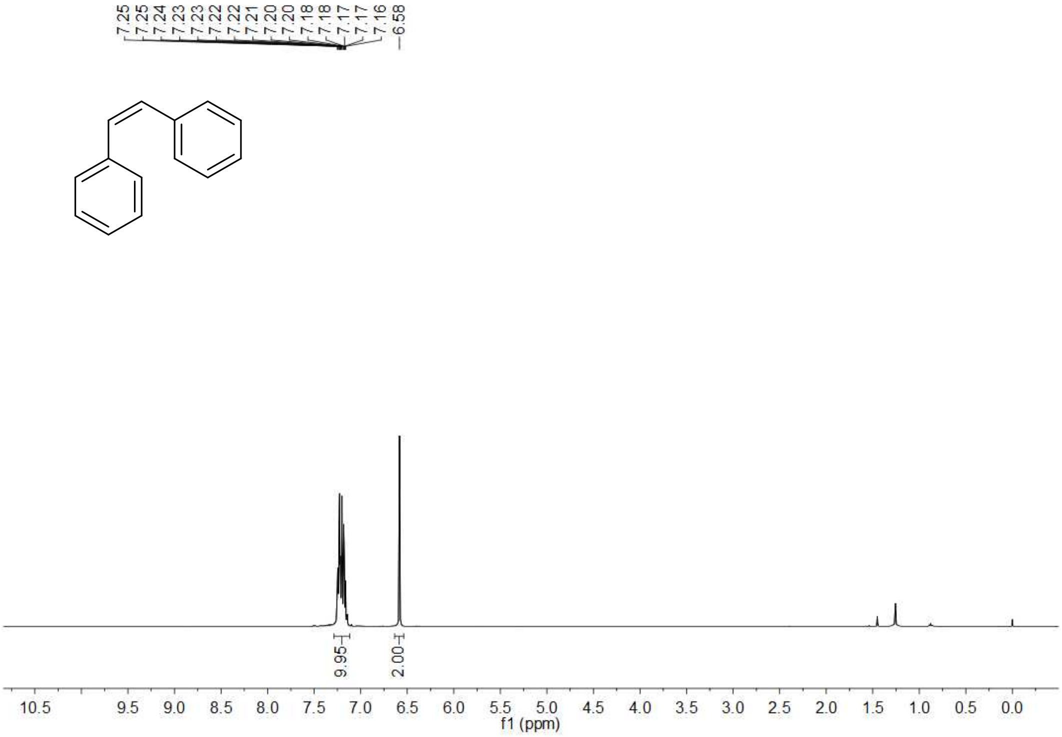

Supplementary Figure S98. ^{1}**H NMR spectrum for compound** ***Z*****-3a (400 MHz, CDCl$_3$, 25 °C).**

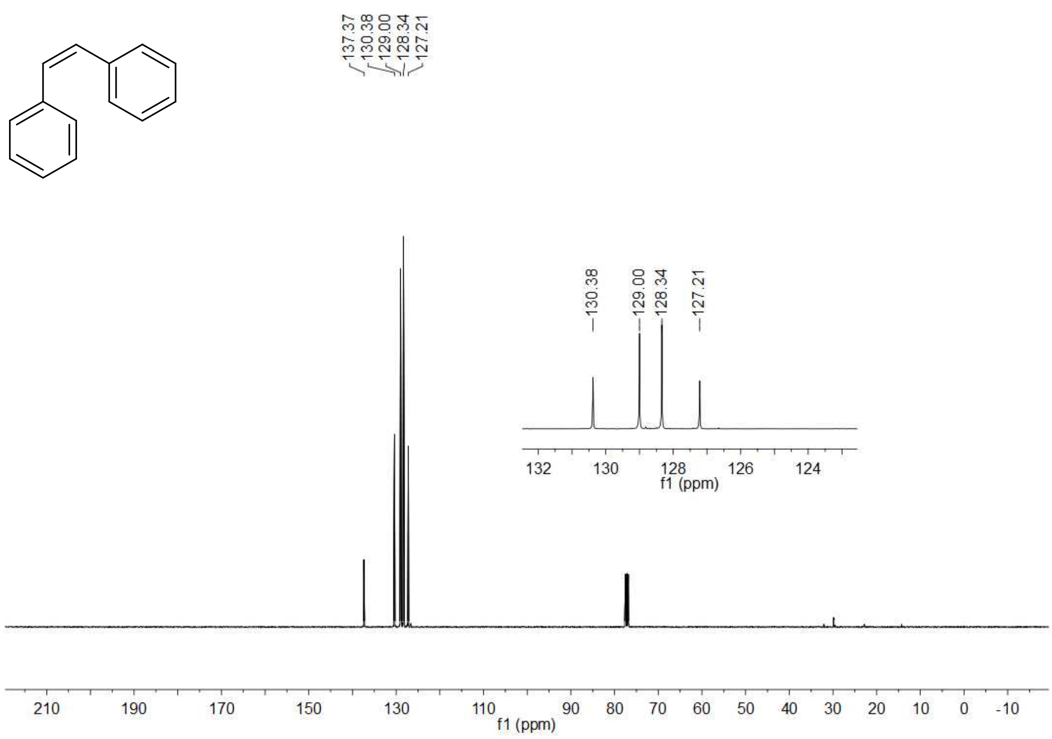

Supplementary Figure S99. ^{13}C NMR spectrum for compound *Z*-3a (100 MHz, $CDCl_3$, 25 °C).

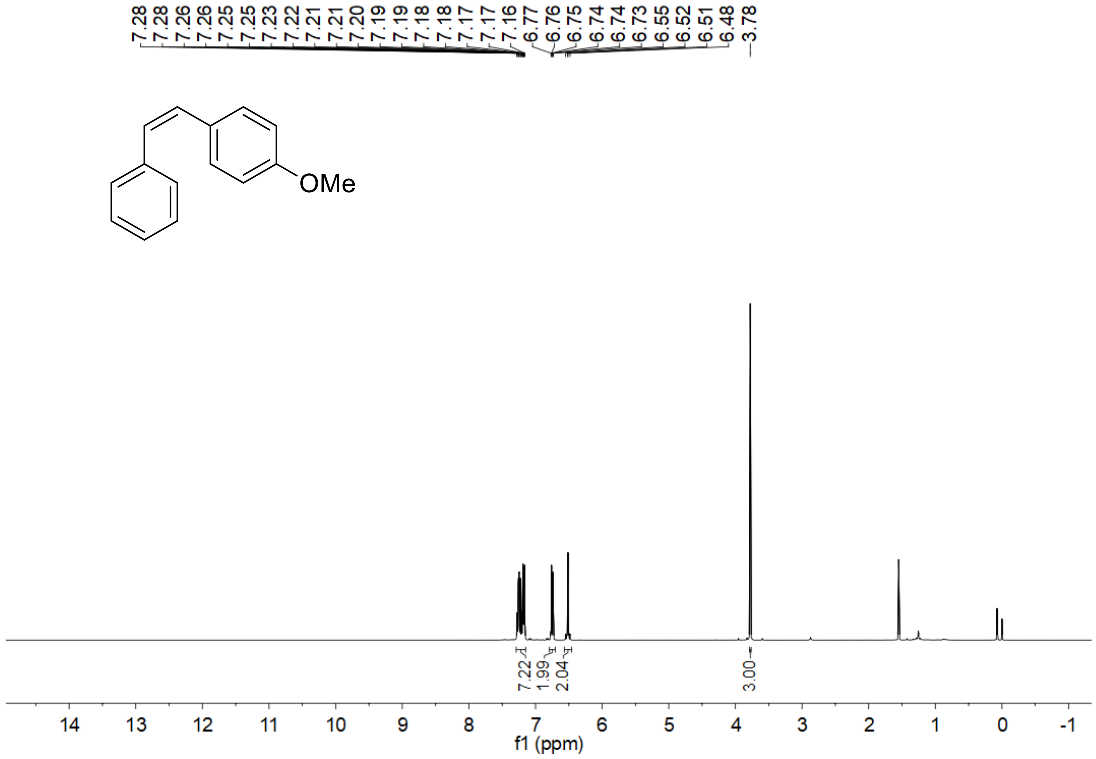

Supplementary Figure S100. ^{1}H NMR spectrum for compound *Z*-3j (400 MHz, $CDCl_3$, 25 °C).

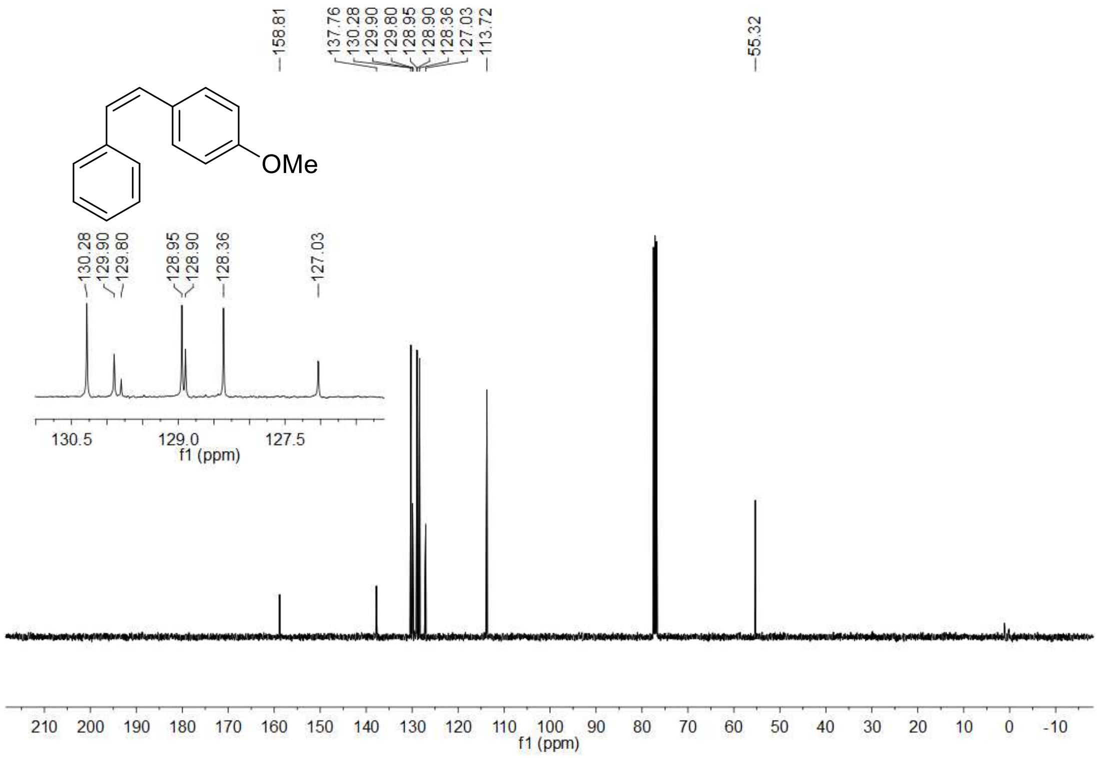

Supplementary Figure S101. ^{13}**C NMR spectrum for compound *Z*-3j (100 MHz, $CDCl_3$, 25 °C).**

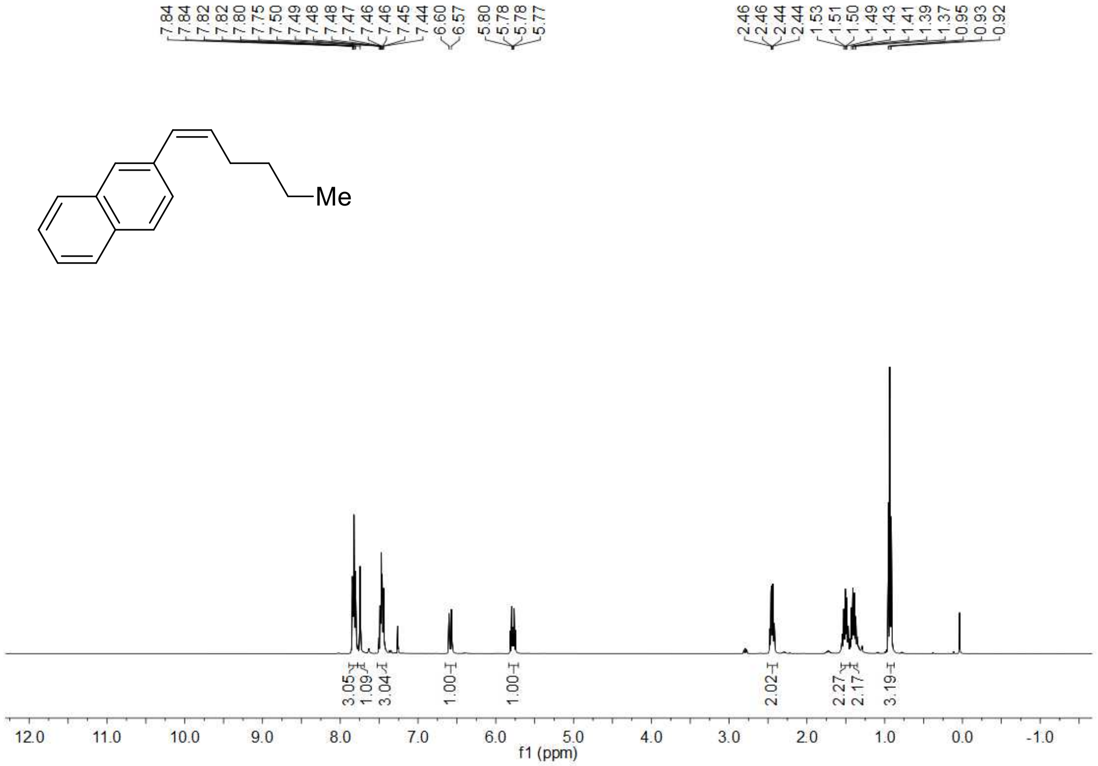

Supplementary Figure S102. ^{1}**H NMR spectrum for compound *Z*-3b (400 MHz, $CDCl_3$, 25 °C).**

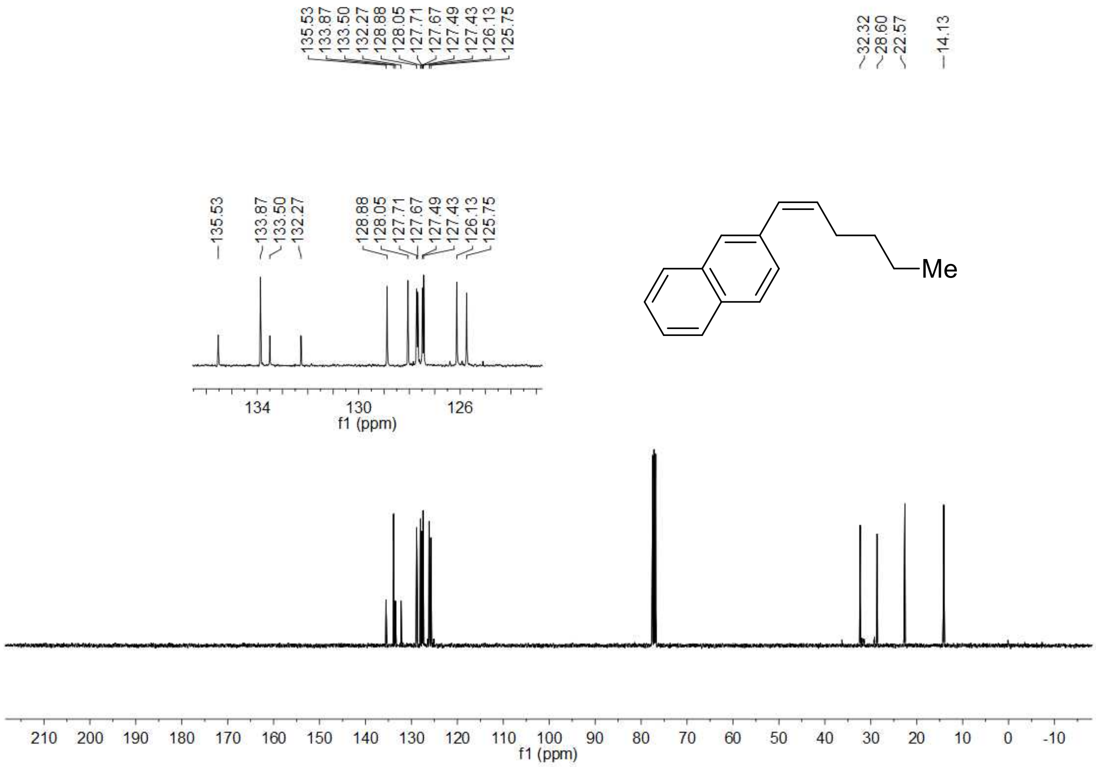

Supplementary Figure S103. ^{13}C NMR spectrum for compound *Z*-3b (100 MHz, $CDCl_3$, 25 °C).

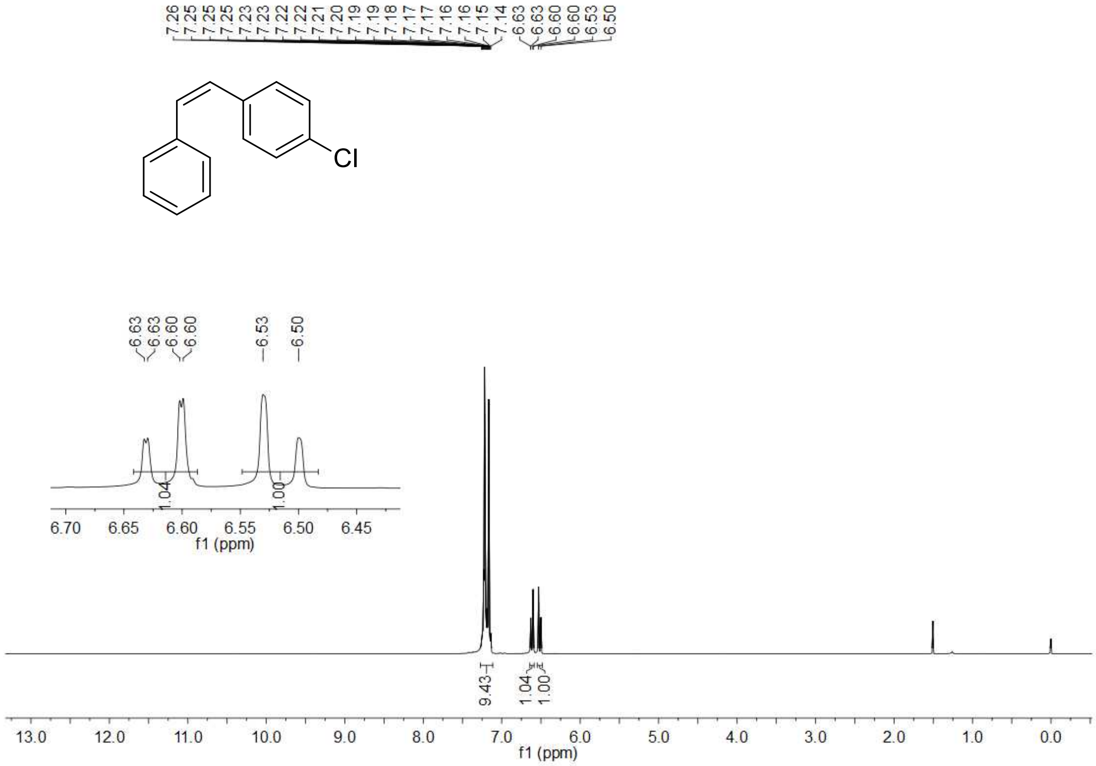

Supplementary Figure S104. ^{1}H NMR spectrum for compound *Z*-3aj (400 MHz, $CDCl_3$, 25 °C).

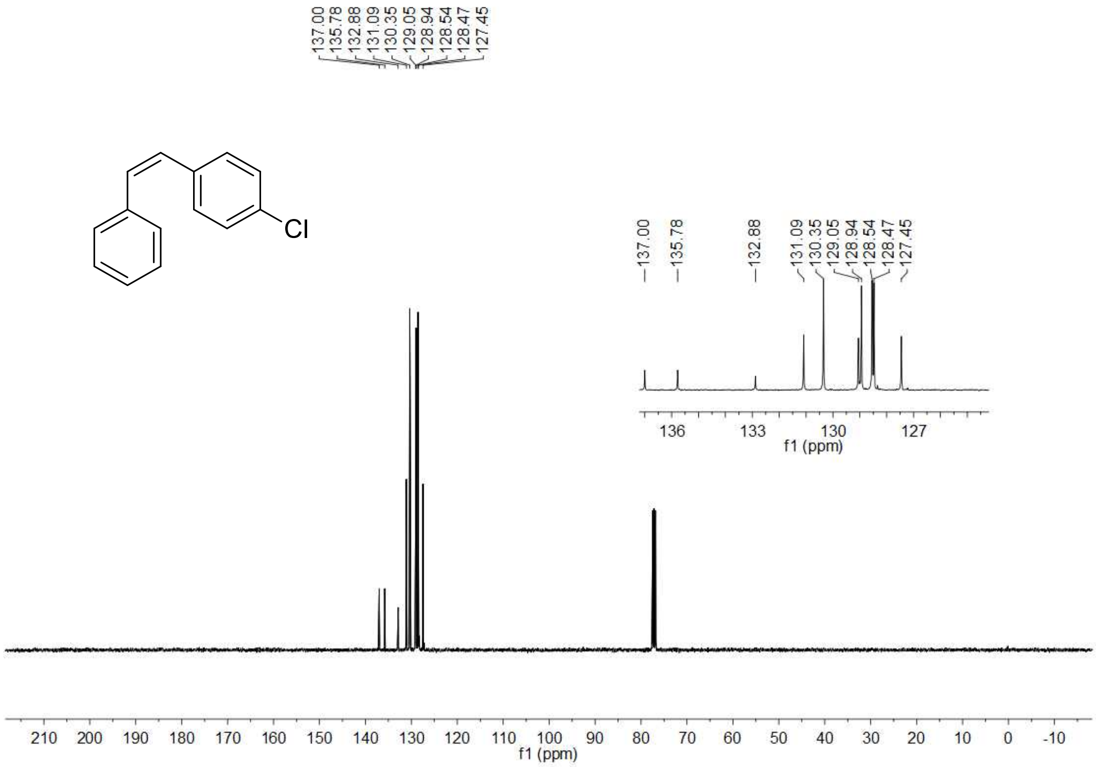

Supplementary Figure S105. ^{13}C NMR spectrum for compound *Z*-3aj (100 MHz, $CDCl_3$, 25 °C).

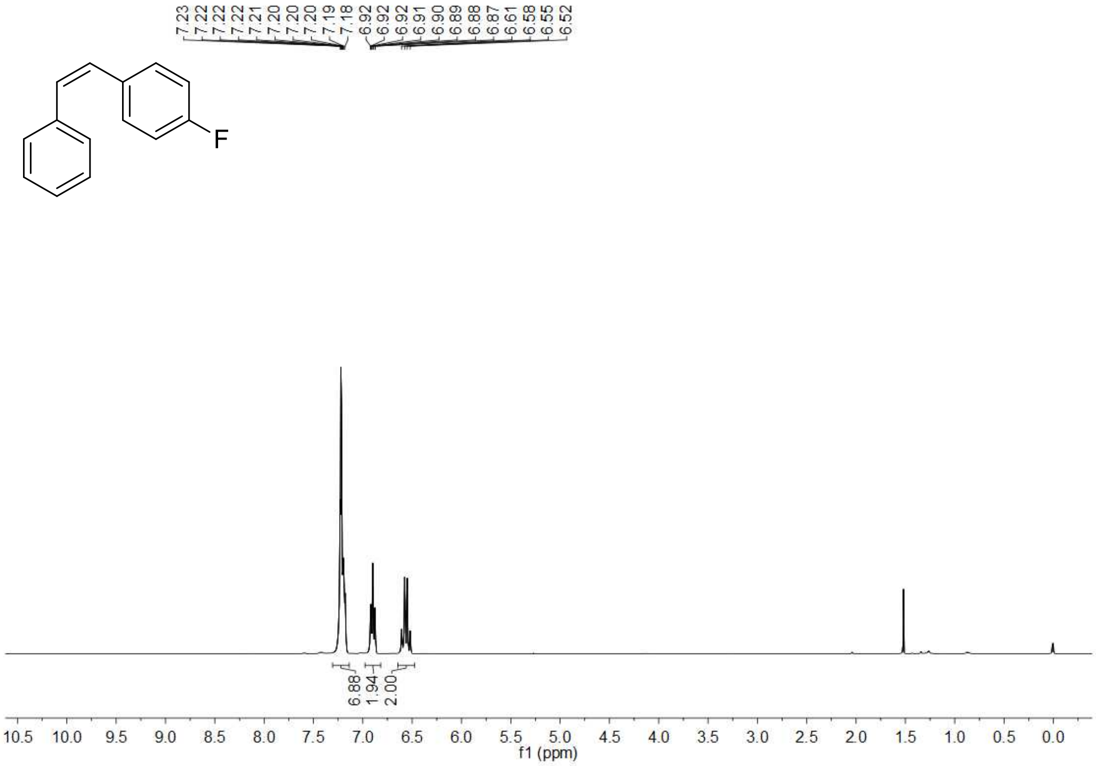

Supplementary Figure S106. ^{1}H NMR spectrum for compound *Z*-3ak (400 MHz, $CDCl_3$, 25 °C).

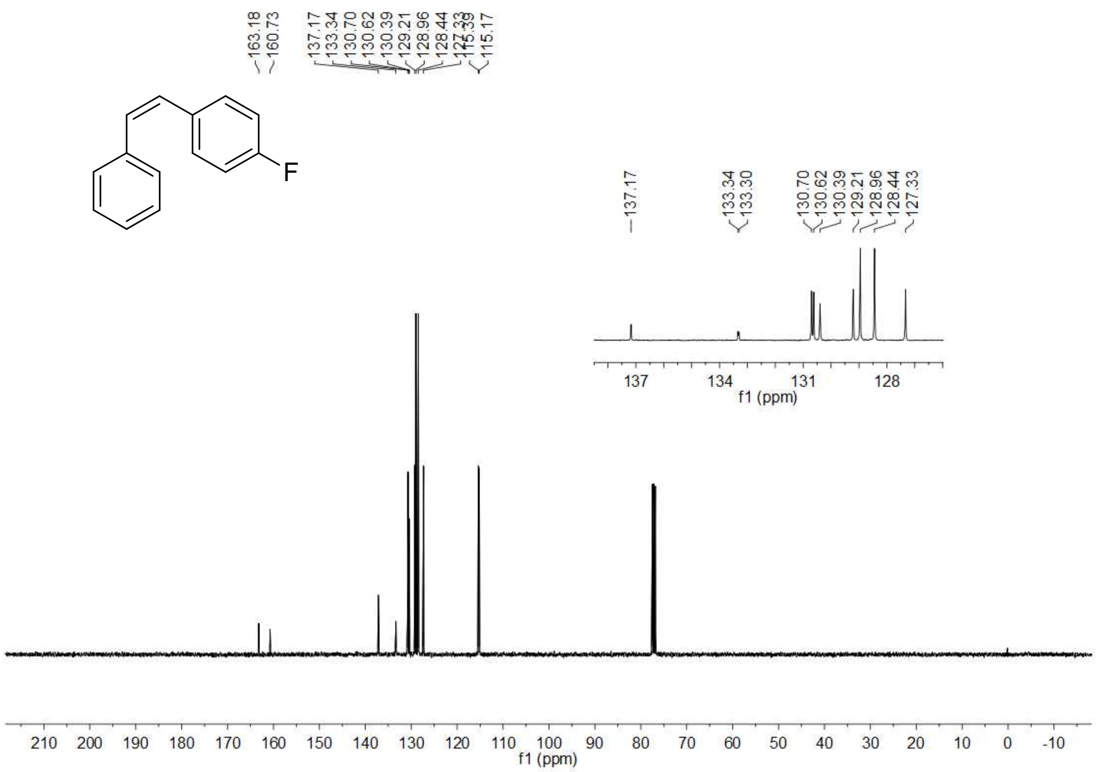

Supplementary Figure S107. ^{13}C NMR spectrum for compound *Z*-3ak (100 MHz, $CDCl_3$, 25 °C).

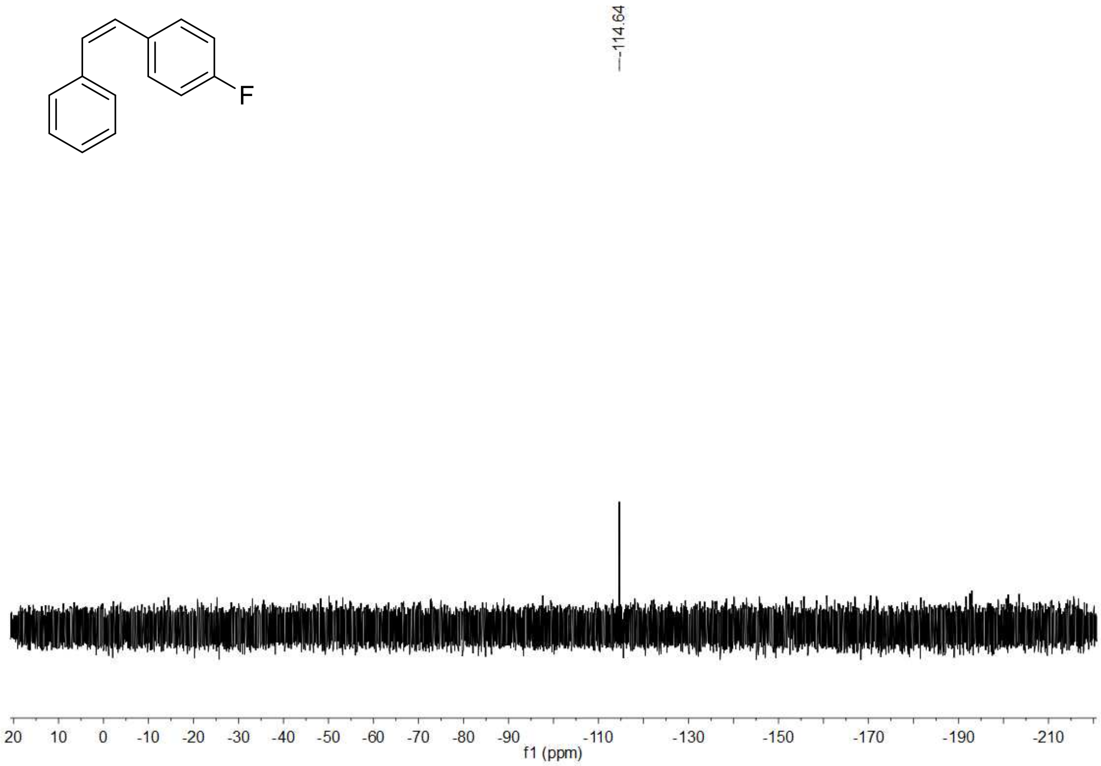

Supplementary Figure S108. ^{19}F NMR spectrum for compound *Z*-3ak (376 MHz, $CDCl_3$, 25 °C).

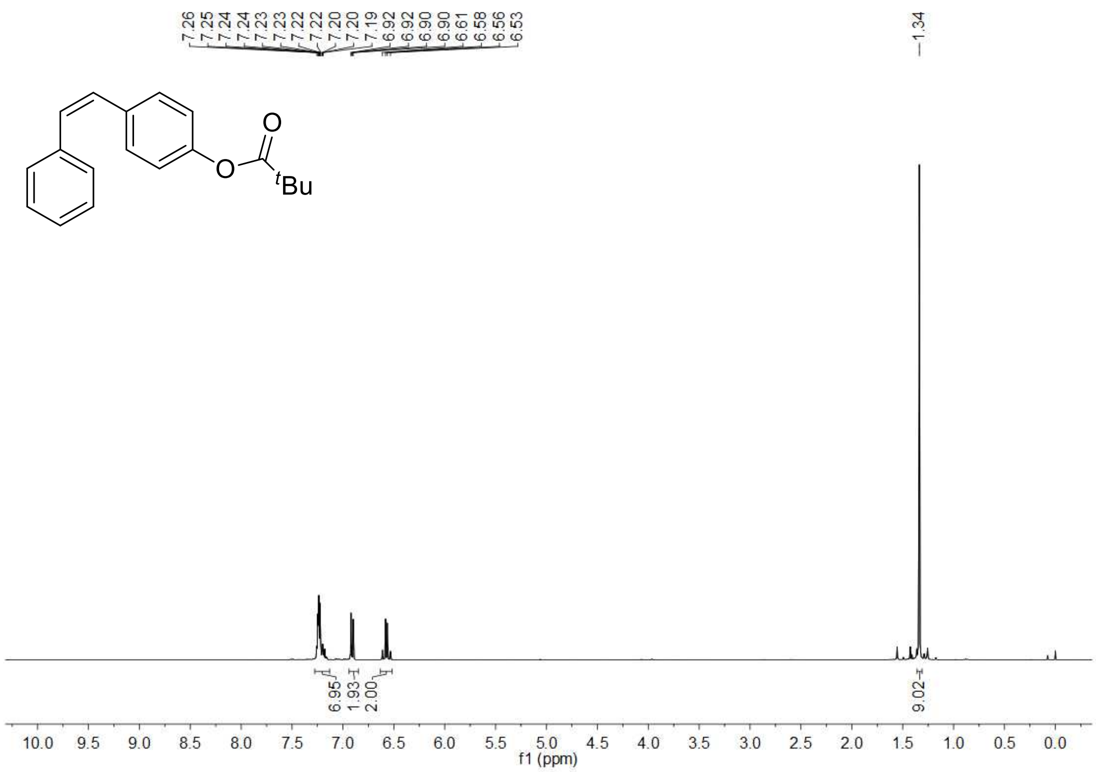

Supplementary Figure S109. ^{1}H NMR spectrum for compound *Z*-3al (400 MHz, $CDCl_3$, 25 °C).

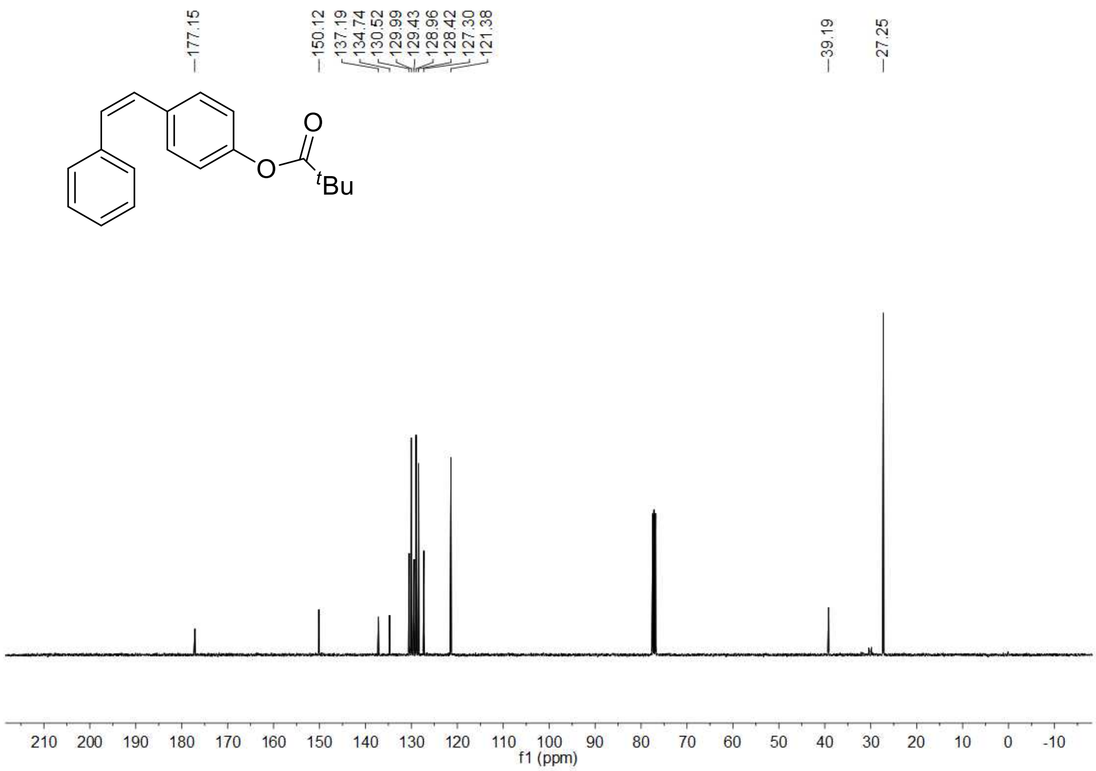

Supplementary Figure S110. ^{13}C NMR spectrum for compound *Z*-3al (100 MHz, $CDCl_3$, 25 °C).

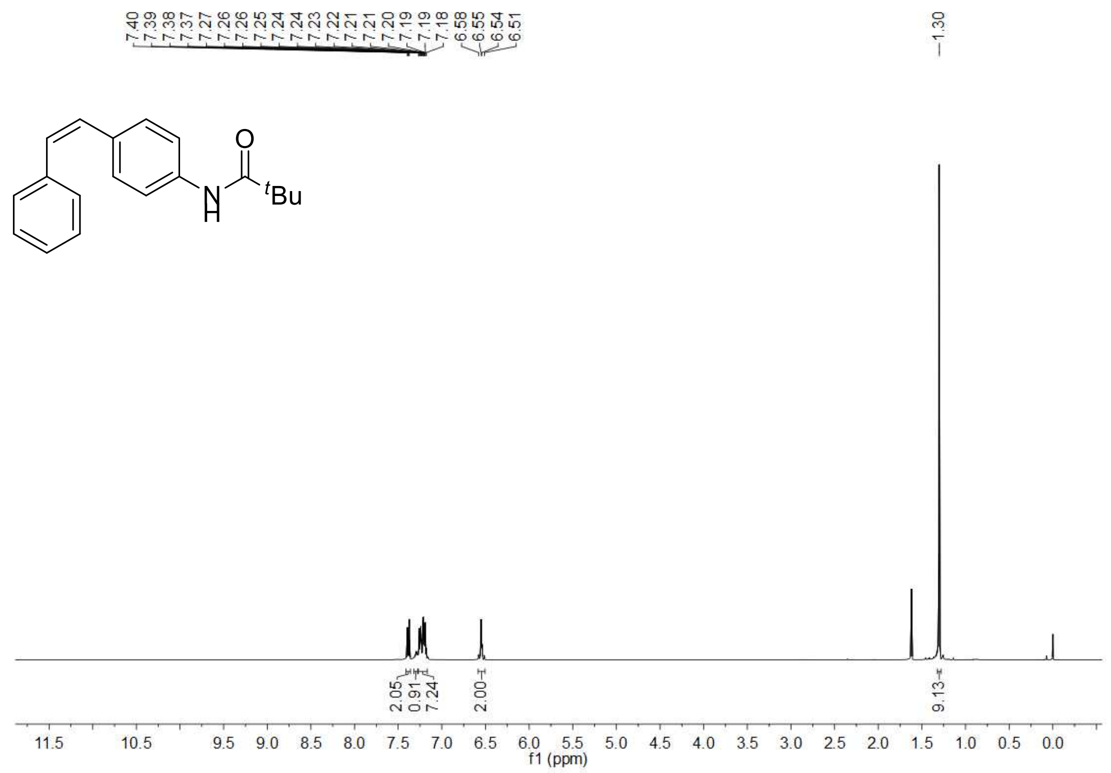

Supplementary Figure S111. ^{1}H NMR spectrum for compound *Z*-3am (400 MHz, $CDCl_3$, 25 ^{o}C).

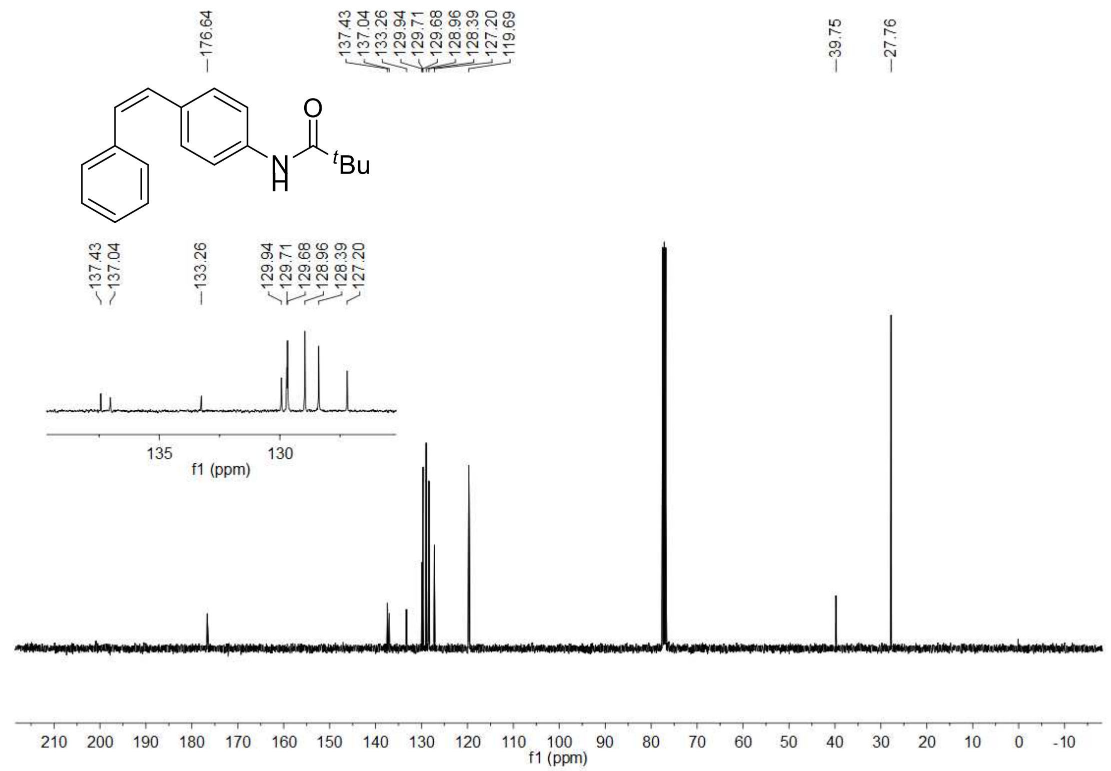

Supplementary Figure S112. ^{13}C NMR spectrum for compound *Z*-3am (100 MHz, $CDCl_3$, 25 ^{o}C).

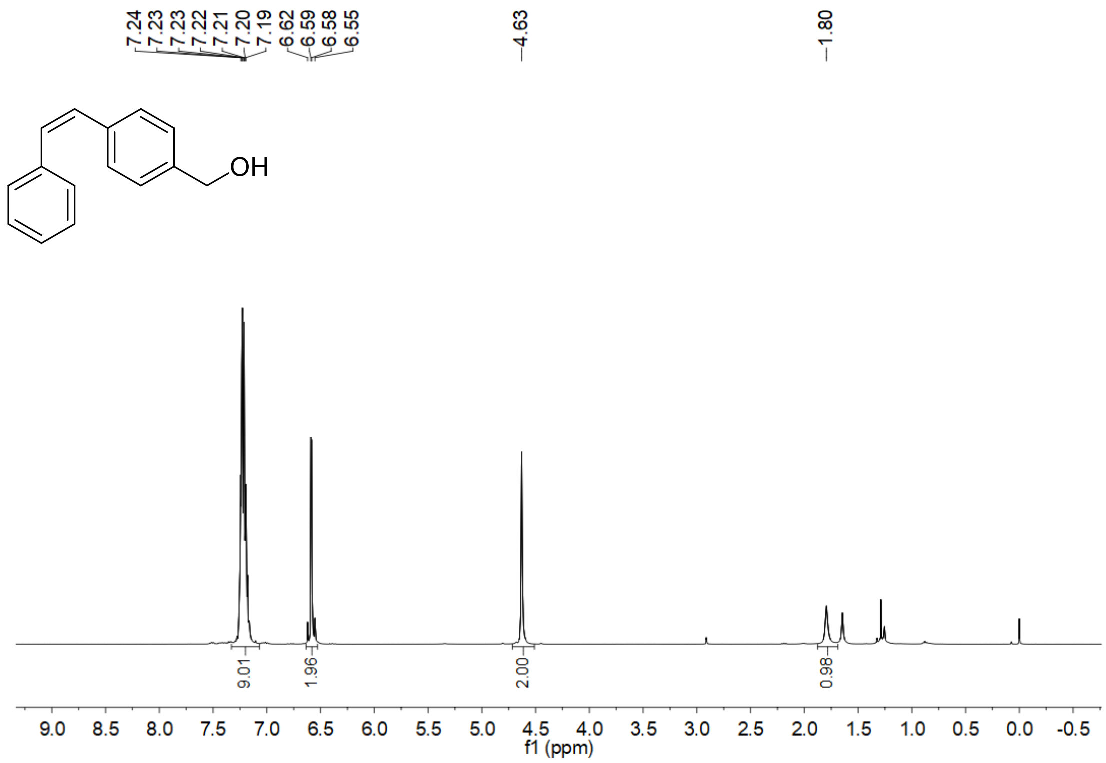

Supplementary Figure S113. ¹H NMR spectrum for compound *Z*-3an (400 MHz, $CDCl_3$, 25 °C).

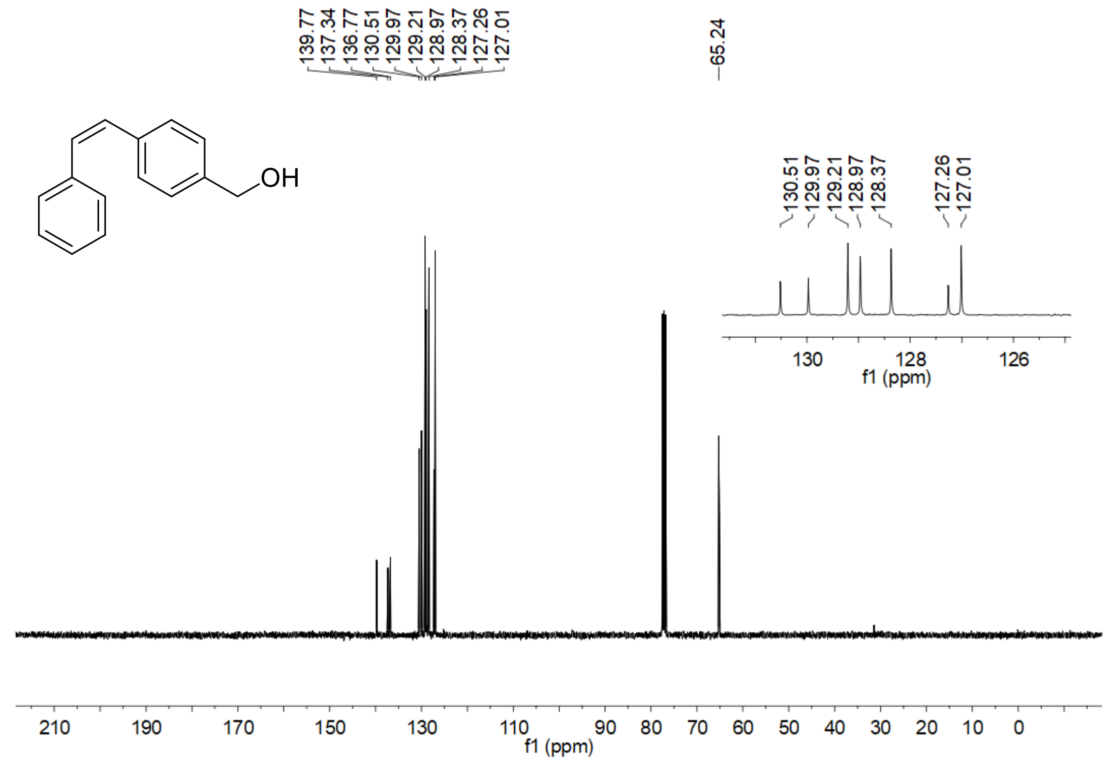

Supplementary Figure S114. ¹³C NMR spectrum for compound *Z*-3an (100 MHz, $CDCl_3$, 25 °C).

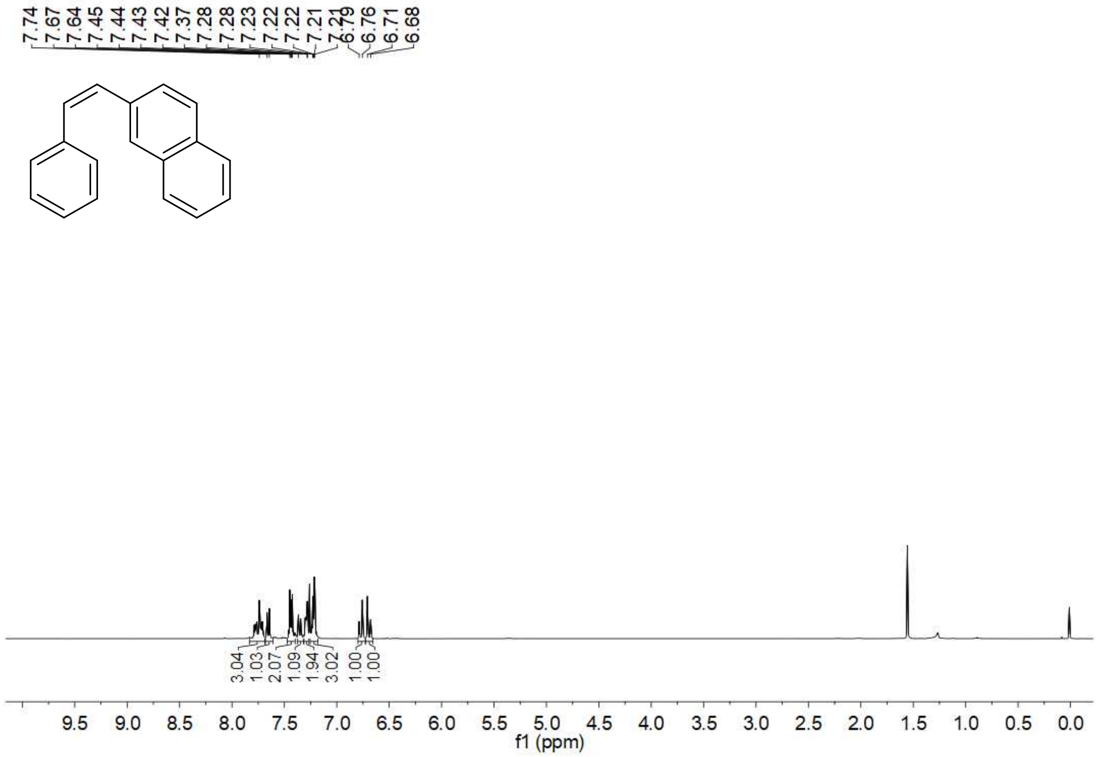

Supplementary Figure S115. ^{1}H NMR spectrum for compound *Z*-3ao (400 MHz, $CDCl_3$, 25 °C).

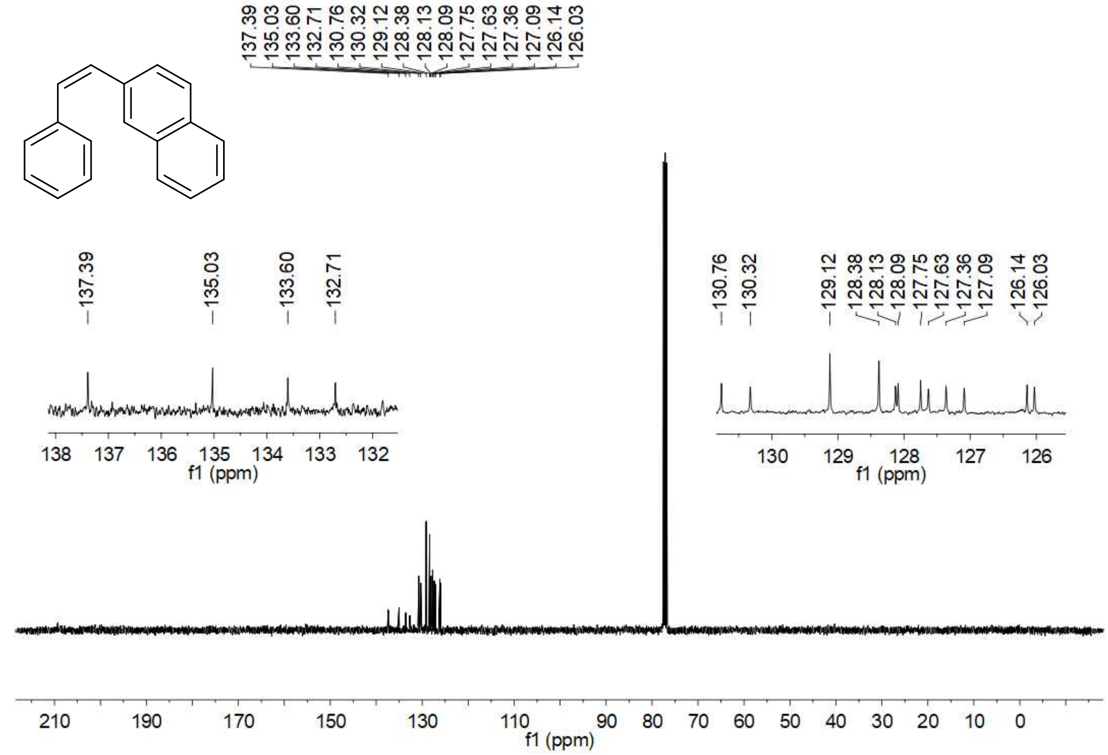

Supplementary Figure S116. ^{13}C NMR spectrum for compound Z-3ao (100 MHz, $CDCl_3$, 25 °C).

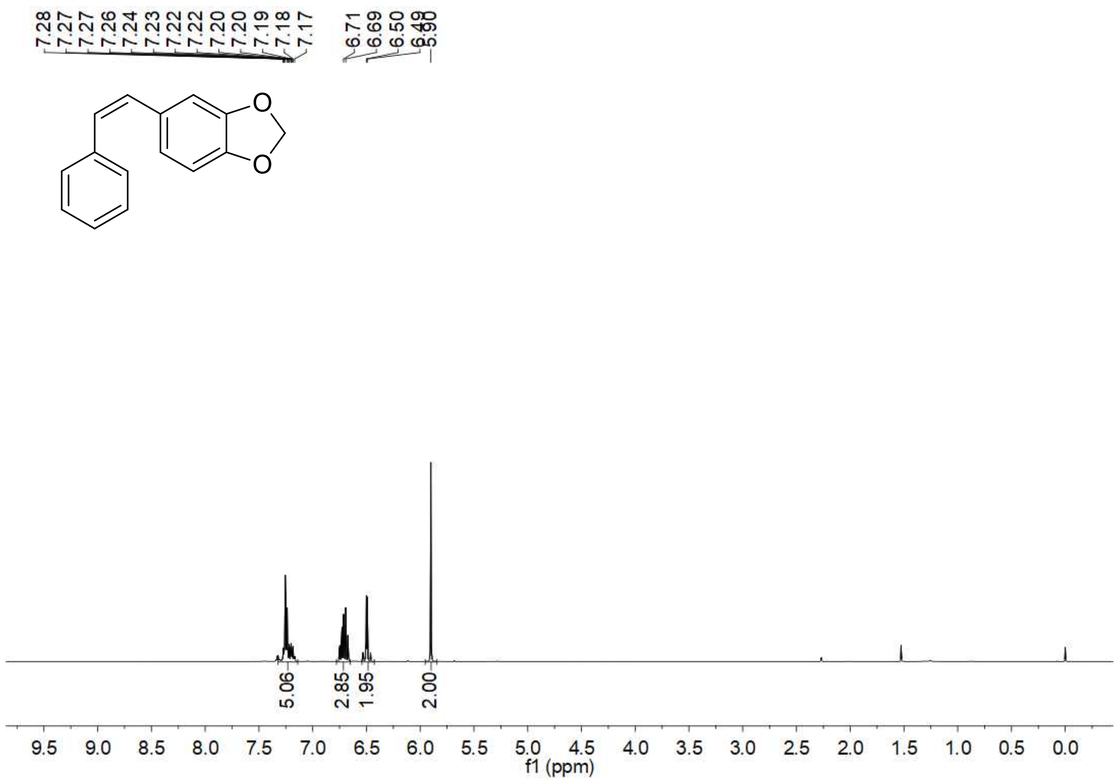

Supplementary Figure S117. ^{1}H NMR spectrum for compound *Z*-3ap (400 MHz, $CDCl_3$, 25 ^{o}C).

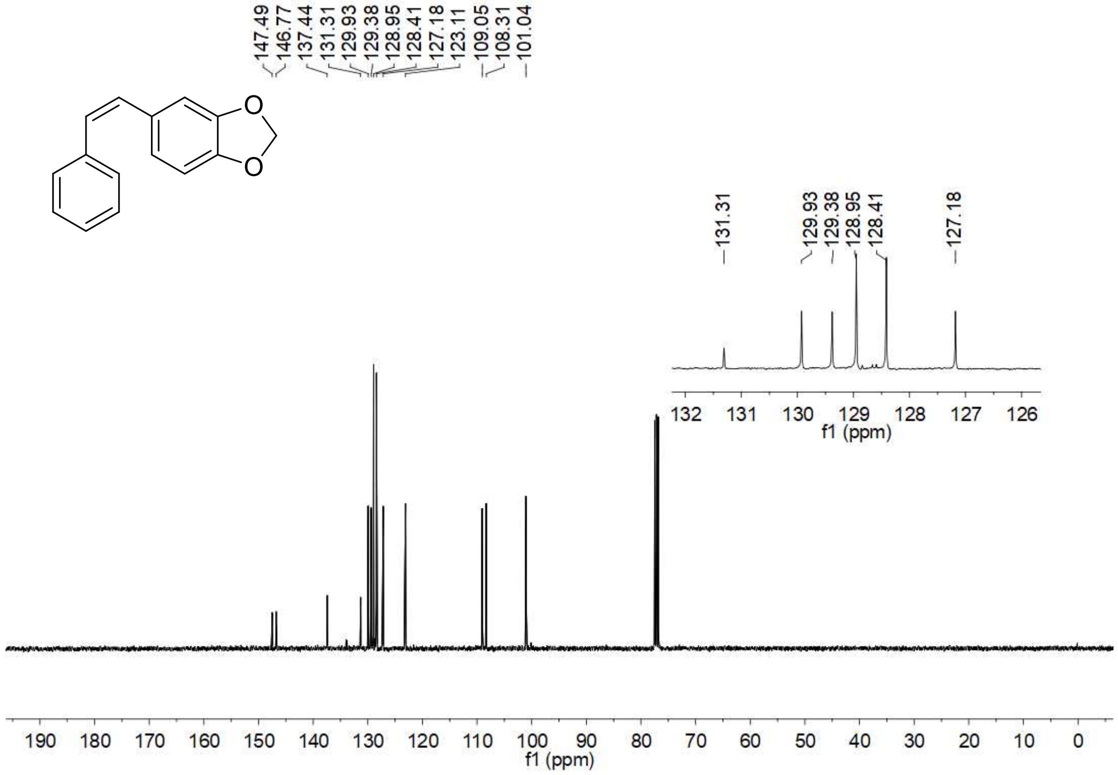

Supplementary Figure S118. ^{13}C NMR spectrum for compound *Z*-3ap (100 MHz, $CDCl_3$, 25 ^{o}C).

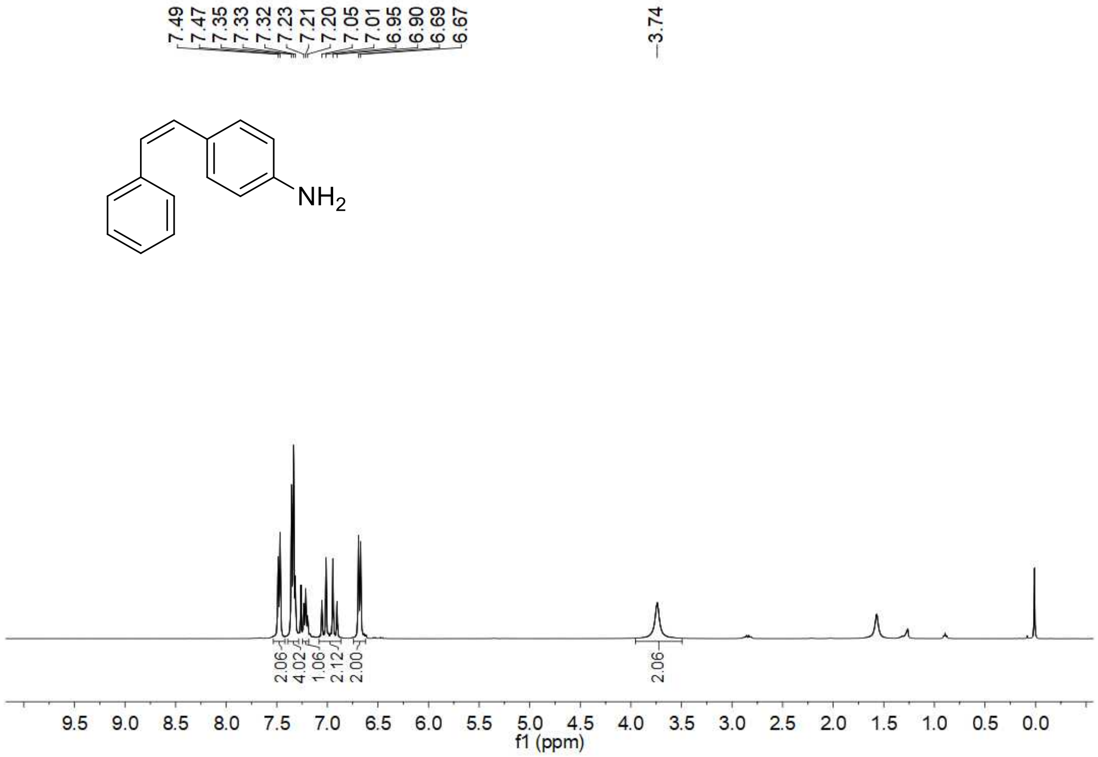

Supplementary Figure S119. ^{1}**H NMR spectrum for compound** ***Z*****-3i (400 MHz, CDCl$_3$, 25 ^{o}C).**

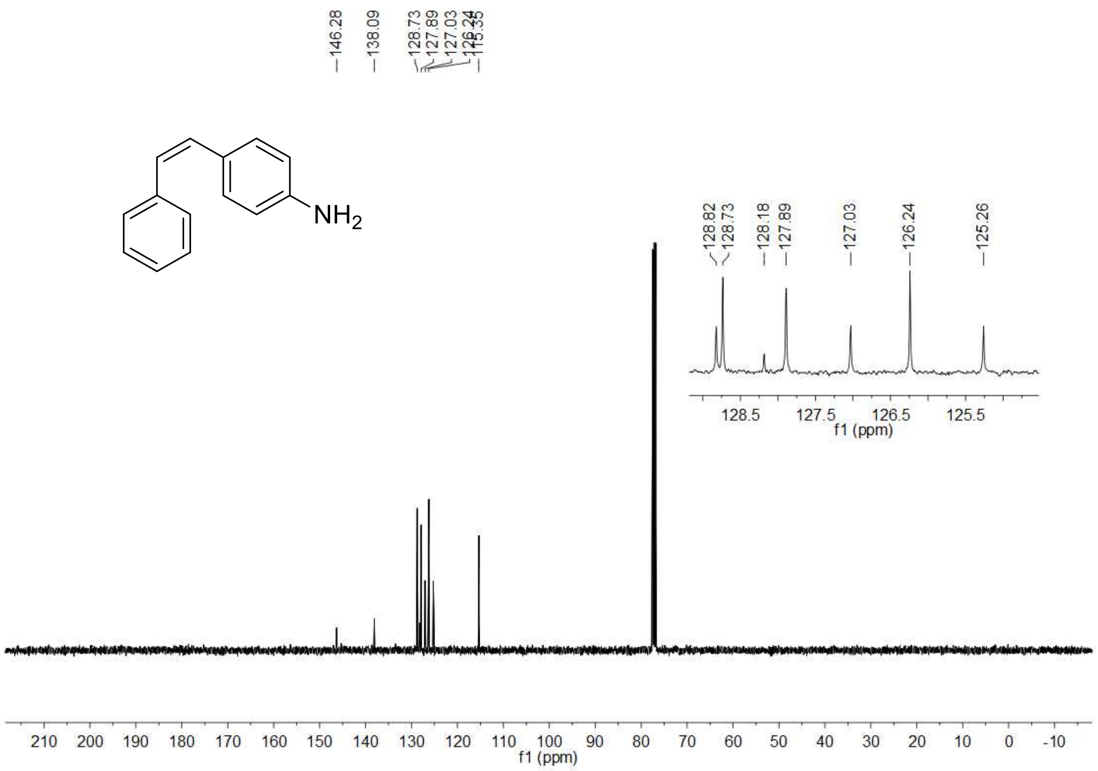

Supplementary Figure S120. ^{13}**C NMR spectrum for compound** ***Z*****-3i (100 MHz, CDCl$_3$, 25 ^{o}C).**

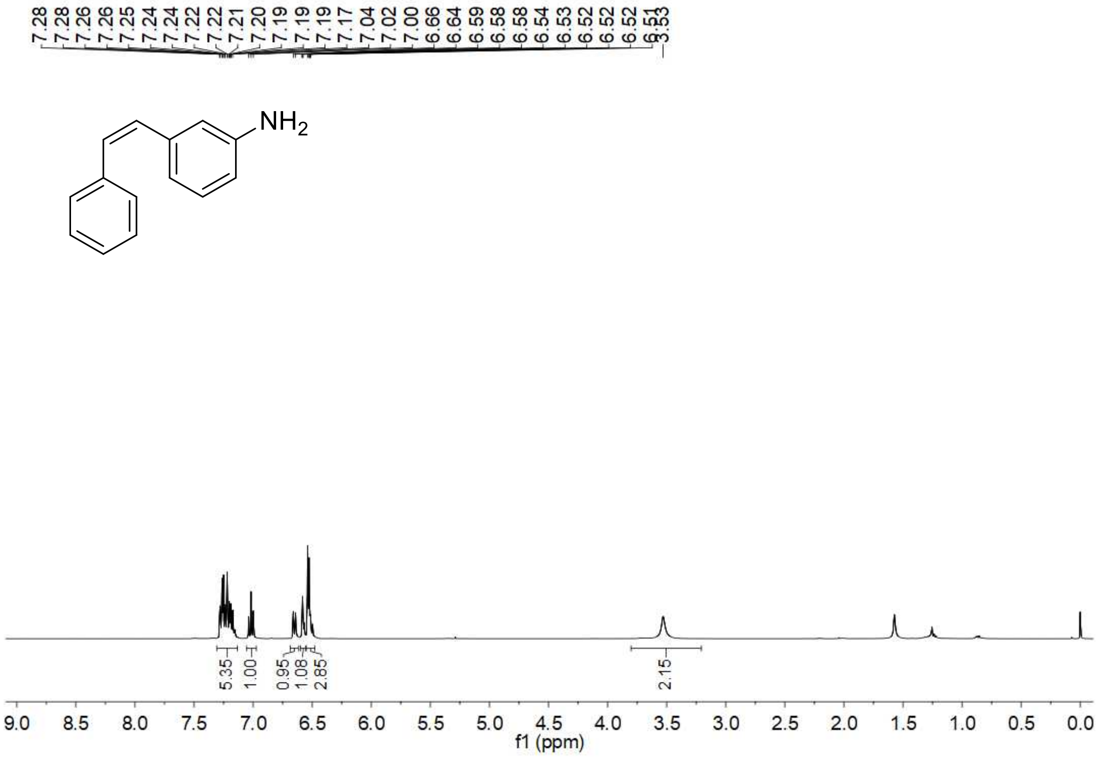

Supplementary Figure S121. ^{1}H NMR spectrum for compound *Z*-3aq (400 MHz, $CDCl_3$, 25 ^{o}C).

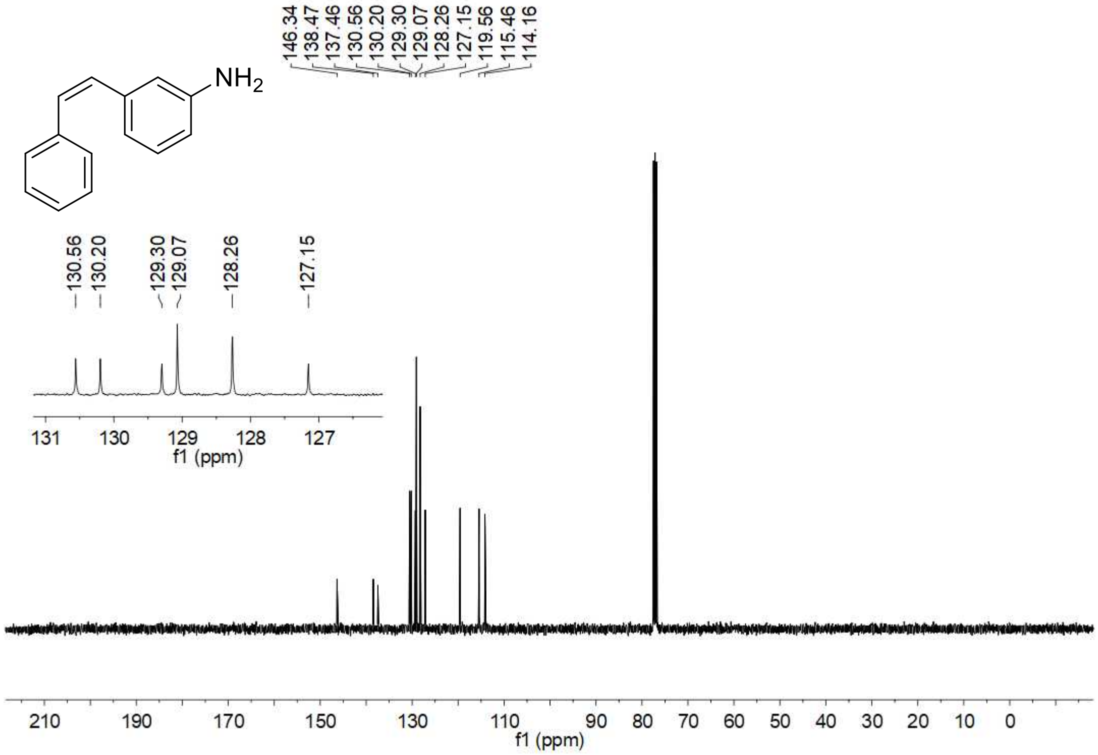

Supplementary Figure S122. ^{13}C NMR spectrum for compound *Z*-3aq (100 MHz, $CDCl_3$, 25 ^{o}C).

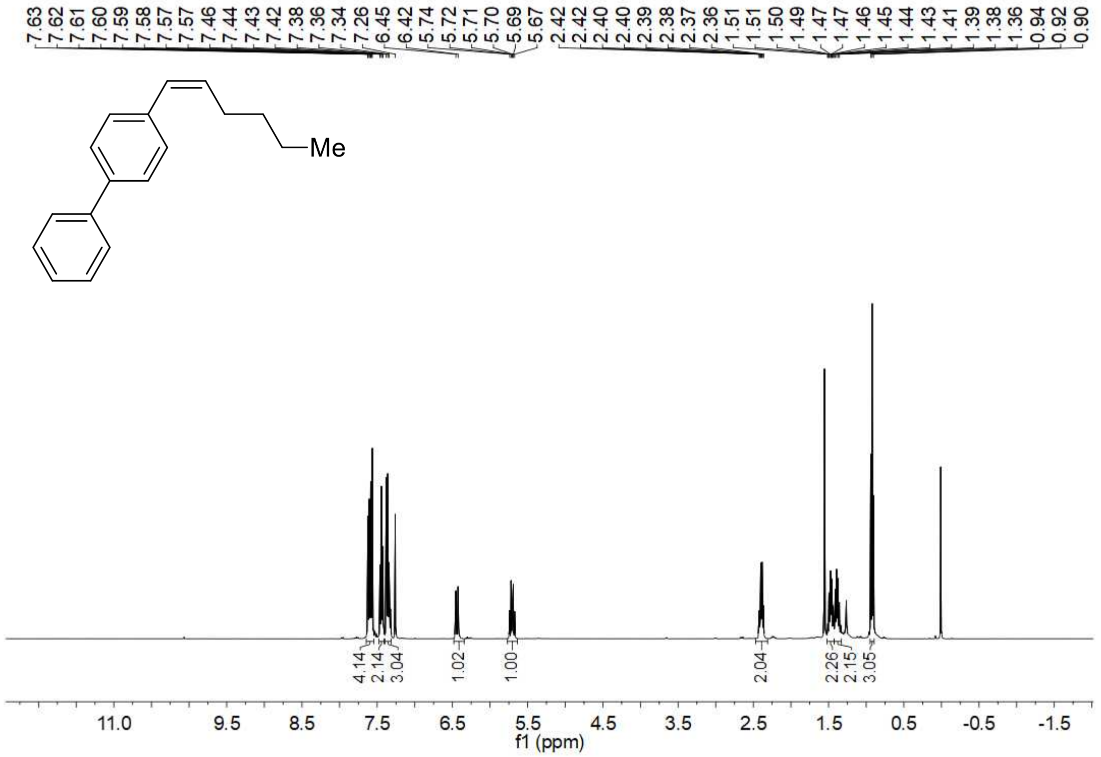

Supplementary Figure S123. ^{1}**H NMR spectrum for compound *Z*-3x (400 MHz, CDCl$_3$, 25 °C).**

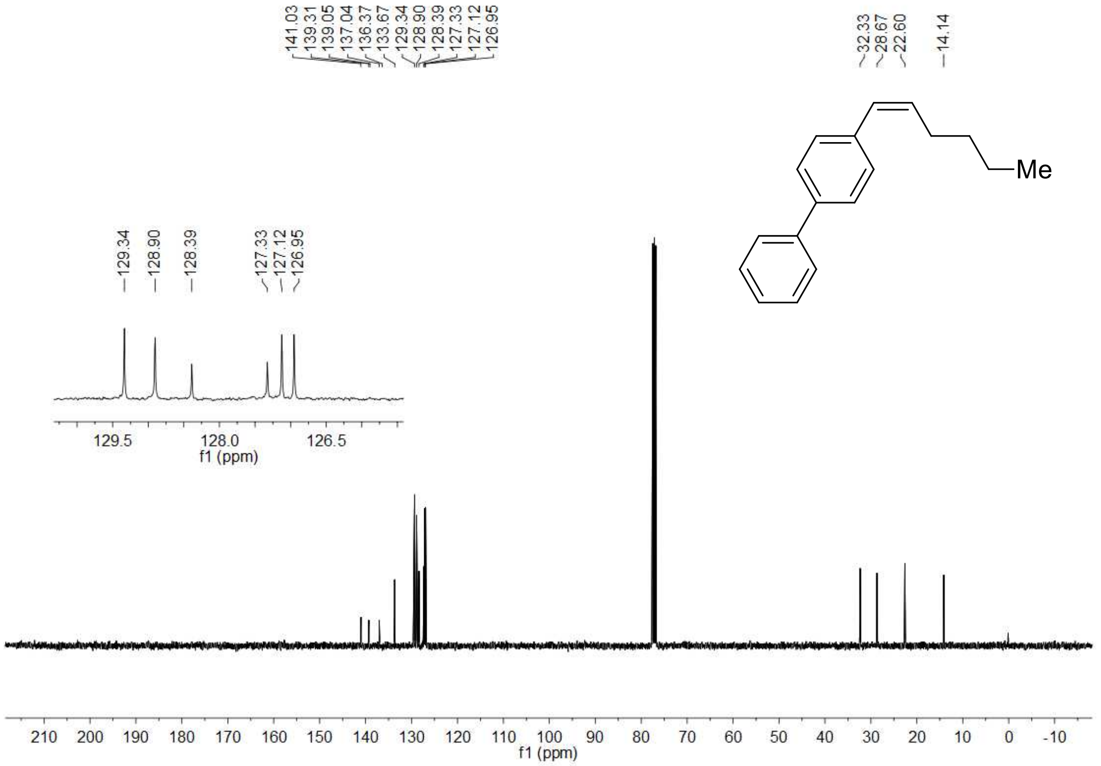

Supplementary Figure S124. ^{13}**C NMR spectrum for compound *Z*-3x (100 MHz, CDCl$_3$, 25 °C).**

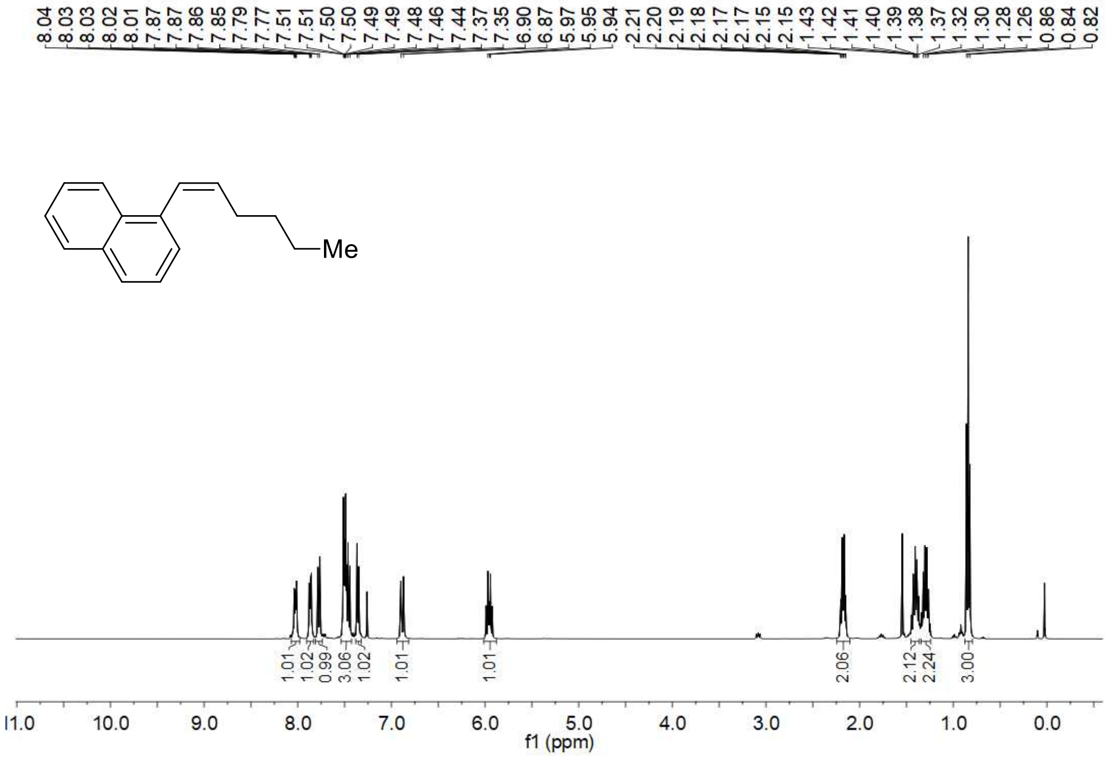

Supplementary Figure S125. ^{1}**H NMR spectrum for compound** ***Z*****-3ar (400 MHz, CDCl$_3$, 25 ^{o}C).**

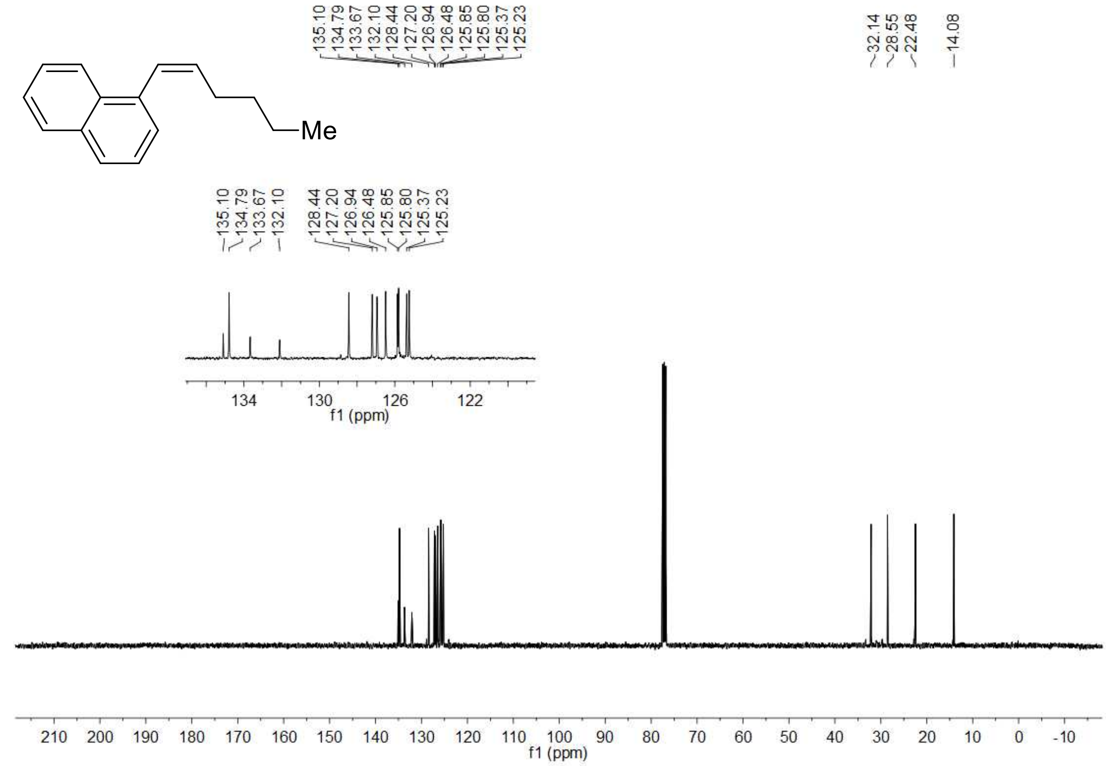

Supplementary Figure S126. ^{13}**C NMR spectrum for compound** ***Z*****-3ar (100 MHz, CDCl$_3$, 25 ^{o}C).**

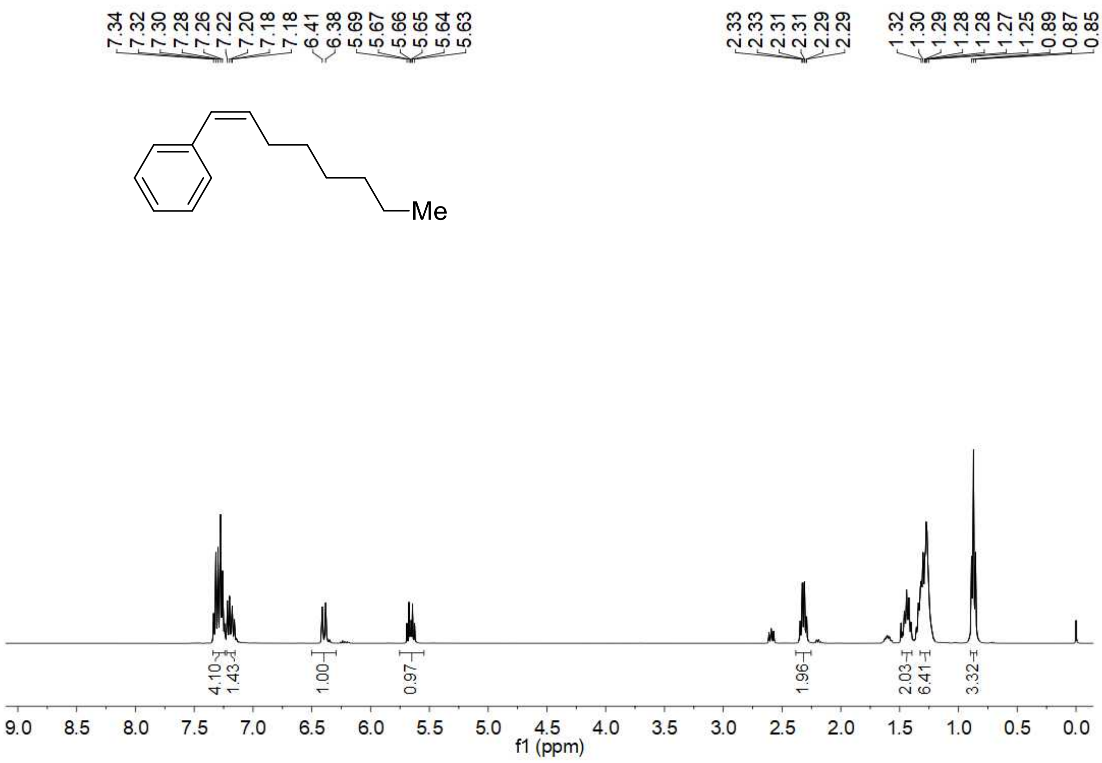

Supplementary Figure S127. ^{1}H NMR spectrum for compound *Z*-3as (400 MHz, $CDCl_3$, 25 °C).

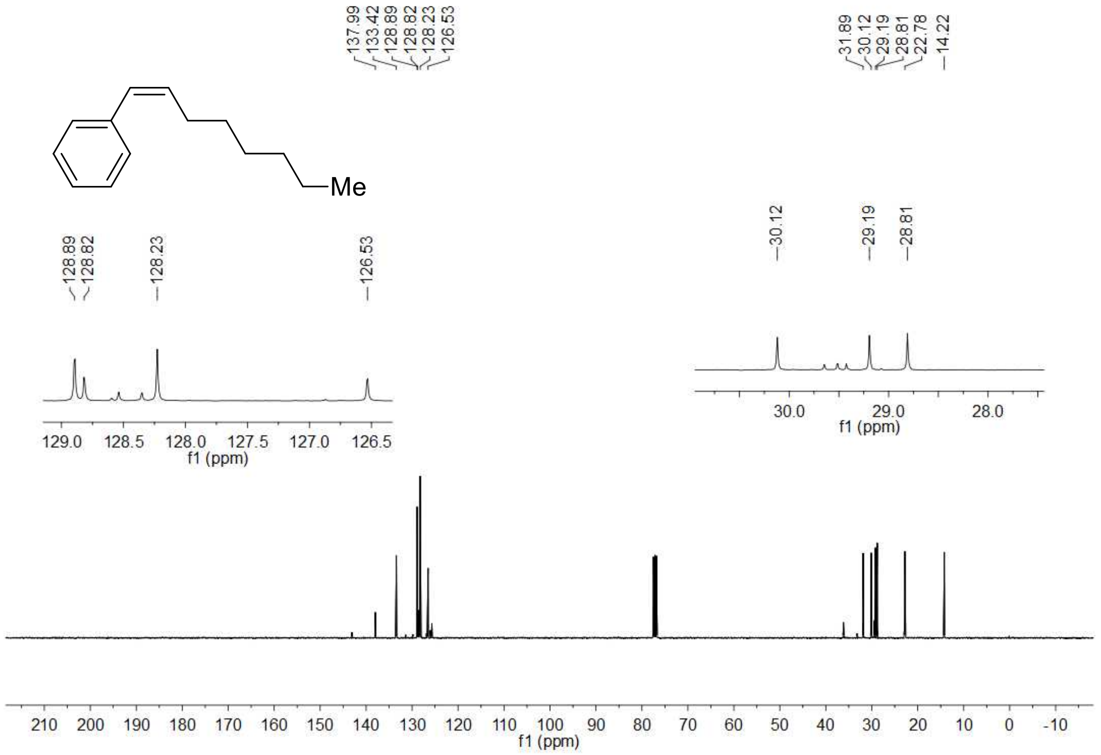

Supplementary Figure S128. ^{13}C NMR spectrum for compound *Z*-3as (100 MHz, $CDCl_3$, 25 °C).

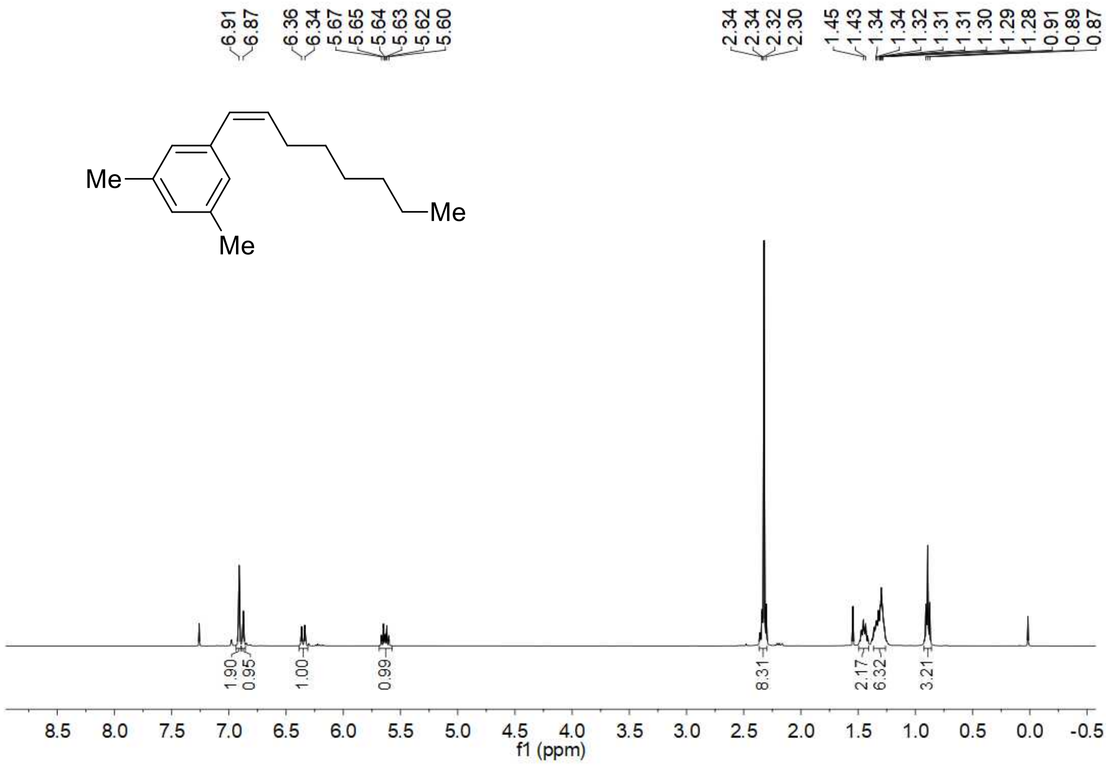

Supplementary Figure S129. ^{1}**H NMR spectrum for compound *Z*-3u (400 MHz, $CDCl_3$, 25 ^{o}C).**

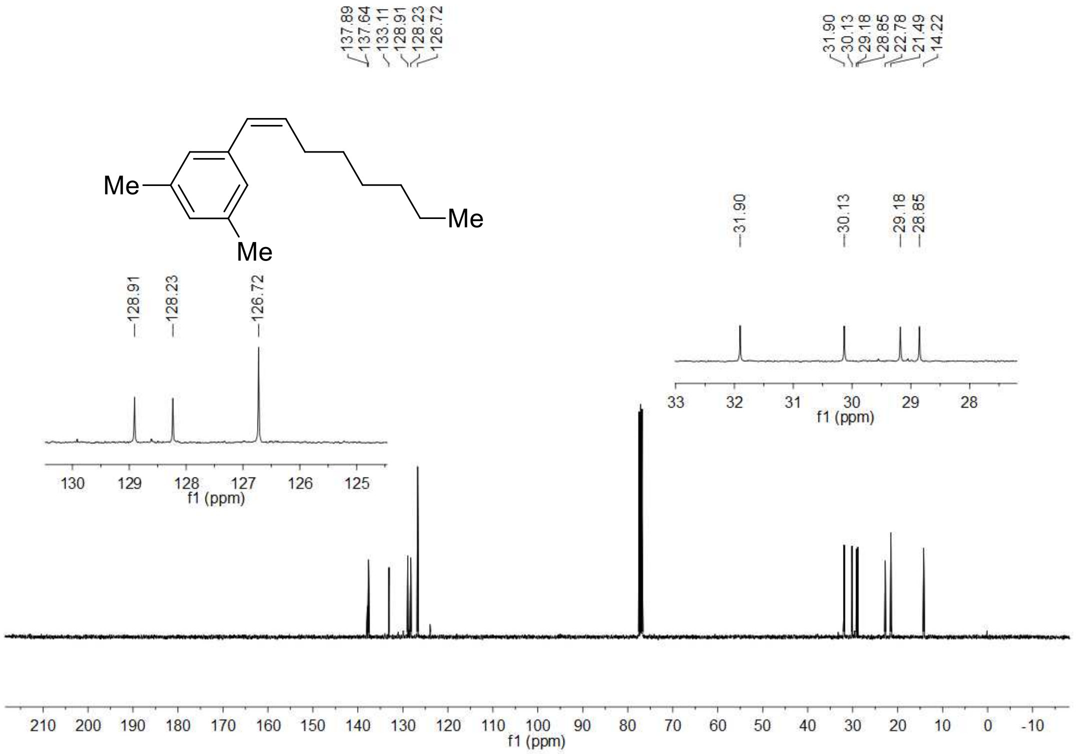

Supplementary Figure S130. ^{13}**C NMR spectrum for compound *Z*-3u (100 MHz, $CDCl_3$, 25 ^{o}C).**

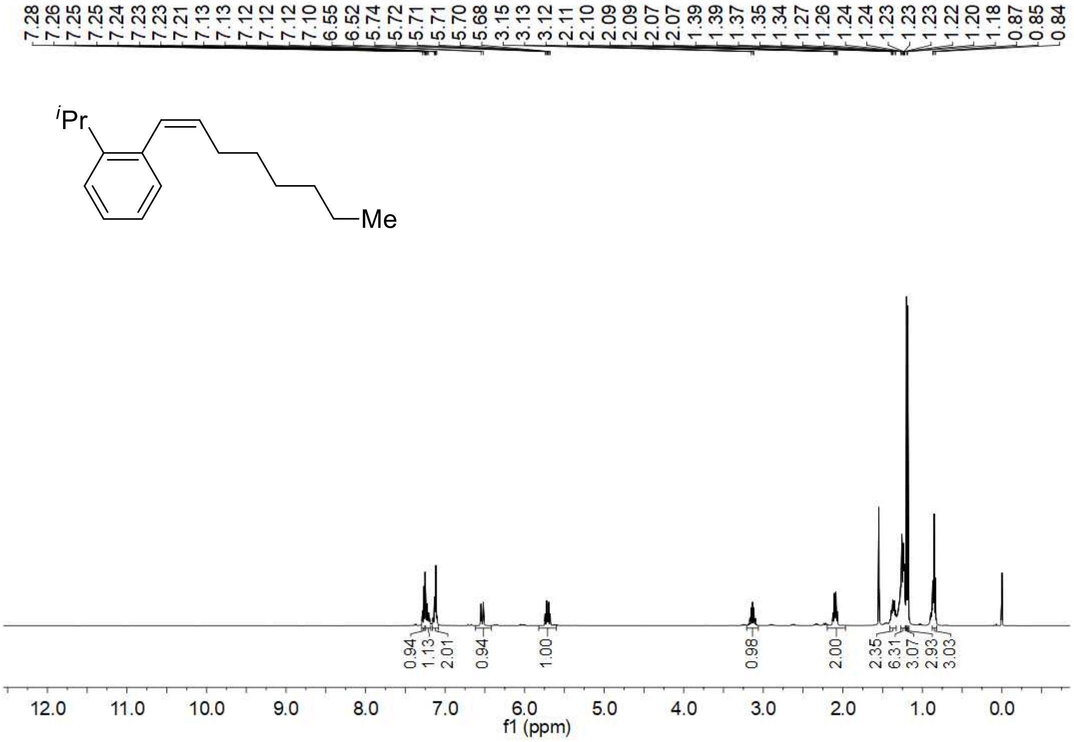

Supplementary Figure S131. ^{1}H NMR spectrum for compound *Z*-3at (400 MHz, $CDCl_3$, 25 ^{o}C).

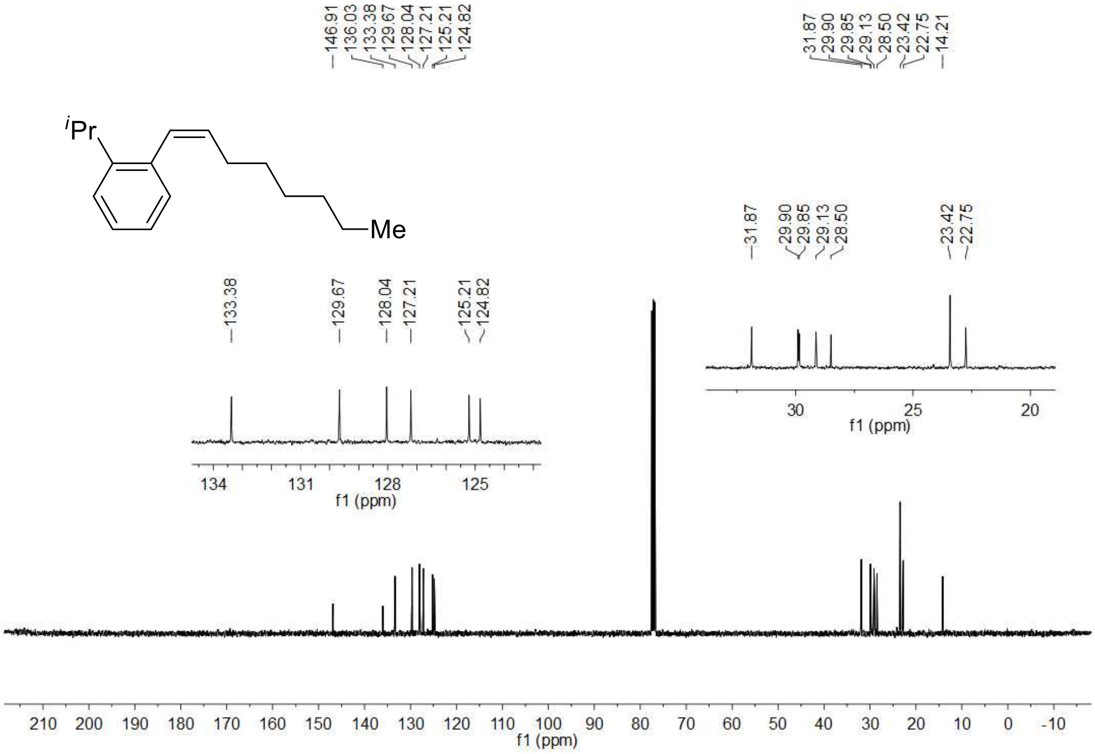

Supplementary Figure S132. ^{13}C NMR spectrum for compound *Z*-3at (100 MHz, $CDCl_3$, 25 ^{o}C).

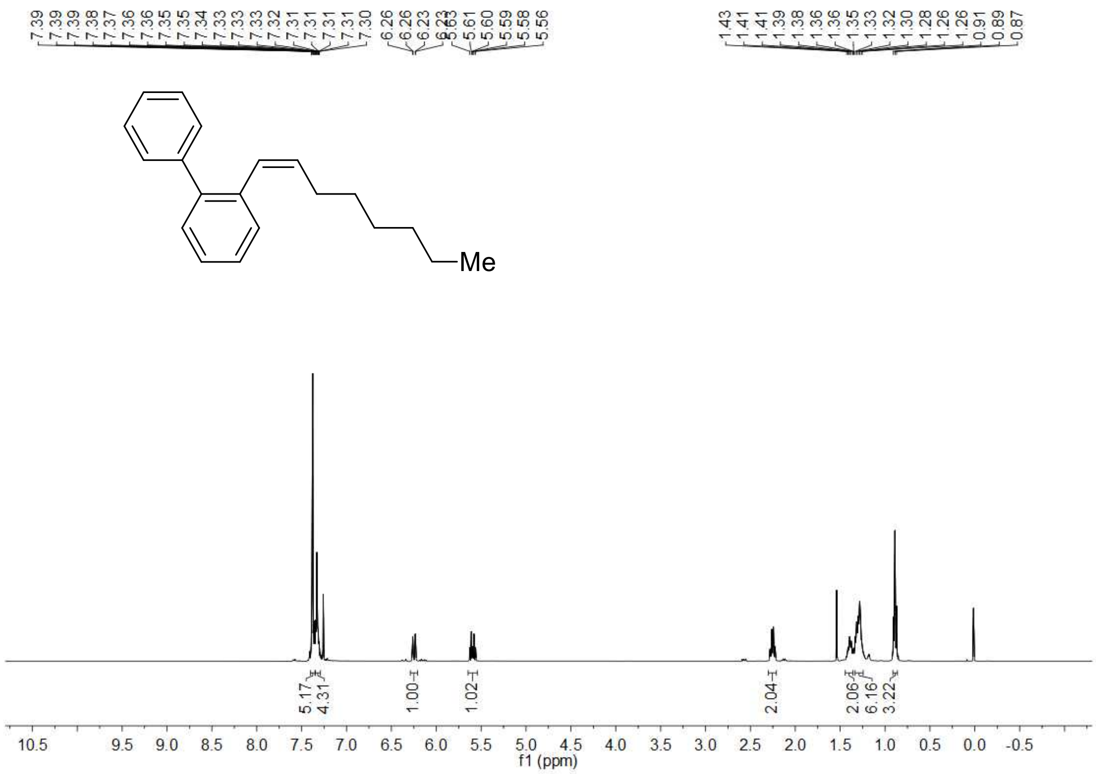

Supplementary Figure S133. ^{1}H NMR spectrum for compound *Z*-3au (400 MHz, $CDCl_3$, 25 °C).

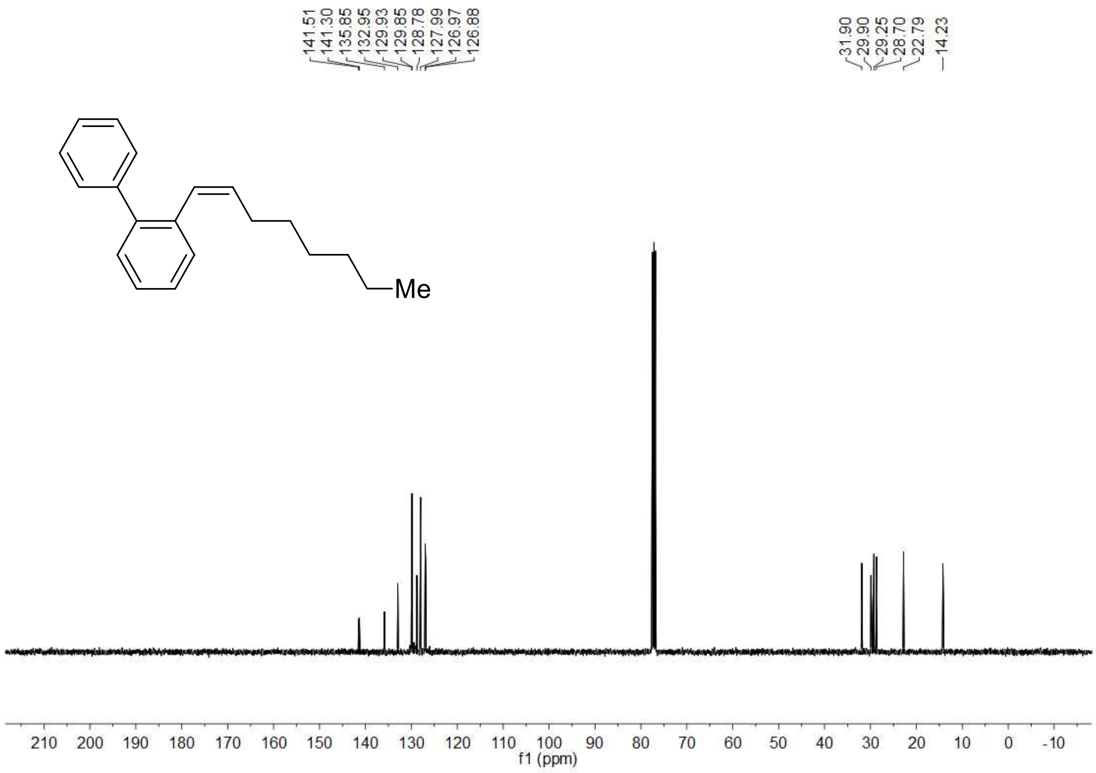

Supplementary Figure S134. ^{13}C NMR spectrum for compound *Z*-3au (100 MHz, $CDCl_3$, 25 °C).

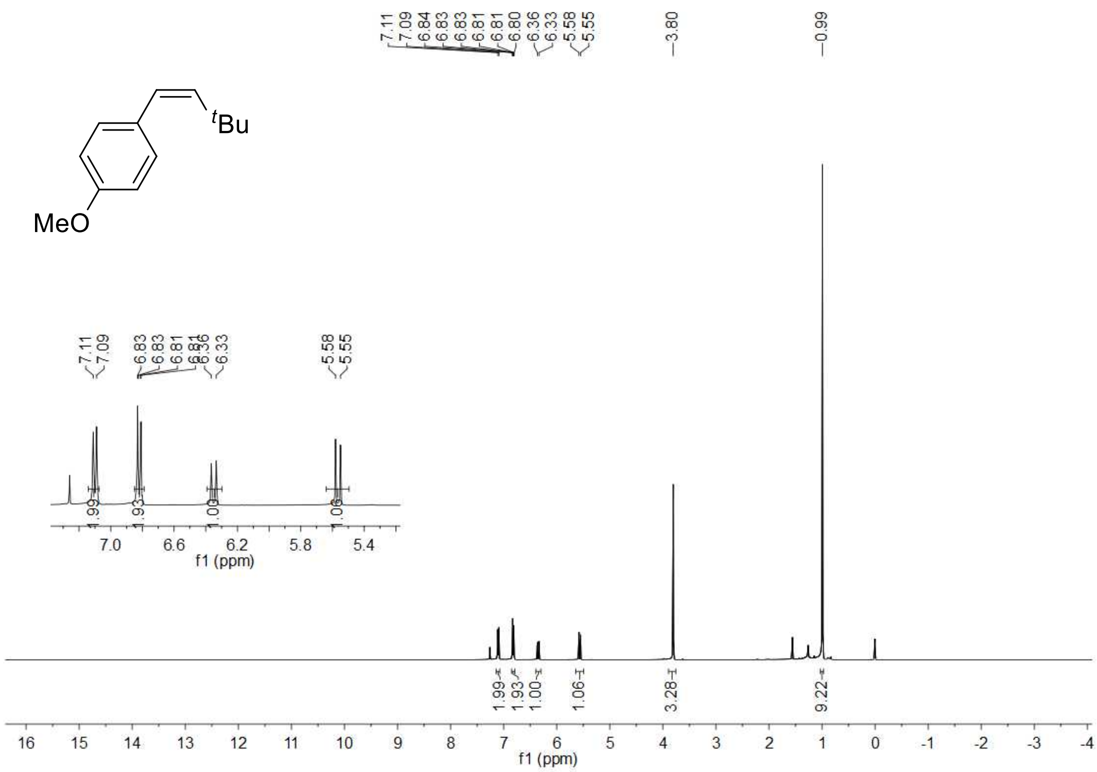

Supplementary Figure S135. ^{1}H NMR spectrum for compound *Z*-3av (400 MHz, $CDCl_3$, 25 °C).

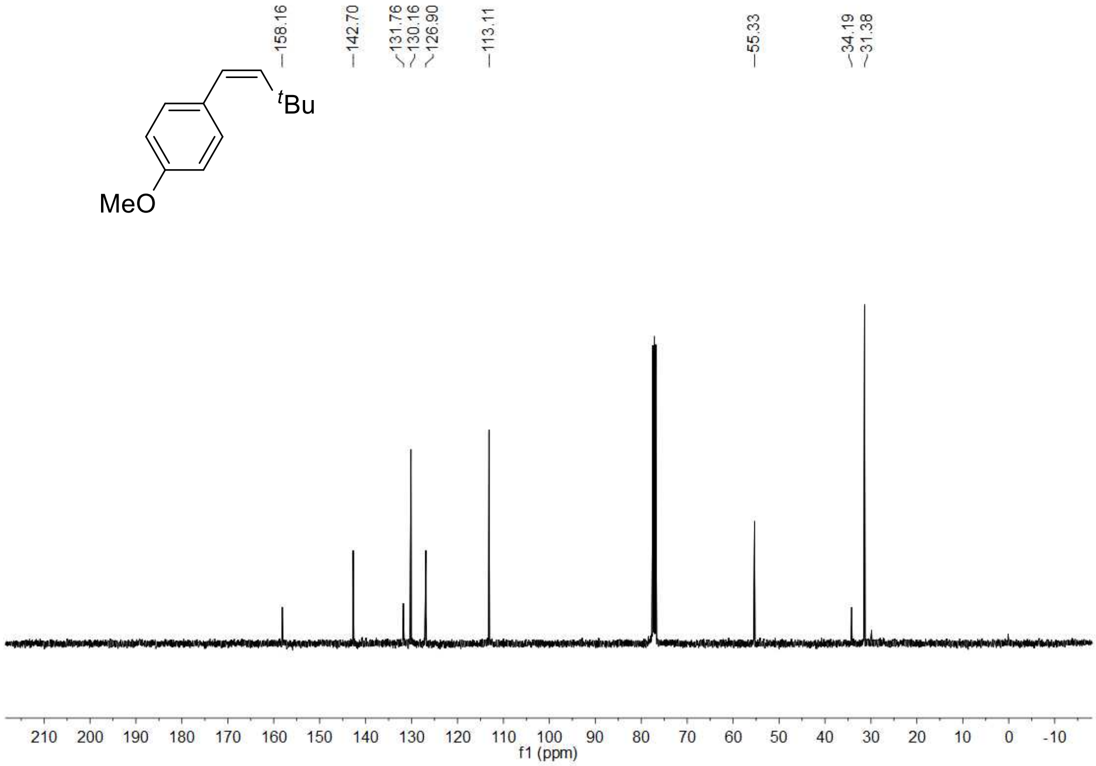

Supplementary Figure S136. ^{13}C NMR spectrum for compound *Z*-3av (100 MHz, $CDCl_3$, 25 °C).

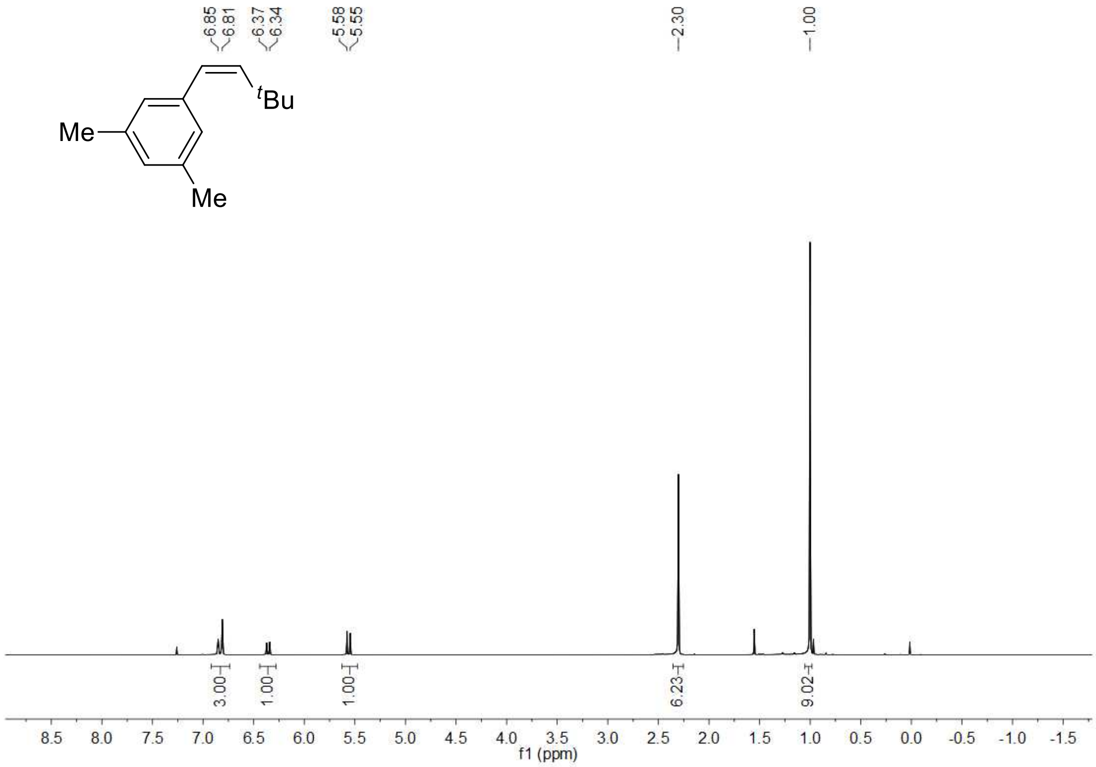

Supplementary Figure S137. ^{1}H NMR spectrum for compound *Z*-3aw (400 MHz, CDCl$_3$, 25 ^{o}C).

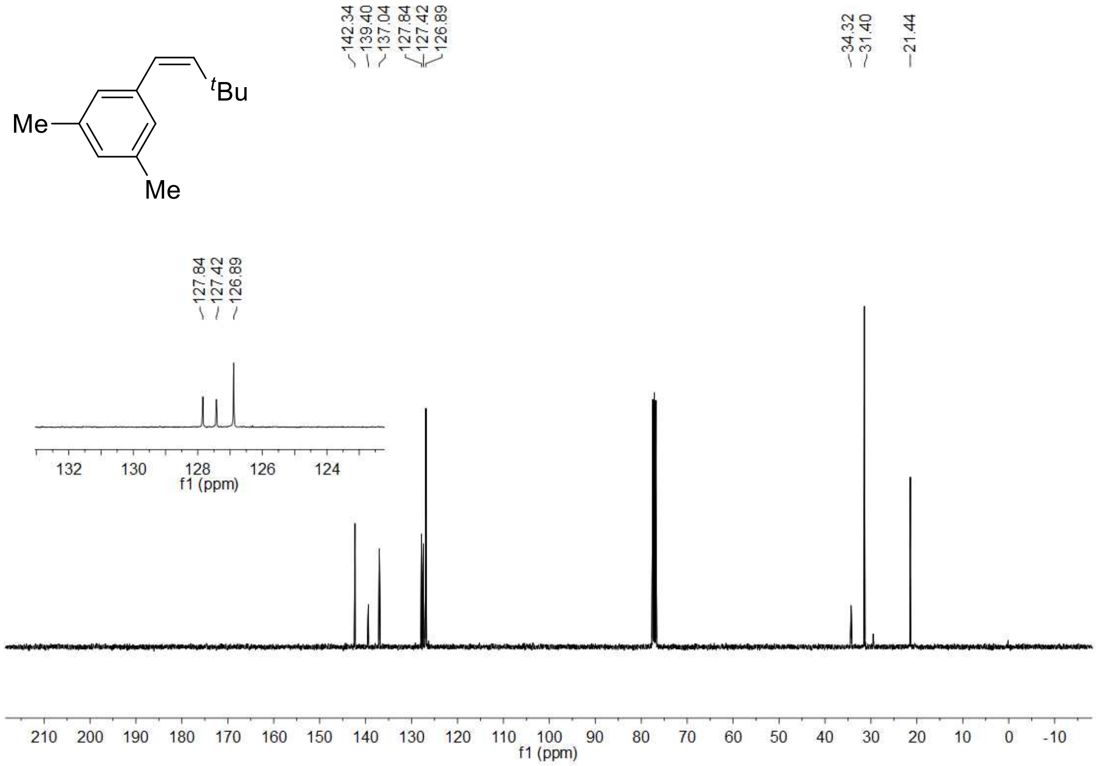

Supplementary Figure S138. ^{13}C NMR spectrum for compound *Z*-3aw (100 MHz, CDCl$_3$, 25 ^{o}C).

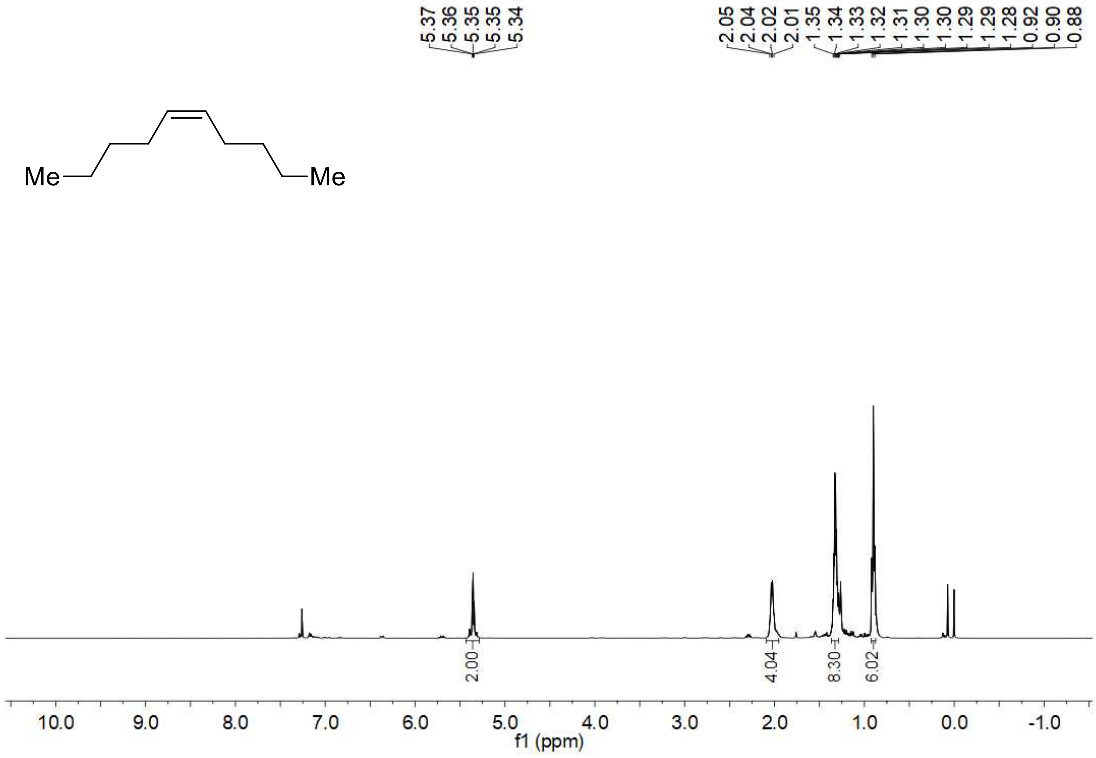

Supplementary Figure S139. ^{1}H NMR spectrum for compound *Z*-3ax (400 MHz, $CDCl_3$, 25 °C).

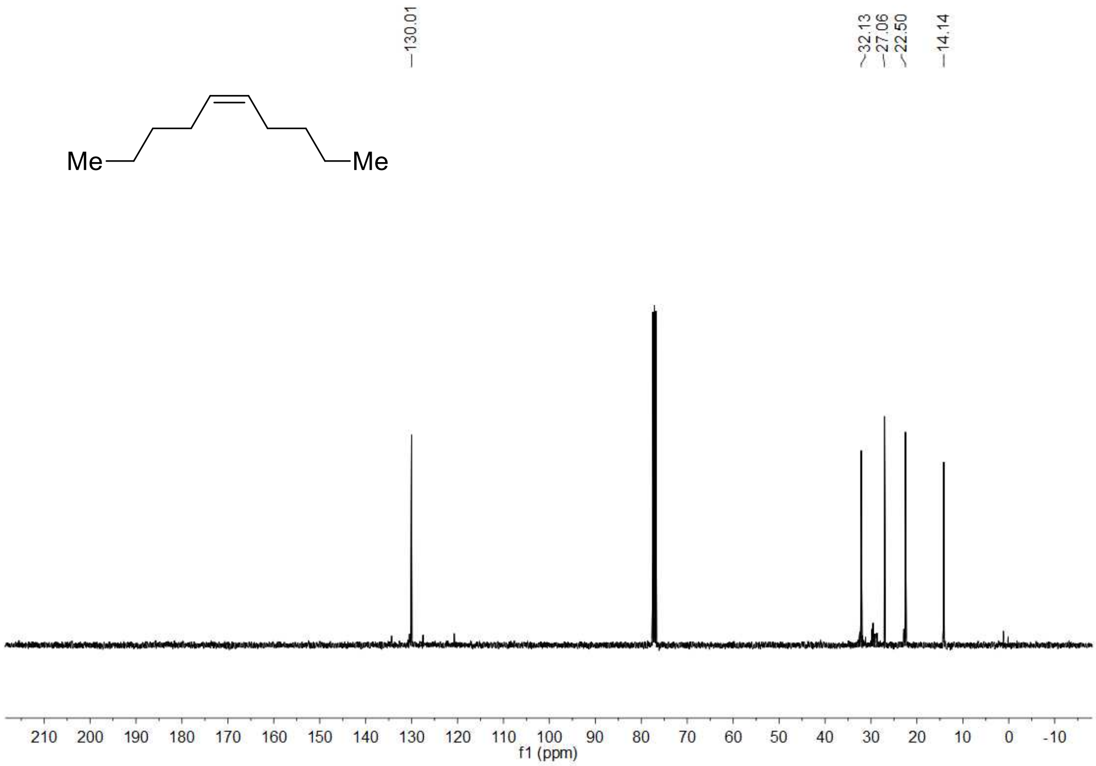

Supplementary Figure S140. ^{13}C NMR spectrum for compound *Z*-3ax (100 MHz, $CDCl_3$, 25 °C).

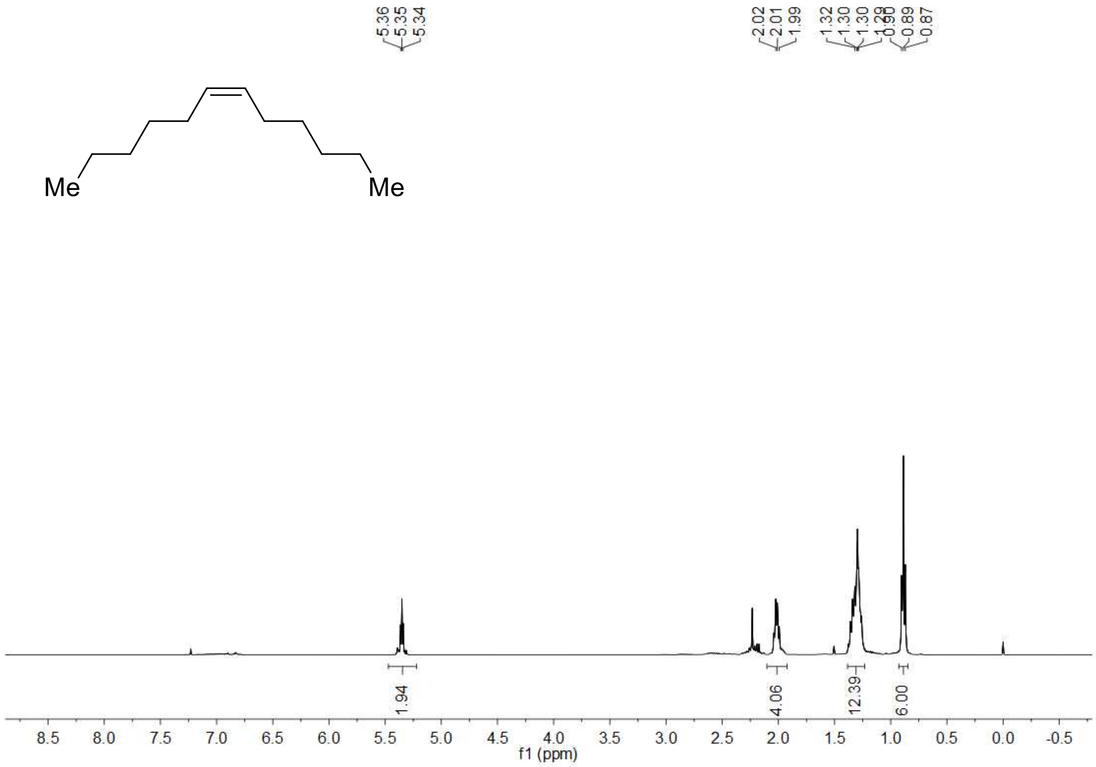

Supplementary Figure S141. ^{1}H NMR spectrum for compound *Z*-3ay (400 MHz, $CDCl_3$, 25 °C).

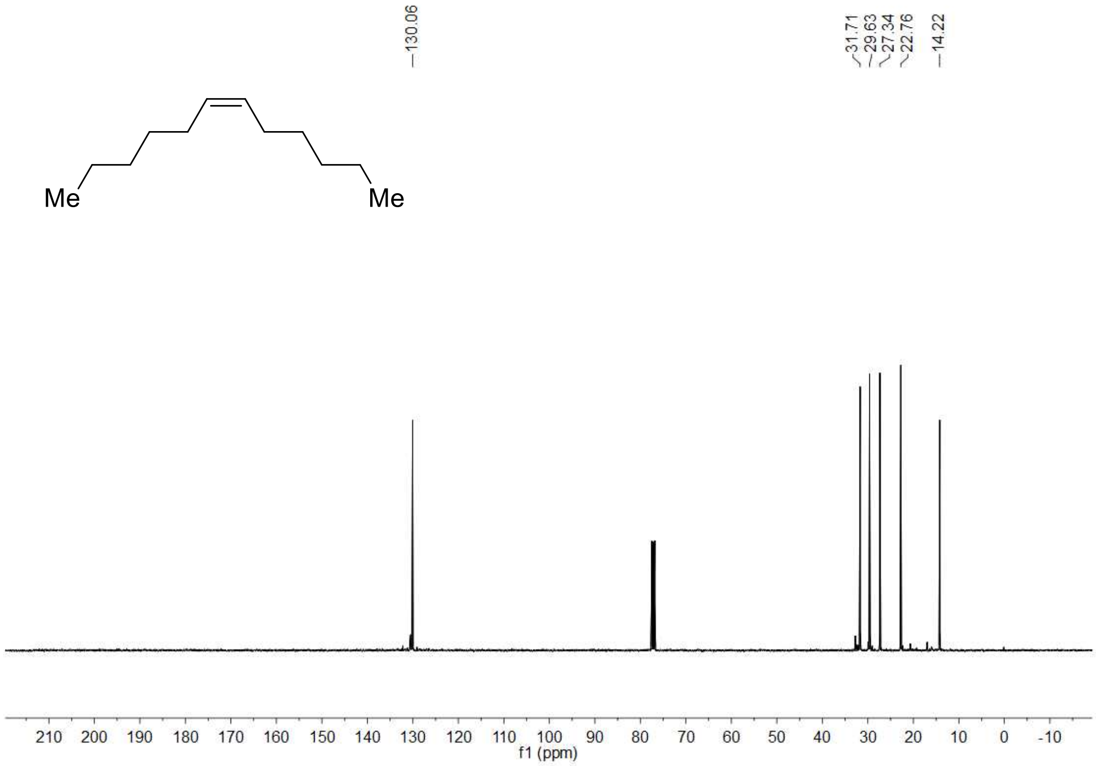

Supplementary Figure S142. ^{13}C NMR spectrum for compound *Z*-3ay (100 MHz, $CDCl_3$, 25 °C).

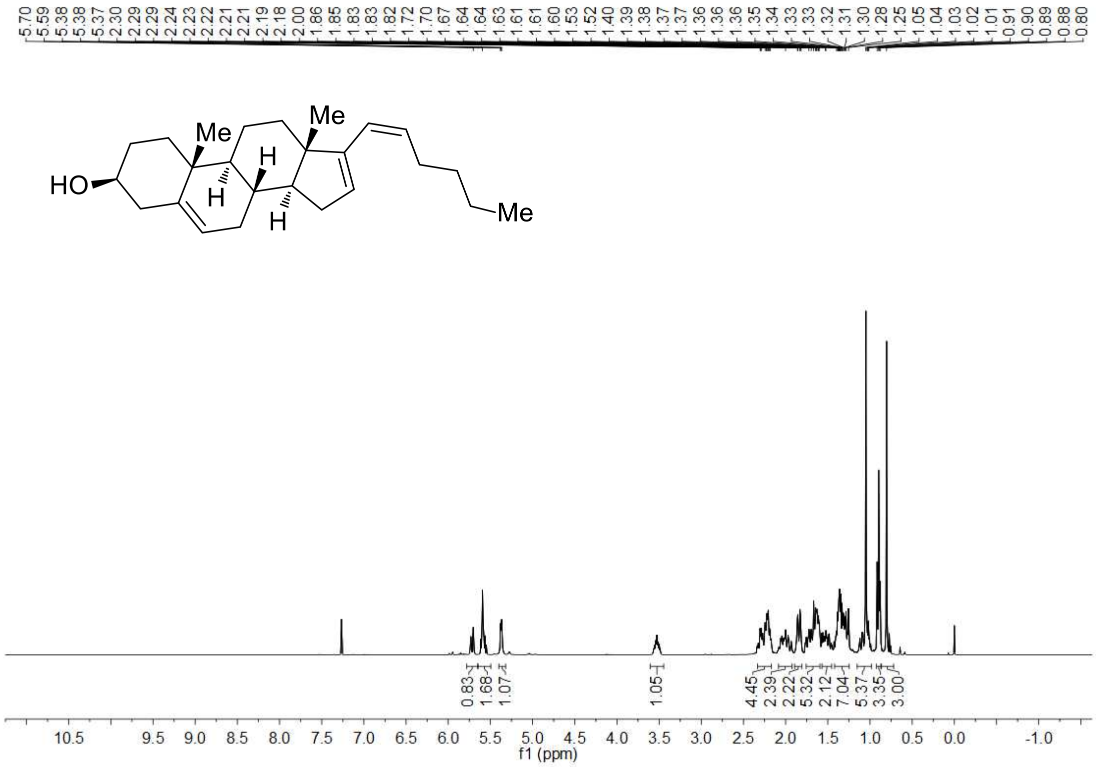

Supplementary Figure S143. ^{1}H NMR spectrum for compound *Z*-3az (400 MHz, $CDCl_3$, 25 ºC).

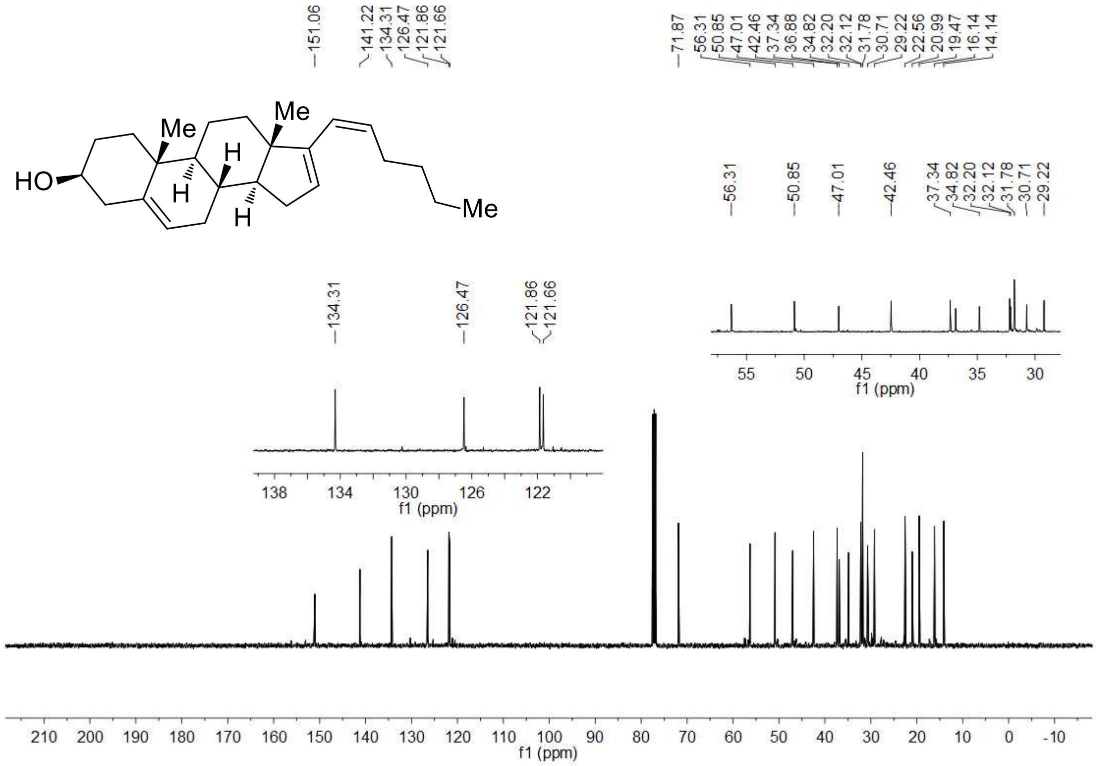

Supplementary Figure S144. ^{13}C NMR spectrum for compound *Z*-3az (100 MHz, $CDCl_3$, 25 ºC).

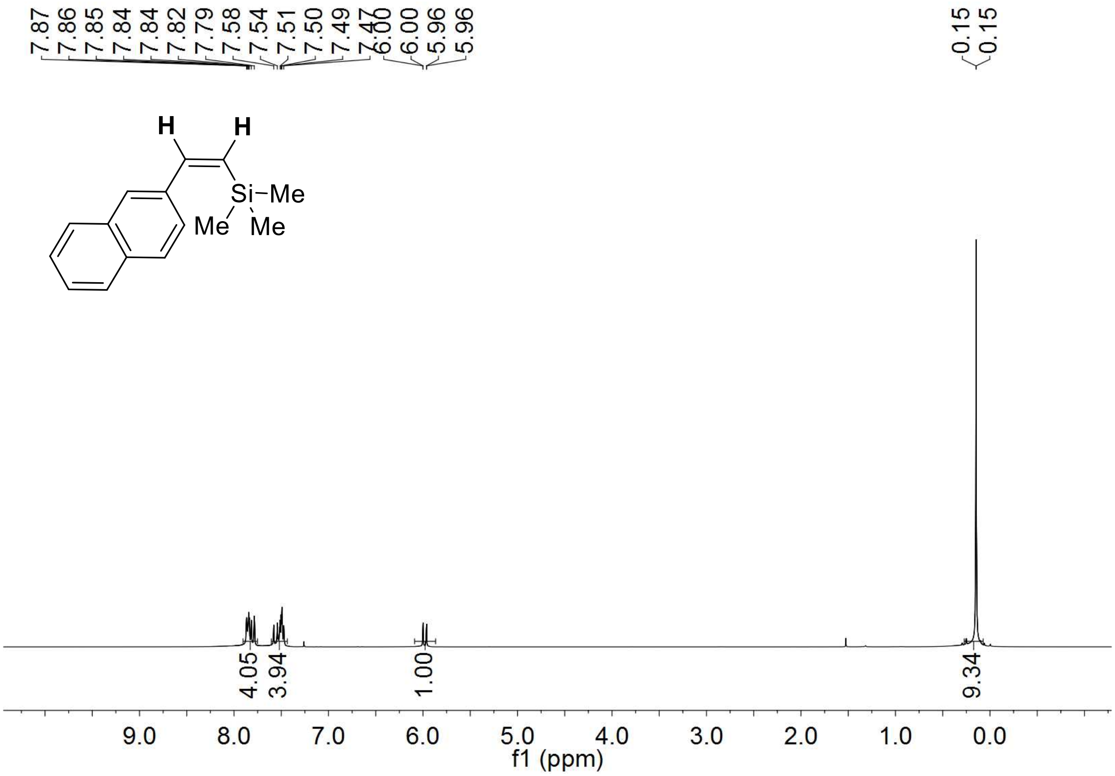

Supplementary Figure S145. ^{1}H NMR spectrum for compound *Z*-3ba (400 MHz, $CDCl_3$, 25 ºC).

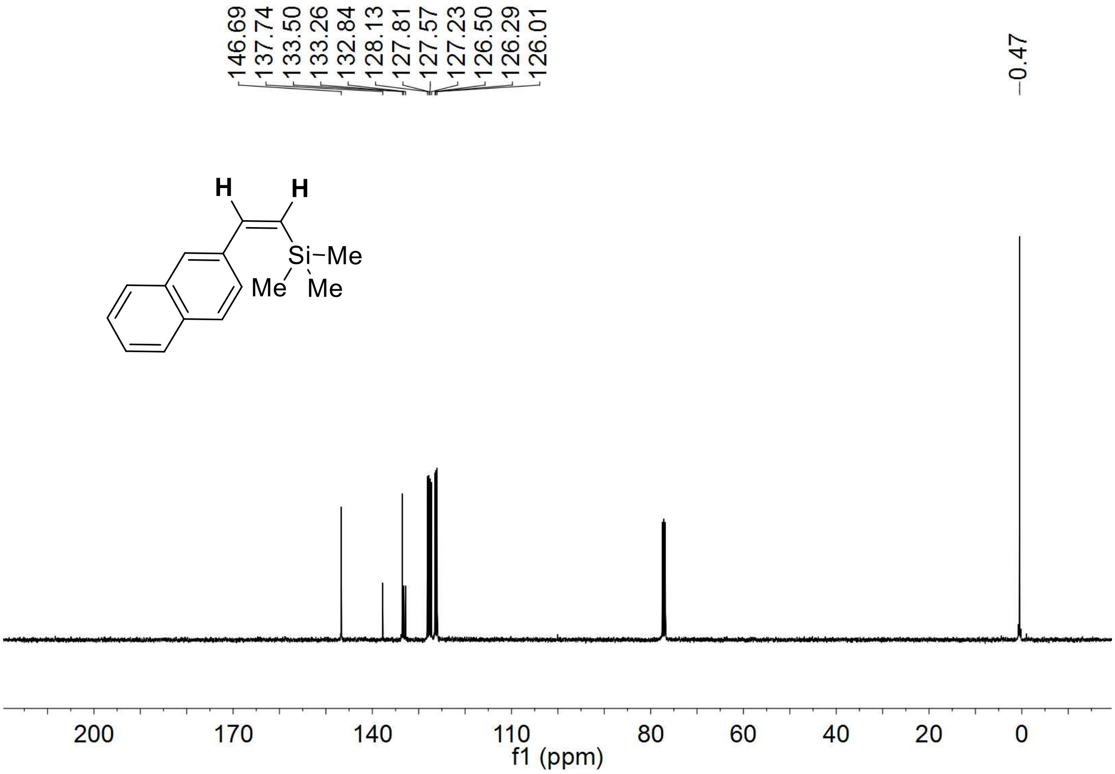

Supplementary Figure S146. ^{13}C NMR spectrum for compound *Z*-3ba (100 MHz, $CDCl_3$, 25 ºC).

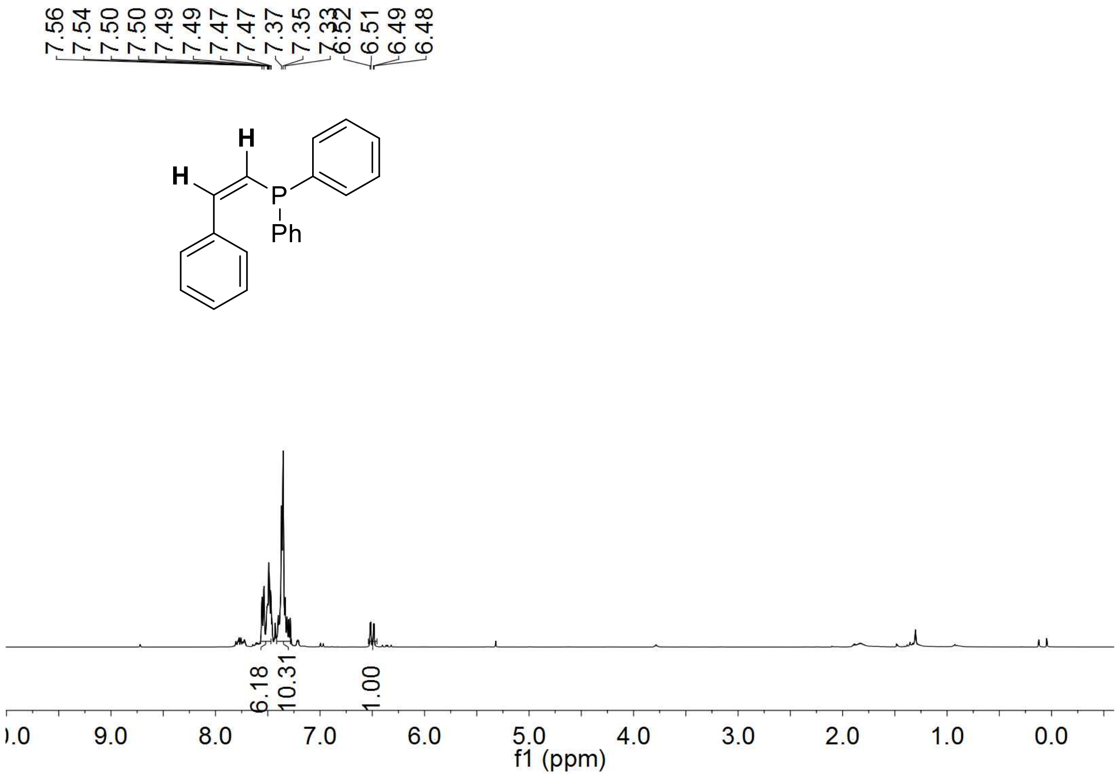

Supplementary Figure S147. ^{1}H NMR spectrum for compound *Z*-3bb (400 MHz, $CDCl_3$, 25 °C).

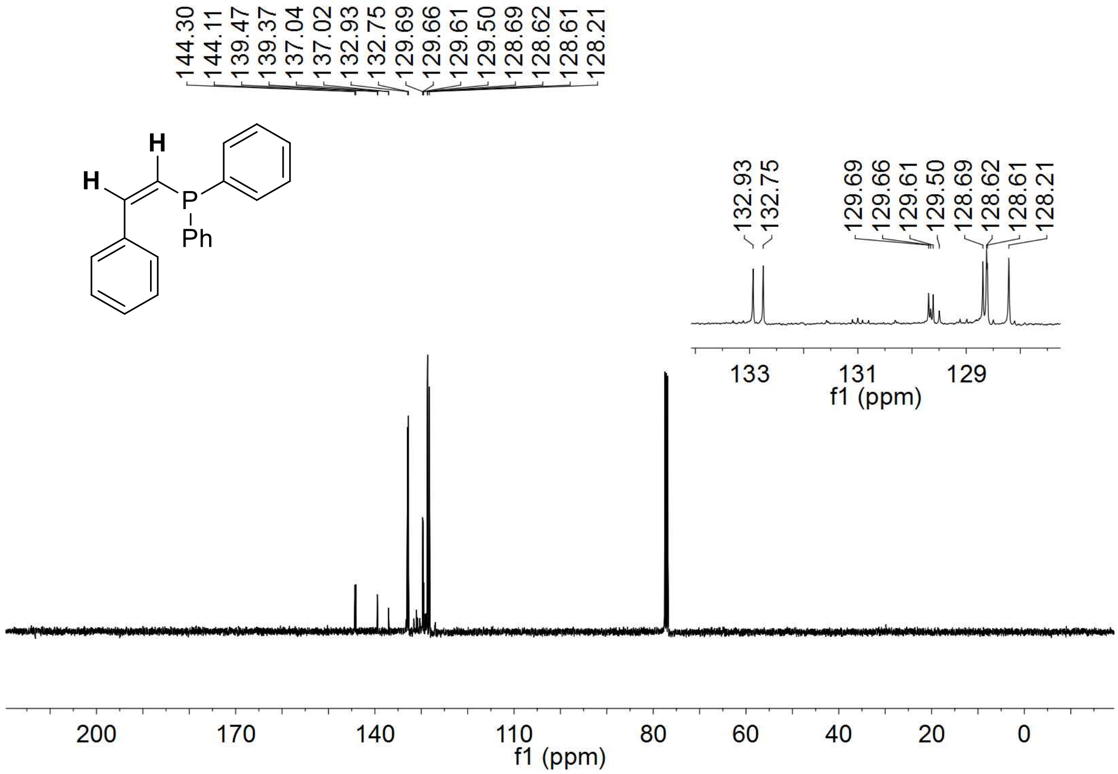

Supplementary Figure S148. ^{13}C NMR spectrum for compound *Z*-3bb (100 MHz, $CDCl_3$, 25 °C).

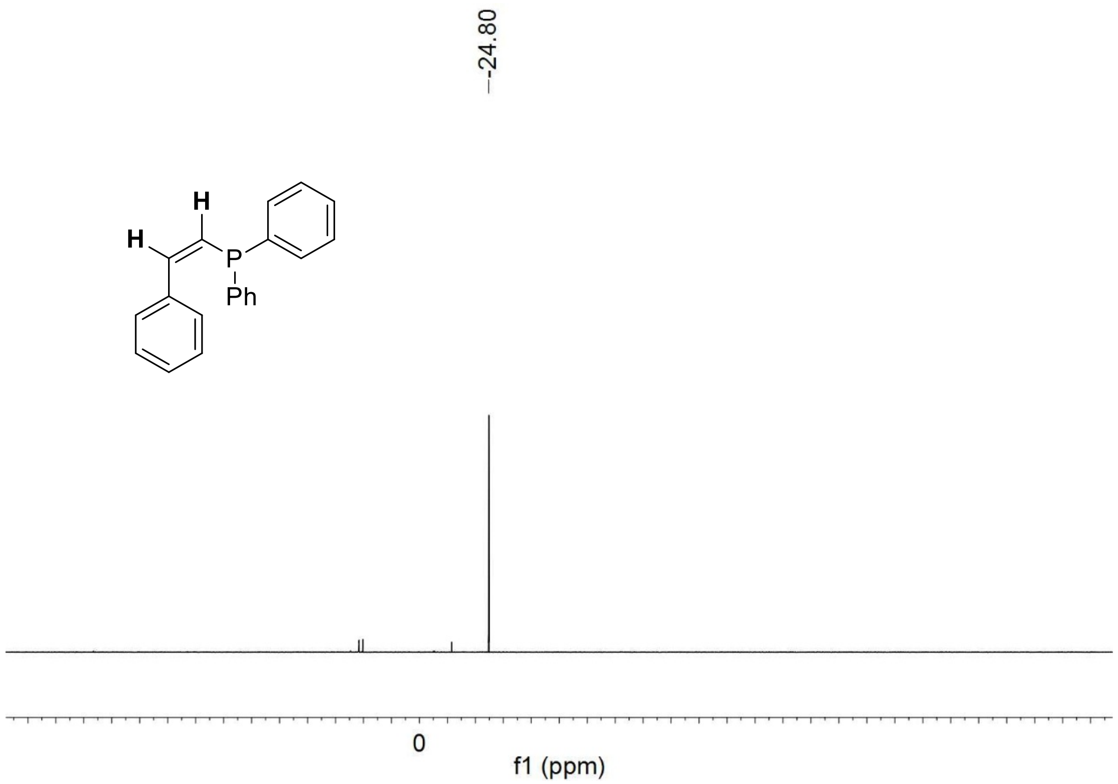

Supplementary Figure S149. ^{31}P NMR spectrum for compound *Z*-3bb (162 MHz, $CDCl_3$, 25 °C).

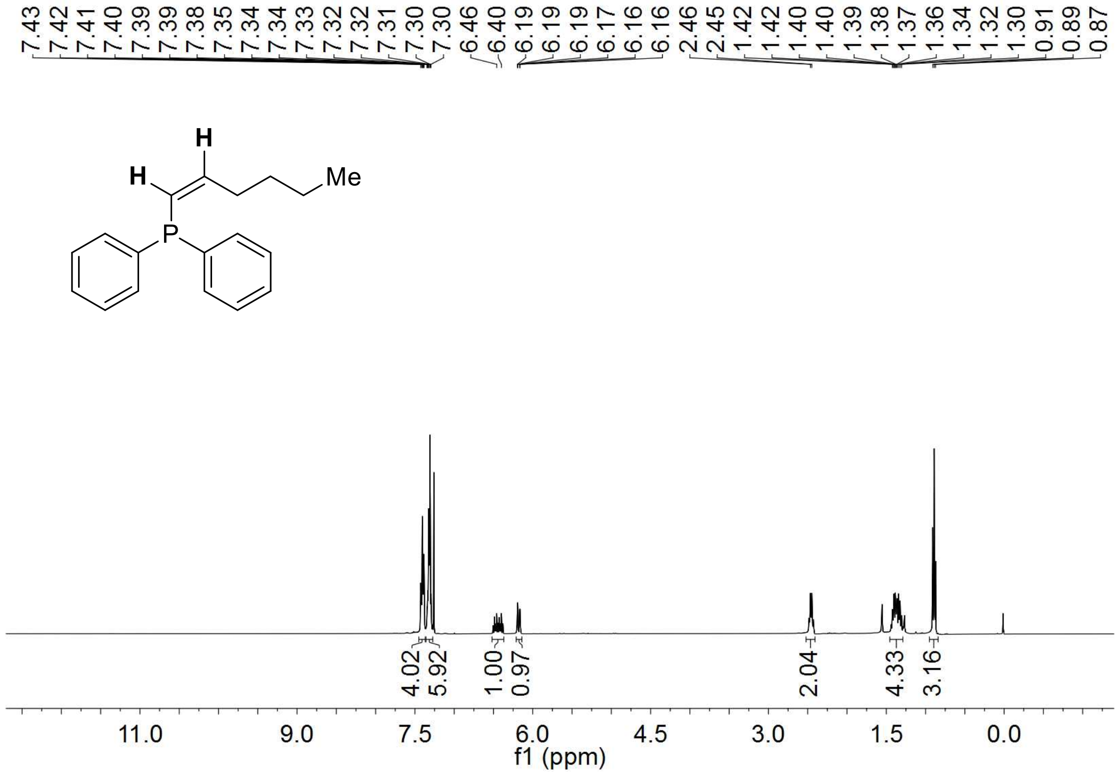

Supplementary Figure S150. ^{1}H NMR spectrum for compound *Z*-3bc (400 MHz, $CDCl_3$, 25 °C).

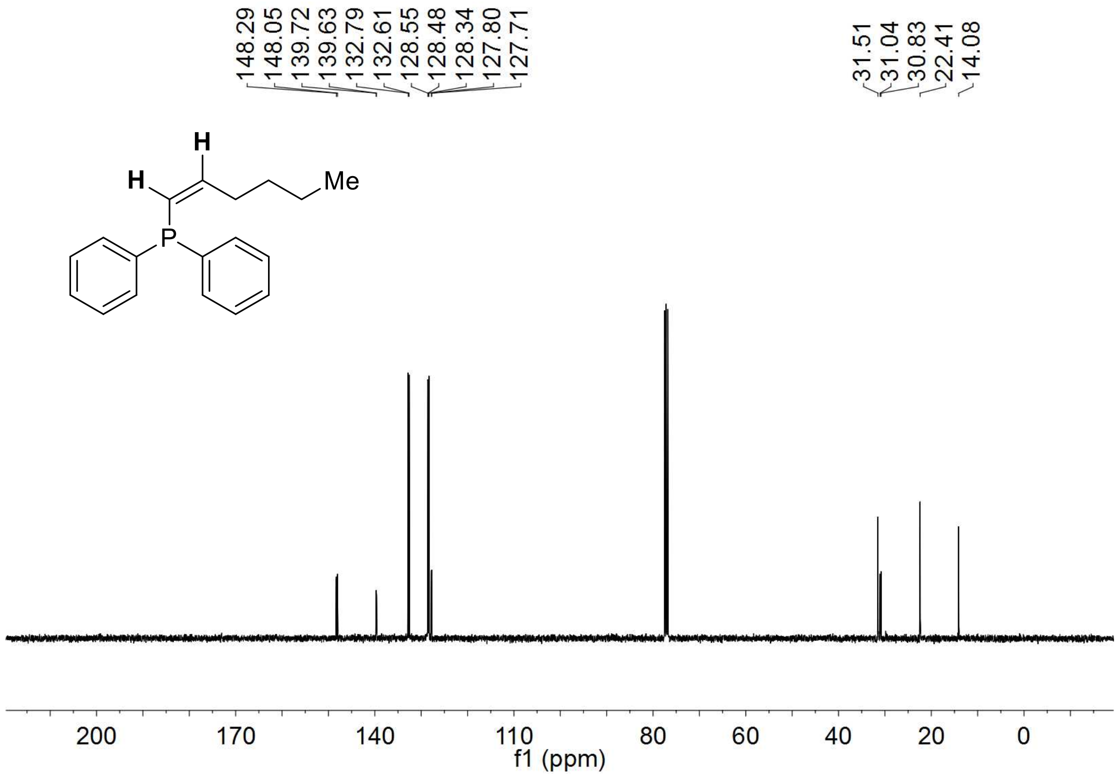

Supplementary Figure S151. ^{13}C NMR spectrum for compound *Z*-3bc (100 MHz, $CDCl_3$, 25 °C).

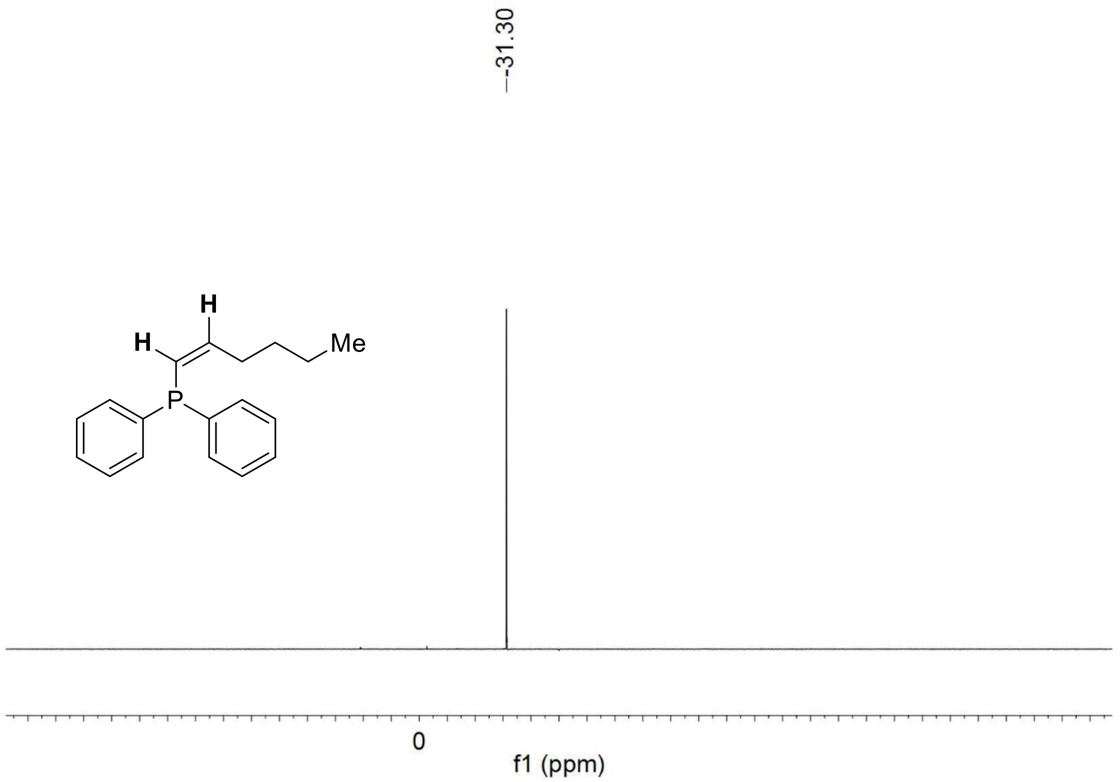

Supplementary Figure S152. ^{31}P NMR spectrum for compound *Z*-3bc (162 MHz, $CDCl_3$, 25 °C).

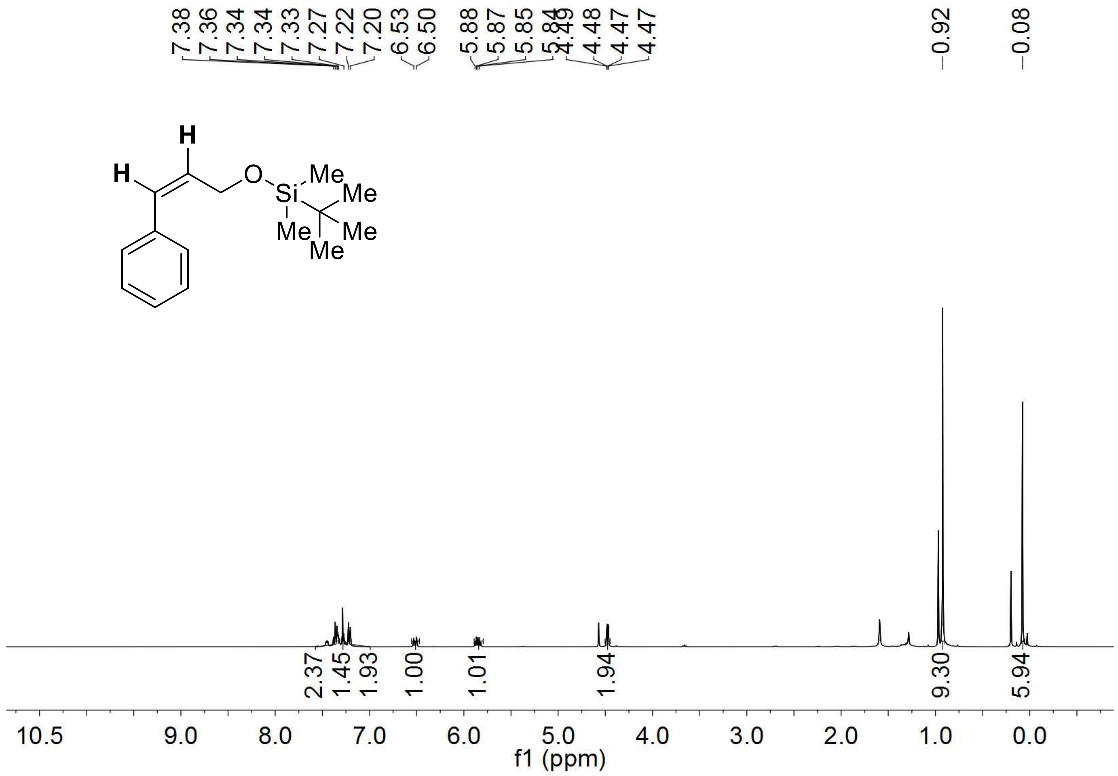

Supplementary Figure S153. ^{1}H NMR spectrum for compound *Z*-3bd (400 MHz, $CDCl_3$, 25 °C).

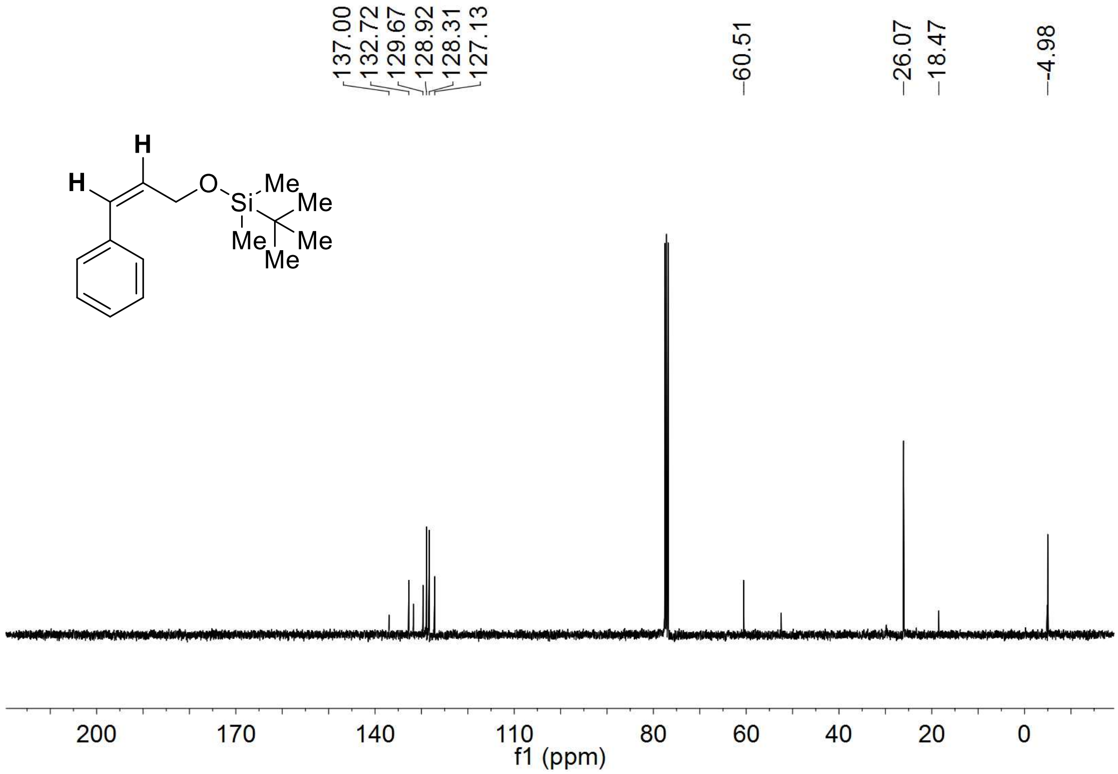

Supplementary Figure S154. ^{13}C NMR spectrum for compound *Z*-3bd (100 MHz, $CDCl_3$, 25 °C).

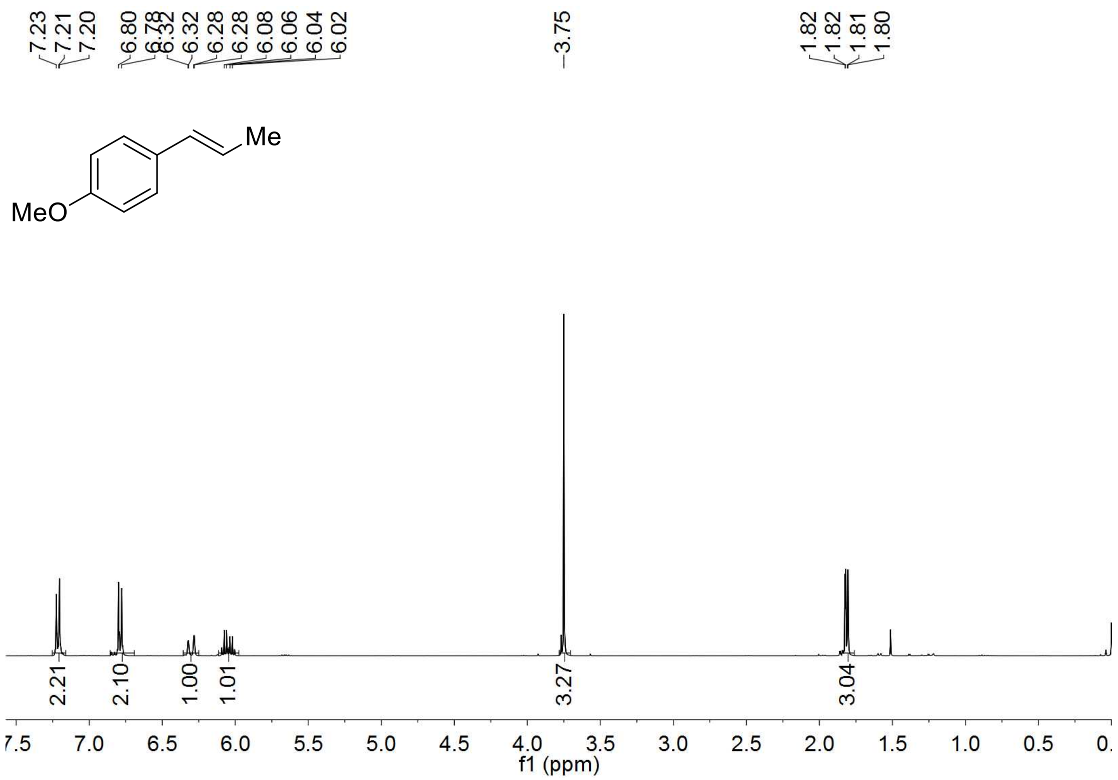

Supplementary Figure S155. **^{1}H NMR spectrum for compound *E*-5 (400 MHz, $CDCl_3$, 25 °C).**

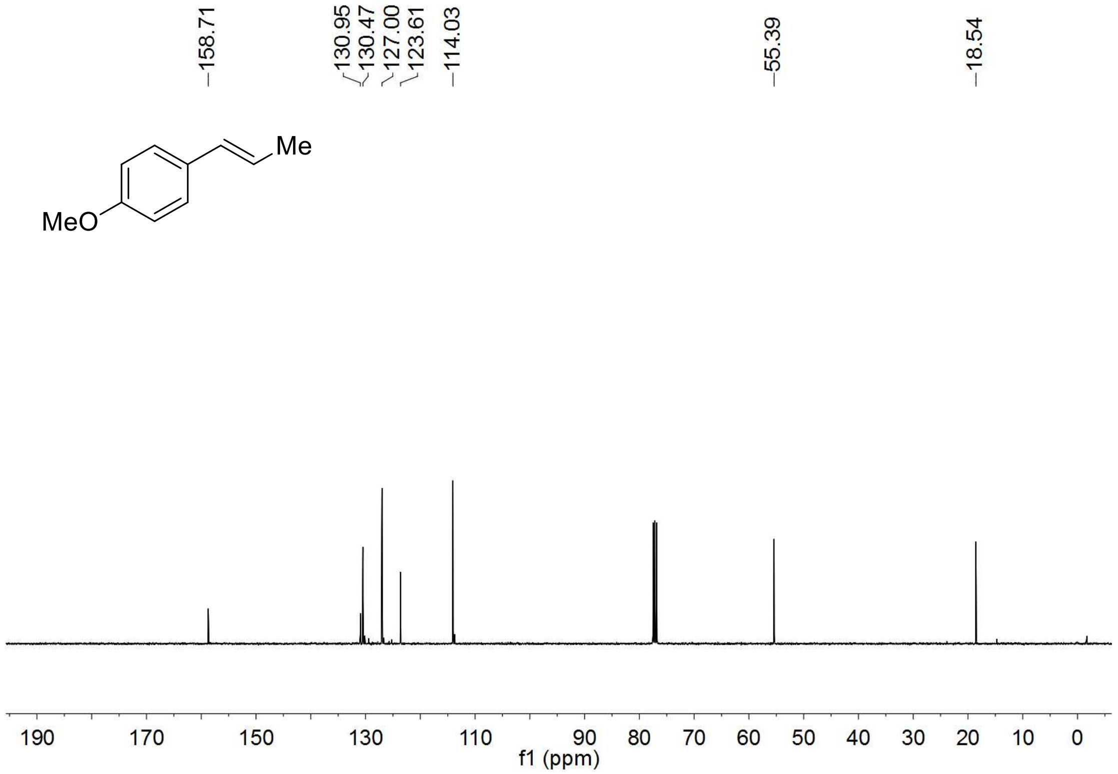

Supplementary Figure S156. **^{13}C NMR spectrum for compound *E*-5 (100 MHz, $CDCl_3$, 25 °C).**

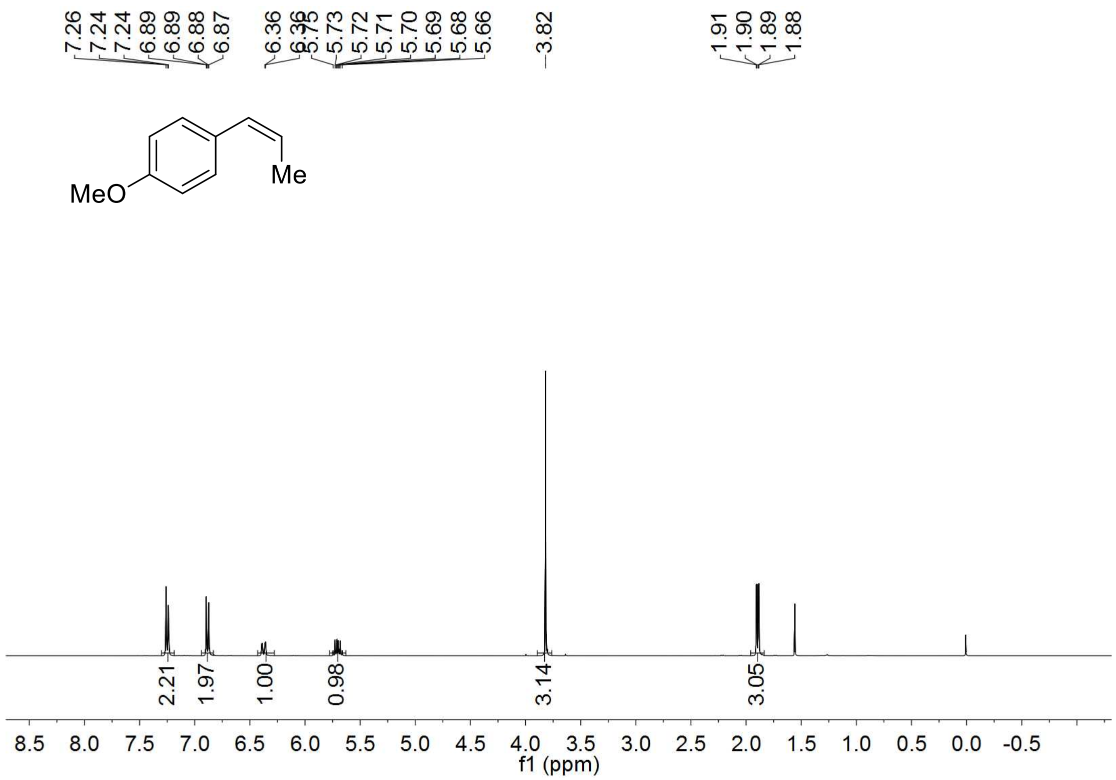

Supplementary Figure S157. ^{1}**H NMR spectrum for compound *Z*-5 (400 MHz, $CDCl_3$, 25 °C).**

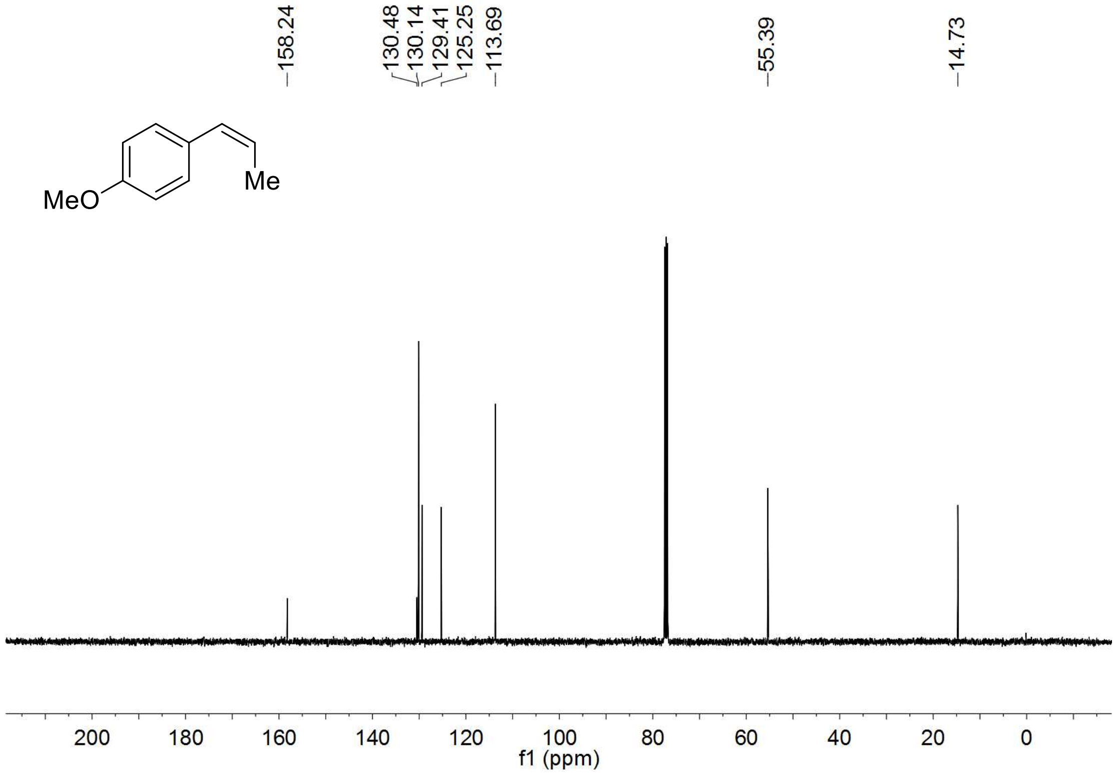

Supplementary Figure S158. ^{13}**C NMR spectrum for compound *Z*-5 (100 MHz, $CDCl_3$, 25 °C).**

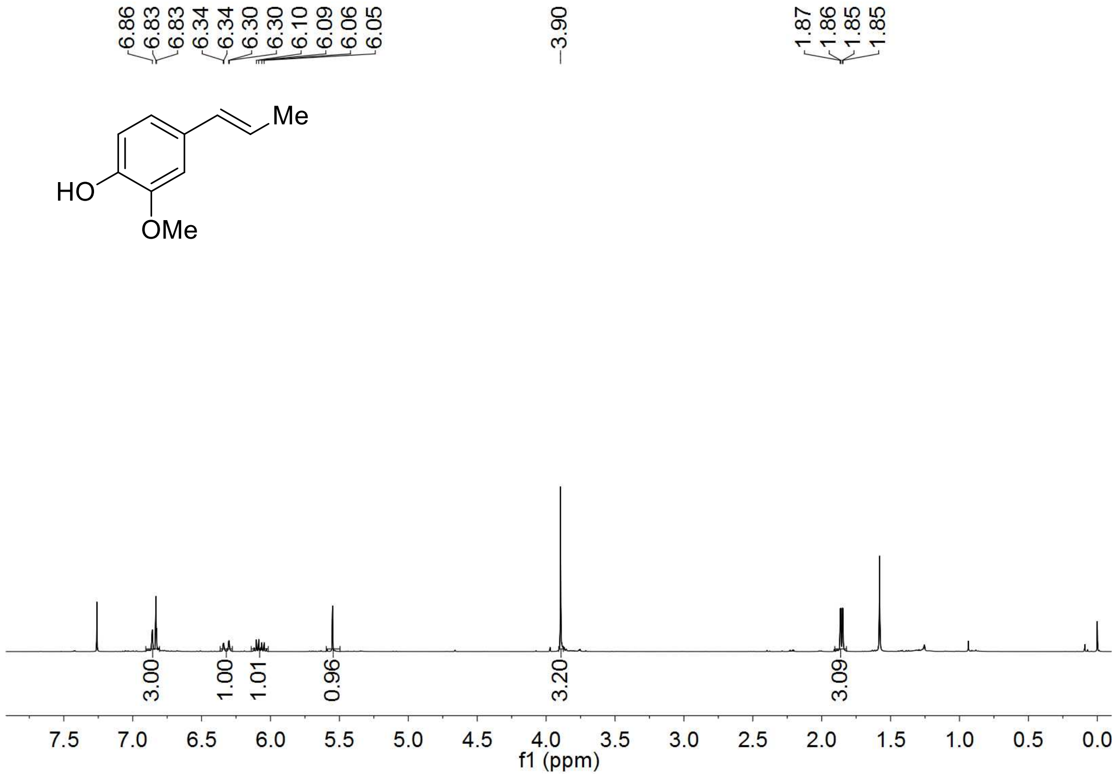

Supplementary Figure S159. **^{1}H NMR spectrum for compound *E*-7 (400 MHz, $CDCl_3$, 25 °C).**

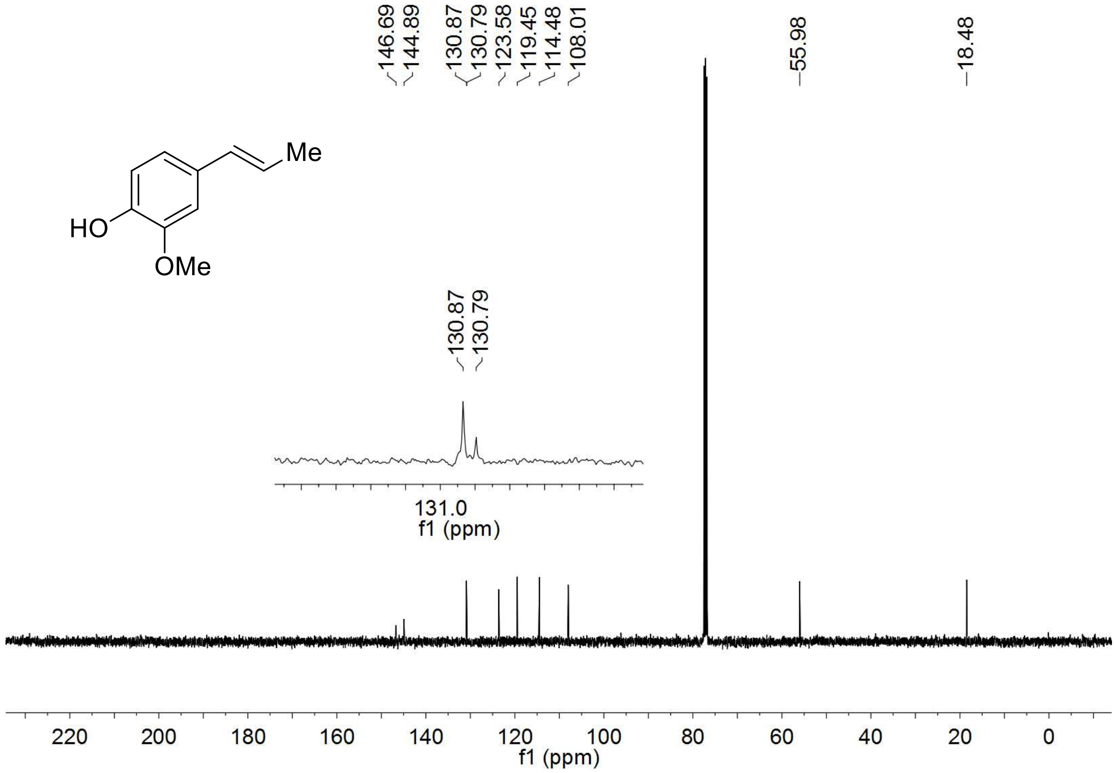

Supplementary Figure S160. **^{13}C NMR spectrum for compound *E*-7 (100 MHz, $CDCl_3$, 25 °C).**

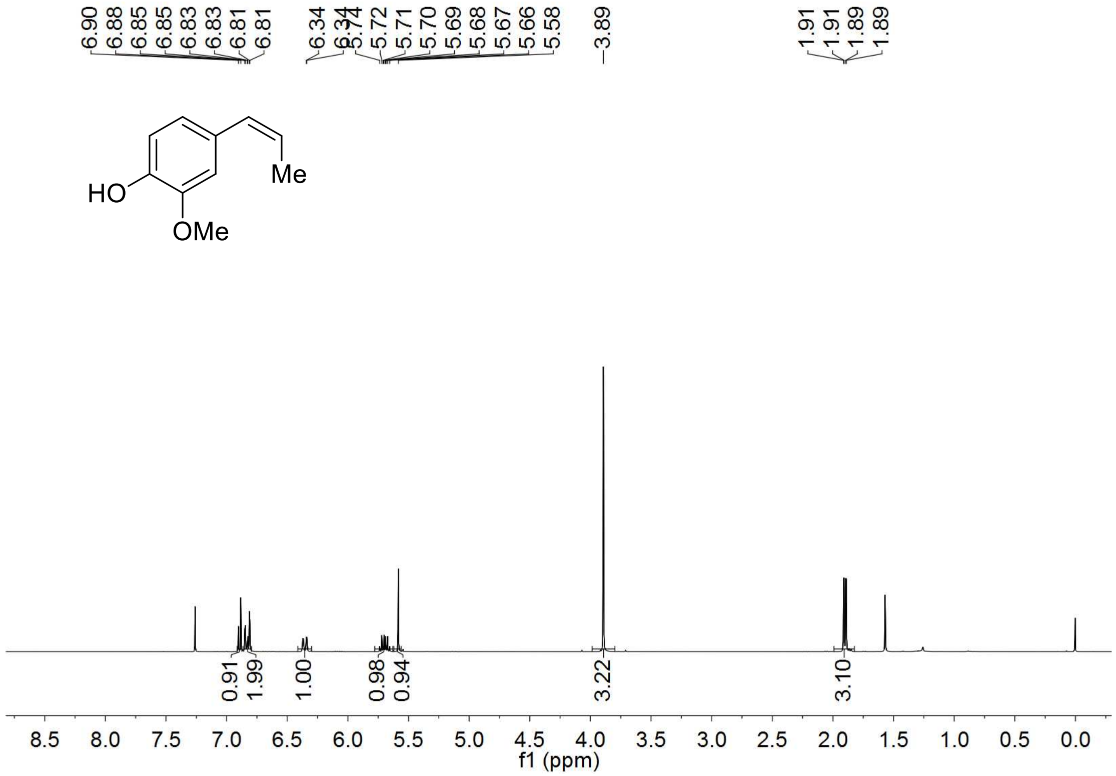

Supplementary Figure S161. ^{1}**H NMR spectrum for compound** ***Z*****-7 (400 MHz,** $CDCl_3$**, 25 °C).**

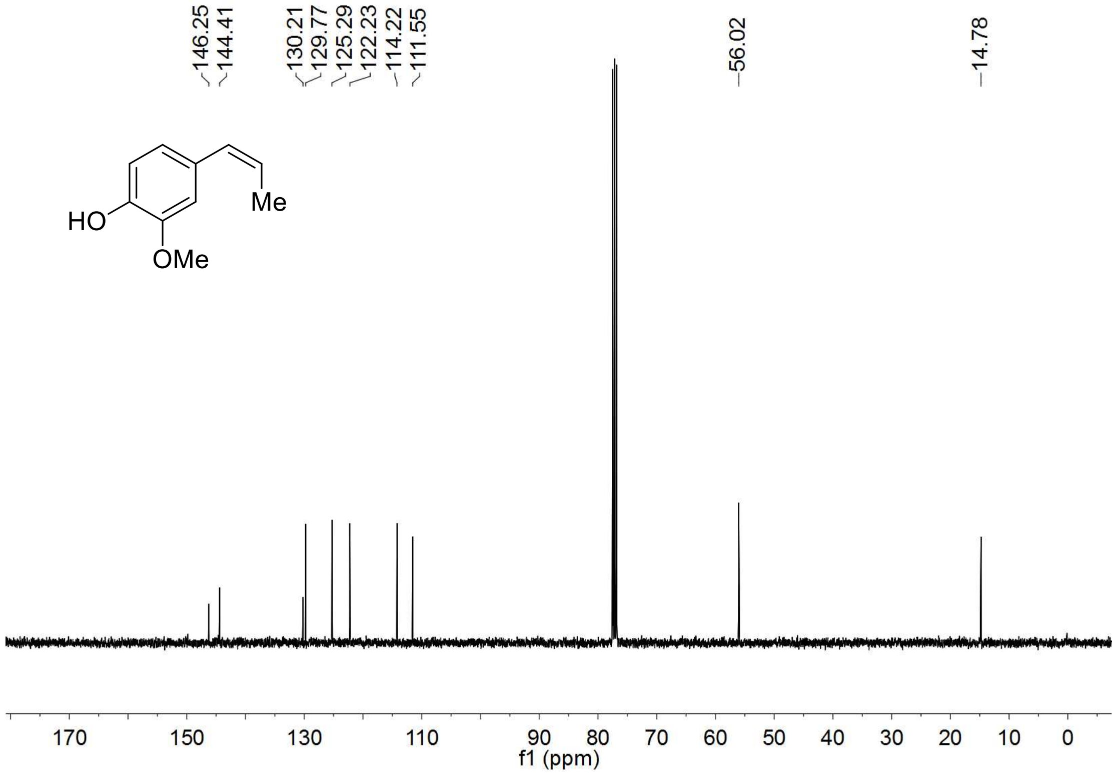

Supplementary Figure S162. ^{13}**C NMR spectrum for compound** ***Z*****-7 (100 MHz,** $CDCl_3$**, 25 °C).**

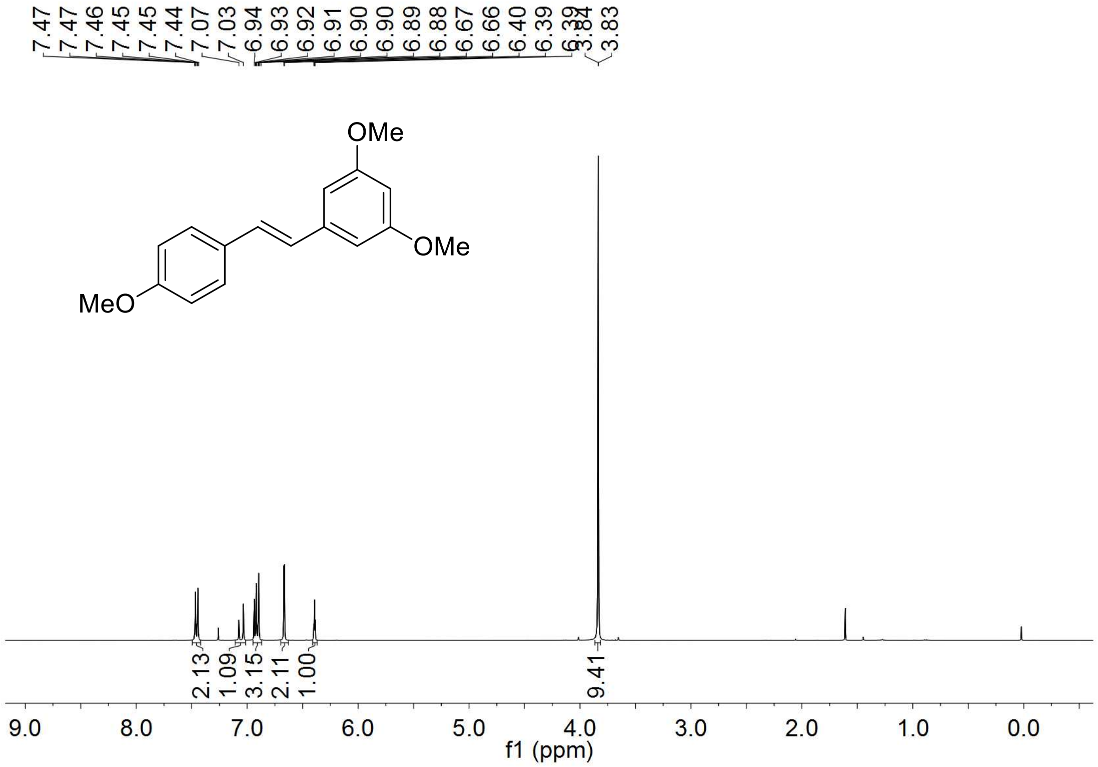

Supplementary Figure S163. ^{1}**H NMR spectrum for compound** ***E*****-9 (400 MHz, CDCl$_3$, 25 °C).**

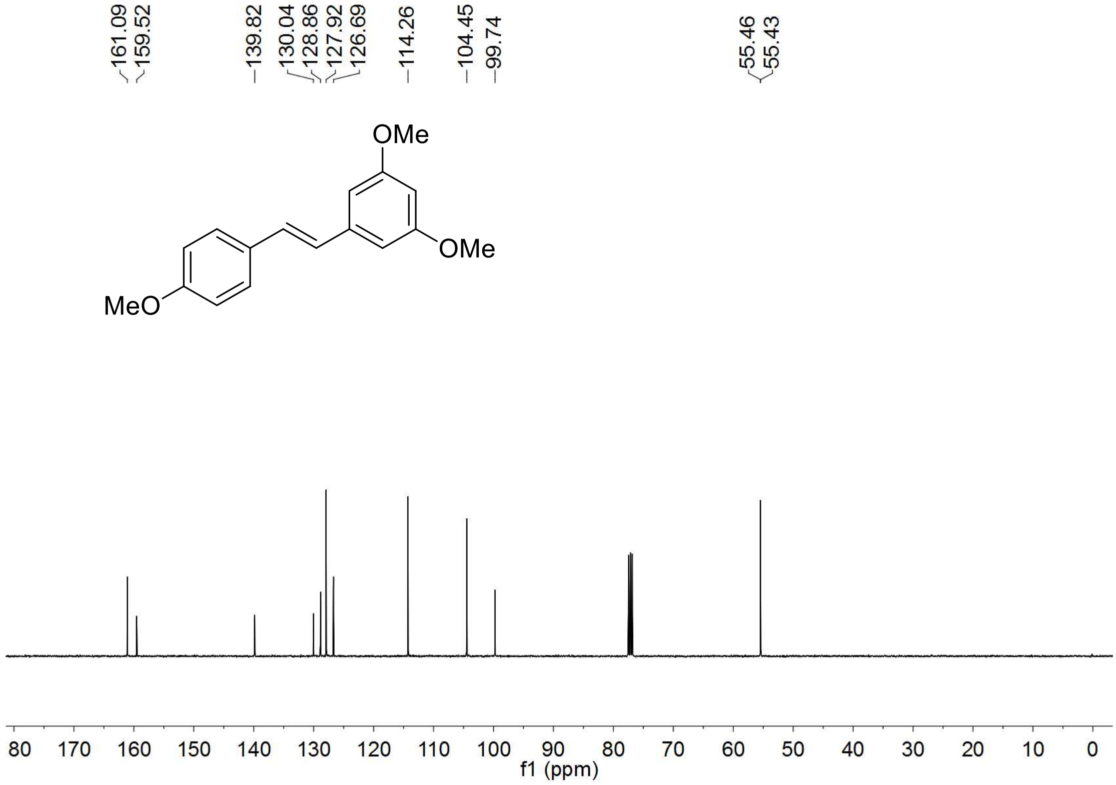

Supplementary Figure S164. ^{13}**C NMR spectrum for compound** ***E*****-9 (100 MHz, CDCl$_3$, 25 °C).**

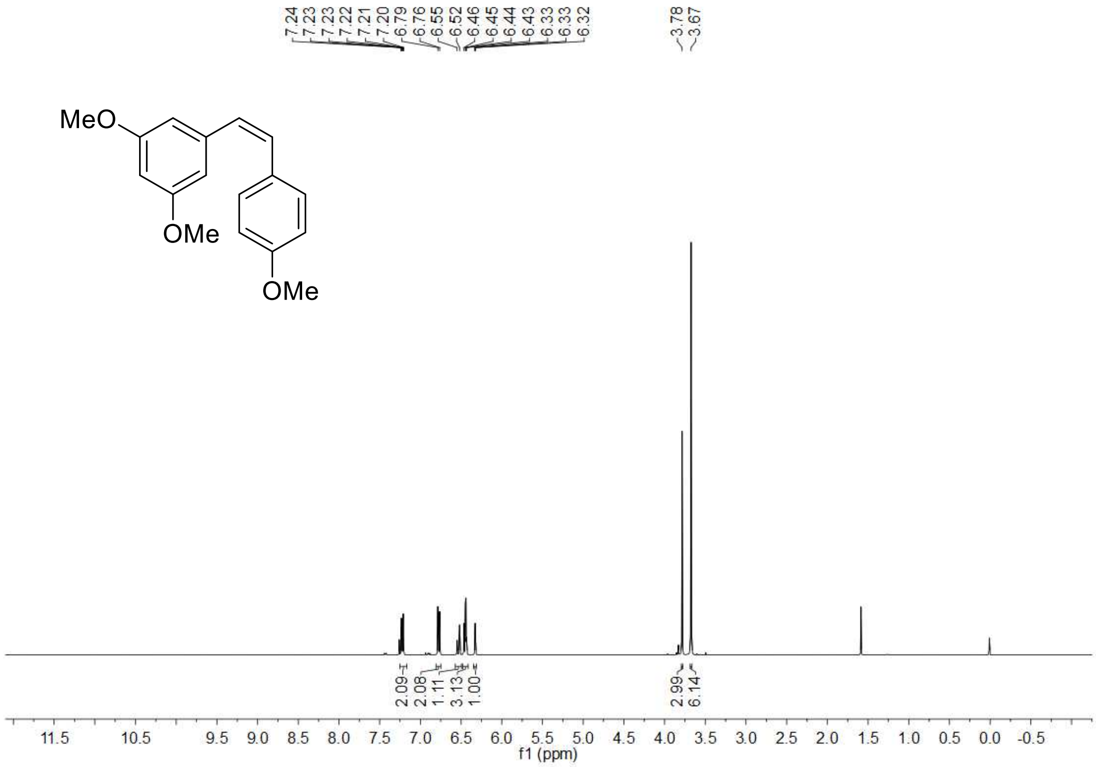

Supplementary Figure S165. ^{1}H NMR spectrum for compound *Z*-9 (400 MHz, $CDCl_3$, 25 °C).

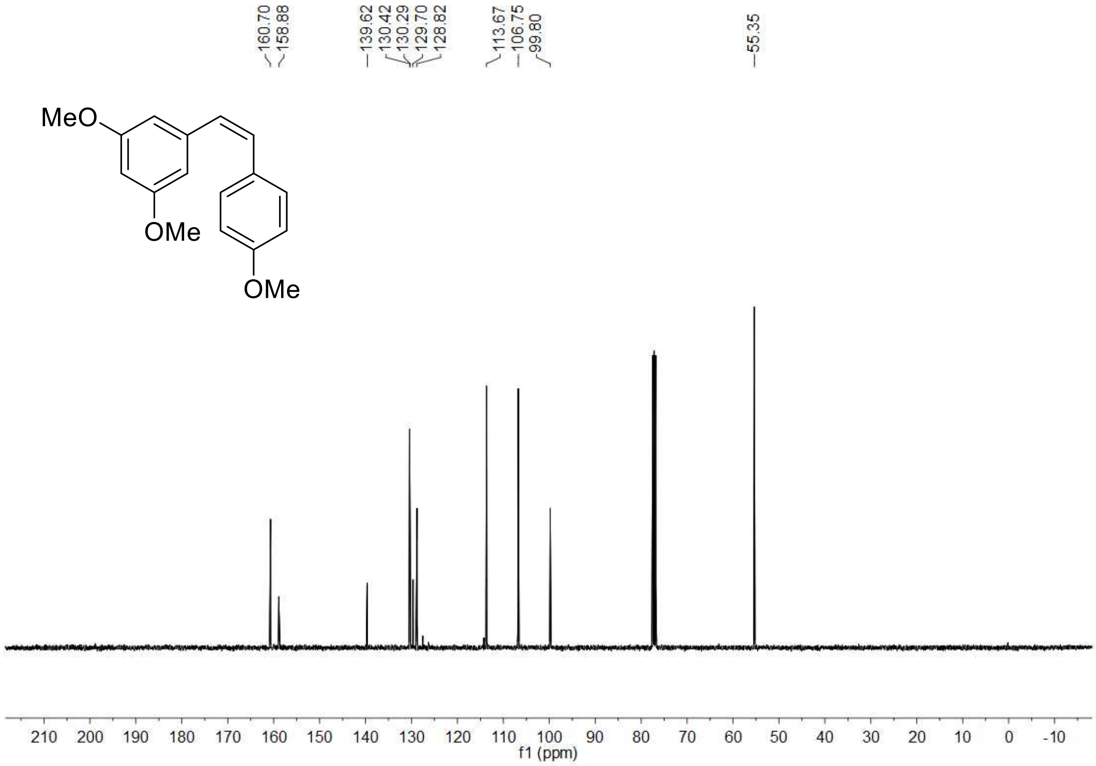

Supplementary Figure S166. ^{13}C NMR spectrum for compound *Z*-9 (100 MHz, $CDCl_3$, 25 °C).

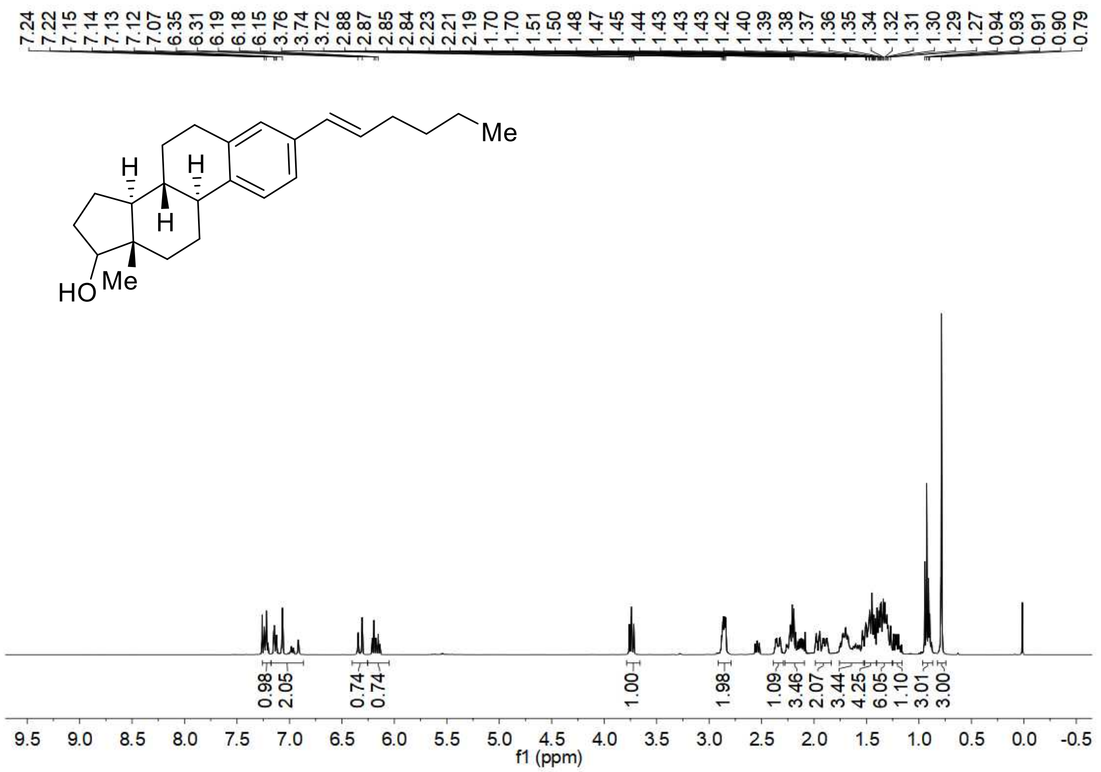

Supplementary Figure S167. **^{1}H NMR spectrum for compound *E*-11 (400 MHz, $CDCl_3$, 25 °C).**

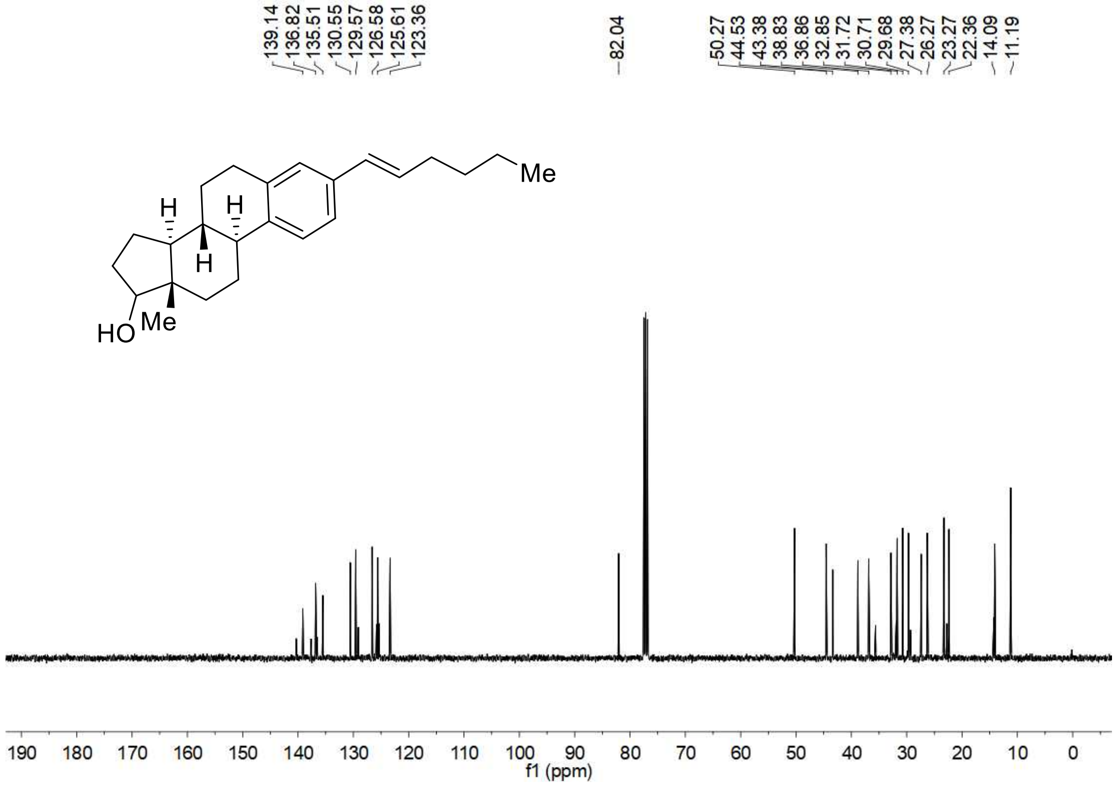

Supplementary Figure S168. **^{13}C NMR spectrum for compound *E*-11 (100 MHz, $CDCl_3$, 25 °C).**

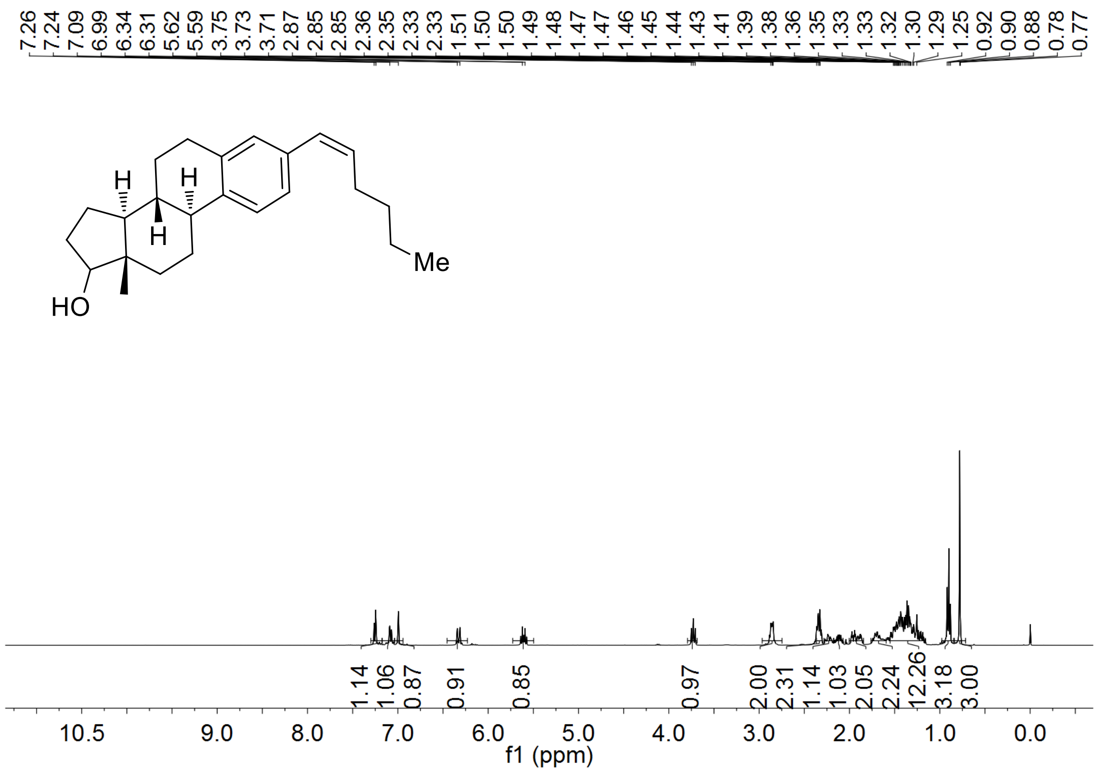

Supplementary Figure S169. **^{1}H NMR spectrum for compound *Z*-11 (400 MHz, $CDCl_3$, 25 °C).**

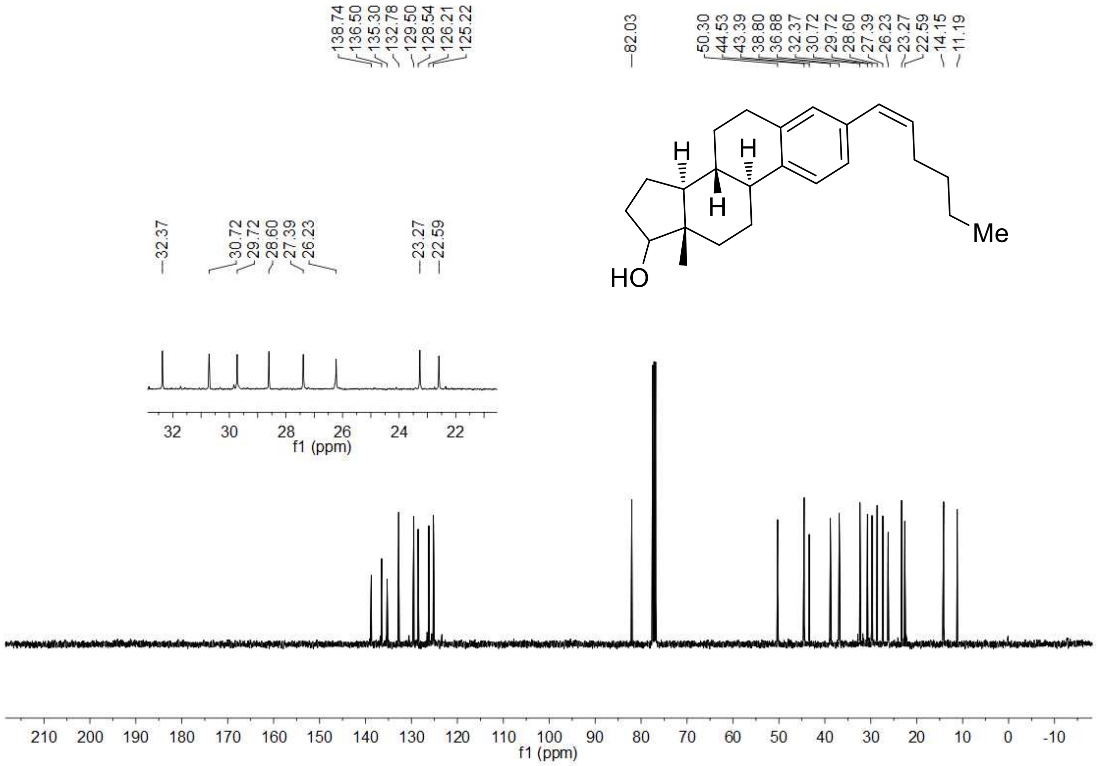

Supplementary Figure S170. **^{13}C NMR spectrum for compound *Z*-11 (100 MHz, $CDCl_3$, 25 °C).**

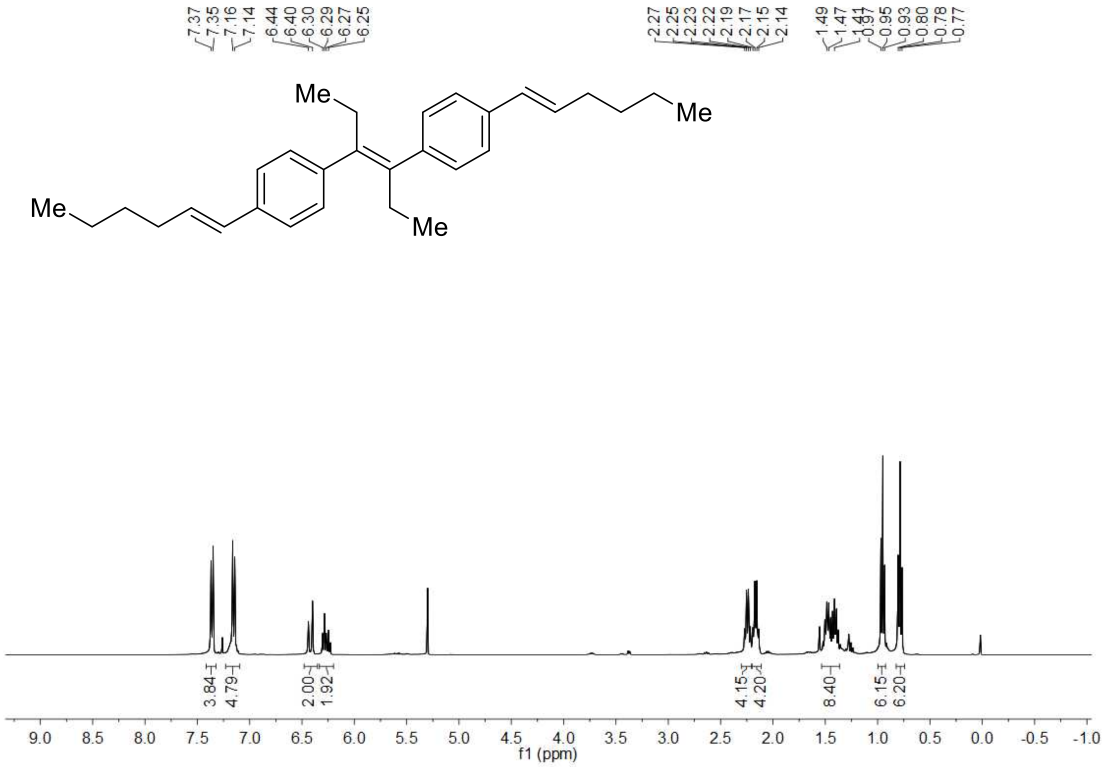

Supplementary Figure S171. ^{1}H NMR spectrum for compound *E*-13 (400 MHz, $CDCl_3$, 25 °C).

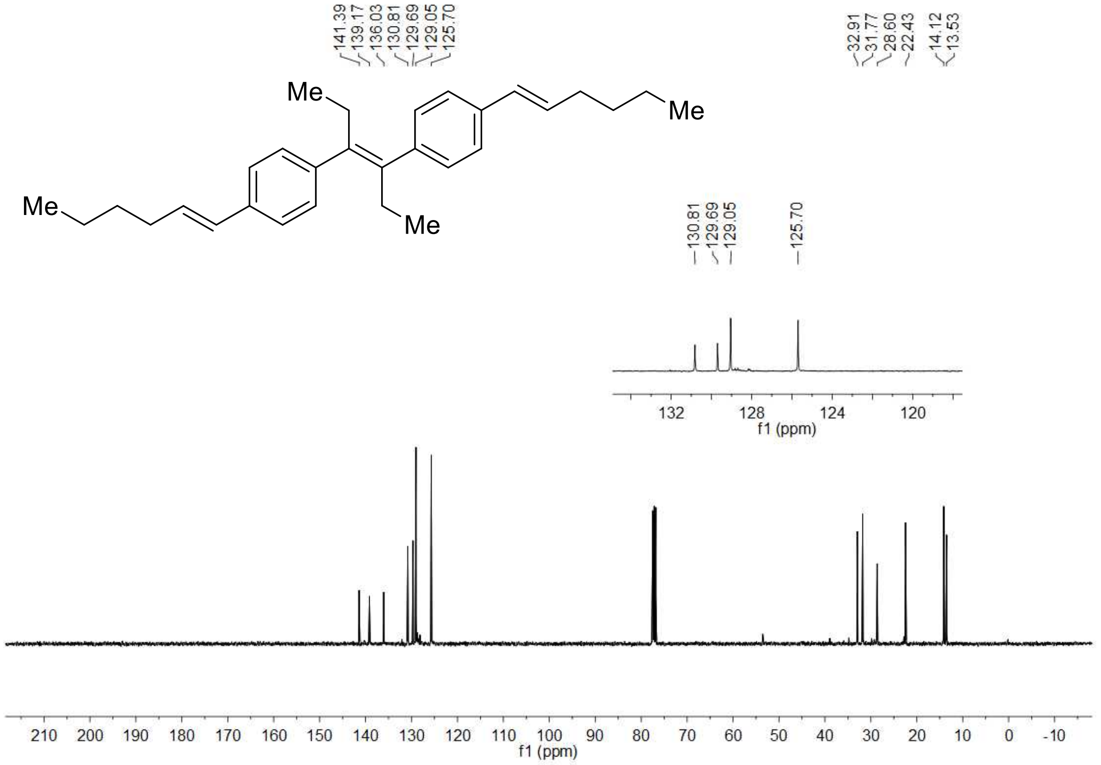

Supplementary Figure S172. ^{13}C NMR spectrum for compound *E*-13 (100 MHz, $CDCl_3$, 25 °C).

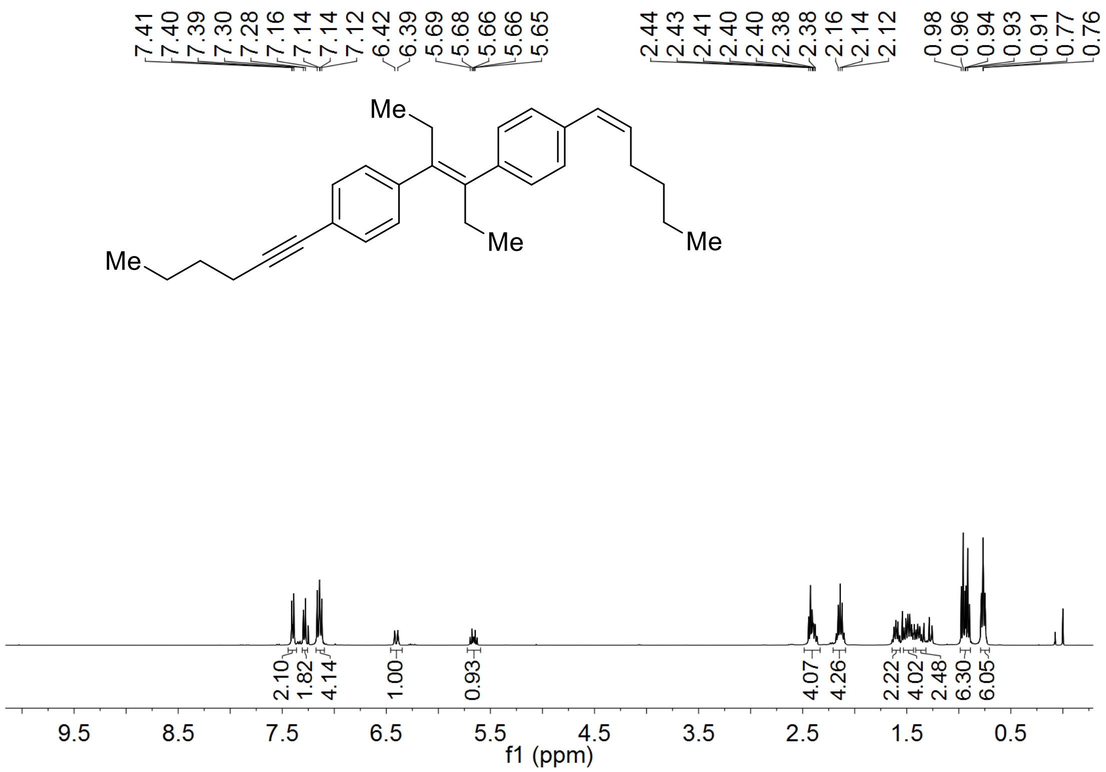

Supplementary Figure S173. ^{1}**H NMR spectrum for compound** ***Z*-13 (400 MHz, CDCl$_3$, 25 °C).**

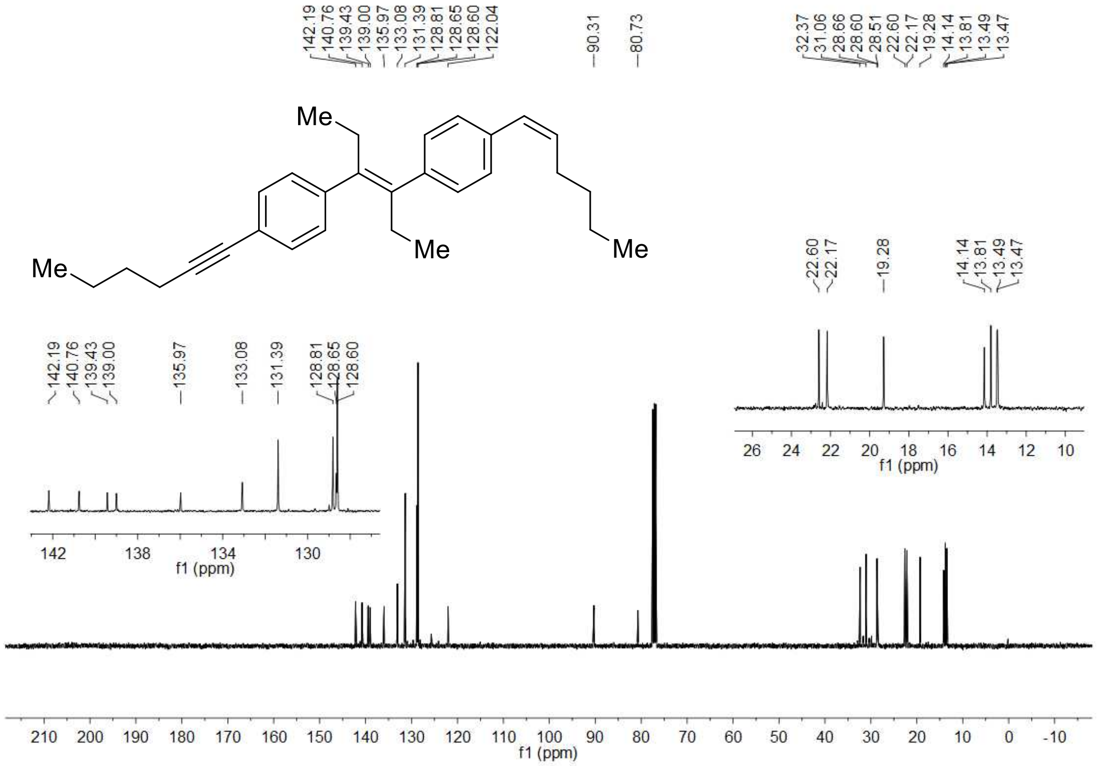

Supplementary Figure S174. ^{13}**C NMR spectrum for compound** ***Z*-13 (100 MHz, CDCl$_3$, 25 °C).**

13. Supplementary References

1. Zhao, D., Candish, L., Paul, D. & Glorius, F. *N*-Heterocyclic Carbenes in Asymmetric Hydrogenation. *ACS Catal*. **6**, 5978–5988 (2016).
2. Zhao, L. et al. Cyclic (Alkyl)(amino)carbene Ligand-Promoted Nitro Deoxygenative Hydroboration with Chromium Catalysis: Scope, Mechanism, and Applications. *J. Am. Chem. Soc*. **143**, 1618−1629 (2021).
3. Chu, J., Munz, D., Jazzar, R., Melaimi, M. & Bertrand, G. Synthesis of Hemilabile Cyclic (Alkyl)(amino)carbenes (CAACs) and Applications in Organometallic Chemistry. *J. Am. Chem. Soc*. **138**, 7884−7887 (2016).
4. Namba, K., Wang, J., Cui, S. & Kishi, Y. Surprisingly Efficient Catalytic Cr-Mediated Coupling Reactions. *Org. Lett*. **7**, 5421–5424 (2005).
5. Fu, S. et al. Ligand-Controlled Cobalt-Catalyzed Transfer Hydrogenation of Alkynes: Stereodivergent Synthesis of *Z*- and *E*-Alkenes. *J. Am. Chem. Soc.* **138**, 8588−8594 (2016).
6. Lvanov, A. et al. Synthesis and phosphatase inhibitory activity of 3-alkynylestrones and their derivatives. *RSC Adv*. **6**, 11118–11127 (2016).
7. Chiu, H., Tonks, D. Trimethylsilyl-Protected Alkynes as Selective Cross-Coupling Partners in Titanium-Catalyzed [2+2+1] Pyrrole Synthesis. *Angew. Chem. Int. Ed*. **57**, 6090–6094 (2018).
8. Liu, Y., Hu, L., Chen, H. & Du, H. An Alkene-Promoted Borane-Catalyzed Highly Stereoselective Hydrogenation of Alkynes to Give *Z*- and *E*-Alkenes.

Chem. Eur. J. **21**, 3495–3501 (2015).

9. Gong, D., Hu, B., Yang, W., Kong, D., Xia, H. & Chen, D. A Bidentate Ru(II)-NC Complex as a Catalyst for Semihydrogenation of Alkynes to (*E*)-Alkenes with Ethanol. *Organometallics*, **39**, 862−869 (2020).
10. Manna, S. & Antonchinck, A. Catalytic Transfer Hydrogenation Using Biomass as Hydrogen Source. *ChemSusChem*, **12**, 3094–3098 (2019).
11. Martina, K., Baricco, F., Caporaso, M., Berlier, G. & Cravotto, G. Cyclodextrin-Grafted Silica-Supported Pd Nanoparticles: An Efficient and Versatile Catalyst for Ligand-Free C−C Coupling and Hydrogenation. *ChemCatChem* **8**, 1176–1184 (2016).
12. Murugesan, K. et al. Nickel-Catalyzed Stereodivergent Synthesis of *E*- and *Z*-Alkenes by Hydrogenation of Alkynes. *ChemSusChem*, **12**, 3363–3369 (2019).
13. Yao, C., Li, Q., Wang, M., Ning, X. & Kang, Y. (*E*)-Specific direct Julia-olefination of aryl alcohols without extra reducing agents promoted by bases. *Chem. Commun.* **51**, 7729–7732 (2015).
14. Luo, X., Chen, X., Chen, L., Zhang, K. & Li, Y. Xanthate-mediated synthesis of (*E*)-alkenes by semi-hydrogenation of alkynes using water as the hydrogen donor. *Chem. Commun.* **55**, 2170–2173 (2019).
15. Westman, J. An Efficient Combination of Microwave Dielectric Heating and the Use of Solid-Supported Triphenylphosphine for Wittig Reactions. *Org. Lett.* **3**, 3745−3747 (2001).
16. Andrews, P., Latham, C., Magre, M., Willcox, D. & Woodward, S.

$ZrCl_2(\eta\text{-}C_5Me_5)_2\text{-}AlHCl_2\cdot(THF)_2$: efficient hydroalumination of terminal alkynes and cross-coupling of the derived alanes. *Chem. Commun.* **49**, 1488–1490 (2013).

17. Yu, X., Zhao, H., Li, P. & Koh, M. Iron-Catalyzed Tunable and Site-Selective Olefin Transposition. *J. Am. Chem. Soc*. **142**, 18223−18230 (2020).

18. Yu, W., Liu, L., Huang, T., Zhou, X. & Chen, T. Palladium-Catalyzed Decarbonylative Heck Coupling of Aromatic Carboxylic Acids with Terminal Alkenes. *Org. Lett*. **22**, 7123−7128 (2020).

19. Birepinte, M., Liautard, V., Chabaud, L. & Pucheault, M. Zirconium-Catalyzed Synthesis of Alkenylaminoboranes: From a Reliable Preparation of Alkenylboronates to a Direct Stereodivergent Access to Alkenyl Bromides. *Org. Lett.* **22**, 2838–2843 (2020).

20. Akporji, N. et al. Selective Deprotection of the Diphenylmethylsilyl (DPMS) Hydroxyl Protecting Group under Environmentally Responsible, Aqueous Conditions. *ChemCatChem* **11**,5743–5747 (2019).

21. Giraud, A., Provot, O., Hamzé, A., Brion, J-D. & Alami, M. One-pot hydrosilylation–protodesilylation of functionalized diarylalkynes: a highly selective access to *Z*-stilbenes. Application to the synthesis of combretastatin A-4. *Tetrahedron Letters*. **49**, 1107–1110 (2008).

22. Xiao, B. et al. Copper Nanocrystal Plane Effect on Stereoselectivity of Catalytic Deoxygenation of Aromatic Epoxides. *J. Am. Chem. Soc*. **137**, 3791−3794 (2015).

23. Hancker, S., Neuman, H. & Beller, M. Development of a Palladium-Catalyzed Process for the Synthesis of *Z*-Alkenes by Sequential Sonogashira–Hydrogenation Reaction. *Eur. J. Org. Chem*. **38**, 5253−5259 (2018).

24. Du, X., Hou, W., Zhang, Y., Huang, Z. Pincer cobalt complex-catalyzed

Z-selective hydrosilylation of terminal alkynes. *Org. Chem. Front.* **4**, 1517–1521 (2017).

25. Miyaura, M., Satoh, M., Suzuki, A. Stereo- and regiospecific syntheses to provide conjugated (*E,Z*)- and (*Z,Z*)-alkadienes, and arylated (*Z*)-alkenes in excellent yields via the palladium-catalyzed cross-coupling reactions of (*Z*)-1-alkenylboronates with 1-bromoalkenes and aryl iodides. *Tetrahedron Letters.* **27**, 3745–3748 (1986).

26. Krasovskiy, A., Haley, S., Voigtritter, K., Lipshutz, B. Stereoretentive Pd-Catalyzed Kumada–Corriu Couplings of Alkenyl Halides at Room Temperature. *Org. Lett.* **16**, 4066−4069 (2014).

27. Wissing, M., Niehues, M., Ravoo, B., Studer, A. Mixed AuPd Nanoparticles as Highly Active Catalysts for Alkyne Z-Semihydrogenation. *Eur. J. Org. Chem.* **26**, 3403–3409 (2018).

28. Carpita, A., Ribecai, A., Rosi, R. & Stabile. P. Synthesis of the racemic forms of carbon–carbon double bond locked analogues of strobilurins which are characterized by a 2-arylcyclopropane ring cis-substituted at C-1 by the methyl (*E*)-3-methoxypropenoate unit. *Tetrahedron* **58**, 3673–3680 (2002).

29. Moglie, Y., González-Soria, M., Martín-García, I., Radivoy, G. & Alonso, F. Catalyst- and solvent-free hydrophosphination and multicomponent hydrothiophosphination of alkenes and alkynes†. *Green Chem.* **18**, 4896–4907 (2016).

30. Wate, C. & Hashimota, T. Organoiodine-Catalyzed Enantioselective Intermolecular Oxyamination of Alkenes. *J. Am. Chem. Soc.* **143**, 1745–1751 (2021).

31. Hor, T. S. A. & Chee, S. Substituted metal carbonyls: III. Chromium, molybdenum and tungsten tricarbonyl complexes containing bipyridyl and a

unidentate diphosphine: facile synthesis via trimethylamine *N*-oxide-induced decarbonylations. *J. Organomet. Chem*. **331**, 23–28 (1987).

32. Gradert, C., Krahmer, J., Sönnichsen, F., Näther, C. & Tuczek, F. Small-Molecule Activation with Molybdenum(0) Complexes Supported by Mixed Imidazol-2-Ylidene/Phosphanyl Hybrid Ligands – Electronic and Structural Consequences of Substituting a Phosphane by a Carbene Group. *Eur. J. Inorg. Chem*. 3943–3955 (2013).
33. Lee, S. X. et al. Synthesis of phenanthroline-based ligand and its UV activable tetracarbonyl photoCORMs based on chromium, molybdenum, and tungsten as cytotoxic and antimicrobial agents. *J. Organomet. Chem.* **954–955**, 122103 (2021).
34. Kapat, A., Sperger, T., Guven, S. & Schoenebeck, F. *E*-Olefins through intramolecular radical relocation. *Science* **363**, 391–396 (2019).
35. Cho, Y., Kim, H., An, H., Ahn, K. & Kang, E. Cycloaddition Reactions of Alkene Radical Cations using Iron(III)-Phenanthroline Complex. *Adv. Synth. Catal*. **362**, 2183–2188 (2020).
36. Thiel, N., Kaewmee, B., Ngoc, T. & Teichert, J. A Simple Nickel Catalyst Enabling an *E*-Selective Alkyne Semihydrogenation. *Chem. Eur. J.* **26**, 1597–1603 (2020).
37. Gaussian 16, Revision E.01: Frisch, M. J., Gaussian, Inc., Wallingford, CT, (2010)
38. Lee, C., Yang, W. & Parr, R. G. Development of the Colle-Salvetti correlation energy formula into a functional of the electron density. *Phys. Rev. B* **37**, 785–789 (1988).

39. Stephens, P. J., Devlin, F. J., Chabalowski, C. F. & Frisch, M. J. Ab Initio Calculation of Vibrational Absorption and Circular Dichroism Spectra Using Density Functional Force Fields. *J. Phys. Chem.* **98**, 11623–11627 (1994).

40. Grimme, S., Antony, J., Ehrlich, S. & Krieg, H. A consistent and accurate ab initio parametrization of density functional dispersion correction (DFT-D) for the 94 elements H-Pu. *J. Chem. Phys.* **132**, 154104 (2010).

41. Grimme, S. Semiempirical GGA-type density functional constructed with a long-range dispersion correction. *J. Comput. Chem.* **27**, 1786–1799 (2006).

42. Grimme, S. Accurate description of van der Waals complexes by density functional theory including empirical corrections. *J. Comput. Chem.* **25**, 1463–1473 (2004).

43. Legault, C. Y. CYL-View, 1.0b; Université de Sherbrooke: Sherbrooke, Quebec, Canada, (2009).

44. Falivene, L. et al. Towards the online computer-aided design of catalytic pockets. *Nat. Chem.* **11**, 872–879 (2019).
