# Supplementary material for: Chromium-catalyzed stereodivergent E- and Z-selective alkyne hydrogenation controlled by cyclic (alkyl)(amino)carbene ligands
Source: Nat Commun. 2023 Feb 22;14:990. doi: 10.1038/s41467-023-36677-9 (PMC9947122; doi:10.1038/s41467-023-36677-9)
Supplement: Supplementary file 2 — Description of Additional Supplementary Files [file 41467_2023_36677_MOESM2_ESM.pdf]

Description of Additional Supplementary Files

Supplementary Data 1: Cartesian Coordinates of the structures
