# Supplementary material for: Chromium-catalyzed stereodivergent E- and Z-selective alkyne hydrogenation controlled by cyclic (alkyl)(amino)carbene ligands
Source: Nat Commun. 2023 Feb 22;14:990. doi: 10.1038/s41467-023-36677-9 (PMC9947122; doi:10.1038/s41467-023-36677-9)
Supplement: Supplementary file 3 — Supplementary Data 1 [file 41467_2023_36677_MOESM3_ESM.pdf]

Chromium-catalyzed stereodivergent *E*- and *Z*-selective alkyne hydrogenation controlled by cyclic (alkyl)(amino)carbene ligands

Liang Ling,[1] Chenyang Hu,[2] Linhong Long[3], Xue Zhang,[1] Lixing Zhao,[1] Liu Leo Liu,[2] Hui Chen[3]*, Meiming Luo[1]* & Xiaoming Zeng[1]*

[1]Key Laboratory of Green Chemistry & Technology, Ministry of Education, College of Chemistry, Sichuan University, Chengdu 610064, China
[2]Shenzhen Grubbs Institute and Department of Chemistry, Southern University of Science and Technology; Shenzhen 518055, China
[3]Beijing National Laboratory for Molecular Sciences (BNLMS), Key Laboratory of Photochemistry, CAS Research/Education Center for Excellence in Molecular Sciences, Institute of Chemistry, Chinese Academy of Sciences, Beijing 100190, China

Cartesian Coordinates for steric map of the related complexes from 1a and 1b, and the related intermediates and transition states for stereoisomerization

***N*-Dipp (coordinated with two THF molecules):**

Cr	6.31332600	6.18359500	1.90878200
N	3.95158700	6.27466000	2.21752300
C	6.06806200	7.38235900	3.44691000
C	4.08052900	2.09316100	1.81632700
H	4.58737000	1.57576100	2.64604700
H	3.26692000	1.43238500	1.47713300
H	4.79971600	2.19863300	0.99080700
C	2.54773600	3.28937600	3.42047300
H	2.96774400	2.66219400	4.22397300
H	2.27546100	4.25476400	3.86621700
H	1.62363600	2.80773600	3.06091400
C	3.56261900	3.45853700	2.28044600

H	4.42241500	4.00949600	2.68671900
C	3.04123600	4.31125300	1.13284300
C	2.38601300	3.73851400	0.03957700
H	2.19355500	2.66403900	0.03403500
C	1.99063500	4.50894300	-1.05656000
H	1.47517600	4.04512000	-1.90049300
C	2.29505900	5.86950300	-1.07677100
H	2.03319400	6.46376000	-1.95549300
C	2.93982200	6.49463100	-0.00229600
C	3.25335500	5.71682600	1.14212200
C	3.34389300	7.95749100	-0.11649000
H	3.76874200	8.26498300	0.84983200
C	2.15962800	8.87756800	-0.44605900
H	2.47401100	9.93350700	-0.42700200
H	1.75712000	8.67267500	-1.45123500
H	1.33576100	8.75637400	0.27239700
C	4.46520900	8.11767700	-1.15536600
H	4.75574700	9.17456200	-1.25716200
H	5.35386500	7.55052900	-0.84695500
H	4.14375100	7.75758900	-2.14658400
C	3.56321700	6.95177400	3.23961700
C	2.09439300	7.36913900	3.54646400
C	1.03617400	6.65054600	2.68239100
H	0.04038000	6.92476600	3.06449700
H	1.07644700	6.94117600	1.62831700
H	1.12138500	5.55955600	2.72707300
C	1.72802700	7.06925000	5.01599700
H	2.38120000	7.58211600	5.72974100
H	0.69846900	7.41254900	5.20213400
H	1.76301600	5.99055100	5.22899900
C	1.91934600	8.89081400	3.29405000
H	2.32174800	9.50246200	4.10864500
H	2.40039000	9.20620000	2.35833500
H	0.84512100	9.11951500	3.21092300

C	4.72505700	7.29462400	4.20744000
C	4.79738600	6.11129800	5.22144800
H	5.78431700	6.09777100	5.69975400
H	4.03377200	6.16229400	6.01308600
H	4.68278700	5.15590200	4.69259400
C	6.11759900	9.06673600	5.23274400
C	6.28104100	10.59071600	5.12949100
H	5.89110700	10.97295600	4.17879300
H	5.72732100	11.08381600	5.94429400
H	7.33830000	10.88374600	5.21398000
C	6.56758500	8.66431200	6.65247100
H	6.46834800	7.58783500	6.83484700
H	7.62068600	8.94065600	6.80610200
H	5.96950400	9.19539100	7.41090100
N	6.83576400	8.32303500	4.17445000
C	4.65927700	8.66162200	4.92902400
H	4.25875400	9.40821600	4.23504800
H	4.04241300	8.67994400	5.83956700
C	9.26593500	8.01650100	4.38466400
C	10.56578300	8.43478200	4.06741000
H	11.41907900	7.94810300	4.54653300
C	10.78747000	9.44929300	3.13993700
H	11.80559600	9.77449900	2.91006600
C	9.70095800	10.02685600	2.48660700
H	9.87808900	10.79171800	1.72650300
C	8.38572600	9.63899000	2.77686600
C	7.25307700	10.22177500	1.94524200
H	6.31121800	9.86720300	2.38411000
C	7.30930500	9.65553800	0.51894400
H	6.48320600	10.05411500	-0.09065900
H	8.25743500	9.91586200	0.01929900
H	7.21456600	8.55874700	0.55254200
C	7.25255500	11.75681800	1.91229500
H	6.35586600	12.12980700	1.39042100

H	7.26555600	12.19104700	2.92252000
H	8.12912800	12.14953100	1.37204900
C	9.08173400	6.80328100	5.28027500
H	8.00401600	6.69812900	5.45390900
C	9.78869900	6.92584300	6.63539300
H	9.47250800	7.82880500	7.17828500
H	9.56429600	6.05249700	7.26997600
H	10.88375300	6.97355300	6.51972900
C	9.54692500	5.54478000	4.53119200
H	9.38722300	4.64468500	5.14727400
H	8.98532700	5.43640900	3.59016400
H	10.62110900	5.59746800	4.28900200
C	8.15879800	8.66072300	3.77957000
C	7.87226300	5.84654300	-0.83091800
O	6.65718200	5.45487800	-0.15861600
C	5.81793900	4.66892800	-1.03156700
C	6.41799700	4.84625000	-2.42127800
C	7.90975500	5.01142500	-2.10837600
H	7.82206300	6.92684000	-1.04545400
H	8.71575900	5.67122400	-0.14782100
H	5.85129000	3.61604600	-0.70234400
H	4.78749900	5.02616800	-0.94146600
H	6.19749900	3.99819300	-3.08550900
H	6.01982500	5.76045700	-2.89004500
H	8.37168200	4.02950000	-1.91366900
H	8.47812700	5.50122300	-2.91214200
C	7.80036100	3.11112900	1.62289800
O	6.90211400	3.89160100	2.41643300
C	6.87522800	3.40783900	3.77231700
C	7.92455300	2.29688400	3.84475100
C	7.99052000	1.80877600	2.39288500
H	7.35756600	2.97299400	0.62746500
H	8.75455600	3.65819500	1.50644700
H	7.07003800	4.25363200	4.44422300

H	5.86309100	3.02606100	3.98413500
H	8.89839400	2.70914600	4.14902800
H	7.65140700	1.50754700	4.56001000
H	8.93377200	1.30046600	2.14370900
H	7.16047800	1.11691600	2.17762100

P-Ph$_2$ (coordinated with two THF molecules):

Cr	3.10955100	16.21419500	10.90481700
P	1.93462100	17.39588700	9.00649900
N	0.71349700	14.40597400	11.72987100
C	1.48101100	15.07977200	10.76389700
C	0.74491200	14.86689400	9.42488800
C	-0.57918800	14.15288500	9.81396300
H	-1.38561300	14.89609700	9.91360500
H	-0.90855800	13.41519700	9.06572900
C	-0.33530400	13.50579000	11.19648300
C	0.47098400	16.25254100	8.78590200
H	-0.34387200	16.73961000	9.34061200
H	0.15784700	16.19405200	7.72862400
C	1.57022600	14.00870300	8.44864700
H	2.52671800	14.50289200	8.22753400
H	1.03216600	13.83892000	7.49793800
H	1.81389600	13.03016500	8.88454400
C	-1.60690200	13.47498100	12.05140400
H	-1.38427500	13.15302700	13.08023200
H	-2.32521300	12.75866400	11.62298500
H	-2.09760900	14.45499100	12.09621500
C	0.17681000	12.05636700	11.07007900
H	1.12094000	12.00901600	10.51232500
H	-0.56262300	11.42668200	10.54928800
H	0.34739000	11.62103000	12.06513100
C	0.99684600	14.57616600	13.10965900
C	0.27713900	15.55472700	13.84539800
C	0.52944400	15.69954800	15.21657000

H	-0.03050500	16.44150800	15.79061700
C	1.49900800	14.92759300	15.85541300
H	1.67952600	15.04734000	16.92691500
C	2.25214400	14.01938100	15.11407700
H	3.03595000	13.44088900	15.60953400
C	2.02644800	13.83638100	13.74186000
C	-0.67510900	16.52051200	13.15469600
H	-0.86550400	16.12026200	12.15198700
C	0.01012900	17.87964700	12.94928400
H	-0.64070800	18.57213500	12.39188500
H	0.92998900	17.74722400	12.36383500
H	0.26454200	18.35283000	13.91314600
C	-2.01743100	16.67501800	13.87879300
H	-2.50058700	15.70085500	14.04998000
H	-2.70410900	17.29910300	13.28431900
H	-1.90029100	17.16557500	14.85874900
C	2.93115400	12.89569500	12.96102500
H	2.58133400	12.90616000	11.92208600
C	4.37551000	13.42049600	12.94458300
H	4.40448300	14.44158800	12.52870000
H	5.01601900	12.77967500	12.31611400
H	4.81416300	13.44449800	13.95613600
C	2.87985000	11.45638400	13.49127400
H	3.28883800	11.38642800	14.51251400
H	3.47465800	10.78481600	12.85063300
H	1.84921800	11.07244100	13.52129900
C	1.16836100	18.99436200	9.51478800
C	1.71295500	19.68217600	10.60851600
H	2.55301900	19.23842600	11.14619800
C	1.16859600	20.89735700	11.03227100
H	1.60129700	21.41921800	11.88970400
C	0.06366600	21.43641500	10.36738700
H	-0.36871900	22.38358100	10.69927000
C	-0.48914100	20.75775400	9.27548300

H	-1.35181900	21.17731500	8.75150700
C	0.06174900	19.54715600	8.84968700
H	-0.36576600	19.02891800	7.98771100
C	2.47499400	17.81720400	7.29210400
C	3.07930200	19.06579600	7.03457900
H	3.05778200	19.84189100	7.80402000
C	3.69774400	19.32801000	5.81287100
H	4.15065000	20.30733400	5.63581400
C	3.73737400	18.34732900	4.81276800
H	4.22452300	18.55175300	3.85660400
C	3.13894300	17.10662800	5.05206500
H	3.15275600	16.33450000	4.27805800
C	2.51588300	16.84350400	6.27447900
H	2.05937800	15.86596800	6.43701300
C	4.98475100	18.50014300	12.48754000
O	3.93035600	17.52622000	12.51384900
C	3.55715700	17.26407800	13.87693500
C	3.71601900	18.61864100	14.54804200
C	4.99944800	19.15160500	13.88642000
H	5.93639400	18.00007500	12.24509800
H	4.75433500	19.21257700	11.68030800
H	2.54042900	16.85946000	13.87399500
H	4.23424900	16.50569300	14.30933900
H	2.85044600	19.25399300	14.30063700
H	3.79005300	18.54888300	15.64268000
H	5.02884500	20.24943000	13.83343500
H	5.88527200	18.82329400	14.45165700
C	5.58608800	14.98163600	9.41747800
O	5.25589300	16.21263000	10.08113800
C	5.66561100	17.32143700	9.25375100
C	5.98225400	16.74408200	7.86207400
C	5.44407800	15.30684700	7.93766600
H	6.62044900	14.68625300	9.67760400
H	4.88599500	14.21855600	9.78606300

H	4.82857800	18.03432800	9.23323700
H	6.53980400	17.81215000	9.71356400
H	5.50511600	17.32456000	7.06103000
H	7.06881300	16.74255700	7.68299400
H	4.38220900	15.28685000	7.65519900
H	5.98784600	14.60505700	7.28886300

THF:

C	-1.18139600	1.79306400	0.00000000
O	0.23279500	1.82147800	0.00000000
C	0.75570000	3.13582000	0.00000000
C	-0.42659400	4.12920500	-0.00000100
C	-1.69666300	3.24872200	-0.00000100
H	-1.53498100	1.23872400	-0.88887900
H	-1.53498100	1.23872500	0.88887800
H	1.39870100	3.27236600	0.88895100
H	1.39870200	3.27236600	-0.88895100
H	-0.39372400	4.78358100	0.88318700
H	-0.39372400	4.78358000	-0.88319000
H	-2.32083500	3.44762000	0.88321400
H	-2.32083500	3.44761900	-0.88321700

TS-9P:

Cr	3.45918600	16.84045000	11.35703400
P	2.48426500	17.43500300	9.20624900
N	1.40989200	14.52407700	11.63732700
C	2.33196000	15.25567500	10.96348900
C	2.44126100	14.66280300	9.54055300
C	1.52242600	13.41472900	9.56840900
H	0.94250000	13.28847600	8.64150500
H	2.14015200	12.51132700	9.68600100
C	0.60703200	13.56309400	10.80432800

C	1.97613100	15.75500100	8.53260800
H	0.88443700	15.73699800	8.41841400
H	2.40003900	15.59863300	7.52780200
C	3.89511400	14.26189300	9.22193700
H	4.55410300	15.14343700	9.16670800
H	3.94950900	13.73923200	8.25136400
H	4.29607000	13.59792000	10.00272900
C	-0.75917000	14.17246300	10.44406100
H	-1.36443800	14.33089800	11.34768600
H	-1.31206100	13.49237100	9.77760500
H	-0.65241700	15.13869800	9.93571100
C	0.36413800	12.22261700	11.50372000
H	1.30252700	11.69179700	11.70356400
H	-0.25356800	11.58062800	10.85693500
H	-0.17004200	12.35899100	12.45593900
C	1.15082900	14.73458400	13.03682600
C	0.27951500	15.76777000	13.45850900
C	0.01578800	15.90308900	14.83030200
H	-0.65490700	16.69558600	15.16923100
C	0.61396600	15.06678900	15.76811300
H	0.39376800	15.18672700	16.83209600
C	1.52353200	14.09961100	15.34598400
H	2.03072300	13.48140600	16.08983500
C	1.81918000	13.91949500	13.98767700
C	-0.29215400	16.80886200	12.50435400
H	-0.05119300	16.50483500	11.48036100
C	0.40254500	18.16070900	12.72979800
H	0.02213800	18.92046800	12.03294600
H	1.48762700	18.07513800	12.56420800
H	0.25872200	18.52609300	13.75911400
C	-1.81731600	16.95620900	12.60466800
H	-2.33252200	15.99205100	12.47339600
H	-2.17837500	17.64854500	11.82793100
H	-2.12424100	17.36666300	13.58009900

C	2.90184900	12.91340900	13.60794300
H	2.94017300	12.86400700	12.51119300
C	4.28488000	13.39637700	14.08280300
H	4.51241900	14.38729900	13.66551700
H	5.06924200	12.69179000	13.75980300
H	4.32685800	13.46756500	15.18194100
C	2.61493800	11.50225000	14.14988200
H	2.68958200	11.47262400	15.24881700
H	3.35106900	10.78314000	13.75501600
H	1.60958200	11.14861500	13.87797100
C	0.96697600	18.47906600	9.28693900
C	1.16277700	19.82799000	9.63742800
H	2.17120800	20.21069900	9.79736700
C	0.07539100	20.68684900	9.79728100
H	0.25769300	21.72591500	10.08051800
C	-1.22918500	20.21344800	9.61822800
H	-2.08239500	20.88309200	9.75274600
C	-1.43443900	18.87606400	9.26928100
H	-2.44839400	18.49459600	9.12424200
C	-0.34360900	18.01525100	9.10254200
H	-0.53674200	16.97789800	8.82776900
C	3.41110500	18.21042000	7.80275400
C	2.78256100	18.86497200	6.73075600
H	1.69375700	18.95665200	6.71052400
C	3.54305000	19.41405000	5.69482800
H	3.04364300	19.92510500	4.86737100
C	4.93864800	19.31470800	5.71626700
H	5.53135500	19.74869300	4.90683700
C	5.57265400	18.66564400	6.78008600
H	6.66292000	18.59161600	6.80743300
C	4.81192100	18.12260000	7.81946500
H	5.30839400	17.63612600	8.66335600
H	4.08787200	15.97674800	12.59521500
C	4.93481700	18.45851200	11.67993600

H	5.69062400	18.32812400	10.89702300
C	5.03306500	17.52793100	12.76172800
H	5.92842700	16.89514300	12.71576000
C	4.65684700	17.86619900	14.18126900
C	3.60087600	17.25798600	14.87789500
C	5.41251700	18.84973600	14.84689900
C	3.30627600	17.62254500	16.19612300
H	2.99668100	16.49817100	14.38115000
C	5.12262400	19.21242800	16.16310000
H	6.22680800	19.34376900	14.31070100
C	4.06371800	18.60064600	16.84438100
H	2.47370100	17.13435800	16.70832000
H	5.72142000	19.98195000	16.65802400
H	3.83090400	18.88862900	17.87306600
C	4.28450100	19.77617900	11.63210100
C	3.32912400	20.24897600	12.56512600
C	4.62295100	20.65361600	10.57037000
C	2.74823800	21.51106300	12.43260600
H	3.03369900	19.62221800	13.40451500
C	4.04426400	21.91549200	10.44446400
H	5.35233300	20.32367000	9.82600500
C	3.09648600	22.35945600	11.37591100
H	2.00970700	21.83421200	13.17168000
H	4.33394000	22.55764700	9.60788100
H	2.64187200	23.34868800	11.28090900

IN-10P:

Cr	3.62536400	16.25735400	11.10412100
P	2.47200700	17.00395100	9.15140300
N	1.33075400	14.28065000	11.61660100
C	2.29570300	14.80324900	10.78062500
C	1.99111800	14.26948500	9.35639300
C	0.75266900	13.35189600	9.55654200
H	0.03645700	13.40300400	8.72212600

H	1.08155700	12.30350300	9.63193000
C	0.11820700	13.77779400	10.90107100
C	1.72117200	15.46675500	8.39592700
H	0.64330700	15.63981200	8.27709300
H	2.12697900	15.30318100	7.38528200
C	3.19132200	13.46090400	8.82589900
H	4.07390700	14.10858000	8.69892600
H	2.95929600	12.99449900	7.85190100
H	3.47354000	12.66817500	9.53393600
C	-0.90656600	14.91154000	10.68220400
H	-1.39391500	15.19624300	11.62348000
H	-1.69385800	14.58285000	9.98494700
H	-0.43121700	15.80992700	10.26588300
C	-0.56843500	12.62565000	11.63698800
H	0.09749200	11.76295200	11.76779600
H	-1.44828100	12.29272900	11.06491400
H	-0.91255400	12.94488500	12.63279500
C	1.56698600	14.17250000	13.02474500
C	1.19118900	15.20579500	13.91559900
C	1.49456500	15.06211100	15.27914600
H	1.20354700	15.84887800	15.97926100
C	2.16851400	13.94104800	15.75604800
H	2.40095100	13.85133700	16.82044300
C	2.55180900	12.93659600	14.86737200
H	3.09456000	12.06677400	15.24449500
C	2.26202100	13.02853900	13.50049800
C	0.47982900	16.47011700	13.44848700
H	0.45692900	16.45175600	12.35104000
C	1.22774500	17.74868700	13.86020900
H	0.69401500	18.63722900	13.49073700
H	2.24489200	17.77232900	13.44251700
H	1.31321300	17.84269900	14.95425100
C	-0.96949000	16.51557700	13.96330400
H	-1.51596000	15.59123800	13.72176600

H	-1.51477700	17.36407900	13.51839900
H	-0.99517800	16.63330700	15.05919500
C	2.74463000	11.92989900	12.55946400
H	2.23600700	12.07650700	11.59951700
C	4.25012600	12.06252300	12.28155700
H	4.48419400	13.05294400	11.86759400
H	4.58048400	11.29422900	11.56249200
H	4.83667600	11.93570600	13.20570500
C	2.39240300	10.51988600	13.05779300
H	2.95394100	10.25312000	13.96762900
H	2.64457900	9.77013800	12.29023700
H	1.31970800	10.42583100	13.28998800
C	1.07225600	18.15077600	9.49159300
C	1.15550500	18.99458600	10.61109000
H	2.03962300	18.95095500	11.25008000
C	0.11998100	19.88447200	10.90972500
H	0.20070300	20.53497200	11.78422100
C	-1.01461800	19.94021700	10.09446500
H	-1.82778600	20.63104500	10.33104300
C	-1.10224100	19.11159600	8.97053400
H	-1.98244600	19.15650600	8.32397300
C	-0.06367800	18.22772200	8.66700000
H	-0.14116800	17.60062700	7.77541900
C	3.36177600	17.83667600	7.75732100
C	3.16036200	19.18291100	7.41508700
H	2.36101000	19.75464000	7.89117100
C	3.98640400	19.80712300	6.47390000
H	3.81888200	20.85741100	6.22117400
C	5.02218800	19.09656100	5.86151300
H	5.66874600	19.58723100	5.12971200
C	5.22903200	17.75233500	6.19400700
H	6.03881600	17.18892600	5.72321000
C	4.41050000	17.13145300	7.13860200
H	4.60153200	16.08837100	7.40880700

H	5.65199400	18.21991300	9.50898700
C	4.83790300	18.05942500	11.50212700
H	4.25899400	18.63668100	12.23913100
C	5.39066200	18.87724600	10.35715200
H	4.57958900	19.50891500	9.95531600
C	6.58853800	19.78144500	10.62145600
C	7.14255700	20.50647600	9.55085300
C	7.16500800	19.92766600	11.88973300
C	8.23467700	21.35413800	9.74232200
H	6.70346200	20.39706100	8.55353300
C	8.26136000	20.77615700	12.08653400
H	6.75458900	19.36398600	12.73002600
C	8.80028700	21.49328900	11.01596500
H	8.64962700	21.90867900	8.89575700
H	8.69762900	20.87333100	13.08469100
H	9.65708900	22.15477200	11.16950200
C	5.54777100	16.93648400	12.05552000
C	6.71261000	16.35180700	11.45132100
C	4.94614500	16.17518000	13.11877300
C	7.26546100	15.17805500	11.93870200
H	7.20207700	16.87920900	10.63044200
C	5.53736600	14.99423100	13.60778600
H	4.10029900	16.59938200	13.67095200
C	6.69554900	14.49097900	13.03208100
H	8.17289100	14.78483000	11.47077600
H	5.05569000	14.47446400	14.43943200
H	7.14831700	13.56878900	13.40213100

TS-10P:

Cr	3.54457500	16.09443800	11.47540500
P	3.04420600	16.40697700	9.11015400
N	0.61904200	15.31895100	12.10927000
C	1.76680900	15.26521900	11.38821500
C	1.51803800	14.33978200	10.18185700

C	0.10946600	13.73962800	10.42627900
H	-0.50701300	13.72542300	9.51461000
H	0.21192600	12.69529400	10.75627700
C	-0.55809000	14.57187300	11.54792500
C	1.57558300	15.25946800	8.92736700
H	0.69985000	15.92599400	8.92655300
H	1.55504100	14.69462500	7.98045000
C	2.56946900	13.21873800	10.08367000
H	3.58657400	13.60577400	9.92380600
H	2.32828400	12.53270200	9.25284400
H	2.59768600	12.63875700	11.01797800
C	-1.62145200	15.52917800	10.98668600
H	-2.02848700	16.16879200	11.78144600
H	-2.45385400	14.94903600	10.55893700
H	-1.21782900	16.17613700	10.19594100
C	-1.22441100	13.67886500	12.60074000
H	-0.52791100	12.92452800	12.98880200
H	-2.08038500	13.15089900	12.15223600
H	-1.59884000	14.27638800	13.44551600
C	0.53958600	16.13421300	13.29288800
C	0.21537600	17.51127000	13.19051900
C	0.11037700	18.26232400	14.37079700
H	-0.14458000	19.32251000	14.30719100
C	0.35182700	17.69263500	15.61771800
H	0.26280400	18.29607100	16.52494200
C	0.73908900	16.35709200	15.69951900
H	0.97121800	15.92545200	16.67591500
C	0.85244700	15.55891000	14.55209600
C	0.06434900	18.24030300	11.85854400
H	0.16824500	17.50136300	11.05502000
C	1.19784600	19.25994100	11.66788700
H	1.10528000	19.75607000	10.69059700
H	2.18335000	18.77687000	11.71410000
H	1.17960800	20.03603100	12.44791000

C	-1.29771500	18.93812400	11.70661100
H	-2.14103700	18.25248900	11.87254100
H	-1.39702500	19.36301700	10.69444900
H	-1.40186700	19.76983700	12.42200100
C	1.37526500	14.13374000	14.70934000
H	1.37859500	13.66835600	13.71481800
C	2.83363800	14.13726400	15.20336600
H	3.47106100	14.71137600	14.51824300
H	3.22730100	13.10837500	15.24731600
H	2.91703700	14.57370100	16.21209600
C	0.50114000	13.28338800	15.64736500
H	0.54952600	13.65412000	16.68406600
H	0.85251900	12.23862200	15.65733100
H	-0.55582600	13.28541400	15.34508300
C	2.51991300	18.00601300	8.36432900
C	3.07529700	19.18829500	8.87712300
H	3.75328000	19.14494800	9.73240000
C	2.74096100	20.42643500	8.32052100
H	3.17754400	21.33911500	8.73396700
C	1.83748200	20.49596000	7.25659700
H	1.56795500	21.46434500	6.82726200
C	1.27531300	19.32121000	6.74296300
H	0.57030500	19.37067700	5.90896400
C	1.62015700	18.08190400	7.28766400
H	1.18967500	17.17084400	6.86390300
C	4.31729700	15.84461200	7.88766200
C	5.42395700	16.68895000	7.67531700
H	5.44892800	17.67819300	8.14034700
C	6.49593000	16.27657700	6.88372000
H	7.34621300	16.94629700	6.73150200
C	6.48696900	15.00703300	6.29285800
H	7.32982300	14.67981900	5.67913300
C	5.39439000	14.16163800	6.49732500
H	5.37723300	13.16767300	6.04265900

C	4.31713600	14.57567100	7.28906200
H	3.48544800	13.88797000	7.44461500
H	5.53976700	15.38430500	13.47117000
C	5.61141300	16.68130600	11.73225300
H	6.22703800	16.78714000	10.83572800
C	5.48683600	15.40008700	12.37520600
H	3.89194000	14.86993400	12.52237900
C	6.07029300	14.17588900	11.75978500
C	6.31581300	13.04644800	12.56294000
C	6.34154900	14.07467800	10.38186500
C	6.81781400	11.86448100	12.01580200
H	6.10089400	13.09966600	13.63428600
C	6.84275300	12.89240400	9.83257700
H	6.14526100	14.91964000	9.72209500
C	7.08429300	11.77983600	10.64455100
H	7.00100300	11.00265500	12.66348600
H	7.03697000	12.84640700	8.75801200
H	7.47467300	10.85419500	10.21369700
C	5.13966500	17.89650900	12.37024500
C	4.32778000	17.87039100	13.54349900
C	5.38397700	19.17554500	11.79084600
C	3.78832500	19.04743600	14.07779100
H	4.14210900	16.93164000	14.06788400
C	4.85043800	20.33532300	12.33720400
H	6.01577700	19.23203300	10.89943700
C	4.03987600	20.28395500	13.48573100
H	3.15614600	18.98031500	14.96669700
H	5.06620300	21.29942000	11.86723700
H	3.61624900	21.19909400	13.90571300

IN-9P:

Cr	3.95434600	16.70463900	10.96485500
P	2.82058900	17.02204200	8.77479100
N	1.69488900	14.87328100	11.86081400

C	2.61890900	15.29260700	10.92971600
C	2.42691000	14.43534800	9.66084700
C	1.35405000	13.39405500	10.07179400
H	0.63938000	13.17490800	9.26325800
H	1.85340800	12.44619300	10.32429600
C	0.65053300	13.94073200	11.33575700
C	1.96082600	15.38196500	8.51735000
H	0.87679100	15.54577000	8.57093000
H	2.17540300	14.98269100	7.51233900
C	3.72967200	13.72866100	9.23710700
H	4.49137000	14.45173700	8.90297100
H	3.54372900	13.02722900	8.40476000
H	4.15685800	13.16757900	10.08197900
C	-0.65058700	14.68982900	10.99198300
H	-1.10658600	15.10820100	11.89989300
H	-1.37684900	14.00172200	10.53141300
H	-0.47227800	15.51673400	10.29323800
C	0.30518900	12.82218400	12.32579900
H	1.17450900	12.19014300	12.54457000
H	-0.48012400	12.18012300	11.89735200
H	-0.07151000	13.23310900	13.27463000
C	1.67677400	15.38622600	13.20009200
C	1.02404400	16.60770400	13.49885700
C	0.97205200	17.03046800	14.83584200
H	0.46636300	17.96725600	15.08220700
C	1.57413400	16.29161100	15.85089700
H	1.52064400	16.63640500	16.88698400
C	2.27669000	15.13025700	15.53509600
H	2.79171900	14.58307500	16.32798700
C	2.35408200	14.66392500	14.21602000
C	0.47470400	17.53161300	12.41761500
H	0.55507700	17.01057100	11.45711000
C	1.34372700	18.79644200	12.30335100
H	0.99879400	19.43307200	11.47488300

H	2.40046200	18.53692600	12.11827300
H	1.31486700	19.38870200	13.23217200
C	-0.99735700	17.91427500	12.63126800
H	-1.64119300	17.02778000	12.73481400
H	-1.36278900	18.50675900	11.77713000
H	-1.12958900	18.52693800	13.53759300
C	3.23398200	13.45785100	13.90653600
H	3.05902300	13.18361900	12.85746100
C	4.72066400	13.84041000	14.02496000
H	4.95997000	14.69613800	13.37622500
H	5.36040600	12.98861700	13.73798400
H	4.97381500	14.11732200	15.06225500
C	2.90901800	12.23723400	14.78236500
H	3.16796400	12.41642900	15.83844800
H	3.49108700	11.36192100	14.45043700
H	1.84086100	11.97397800	14.74330100
C	1.54427400	18.35170500	8.77250200
C	2.01716800	19.67836400	8.75649200
H	3.09055900	19.87051900	8.67268100
C	1.13265800	20.75350700	8.84675500
H	1.51948900	21.77558600	8.83487200
C	-0.24352200	20.52410900	8.95971400
H	-0.93701200	21.36495900	9.03727200
C	-0.72379400	19.21260000	8.97400500
H	-1.79626100	19.02095300	9.05978400
C	0.16341900	18.13449000	8.88082300
H	-0.24073600	17.12220700	8.90002800
C	3.68392000	17.33025400	7.16329700
C	5.06974600	17.11724600	7.10549800
H	5.60881600	16.81985700	8.01058600
C	5.76628800	17.28647300	5.90408500
H	6.84580700	17.11800700	5.87275700
C	5.08216800	17.67751000	4.75000300
H	5.62482600	17.81473800	3.81113600

C	3.70048700	17.89926100	4.79973500
H	3.16357100	18.20935100	3.89928100
C	3.00479500	17.72736900	5.99845900
H	1.92743800	17.91079100	6.03148100
H	5.07396600	16.70782400	12.25394900

IN-8N:

Cr	6.42679800	5.55784600	2.39901200
N	4.30471500	5.81695400	2.27315500
C	6.15052700	7.07084900	3.54658800
C	4.47395000	1.66370800	3.12254200
H	4.84238900	1.43457800	4.13476200
H	3.70870700	0.90950300	2.87739000
H	5.31160600	1.54197700	2.41884400
C	2.70664100	3.22527000	4.02918000
H	2.96222100	2.85219100	5.03403100
H	2.38800900	4.27079400	4.13632700
H	1.84687200	2.64431000	3.65689700
C	3.90961800	3.09077900	3.08134900
H	4.70028300	3.75448000	3.47541200
C	3.60398100	3.56981100	1.66210700
C	3.14347200	2.68055400	0.68296000
H	2.95442800	1.63931700	0.94931500
C	2.94105800	3.09392500	-0.63422200
H	2.58082500	2.38313100	-1.38181900
C	3.24125300	4.40464300	-1.00192500
H	3.13350400	4.70728300	-2.04602500
C	3.68928000	5.34056200	-0.06074300
C	3.80364800	4.92479900	1.28764200
C	4.09764200	6.73981300	-0.50748200
H	4.27620200	7.34326800	0.39404700
C	3.01390400	7.44532600	-1.33780600
H	3.31068600	8.48480600	-1.55137700
H	2.85457300	6.94543600	-2.30650400

H	2.04695600	7.46916900	-0.81232400
C	5.43122700	6.68620100	-1.27437100
H	5.75275600	7.69812600	-1.56892500
H	6.23138000	6.24729600	-0.65830400
H	5.33735000	6.07755200	-2.18840300
C	3.71960900	6.69533900	3.02127300
C	2.22951500	7.12426600	2.94864700
C	1.32263800	6.15433000	2.15840200
H	0.27990800	6.49200900	2.26424200
H	1.55385100	6.13493900	1.08846200
H	1.37825300	5.12485400	2.52941500
C	1.61176100	7.27193600	4.35698100
H	2.14526200	8.00088300	4.97847600
H	0.57415000	7.62567200	4.25460200
H	1.58499700	6.31178200	4.89286500
C	2.14604500	8.50416600	2.23807700
H	2.52121100	9.32217100	2.86373500
H	2.70088200	8.51035400	1.29016300
H	1.09104600	8.72235500	2.00839300
C	4.71227100	7.28601100	4.04743300
C	4.52995900	6.47382600	5.36858600
H	5.41279500	6.60801000	6.00402100
H	3.64733600	6.77847400	5.94803200
H	4.45595000	5.39878900	5.15222300
C	6.13182500	9.21731600	4.68123400
C	6.49077600	10.58471400	4.07786500
H	6.31543100	10.60552000	2.99476000
H	5.87247600	11.37229900	4.53725200
H	7.54717400	10.83083200	4.26236200
C	6.33357200	9.31991000	6.20644300
H	6.00898100	8.41731300	6.73761200
H	7.39562600	9.48874300	6.43634900
H	5.76111500	10.17141900	6.60722700
N	6.91419800	8.10325100	4.07461600

C	4.68658100	8.81440500	4.29186500
H	4.44362600	9.32143900	3.35018500
H	3.95942900	9.14333300	5.04927800
C	9.11485200	7.54773600	5.03206900
C	10.51102500	7.65845000	4.97960200
H	11.11459300	7.22094000	5.77831300
C	11.14401500	8.29495500	3.91394800
H	12.23392000	8.37854000	3.89233200
C	10.38311000	8.78663900	2.85617800
H	10.88781800	9.23144100	1.99511100
C	8.98344500	8.69519600	2.86211800
C	8.21655200	9.10393800	1.60901100
H	7.14473000	9.02083000	1.83495600
C	8.50939800	8.10994400	0.47018200
H	7.89404900	8.34036700	-0.41479000
H	9.56823800	8.15537300	0.16636100
H	8.30174400	7.07546000	0.78633200
C	8.51034400	10.54305200	1.15639600
H	7.86856800	10.81657200	0.30241100
H	8.33726000	11.27252100	1.96129200
H	9.55556600	10.65601100	0.82590900
C	8.47674900	6.71195600	6.13373400
H	7.39633900	6.88125400	6.08327700
C	8.94509000	7.09102800	7.54565500
H	8.79352000	8.16181900	7.75282700
H	8.38629500	6.51548900	8.30202300
H	10.01434400	6.87024600	7.69569900
C	8.70478900	5.21590500	5.85083200
H	8.17365800	4.59541800	6.59284100
H	8.34527800	4.95041400	4.84503400
H	9.77713200	4.96375300	5.90335100
C	8.34377000	8.12451700	3.99097000
H	8.02808700	4.97802800	2.25542600

TS-8N:

Cr	6.54688100	5.35494700	2.58319500
N	4.40972600	5.73194800	2.19964100
C	6.23303200	6.99928100	3.58300000
C	4.15940500	1.51183900	2.88877800
H	4.53203000	1.20746600	3.87954700
H	3.28614500	0.87892100	2.66257800
H	4.94090900	1.28770200	2.14750200
C	2.65249800	3.25269000	3.89995000
H	2.89706800	2.81781700	4.88264800
H	2.46670400	4.32310000	4.05122700
H	1.71535500	2.79177200	3.54711700
C	3.79541400	3.00391200	2.90192800
H	4.67763300	3.54549500	3.28738600
C	3.51625900	3.56938500	1.50832600
C	2.95092500	2.77025000	0.50846600
H	2.66983500	1.74120600	0.73826400
C	2.75309800	3.25624300	-0.78599900
H	2.31158500	2.61370500	-1.55141100
C	3.16220400	4.54857100	-1.10505400
H	3.05366100	4.90765000	-2.13080900
C	3.71891100	5.39927600	-0.14050500
C	3.84224000	4.91264800	1.18116100
C	4.17211200	6.80022300	-0.54096700
H	4.45247400	7.33796100	0.37714400
C	3.05792400	7.60055000	-1.23889300
H	3.37808200	8.64274200	-1.40088000
H	2.82114500	7.17568900	-2.22726100
H	2.12634000	7.61848300	-0.65423600
C	5.42501300	6.74934800	-1.42949200
H	5.75116800	7.76983900	-1.68937600
H	6.25604000	6.23388800	-0.93244500
H	5.22444300	6.20901500	-2.36679000
C	3.81870800	6.59798400	2.95934500

C	2.32768000	7.05645900	2.88559400
C	1.39364200	6.14143700	2.05884500
H	0.36315100	6.50962200	2.18259500
H	1.62023600	6.15268200	0.98922800
H	1.41509400	5.09900200	2.39116100
C	1.69366100	7.15897700	4.29294900
H	2.22720400	7.84999000	4.95524100
H	0.66588900	7.53931500	4.18850200
H	1.63481200	6.17878700	4.78788700
C	2.26844300	8.46852300	2.23208100
H	2.54029200	9.26775400	2.93099600
H	2.91761600	8.54438200	1.35044100
H	1.23644200	8.66406200	1.90190300
C	4.77209100	7.17582700	4.03764900
C	4.59373500	6.32747500	5.33483400
H	5.46923700	6.45188300	5.98340600
H	3.70068600	6.61134700	5.90711300
H	4.53182600	5.25826700	5.09020500
C	6.12340800	9.12766100	4.73780000
C	6.48748400	10.49698500	4.14354100
H	6.34047800	10.51968400	3.05636800
H	5.84709400	11.27485000	4.58795500
H	7.53550000	10.75618100	4.35600000
C	6.27815500	9.22666400	6.26426500
H	6.00039900	8.29991700	6.78006000
H	7.31488100	9.47236400	6.52813200
H	5.63486500	10.03382900	6.64777000
N	6.93876900	8.02463100	4.13147800
C	4.70293200	8.69826400	4.30853700
H	4.46916800	9.21698700	3.37220400
H	3.95052300	8.99364000	5.05330900
C	9.19389400	7.64153000	5.08027700
C	10.58219500	7.80617000	4.96403800
H	11.22975100	7.45293300	5.76936900

C	11.15438400	8.38016900	3.83224500
H	12.23890400	8.49867300	3.76431800
C	10.33966500	8.76757000	2.77169400
H	10.79594600	9.16908300	1.86437200
C	8.94561100	8.63379100	2.83986800
C	8.12079700	8.96886500	1.60223800
H	7.05910300	8.92173500	1.87765500
C	8.34252200	7.89622000	0.52576500
H	7.75039300	8.10965100	-0.37596500
H	9.40203200	7.82902600	0.23150700
H	8.04356400	6.90383700	0.89383300
C	8.40580600	10.37367100	1.04882700
H	7.71484900	10.60558700	0.22199700
H	8.28904800	11.14851300	1.82187600
H	9.42891600	10.45435500	0.64828900
C	8.65920700	6.89091700	6.29625800
H	7.56529900	6.88677700	6.22594400
C	9.06176200	7.55009600	7.62678000
H	8.79877700	8.61735600	7.66413500
H	8.56260000	7.04638700	8.47062800
H	10.14759200	7.47260000	7.79763500
C	9.11373000	5.41919400	6.27955900
H	8.67785000	4.87745600	7.13556700
H	8.79182900	4.92316700	5.35392800
H	10.21015000	5.33653700	6.35221500
C	8.37017400	8.10780100	4.02324000
H	7.55227900	5.16403600	3.84258500
C	7.03453800	3.56078500	1.36063200
H	6.13143200	2.95530900	1.46939800
C	7.88559200	3.59879900	2.50057700
H	7.55950800	2.94596000	3.32117000
C	9.38273800	3.70824200	2.42463400
C	10.10636600	4.87060100	2.73407500
C	10.10352500	2.57451400	2.00365900

C	11.50088000	4.90062500	2.62695700
H	9.56820200	5.76202000	3.05501700
C	11.49551500	2.59981600	1.89841100
H	9.55371400	1.66613300	1.74296300
C	12.20223800	3.76706200	2.20932900
H	12.03272400	5.82267200	2.87272300
H	12.03133900	1.70616200	1.56633000
H	13.29196700	3.79132600	2.12337900
C	7.34651300	3.85341300	-0.04357900
C	8.49311800	4.55006400	-0.49684600
C	6.43757800	3.40553200	-1.03347900
C	8.70225900	4.78994900	-1.85600000
H	9.22949500	4.90808400	0.21985100
C	6.65590400	3.63788500	-2.38893000
H	5.54058200	2.86832700	-0.71937500
C	7.79079100	4.33733000	-2.81620900
H	9.59723200	5.33631300	-2.16730500
H	5.92611100	3.27576800	-3.11889300
H	7.96248800	4.52600600	-3.87891400

IN-9N:

Cr	6.32438400	5.53090300	2.11983800
N	4.17270400	5.78855200	2.04087100
C	6.07286700	6.95580800	3.42522200
C	3.92349400	1.55921500	2.56245500
H	4.34033300	1.20975300	3.52016900
H	3.04878900	0.92700800	2.34049500
H	4.67380400	1.38066300	1.77736700
C	2.42832000	3.23033200	3.70795500
H	2.70341300	2.74389400	4.65771300
H	2.24333700	4.29024500	3.92257700
H	1.48374200	2.78364900	3.35671500
C	3.54259700	3.04425600	2.66496600
H	4.42774700	3.58066200	3.04932500

C	3.21398200	3.66808400	1.30832800
C	2.60169400	2.91529200	0.29907200
H	2.29957500	1.88663800	0.50307500
C	2.39226600	3.44576900	-0.97529800
H	1.91590400	2.83951500	-1.74949900
C	2.83283100	4.73547600	-1.26705200
H	2.71492200	5.12639900	-2.28004100
C	3.42451000	5.54187100	-0.28607300
C	3.55539200	5.01253200	1.01703800
C	3.94002900	6.93191800	-0.64275200
H	4.18137800	7.45192200	0.29573000
C	2.90026200	7.77968400	-1.39260700
H	3.26839100	8.81017600	-1.52269200
H	2.69747000	7.37735300	-2.39791900
H	1.94199700	7.82575700	-0.85259500
C	5.25027000	6.83520900	-1.44189200
H	5.63767900	7.84052000	-1.67376500
H	6.02617700	6.29021100	-0.88628600
H	5.09501600	6.29875600	-2.39112400
C	3.61966500	6.61595100	2.87046100
C	2.13289200	7.09173500	2.85823000
C	1.15902400	6.22277100	2.02695400
H	0.13927000	6.60272900	2.19571900
H	1.35306800	6.27391900	0.95121700
H	1.17089000	5.16798100	2.31851000
C	1.54008000	7.16167200	4.28450000
H	2.10897200	7.81577500	4.95411200
H	0.51870700	7.56777900	4.22217200
H	1.47246500	6.16622200	4.74698400
C	2.08020200	8.52022400	2.24286300
H	2.44656700	9.29103300	2.92963900
H	2.65510700	8.58604700	1.30961800
H	1.03286800	8.76453300	2.00578500
C	4.62468500	7.12234800	3.92758500

C	4.45698800	6.21106800	5.18467200
H	5.34391200	6.30737500	5.82173400
H	3.57391500	6.45688900	5.79097800
H	4.39592800	5.15566100	4.88422800
C	6.00079800	9.01622300	4.73631400
C	6.36507600	10.42472600	4.23595800
H	6.21622700	10.52115000	3.15348000
H	5.72780700	11.17412400	4.73149800
H	7.41380200	10.66727800	4.46460500
C	6.14208500	9.03290900	6.27249500
H	5.80952600	8.09772700	6.73857800
H	7.18821600	9.21041400	6.56080700
H	5.53550500	9.85042400	6.69316700
N	6.80712800	7.94541700	4.07977800
C	4.57724600	8.62910000	4.27869500
H	4.35908500	9.19548000	3.36593000
H	3.82548200	8.90280700	5.03303900
C	9.03160900	7.48956100	5.02844900
C	10.42204300	7.66269600	4.96965200
H	11.05002700	7.25055000	5.76228700
C	11.02097400	8.32165700	3.89769800
H	12.10636100	8.44845100	3.86819500
C	10.23365400	8.78711100	2.84724100
H	10.71443700	9.26105600	1.98821600
C	8.83782800	8.64977700	2.86669700
C	8.03605500	9.11608400	1.65471000
H	6.97185500	9.03205300	1.91018400
C	8.26607600	8.18713600	0.45207400
H	7.66939400	8.51466900	-0.41391600
H	9.32549900	8.17318600	0.14801400
H	7.97397800	7.15433700	0.69001300
C	8.33129700	10.57518400	1.27030000
H	7.64684000	10.90777800	0.47256900
H	8.21631400	11.25641400	2.12635700

H	9.35772300	10.69357400	0.88727200
C	8.43331000	6.63072000	6.13688900
H	7.35474800	6.81269100	6.13893000
C	8.96981400	6.97330800	7.53354600
H	8.87405200	8.04788900	7.75520000
H	8.41345400	6.41505900	8.30401700
H	10.03249400	6.70315100	7.64365000
C	8.61731100	5.13555300	5.81954300
H	8.18571600	4.51526600	6.62301600
H	8.11213200	4.87925200	4.87817800
H	9.68317200	4.87240300	5.72102400
C	8.23067800	8.03222600	3.98998500
H	9.79914800	6.06897900	1.58340300
C	8.29286600	4.60528800	2.07560700
H	8.06347500	3.83406900	2.83193100
C	9.64623500	5.23970200	2.29296700
H	9.65489600	5.73246400	3.27264000
C	10.86989000	4.33452800	2.22721700
C	12.11782200	4.84549500	2.62983800
C	10.81570800	3.00575000	1.78305400
C	13.27055600	4.05955800	2.58712800
H	12.17332100	5.87848200	2.98480600
C	11.96947100	2.21372300	1.73713300
H	9.86001100	2.58513900	1.46548300
C	13.20159700	2.73507800	2.13826700
H	14.22836600	4.47967700	2.90757200
H	11.90166800	1.18061500	1.38437500
H	14.10201700	2.11587100	2.10380000
C	7.86109400	4.21365200	0.74479500
C	8.49358800	4.63865000	-0.46495400
C	6.63419000	3.49873100	0.59438700
C	7.94281200	4.35542600	-1.70546800
H	9.44631700	5.16680500	-0.40696500
C	6.08277700	3.22698600	-0.66903100

H	6.17198800	3.05139700	1.48160200
C	6.72683000	3.65349800	-1.82432500
H	8.46726900	4.68186400	-2.60835400
H	5.14264200	2.67628400	-0.73326500
H	6.30009000	3.44518800	-2.80788000

TS-9N:

Cr	3.26169100	16.82440100	10.60432100
N	1.13883600	15.16765300	12.20401500
C	1.93677800	15.50557700	11.13470200
C	1.59033500	14.51768600	9.98651600
C	0.39464900	13.69183400	10.55259500
H	-0.42230300	13.50322300	9.85215900
H	0.76834700	12.70465500	10.86292800
C	-0.10410400	14.45109500	11.80029700
C	1.45413600	15.33479000	8.67686900
C	2.81713100	13.57284700	9.78549400
H	3.69304100	14.12817000	9.42090500
H	2.60366000	12.75465100	9.08103000
H	3.09185300	13.12823700	10.75120800
C	-1.20251200	15.46379100	11.42057300
H	-1.52819700	16.03496100	12.29923700
H	-2.08419200	14.94631500	11.00956500
H	-0.83408800	16.17534100	10.66729500
C	-0.64652800	13.51443400	12.88235800
H	0.08398900	12.74664600	13.16841000
H	-1.54793500	13.00415000	12.50872900
H	-0.92347800	14.07577200	13.78710300
C	1.42733000	15.51066700	13.56362900
C	1.02659500	16.74716600	14.11777300
C	1.26400600	16.97938000	15.48268000
H	0.95059000	17.92756100	15.92622600
C	1.90977200	16.03655900	16.27620300
H	2.08373200	16.23685500	17.33682000

C	2.35643300	14.84467000	15.70555300
H	2.89567000	14.12474900	16.32478500
C	2.13029800	14.56189500	14.35317900
C	0.43990400	17.87506400	13.27860800
H	0.33982100	17.51009900	12.24817900
C	1.42281500	19.05682700	13.23966400
H	1.05464200	19.86422300	12.58952100
H	2.39609900	18.72799300	12.85116500
H	1.58497600	19.47991900	14.24436400
C	-0.94322400	18.33053200	13.76884000
H	-1.65244000	17.49111200	13.83900500
H	-1.36798100	19.08098700	13.08180400
H	-0.88449400	18.79403300	14.76702500
C	2.71582600	13.29497600	13.74012300
H	2.17449800	13.10515800	12.80473400
C	4.18646700	13.53265300	13.35891900
H	4.27711400	14.38957600	12.67757900
H	4.61665200	12.64649700	12.86267700
H	4.79341100	13.74705400	14.25406100
C	2.55882200	12.04918600	14.62269800
H	3.17528100	12.10841500	15.53412200
H	2.88354600	11.15072500	14.07308200
H	1.51319900	11.90069900	14.93661600
H	5.26854800	18.30429700	12.25720200
C	4.84417500	18.39249400	10.15100000
H	5.18767700	18.00919700	9.18864800
C	5.25943800	17.73027500	11.32698700
H	3.93822000	16.84658000	12.12492800
C	6.24217300	16.60747900	11.33310200
C	6.54976100	15.97069700	12.55070100
C	6.88868600	16.15045600	10.17182200
C	7.45532500	14.91270200	12.60445400
H	6.04331000	16.30033000	13.46156600
C	7.78884800	15.08212600	10.22041000

H	6.70813200	16.64936700	9.22211400
C	8.07553500	14.45468700	11.43552900
H	7.67129900	14.43355800	13.56293000
H	8.27806100	14.74813000	9.30116100
H	8.78074100	13.62063200	11.47467700
C	4.29369800	19.75100400	10.10619600
C	3.97567300	20.50447700	11.25936300
C	4.05110500	20.35985600	8.85374100
C	3.42935600	21.78456100	11.16123900
H	4.15315800	20.08432500	12.25052200
C	3.51252400	21.64108500	8.75754900
H	4.28105100	19.80504700	7.94300400
C	3.19047100	22.36637500	9.91093700
H	3.18966400	22.33526100	12.07523900
H	3.33416000	22.07235000	7.76862800
H	2.76278800	23.36933700	9.83758400
N	2.21529100	16.38575600	8.70932800
C	0.53799500	14.86416200	7.50396700
C	0.68821000	13.33787400	7.28218200
C	-0.94359600	15.17971100	7.84811500
C	0.79635700	15.52493400	6.12852200
H	1.69828000	13.08305000	6.92954900
H	0.48026700	12.74081100	8.17678200
H	-0.02139800	13.02043100	6.50267700
H	-1.30728500	14.66585300	8.74214900
H	-1.10153900	16.25613800	7.99069900
H	-1.57513800	14.86157900	7.00367300
H	0.16125200	15.01642200	5.38606900
H	0.53503500	16.58784400	6.10969700
H	1.83354000	15.42882800	5.79564700
C	2.39525500	17.35257700	7.68064900
C	1.47345800	18.42147000	7.57161700
C	3.54399400	17.27529400	6.85373400
C	1.66636000	19.35310900	6.54418000

C	3.67393700	18.22228900	5.82919300
C	2.73678000	19.24225800	5.65860000
H	0.96638700	20.18281500	6.43410500
H	4.53074800	18.17447300	5.15599400
H	2.86066300	19.96999100	4.85311300
C	4.62065700	16.21494500	7.08829300
C	6.01616400	16.67218100	6.63972100
C	4.30370900	14.84594200	6.46412300
H	4.66300300	16.05524300	8.17941700
H	6.27342000	17.66438600	7.04127500
H	6.77700200	15.95402100	6.98231500
H	6.09544200	16.72297600	5.54208900
H	3.38599400	14.41127600	6.87681800
H	4.18717500	14.92597800	5.37095600
H	5.12202700	14.13671900	6.66809300
C	0.31754300	18.56987100	8.55541400
C	-0.96788500	19.10743700	7.91225800
C	0.72506200	19.42090900	9.76726500
H	0.08864000	17.57098100	8.94851300
H	-1.24183900	18.53945000	7.00875500
H	-1.80426800	19.04056400	8.62629900
H	-0.87201300	20.16709300	7.62685500
H	1.58468300	18.98130100	10.29259500
H	1.01765300	20.43828700	9.46835000
H	-0.10763200	19.48834800	10.48551700
